# Supplementary material for: Autonomous closed-loop mechanistic investigation of molecular electrochemistry via automation
Source: Nat Commun. 2024 Mar 30;15:2781. doi: 10.1038/s41467-024-47210-x (PMC10981680; doi:10.1038/s41467-024-47210-x)
Supplement: Supplementary file 1 — Supplementary Information [file 41467_2024_47210_MOESM1_ESM.pdf]

Supplementary Information

Autonomous closed-loop mechanistic investigation of molecular electrochemistry via automation

Hongyuan Sheng,[1,†,*] Jingwen Sun,[1,†] Oliver Rodríguez,[2,3,4] Benjamin B. Hoar,[1] Weitong Zhang,[5] Danlei Xiang,[1] Tianhua Tang,[6] Avijit Hazra,[6] Daniel S. Min,[1] Abigail G. Doyle,[1] Matthew S. Sigman,[6] Cyrille Costentin,[7] Quanquan Gu,[5] Joaquín Rodríguez-López,[2,3,4] Chong Liu[1,8,*]

[1] Department of Chemistry and Biochemistry, University of California, Los Angeles, Los Angeles, California 90095, United States

[2] Department of Chemistry, University of Illinois at Urbana−Champaign, Urbana, Illinois 61801, United States

[3] Beckman Institute for Advanced Science and Technology, University of Illinois at Urbana−Champaign, Urbana, Illinois 61801, United States

[4] Joint Center for Energy Storage Research (JCESR), Argonne National Laboratory, Lemont, Illinois 60439, United States

[5] Department of Computer Science, University of California Los Angeles, Los Angeles, California 90095, United States

[6] Department of Chemistry, University of Utah, Salt Lake City, Utah 84112, United States

[7] Université Grenoble Alpes, DCM, CNRS, 38000 Grenoble, France

[8] California NanoSystems Institute, University of California, Los Angeles, Los Angeles, California 90095, United States

[†] These authors contributed equally to this work

* Corresponding author. Email: hsheng7@g.ucla.edu (H. S.), chongliu@chem.ucla.edu (C. L.)

Index Page

Supplementary Note 1. Hardware and software specifications for our constructed autonomous electrochemical platform. S7

Supplementary Fig. 1. Schematic of the autonomous electrochemical platform. S7

Supplementary Fig. 2. Annotated photographs of the autonomous electrochemical platform. S8

Supplementary Table 1. Hardware specifications of the autonomous electrochemical platform. S8

Supplementary Fig. 3. Instructions on how to assemble the flow system. S9

Supplementary Table 2. Software specifications of the autonomous electrochemical platform. S10

Supplementary Note 2. Standard operating procedures (SOPs) for our constructed autonomous electrochemical platform. S11

Supplementary Fig. 4. Schematic of the setup for flow rate check. S11

Supplementary Table 3. A demonstration of flow rate check using deionized water as the solvent. S11

Supplementary Fig. 5. Routine flow rate checks using dimethylformamide (DMF) as the solvent. S12

Supplementary Fig. 6. Detailed protocols for automated electrolyte formulation and disposal. S13

Supplementary Fig. 7. Calibration of the nonaqueous Ag/Ag^{+} reference electrode potential**.** S14

Supplementary Fig. 8. Detailed protocols for automated electrochemical testing. S14

Supplementary Note 3. The parameter space for automated exhaustive CV experiments of cobalt tetraphenylporphyrin (CoTPP) with 1-bromobutane (*n*-BuBr). S18

Supplementary Table 4. The stock solutions used for automated exhaustive CV experiments. S18

Supplementary Table 5. Parameterization of scan rates for automated exhaustive CV experiments. S18

Supplementary Table 6. Five prototypical mechanisms in molecular electrochemistry included in the deep-learning (DL) model. S20

Supplementary Fig. 9. Summary of all the DL-generated mechanism propensity values obtained from automated exhaustive CV experiments of CoTPP with *n*-BuBr. S21

Supplementary Note 4. Bayesian optimization of the DL-generated propensity of an *EC* mechanism for a model system of CoTPP with *n*-BuBr. S22

Supplementary Fig. 10. A 20-step campaign of Bayesian optimization. S22

Supplementary Fig. 11. Detailed results from a 20-step campaign of Bayesian optimization. S23

Supplementary Fig. 12. Three replicates of a 15-step campaign of Bayesian optimization. S24

Supplementary Fig. 13. A 10-step campaign of Bayesian optimization. S25

Supplementary Fig. 14. The step-number-dependence of Bayesian optimization. S25

Supplementary Note 5. Extracting kinetic information of the *C* step in an *EC* mechanism from the reverse-to-forward peak current ratio of a voltammogram. S26

Supplementary Table 7. An *EC* mechanism with a monomolecular versus a bimolecular *C* step. S26

Supplementary Table 8. A working curve for kinetic analysis of a monomolecular *C* step in an *EC* mechanism. S26

Supplementary Fig. 15. Generation of an empirical equation that matches with the working curve. S27

Supplementary Fig. 16. A flowchart illustrating the design of a closed-loop workflow for autonomous investigations of an *EC* mechanism between CoTPP and a library of organohalide (RX) substrates. S29

Supplementary Note 6. Introduction of additional RX reservoirs in the flow chemistry module to expand the accessible range of [RX] for a generally applicable closed-loop workflow. S30

Supplementary Fig. 17. The modified autonomous electrochemical platform with additional RX reservoirs. S30

Supplementary Table 9. Hardware modification of the flow chemistry module. S30

Supplementary Fig. 18. Instructions on how to modify the flow chemistry module. S31

Supplementary Table 10. Derivation of the accessible range of [RX] for the modified platform. S32

Supplementary Fig. 19. Annotated photographs of the modified platform. S32

Supplementary Fig. 20. Modified protocols for automated electrolyte formulation and disposal when three RX reservoirs are used. S33

Supplementary Fig. 21. Modified protocols for automated electrolyte formulation and disposal when only two RX reservoirs are used. S34

Supplementary Note 7. Determination of a cutoff RX concentration, $[RX]_{cutoff}$, for electrokinetic analysis in Stage II of the closed-loop workflow. S35

Supplementary Fig. 22. The created response surface of the *EC* mechanism propensity right after Stage I of the closed-loop workflow when RX = *n*-BuBr. S35

Supplementary Note 8. Design of suitable parameter combinations for electrokinetic analysis in Stage II of the closed-loop workflow. S37

Supplementary Fig. 23. Autonomous investigation of CH_3CN (acetonitrile) with an undetected reactivity toward CoTPP in the parameter space. S39

Supplementary Fig. 24. Autonomous discernment of an *EC* mechanism between CoTPP and *n*-BuBr based on a 15-step campaign of Bayesian optimization in Stage I of the closed-loop workflow. S40

Supplementary Fig. 25. On-the-fly update of the response surface of i_{pa}/i_{pc} during Stage II of the closed-loop workflow when RX = *n*-BuBr. S41

Supplementary Fig. 26. CV data measured in Stage I and Stage II of the closed-loop workflow when RX = *n*-BuBr. S42

Supplementary Note 9. *In silico* analysis of a hypothetical scenario where the response surface of i_{pa}/i_{pc} was not updated on-the-fly during Stage II of the closed-loop workflow when RX = *n*-BuBr. S43

Supplementary Fig. 27. A "ground-truth" response surface of i_{pa}/i_{pc} constructed based on all the cumulative CV data measured in Stage I and Stage II. S43

Supplementary Table 11. Determination of the "ground-truth" range of v values to satisfy $i_{pa}/i_{pc} \in [0.65, 0.75]$ under each assigned [*n*-BuBr] value in Stage II. S44

Supplementary Fig. 28. Three replicates of autonomous investigation of *n*-BuBr by the closed-loop workflow. S46

Supplementary Fig. 29. Autonomous investigation of *n*-BuI (1-iodobutane) that reacts with CoTPP following an *EC* mechanism. S47

Supplementary Fig. 30. Autonomous investigation of *n*-BuCl (1-chlorobutane) with an undetected reactivity toward CoTPP in the parameter space. S48

Index (continued) *Page*

Supplementary Fig. 31. Autonomous investigation of *n*-HexBr (1-bromohexane) that reacts with CoTPP following an *EC* mechanism. S49

Supplementary Fig. 32. Autonomous investigation of *n*-OctBr (1-bromooctane) that reacts with CoTPP following an *EC* mechanism. S50

Supplementary Fig. 33. Autonomous investigation of *i*-BuBr (1-bromo-2-methylpropane) that reacts with CoTPP following an *EC* mechanism. S51

Supplementary Fig. 34. Autonomous investigation of 2-BuBr (2-bromobutane) that reacts with CoTPP following an *EC* mechanism. S52

Supplementary Fig. 35. Autonomous investigation of Me_3CCH_2I (neopentyl iodide) that reacts with CoTPP following an *EC* mechanism. S53

Supplementary Fig. 36. Autonomous investigation of CH_2Cl_2 (dichloromethane) that reacts with CoTPP following an *EC* mechanism. S54

Supplementary Fig. 37. Autonomous investigation of $ClCH_2CN$ (chloroacetonitrile) that reacts with CoTPP following an *EC* mechanism. S55

Supplementary Fig. 38. Autonomous investigation of $Cl(CH_2)_2CN$ (3-chloropropionitrile) that reacts with CoTPP following an *EC* mechanism. S56

Supplementary Fig. 39. Autonomous investigation of $Cl(CH_2)_4CN$ (5-chlorovaleronitrile) that reacts with CoTPP following an *EC* mechanism. S57

Supplementary Fig. 40. Autonomous investigation of *n*-BuBr-d_9 that reacts with CoTPP following an *EC* mechanism. S58

Supplementary Fig. 41. Autonomous investigation of *n*-BuI-d_9 that reacts with CoTPP following an *EC* mechanism. S59

Supplementary Note 10. Extracting kinetic information of the *C* step between CoTPP and benzyl bromide substrates with fast rates using an alternative approach based on the forward peak position. S60

Supplementary Fig. 42. Method validation for determination of k_0 using the E_{pc} approach (part 1). S61

Supplementary Fig. 43. Method validation for determination of k_0 using the E_{pc} approach (part 2). S62

Supplementary Table. 12. Comparisons of k_0 determined by the E_{pc} approach with k_0 determined by the i_{pa}/i_{pc} approach for six representative RX substrates. S62

Supplementary Fig. 44. The revised workflow for kinetic analysis of the *C* step between CoTPP and highly reactive RX substrates using the E_{pc} approach. S63

Supplementary Fig. 45. Autonomous investigation of $PhCH_2Br$ (benzyl bromide) that reacts with CoTPP following an *EC* mechanism (replicate #1). S64

Supplementary Fig. 46. Autonomous investigation of $PhCH_2Br$ (benzyl bromide) that reacts with CoTPP following an *EC* mechanism (replicate #2). S65

Supplementary Fig. 47. Autonomous investigation of $PhCD_2Br$ (benzyl bromide-α,α-d_2) that reacts with CoTPP following an *EC* mechanism. S66

Supplementary Fig. 48. Determination of k_0 for $PhCH_2Br$ and $PhCD_2Br$ using the E_{pc} approach. S67

Supplementary Fig. 49. Autonomous investigation of *p*-MeO-$PhCH_2Br$ (4-methoxybenzyl bromide) that reacts with CoTPP following an *EC* mechanism. S68

Index (continued) *Page*

Supplementary Fig. 50. Autonomous investigation of *p*-PhO-$PhCH_2Br$ (4-phenoxybenzyl bromide) that reacts with CoTPP following an *EC* mechanism. S69

Supplementary Fig. 51. Autonomous investigation of *p*-Me-$PhCH_2Br$ (4-methylbenzyl bromide) that reacts with CoTPP following an *EC* mechanism. S70

Supplementary Fig. 52. Autonomous investigation of *p*-F-$PhCH_2Br$ (4-fluorobenzyl bromide) that reacts with CoTPP following an *EC* mechanism (replicate #1). S71

Supplementary Fig. 53. Autonomous investigation of *p*-F-$PhCH_2Br$ (4-fluorobenzyl bromide) that reacts with CoTPP following an *EC* mechanism (replicate #2). S72

Supplementary Fig. 54. Autonomous investigation of *p*-F_3CO-$PhCH_2Br$ (4-(trifluoromethoxy)benzyl bromide) that reacts with CoTPP following an *EC* mechanism. S73

Supplementary Fig. 55. Autonomous investigation of *p*-F_3C-$PhCH_2Br$ (4-trifluoromethylbenzyl bromide) that reacts with CoTPP following an *EC* mechanism. S74

Supplementary Fig. 56. Autonomous investigation of *p*-MeO_2C-$PhCH_2Br$ (methyl 4-(bromomethyl) benzoate) that reacts with CoTPP following an *EC* mechanism. S75

Supplementary Fig. 57. Autonomous investigation of *p*-PhOC-$PhCH_2Br$ (4-(bromomethyl) benzophenone) that reacts with CoTPP following an *EC* mechanism. S76

Supplementary Fig. 58. Autonomous investigation of *p*-NC-$PhCH_2Br$ (4-cyanobenzyl bromide) that reacts with CoTPP following an *EC* mechanism. S77

Supplementary Fig. 59. Determination of k_0 for *para*-substituted primary benzyl bromide substrates using the E_{pc} approach (part 1). S78

Supplementary Fig. 60. Determination of k_0 for *para*-substituted primary benzyl bromide substrates using the E_{pc} approach (part 2). S79

Supplementary Table 13. Various polar- and radical-derived Hammett parameters for the *para*-substituted primary benzyl bromide substrates studied in this work. S80

Supplementary Fig. 61. Hammett plots of *para*-substituted primary benzyl bromide substrates using polar-derived Hammett parameters. S81

Supplementary Fig. 62. Hammett plots of *para*-substituted primary benzyl bromide substrates using radical-derived Hammett parameters. S82

Supplementary Fig. 63. Autonomous investigation of $PhCH(CH_3)Br$ (1-bromoethyl)benzene) that reacts with CoTPP following an *EC* mechanism. S83

Supplementary Note 11. Possible pathways for oxidative addition of RX electrophiles to Co^{I}TPP, out of which a S_N2-type pathway is supported by our studies of benzyl bromide substrates and beyond. S84

Supplementary Fig. 64. Possible pathways for oxidative addition of RX electrophiles to Co^{I}TPP. S84

Supplementary Table 14. Comparisons of relative reaction rates between Co^{I}TPP and RX substrates with respect to leaving group the same alkyl group but different leaving groups). S86

Supplementary Table 15. Comparisons of relative reaction rates between Co^{I}TPP and RX substrates with respect to alkyl group (the same leaving group but different alkyl groups). S87

Supplementary Fig. 65. Autonomous investigation of $Cl(CH_2)_3CN$ (3-chlorobutyronitrile) that reacts with CoTPP but does not follow an *EC* mechanism. S88

Index (continued) *Page*

Supplementary Fig. 66. Autonomous investigation of $Br(CH_2)_3CN$ (4-bromobutyronitrile) that reacts with CoTPP following an *EC* mechanism. S89

Supplementary Fig. 67. Autonomous investigation of $I(CH_2)_3CN$ (4-iodobutyronitrile) that reacts with CoTPP following an *EC* mechanism. S90

Supplementary Note 12. Additional manual experiments to confirm $Cl(CH_2)_3CN$ as a mechanistic outlier, with the exclusion of potential artifacts. S91

Supplementary Fig. 68. Exclusion of the potential artifact concerning chemical purity. S91

Supplementary Fig. 69. Exclusion of other potential artifacts concerning automated electrolyte formulation or electrode fouling. S92

Supplementary Note 13. Mechanistic insights into the existence of $Cl(CH_2)_3CN$ as a mechanistic outlier that reacts with CoTPP but does not follow an *EC* mechanism. S93

Supplementary Fig. 70. Further analysis of the CV responses of CoTPP with $Cl(CH_2)_3CN$. S93

Supplementary Fig. 71. The CV responses of CoTPP with $Br(CH_2)_3CN$ and $I(CH_2)_3CN$. S94

Supplementary References S95

Supplementary Note 1. Hardware and software specifications for our constructed autonomous electrochemical platform.

General considerations. The schematic of our constructed autonomous electrochemical platform is reproduced below in Supplementary Fig. 1 (the same as Fig. 1c in the main text).

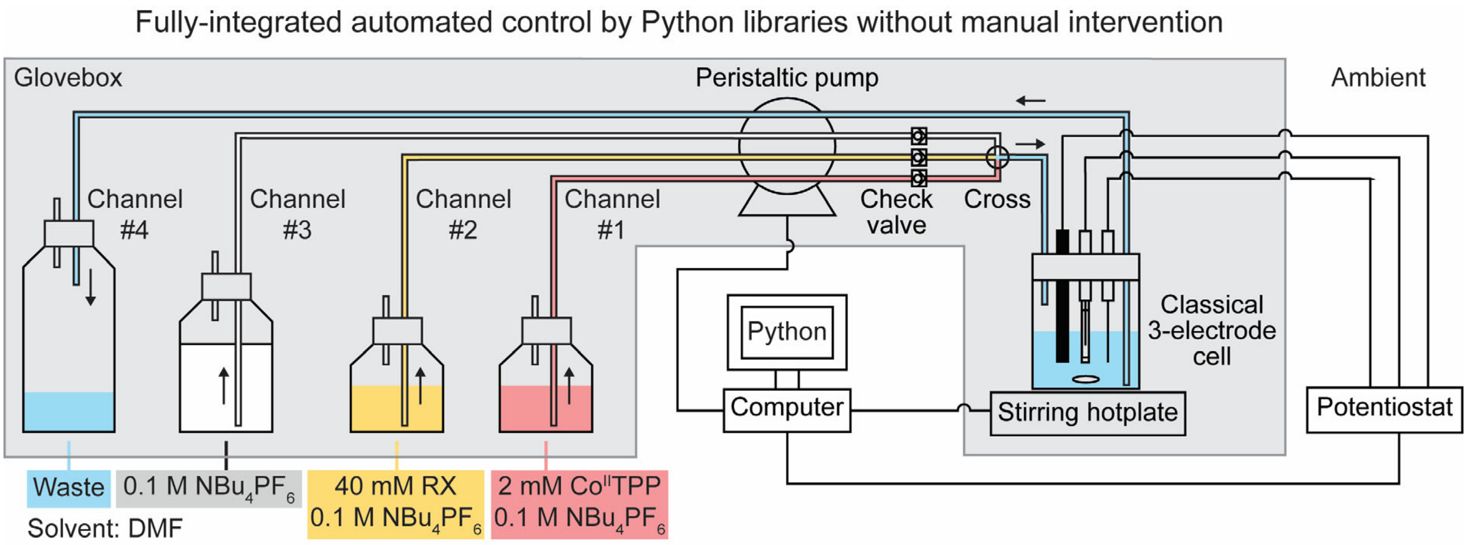

Supplementary Fig. 1. Schematic of the autonomous electrochemical platform. This schematic is the same as Fig. 1c in the main text.

To realize a tunable equivalent of a given organohalide (RX) substrate relative to cobalt(II) tetraphenylporphyrin (Co^{II}TPP) in the formulated electrolyte solution for the purpose of investigating the oxidative addition kinetics of RX to the electroreductively generated Co^{I}TPP, three stock solutions are prepared as follows: (1) 2 mM Co^{II}TPP in anhydrous dimethylformamide (DMF) solution containing 0.1 M tetrabutylammonium hexafluorophosphate (0.1 M NBu_4PF_6) as the supporting electrolyte; (2) 40 mM RX in anhydrous DMF solution containing 0.1 M NBu_4PF_6; (3) anhydrous DMF solution containing 0.1 M NBu_4PF_6. By employing a four-channel peristaltic pump whose rotation speed and direction can be independently controlled for each channel, the aforementioned three stock solutions occupy three of the four channels (see Channels #1, #2, and #3 in Supplementary Fig. 1 above) and can be delivered at different flow rates into a conventional single-chamber electrochemical cell with a three-electrode configuration, allowing for tuning the equivalent of RX relative to Co^{II}TPP under a constant concentration of the supporting electrolyte (0.1 M NBu_4PF_6). An empty waste bottle occupies the fourth channel that rotates in the opposite direction to the other three channels, so that the electrochemical cell can be emptied out after testing a certain electrolyte composition and be ready for re-dispensing a different electrolyte composition. Due to the oxygen- and moisture-sensitive nature of the electrochemical and chemical reactions studied here, the flow system and the electrochemical cell are placed in an Argon-filled glovebox (Vigor SG1200/750TS), and communicate with the computer via a USB feedthrough (4 double-sided USB cables through a KF-40 flange, provided by Vigor) and with the potentiostat via an electrochemical feedthrough (a bundle of 8 double-sided electrical wires with banana

clips through a KF-40 flange, provided by Vigor). The annotated photographs of the whole platform are shown below in Supplementary Fig. 2.

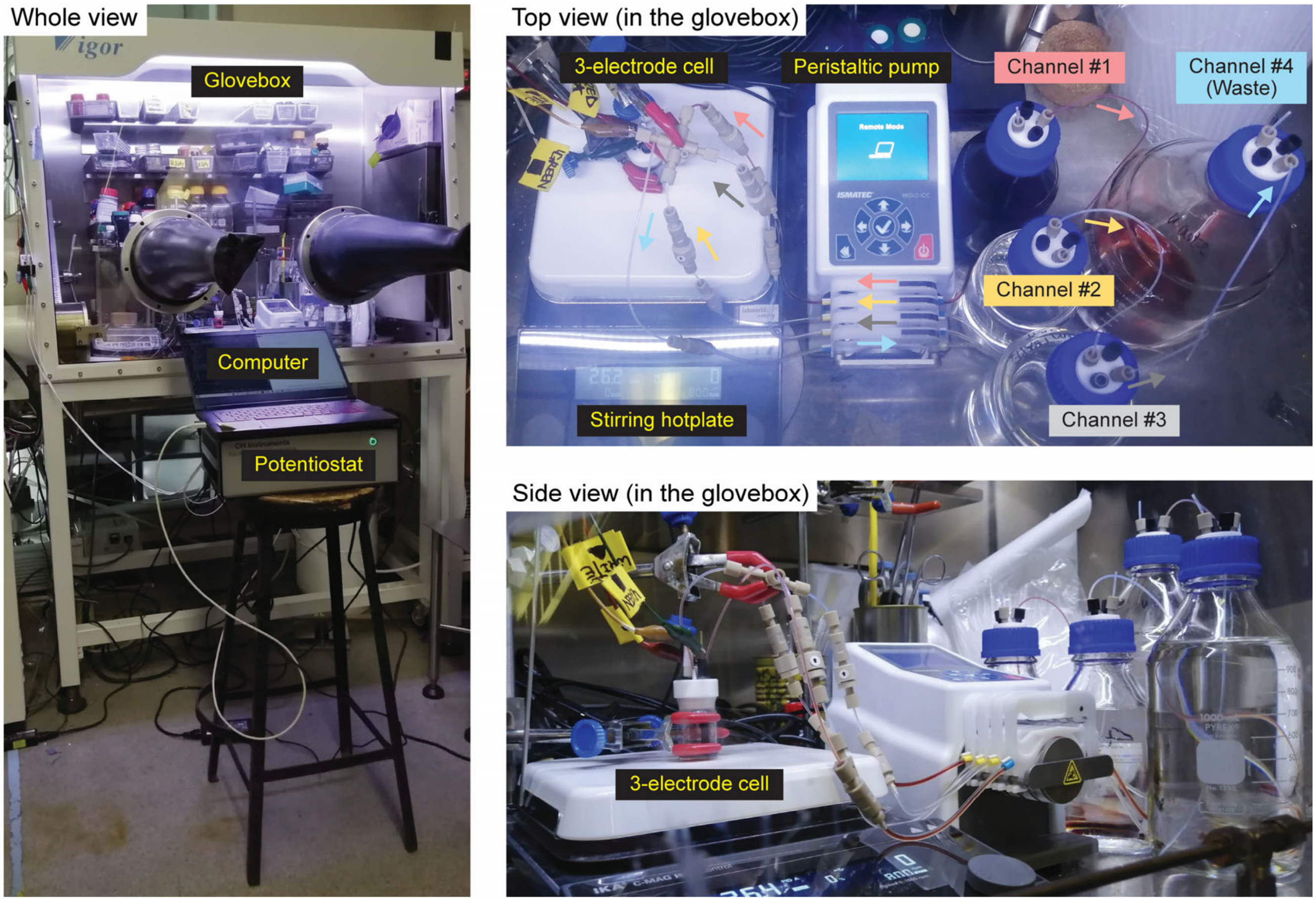

Supplementary Fig. 2. Annotated photographs of the autonomous electrochemical platform. The whole view (away from the glovebox) and the top or side view (in the glovebox) are provided.

Hardware specifications. Below in Supplementary Table 1, we provide detailed information on all the parts used to construct our autonomous electrochemical platform depicted in Supplementary Figs. 1 and 2 above. It is worth noting that all the parts are commercially available from common vendors, with a total cost of US$ 21,306.

Supplementary Table 1. Hardware specifications of the autonomous electrochemical platform.

Hardware	Manufacturer, Model, and Part Number	Quantity	Total Price
Computer	MSI GF63 Thin 10SC Laptop (Intel Core i5-10500H CPU @ 2.50GHz, 16GB RAM, 1TB SSD)	1 Piece	US$ 879
Potentiostat	CH Instruments CHI760E	1 Piece	US$ 13,540
Electrochemical Cell	3 mm Diameter Glassy Carbon Working Electrode (CHI104)	1 Piece	US$ 75
	Non-Aqueous Ag/Ag^+ Reference Electrode (CHI112)	1 Piece	US$ 25
	Platinum Wire Counter Electrode (CHI115)	1 Piece	US$ 75
	Glass Cell (CHI222)	1 Piece	US$ 5
	Teflon Cap (CHI223)	1 Piece	US$ 35
	VWR Spinbar Micro Stir Bars, 2 mm × 7 mm, PTFE Coating (58948-976)	1 Piece	US$ 3

Stirring Hotplate	IKA C-Mag HS 7 Control	1 Piece	US$ 972
Peristaltic Pump	Masterflex Ismatec Reglo Independent Channel Control (ICC) Digital Pump, 4-Channel, 8-Roller (MFLX78001-80)	1 Piece	US$ 4,406
Flow System	Masterflex Ismatec Pump Tubing, 3-Stop Microbore, Versilon 2001, 1.52 mm ID (MFLX96466-36)	4 Pieces	US$ 64
	Masterflex Transfer Tubing, PTFE, 1/16” OD, 25 Feets (MFLX06407-41)	1 Pack	US$ 61
	IDEX PEEK Adapter, 1/16” Barbed to 1/4”-28 Flat-Bottom Male (P-668)	8 Pieces	US$ 87
	IDEX Super Flangeless Ferrule with Stainless Steel Ring, 1/4”-28 Flat-Bottom, for 1/16” OD Tubing (P-250X)	26 Pieces	US$ 102
	IDEX Super Flangeless Nut, 1/4”-28 Flat-Bottom (P-255X)	26 Pieces	US$ 93
	IDEX PEEK Low-Pressure Union Body, 1/4”-28 Flat-Bottom (P-702-01)	11 Pieces	US$ 160
	IDEX PEEK Low-Pressure Cross Body, 1/4”-28 Flat-Bottom (P-722-01)	1 Piece	US$ 25
	IDEX Non-Metallic Inlet Check Valve (CV-3320)	3 Pieces	US$ 356
	IDEX Port Plug, Black Polypropylene, 1/4”-28 Flat-Bottom (D-324BLK)	8 Pieces	$US 15
	Cole-Parmer VapLock Solvent Delivery Cap, with Four Stainless Steel 1/4”-28 Threaded Ports, GL45 (EW-12018-02)	4 Pieces	US$ 225
	Corning Pyrex 500 mL Medium Bottle, with GL45 Screw Cap (1395-500)	2 Pieces	US$ 32
	Corning Pyrex 1 L Medium Bottle, with GL45 Screw Cap (1395-1L)	1 Piece	US$ 20
	Corning Pyrex 2 L Medium Bottle, with GL45 Screw Cap (1395-2L)	1 Piece	US$ 51
Sum of Total Price			US$ 21,306

Below in Supplementary Fig. 3, further instructions are provided on how to assemble the flow system using the Masterflex tubings, the IDEX fittings, and other accessories as listed in Supplementary Table 1 above.

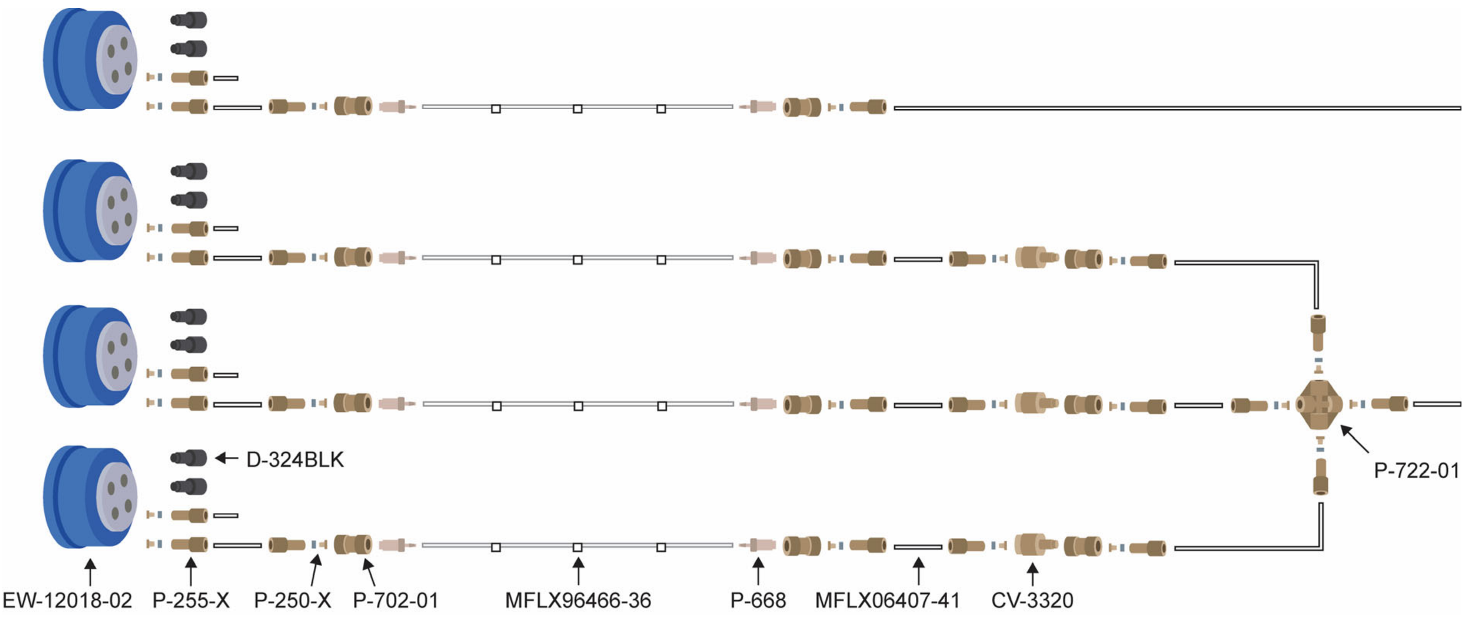

Supplementary Fig. 3. Instructions on how to assemble the flow system. The part numbers of the Masterflex tubings, the IDEX fittings, and other accessories (as listed in Supplementary Table 1 above) are annotated in this figure. The schematics of the parts are drawn to reflect their actual shapes.

Software specifications. Automated control of all the hardware listed in Supplementary Table 1 above is realized using a Python 3.9.12 programming language in a PyCharm 2022.1.2 integrated development environment (IDE). We employed several open-source Python libraries (as listed below in Supplementary Table 2) when developing our customized codes to realize the functionality of our autonomous electrochemical platform.

Supplementary Table 2. Software specifications of the autonomous electrochemical platform.

Functionality	Open-Source Python Library
Potentiostat	Hard Potato[1] (https://github.com/jrlLAB/hardpotato)
Stirring Hotplate	ika 1.16.0 (https://pypi.org/project/ika/)
Peristaltic Pump	ismatec 1.5.2 (https://pypi.org/project/ismatec/)
Bayesian Optimization	Dragonfly[2, 3] (https://dragonfly-opt.readthedocs.io/en/master/)

Supplementary Note 2. Standard operating procedures (SOPs) for our constructed autonomous electrochemical platform.

Section 1: Protocols for flow rate check. According to the operating manual of the Masterflex Ismatec Reglo ICC Digital Pump[4], the rotation speed range of each channel is 1.0–100 rpm with a step resolution of 0.01 rpm. We chose to use the Masterflex Ismatec Pump Tubing with a 1.52 mm ID, which corresponds to a flow rate range of 0.13–13 mL/min with a step resolution of 0.0013 mL/min for the 8-roller pump used in this work[4]. The chosen Masterflex Ismatec Pump Tubing is made of Versilon 2001 (formerly Tygon Chemical)[5], which is chemically compatible with the DMF solvent used in this work[6]. As described earlier in Supplementary Note 1, three of the four channels are used for electrolyte formulation from three stock solutions to realize a tunable equivalent of RX relative to Co^{II}TPP in DMF solvent containing 0.1 M NBu_4PF_6 supporting electrolyte. We checked the accuracy of the flow rates of these three channels based on the following procedure: (1) the inlets of these three channels were placed in solvent reservoirs containing deionized water (MilliporeSigma Milli-Q, 18.2 MΩ·cm), and their merged outlet was placed in a receiving flask (see Supplementary Fig. 4 below); (2) all these three channels were pre-filled with deionized water prior to flow rate check; (3) we first checked the flow rate of each channel individually by running it at a set rotation speed for 1 min and measuring the mass change of the receiving flask afterwards (see Supplementary Table 3 below); (4) we also checked the combined flow rate of the merged stream by running all three channels at the same time and at the same set rotation speed for 1 min (see Supplementary Table 3 below).

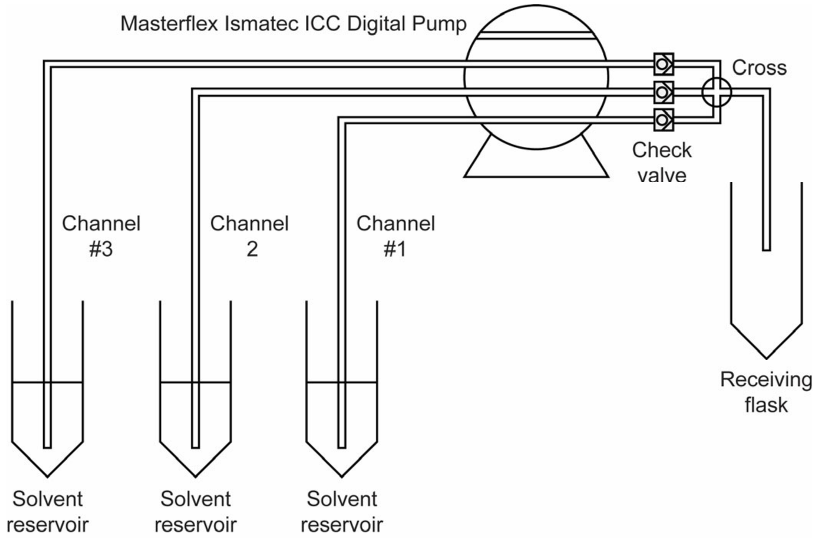

Supplementary Fig. 4. Schematic of the setup for flow rate check. This setup resembles the operating mode of our constructed autonomous electrochemical platform as depicted in Supplementary Fig. 1.

Supplementary Table 3. A demonstration of flow rate check using deionized water as the solvent.

Rotation speed (rpm)	Mass (g) of deionized water transferred into the receiving flask after 1 min					
	Channel #1	Channel #2	Channel #3	Expected	Channels #1, #2, and #3	Expected
1	0.1344	0.1422	0.1326	0.13	0.4088	0.39
3	0.3989	0.4244	0.4026	0.39	1.228	1.17

6	0.8009	0.8482	0.8085	0.78	2.458	2.34
10	1.331	1.411	1.348	1.3	4.092	3.9
30	3.952	4.211	4.017	3.9	12.18	11.7
60	7.788	8.374	8.013	7.8	24.01	23.4
100	12.65	13.88	13.37	13	39.54	39

The as-described flow rate check using deionized water suggests that (1) each individual channel can deliver an accurate flow rate spanning the entire rotation speed range, and (2) these three channels can be merged into a well-mixed stream with an accurate combined flow rate. Note that the differences in the flow rates of these three channels when running at the same set rotation speed are marginal and can be safely neglected without performing flow rate calibration.

Throughout the data acquisition process of this work, we routinely performed flow rate check every time prior to setting up a new experiment. Our routine flow rate check used anhydrous DMF (Sigma-Aldrich, 99.8%, density = 0.944 g/mL) as the solvent instead, and only ran each channel individually at 50 rpm for 30 seconds. Supplementary Fig. 5 below summarizes the statistical results of 31 routine flow rate checks performed throughout this work. Note that the absolute flow rate of each channel gradually decreased over time because the Masterflex Ismatec Pump Tubing was being squeezed over the course of long-term usage (see the left panel of Supplementary Fig. 5 below). Nevertheless, the relative flow rates of these three channels were fairly consistent throughout this work and were close to unity when running at the same rotation speed (see the right panel of Supplementary Fig. 5 below). Since the concentrations of $Co^{II}TPP$ and RX in the formulated electrolyte solution were governed by the relative flow rates of these three channels rather than the absolute flow rates, the accuracy of the electrolyte formulation process was warranted even without flow rate calibration.

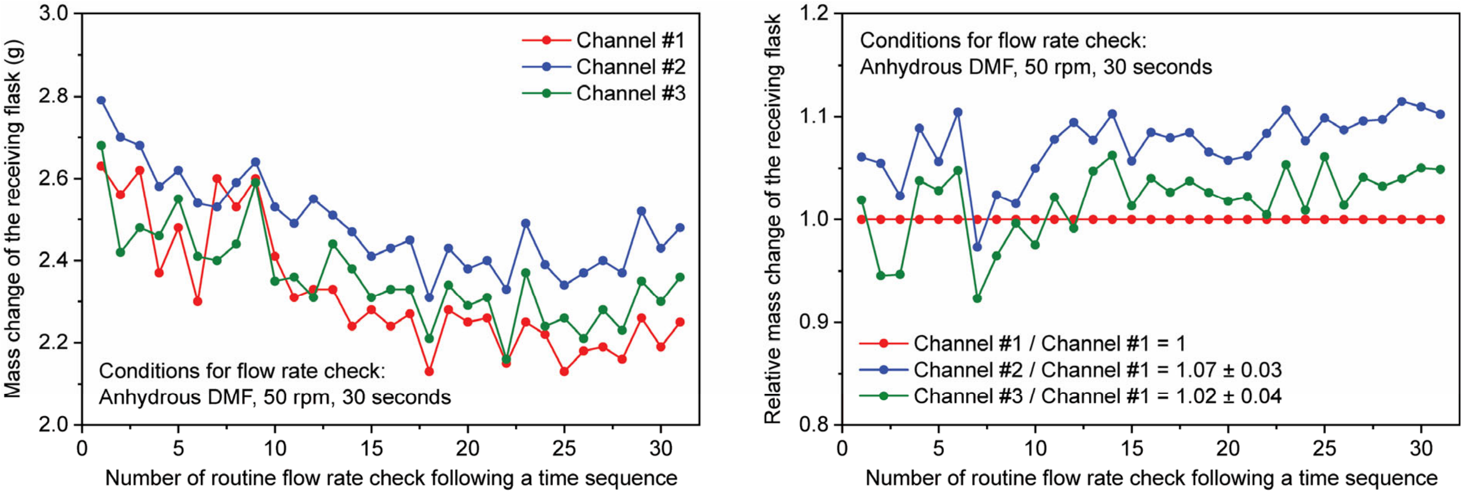

Supplementary Fig. 5. Routine flow rate checks using dimethylformamide (DMF) as the solvent. The left panel indicates the absolute flow rate of each channel, and the right panel indicates their relative flow rates.

Section 2: Protocols for automated electrolyte formulation and disposal. We established rigorous protocols that involve flushing the flow system and rinsing the electrochemical cell to ensure the accuracy of the formulated

electrolyte composition. General considerations are as follows: (1) once a new experiment is set up, the tubings for electrolyte formulation are empty, which requires pre-filling them with the corresponding stock solutions to get rid of void volumes prior to electrolyte formulation; (2) when switching to a new electrolyte composition that is different from the previous one, the tubing section between the cross body and the merged outlet (see the blue tubing section in Supplementary Fig. 1) contains a nontrivial volume of solution with the previous electrolyte composition, which requires flushing the flow system with the targeted new electrolyte composition to minimize cross-contamination; (3) similarly, the electrochemical cell needs to be rinsed with the targeted new electrolyte composition to minimize cross-contamination from the previous solution residues; (4) to keep [Co^{II}TPP] constant and vary [RX] during automated electrolyte formulation, we set the rotation speed of Channel #1 (which flows Co^{II}TPP stock solution) to be 50 rpm, and the combined rotation speeds of Channel #2 (which flows RX stock solution) and Channel #3 (which flows NBu_4PF_6 supporting electrolyte solution) to be 50 rpm. Detailed protocols for automated electrolyte formulation and disposal are visualized below in Supplementary Fig. 6.

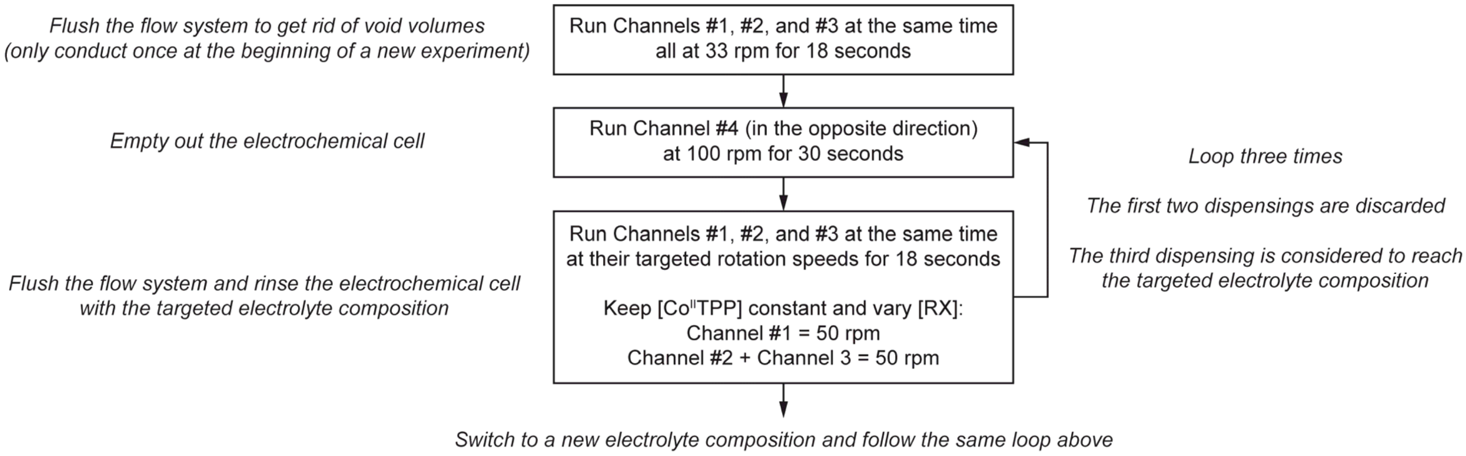

Supplementary Fig. 6. Detailed protocols for automated electrolyte formulation and disposal. The protocols in this flowchart are applicable to the autonomous electrochemical platform as depicted in Supplementary Fig. 1 in Supplementary Note 1.

Section 3: Protocols for automated electrochemical testing. Electrochemical measurements were conducted in a three-electrode cell comprising of a glassy carbon disk electrode, a nonaqueous Ag/Ag^+ reference electrode, and a platinum wire counter electrode connected to a CHI760 potentiostat (see the hardware list in Supplementary Table 1). 10 mM $AgNO_3$ in anhydrous acetonitrile (Sigma-Aldrich, 99.8%) containing 0.1 M NBu_4PF_6 was prepared as the filling solution for the reference electrode. A ferrocene/ferrocenium (Fc/Fc^+) redox couple was used for calibrating the potential of the reference electrode: E vs. Ag/Ag^+ = E vs. Fc/Fc^+ + 0.072 V (see the left panel of Supplementary Fig. 7 on the next page). The standard potential of the $Co^{II/I}$TPP redox couple was thus determined as: E^o($Co^{II/I}$TPP) = – 1.203 V vs. Ag/Ag^+ = – 1.275 V vs. Fc/Fc^+ (see the right panel of Supplementary Fig. 7 on the next page). We found that the reference electrode potential remained fairly stable throughout this work (less than ~10 mV drift over long-term storage), so that we did not include reference electrode calibration as part of the protocols for automated electrochemical testing.

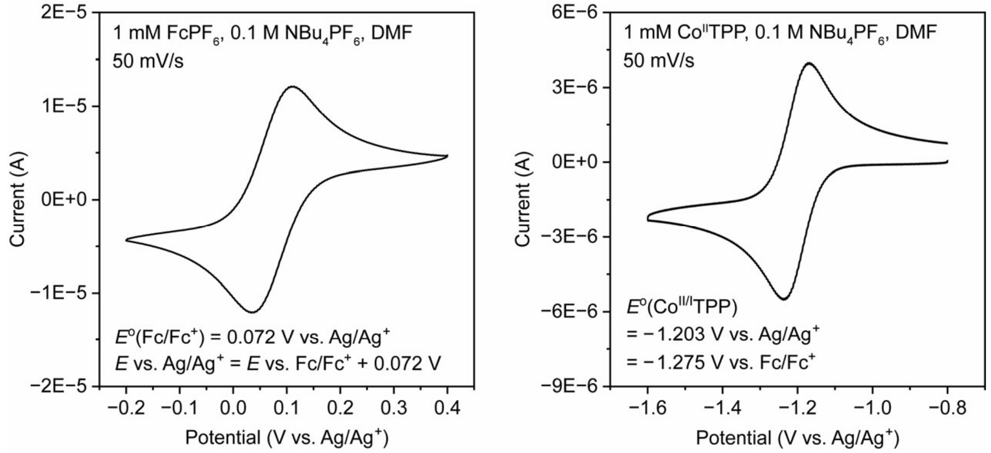

Supplementary Fig. 7. Calibration of the nonaqueous Ag/Ag$^+$ reference electrode potential. The left panel shows the cyclic voltammogram of the Fc/Fc$^+$ redox couple, and the right panel shows the cyclic voltammogram of the Co$^{II/I}$TPP redox couple.

After an electrolyte solution with a targeted electrolyte composition was formulated (as described in the Section 2 above), automated electrochemical testing was conducted via a modified Hard Potato[1] Python library. Detailed protocols for automated electrochemical testing, including detailed electroanalytical parameters, are shown below in Supplementary Fig. 8.

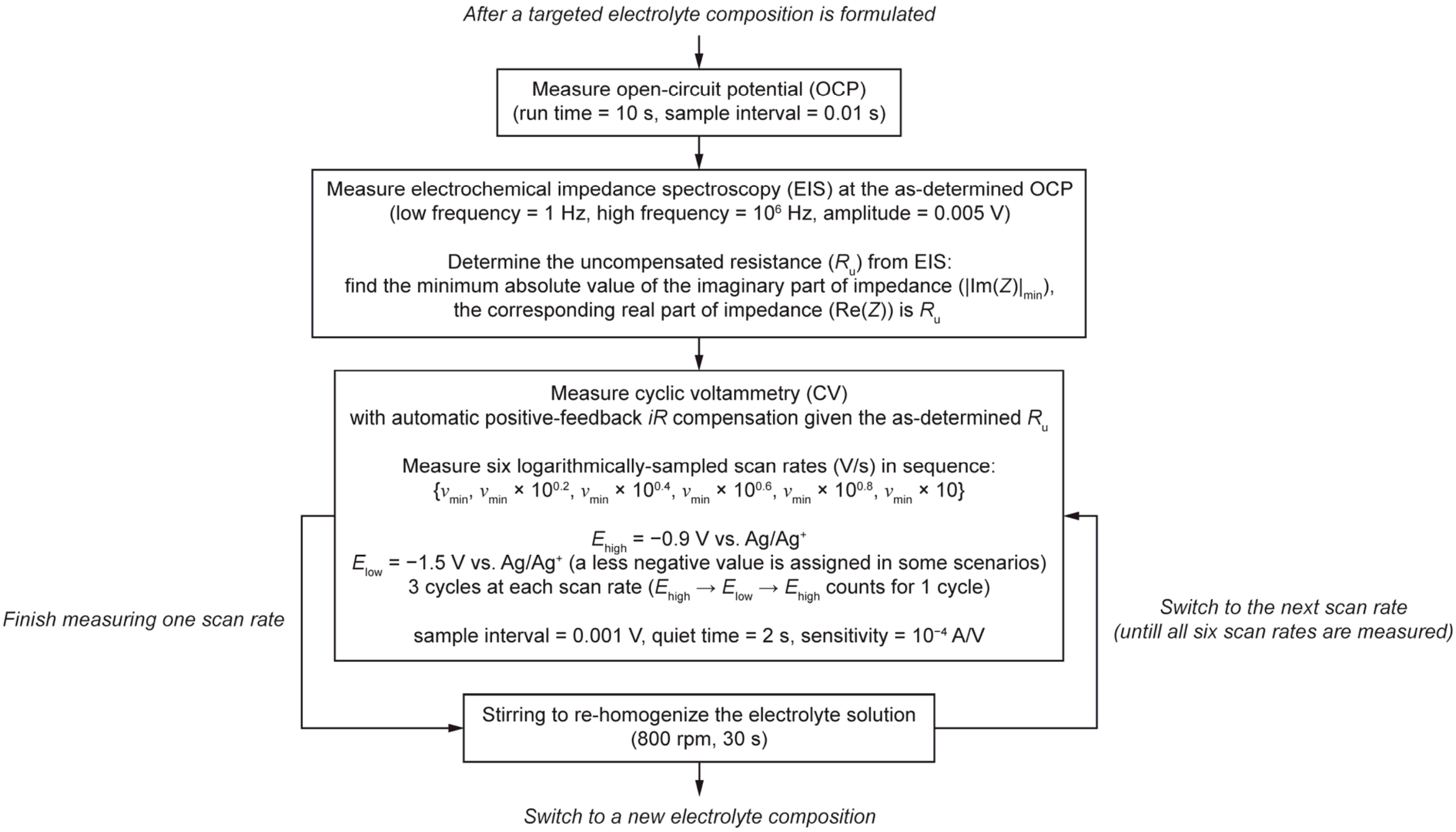

Supplementary Fig. 8. Detailed protocols for automated electrochemical testing. Detailed electroanalytical parameters for measuring open-circuit potential (OCP), electrochemical impedance spectroscopy (EIS), and cyclic voltammetry (CV) with automatic positive-feedback *iR* compensation are shown in this flowchart.

Automated electrochemical testing proceeds via the following four iterative steps: (1) open-circuit potential (OCP) measurement; (2) electrochemical impedance spectroscopy (EIS) measurement at the as-determined OCP, from which the uncompensated resistance (R_u) between the working and reference electrodes was determined from the real part of impedance (Re(Z)) at the minimum absolute value of the imaginary part of impedance ($|\text{Im}(Z)|_{\text{min}}$) (note that we introduced this functionality of R_u determination by modifying the original version of the Hard Potato[1] Python library); (3) given the as-determined R_u, automatic positive-feedback iR compensation was enabled during a set of cyclic voltammetry (CV) measurements at six logarithmically-sampled scan rates with the maximal and minimal scan rates differing by a factor of 10 ($v_{\text{min}}/v_{\text{max}} = 1/10$): $\{v_{\text{min}}, v_{\text{min}} \times 10^{0.2}, v_{\text{min}} \times 10^{0.4}, v_{\text{min}} \times 10^{0.6}, v_{\text{min}} \times 10^{0.8}, v_{\text{min}} \times 10^{1} = v_{\text{max}}\}$; (4) stirring to re-homogenize the electrolyte solution every time after measuring one scan rate and before switching to the next scan rate.

Section 4: Protocols for deep-learning-based mechanistic analysis of cyclic voltammetry. In our previous work[7], we developed a deep-learning model based on the residual neural network (ResNet) architecture that automatically analyzes a set of either simulated or experimental voltammograms at different scan rates (v) and classifies five prototypical mechanisms in molecular electrochemistry (*E*, *EC*, *CE*, *ECE*, *DISP1*, as defined in Supplementary Table 6). Prior to deep-learning analysis, each voltammogram set is automatically reformatted as follows (more details were described in our previous work[7]): (1) all the voltammograms are reformatted so that the forward scan points in the positive direction with a positive current; (2) all the potentials (E) are adjusted and rescaled so that the adjusted zero potential roughly corresponds to either the standard potential for a reversible redox event or the largest slope of the first rising peak for an irreversible redox event; (3) all the currents (i) are normalized against the largest current in the forward scan among all the voltammograms. Each reformatted voltammogram set, along the relative potential and relative current axes (see the voltammogram sets plotted in Supplementary Note 4 for example), is then translated into a two-dimensional matrix of $\{v, i(E)\}_n$ (where v is scan rate, $i(E)$ is reformatted voltammogram under each scan rate, and n is the number of different scan rates) as the input for deep-learning analysis.

Deep-learning analysis of each reformatted voltammogram set is conducted using eight individually pre-trained deep-learning models to decrease the effect of randomness in individual model training (details about the model training procedure were described in our previous work[7]). Each of the eight individually pre-trained deep-learning models yields numerical distributions of five propensity values for the as-mentioned five mechanisms: {*E*%, *EC*%, *CE*%, *ECE*%, *DISP1*%}, where the five propensity values add up to unity. The resultant numerical distributions of mechanism propensities are subsequently averaged, yielding the output of deep-learning analysis.

In this work, each voltammogram set comprises the first cycles of a set of six voltammograms measured at six logarithmically-sampled scan rates (see protocols for CV measurements in the Section 3 above), which are first smoothed by a Savitzky-Golay filter to reduce environmental noises in voltammograms (49-point filter window, third-order polynomial) and then reformatted along the relative potential and relative current axes as the input for

deep-learning analysis (as described above in this Section 4). Deep-learning analysis is performed immediately after each voltammogram set is measured and reformatted.

Section 5: Protocols for Bayesian optimization of the deep-learning-generated mechanism propensity. The Bayesian optimization functionality is enabled by a Dragonfly[2, 3] Bayesian optimization package, on top of which our customized algorithm was further developed to achieve a designated goal of maximizing the voltammograms' propensity, obtained from the deep-learning model, toward an *EC* mechanism. Without *a priori* knowledge of the parameter space, a typical campaign of Bayesian optimization includes a finite number of random sampling steps and optimization steps. Each step inquires a combination of [RX] and v_{min} (defined in the Section 3 above) in the parameter space, yielding a deep-learning-generated propensity value of an *EC* mechanism based on the measured voltammogram set (see the Sections 3 and 4 above).

We first performed proof-of-concept Bayesian optimization trials for a model system when RX = 1-bromobutane (*n*-BuBr) (detailed in Supplementary Note 4). For these trials, the parameter space for Bayesian optimization is [RX] ∈ [0, 20] mM and v_{min} ∈ [0.01, 0.2] V/s, the number of random sampling steps is set to 6, and the number of optimization steps is varied (6 or 9 or 14). We found that 9 optimization steps can effectively converge to the desired maximized propensity of an *EC* mechanism with good statistical reproducibility. Therefore, the number of optimization steps is set to 9 for the rest of this work, leading to a 15-step campaign of Bayesian optimization.

A 15-step of Bayesian optimization was then included in a closed-loop workflow for autonomous investigations of an *EC* mechanism between CoTPP and a diverse scope of RX substrates (see the workflow in Supplementary Fig. 16). As RX's reactivity may vary greatly, the accessible range of [RX] was expanded by introducing additional RX reservoirs in the flow chemistry module (Supplementary Note 6).

In a typical case when a given RX can be obtained in plentiful quantities, three RX reservoirs are used to access RX ∈ [0.008, 1000] mM, where the 0.8 mM, 40 mM, and 2000 mM RX reservoir access RX ∈ [0.008, 0.4] mM, [0.4, 20] mM, and [20, 1000] mM, respectively. The parameter space for Bayesian optimization is $\log_{10}$[RX] ∈ [$\log_{10}$(0.008 mM), $\log_{10}$(1000 mM)] and v_{min} ∈ [0.01, 0.2] V/s. To ensure a wide coverage of the parameter space by the initial 6 random sampling steps, [RX] is sampled from [0.008, 0.4] mM for the first two steps, [0.4, 20] mM for the next two steps, and [20, 1000] mM for the last two steps in an incremental manner, while v_{min} is always sampled from [0.01, 0.2] V/s for all six steps. For the 9 optimization steps, [RX] is always sampled from [0.008, 1000] mM, and v_{min} is always sampled from [0.01, 0.2] V/s.

In a special case when a given RX cannot be obtained in plentiful quantities, only two of the three RX reservoirs are used to access RX ∈ [0.008, 20] mM, where the 0.8 mM and 40 mM RX reservoir access RX ∈ [0.008, 0.4] mM and [0.4, 20] mM, respectively. The parameter space for Bayesian optimization is $\log_{10}$[RX] ∈ [$\log_{10}$(0.008 mM), $\log_{10}$(20 mM)] and v_{min} ∈ [0.01, 0.2] V/s. To ensure a wide coverage of the parameter space by the initial 6 random sampling steps, [RX] is sampled from [0.008, 0.4] mM for the first three steps, and from [0.4, 20] mM for

the last three steps, while v_{min} is always sampled from [0.01, 0.2] V/s for all six steps. For the 9 optimization steps, [RX] is always sampled from [0.008, 20] mM, and v_{min} is always sampled from [0.01, 0.2] V/s.

In either case described above, at the end of a 15-step campaign of Bayesian optimization, if the deep-learning-generated propensity of an *EC* mechanism can exceed a threshold of 50%, the system is considered to bear an *EC* mechanism, otherwise the system is considered to bear alternative mechanisms.

Supplementary Note 3. The parameter space for automated exhaustive CV experiments of cobalt tetraphenylporphyrin (CoTPP) with 1-bromobutane (*n*-BuBr).

[RX] ∈ [0, 20] mM with an increment of 1 mM in [RX]. As described in the Section 2 of Supplementary Note 2, [Co^{II}TPP] is kept constant and [RX] is varied during automated electrolyte formulation. To do so, the rotation speed of Channel #1 (which flows Co^{II}TPP stock solution) is set to be 50 rpm, and the combined rotation speeds of Channel #2 (which flows RX stock solution) and Channel #3 (which flows NBu_4PF_6 supporting electrolyte solution) are set to be 50 rpm. The compositions of the stock solutions used for automated exhaustive CV experiments are tabulated below in Supplementary Table 4 (also see Fig. 1c in the main text).

Supplementary Table 4. The stock solutions used for automated exhaustive CV experiments.

Channel	Stock solution	Rotation speed
#1	2 mM Co^{II}TPP in 0.1 M NBu_4PF_6 in DMF	50 rpm
#2	40 mM RX in 0.1 M NBu_4PF_6 in DMF [1]	50 rpm combined
#3	0.1 M NBu_4PF_6 in DMF	

[1] RX = *n*-BuBr in the case of automated exhaustive CV experiments.

As a result, the formulated electrolyte solution has a constant [Co^{II}TPP] = 1 mM and a tunable [RX] ∈ [0, 20] mM. Moreover, since each running channel has a minimum rotation speed of 1.0 rpm and a step resolution of 0.01 rpm (see page S11), the minimum non-zero [RX] value that can be accessed is 0.4 mM, and the step resolution of [RX] is 0.004 mM. Therefore, it is feasible to achieve a small increment of 1 mM in [RX] ∈ [0, 20] mM by increasing the rotation speed of Channel #2 by 2.5 rpm and decreasing that of Channel #3 by 2.5 rpm accordingly, leading to 21 different [RX] values for automated exhaustive CV experiments.

v_{min} ∈ [0.01, 0.2] V/s with an increment of 0.01 V/s in v_{min}. As described in the Sections 3 and 4 of Supplementary Note 2, deep-learning analysis is performed on a set of voltammograms measured at six logarithmically-sampled scan rates with the maximal and minimal scan rates differing by a factor of 10 ($v_{min}/v_{max} = 1/10$): $\{v_{min}, v_{min} \times 10^{0.2}, v_{min} \times 10^{0.4}, v_{min} \times 10^{0.6}, v_{min} \times 10^{0.8}, v_{min} \times 10^{1} = v_{max}\}$. By setting v_{min} ∈ [0.01, 0.2] V/s with an increment of 0.01 V/s in v_{min}, 20 voltammogram sets (each of which is represented by a v_{min}) can be measured under a given [RX], as tabulated below in Supplementary Table 5.

Supplementary Table 5. Parameterization of scan rates for automated exhaustive CV experiments.

v_{min} (V/s)	Six logarithmically-sampled scan rates $\{v_{min}, v_{min} \times 10^{0.2}, v_{min} \times 10^{0.4}, v_{min} \times 10^{0.6}, v_{min} \times 10^{0.8}, v_{min} \times 10 = v_{max}\}$ (V/s)
0.01	{0.01, 0.02, 0.03, 0.04, 0.06, 0.1}
0.02	{0.02, 0.03, 0.05, 0.08, 0.13, 0.2}
0.03	{0.03, 0.05, 0.08, 0.12, 0.19, 0.3}
0.04	{0.04, 0.06, 0.1, 0.16, 0.25, 0.4}
0.05	{0.05, 0.08, 0.13, 0.2, 0.32, 0.5}

0.06	{0.06, 0.1, 0.15, 0.24, 0.38, 0.6}
0.07	{0.07, 0.11, 0.18, 0.28, 0.44, 0.7}
0.08	{0.08, 0.13, 0.2, 0.32, 0.5, 0.8}
0.09	{0.09, 0.14, 0.23, 0.36, 0.57, 0.9}
0.10	{0.1, 0.16, 0.25, 0.4, 0.63, 1}
0.11	{0.11, 0.17, 0.28, 0.44, 0.69, 1.1}
0.12	{0.12, 0.19, 0.3, 0.48, 0.76, 1.2}
0.13	{0.13, 0.21, 0.33, 0.52, 0.82, 1.3}
0.14	{0.14, 0.22, 0.35, 0.56, 0.88, 1.4}
0.15	{0.15, 0.24, 0.38, 0.6, 0.95, 1.5}
0.16	{0.16, 0.25, 0.4, 0.64, 1.01, 1.6}
0.17	{0.17, 0.27, 0.43, 0.68, 1.07, 1.7}
0.18	{0.18, 0.29, 0.45, 0.72, 1.14, 1.8}
0.19	{0.19, 0.3, 0.48, 0.76, 1.2, 1.9}
0.20	{0.2, 0.32, 0.5, 0.8, 1.26, 2}

Such settings lead to CV measurements in the scan rate range of [0.01, 2] V/s, which is feasible for the CHI760 potentiostat used in this work. The reason we consider not measuring CV at scan rates higher than 2 V/s is because of the environmental noises in voltammograms that appear to magnify at higher scan rates. Such environmental noises likely originate from the use of an electrochemical feedthrough, connecting the electrochemical cell in the glovebox and the potentiostat outside the glovebox (see pages S7–S8), that may not be well-shielded. We verify that such environmental noises cannot be eliminated by placing the electrochemical cell in a Faraday cage (CH Instruments, CHI202) in the glovebox and grounding the Faraday cage to the potentiostat via the electrochemical feedthrough. Therefore, we set the upper limit of scan rate as 2 V/s for CV measurements without the use of a Faraday cage, and apply a Savitzky-Golay filter (49-point filter window, third-order polynomial) to smooth the measured voltammograms (see page S15).

420 combinations of [RX] and v_{min}, representing 2,520 voltammograms, for automated exhaustive CV experiments. [RX] ∈ [0, 20] mM and v_{min} ∈ [0.01, 0.2] V/s with an increment of 1 mM in [RX] and 0.01 V/s in v_{min} result in 420 combinations of [RX] and v_{min} in the parameter space for automated exhaustive CV experiments. As each combination of [RX] and v_{min} represents a set of six voltammograms, a total of 2,520 voltammograms are measured for automated exhaustive CV experiments.

Supplementary Table 6. Five prototypical mechanisms in molecular electrochemistry included in the deep-learning (DL) model.

Notation	Description	Equation(s) [1]
E	An interfacial single-electron transfer with any level of reversibility	$R \rightleftharpoons O + e^-$
EC	An *E* step with any level of reversibility followed by a solution reaction (*C* step) with any level of reversibility	$R \rightleftharpoons O + e^-$<br>$O \rightleftharpoons A$
CE	An *E* step with any level of reversibility preceded by a solution reaction (*C* step) with any level of reversibility	$A \rightleftharpoons R$<br>$R \rightleftharpoons O + e^-$
ECE	Two *E* steps connected by an irreversible rate-limiting *C* step, with the second *E* step being more thermodynamically favored than the first one	$R_1 \rightleftharpoons O_1 + e^-$<br>$O_1 \rightarrow R_2$<br>$R_2 \rightleftharpoons O_2 + e^-$
DISP1	An *E* step followed by an irreversible, rate-determining *C* step, with the product from the rate-determining *C* step undergoing an irreversible disproportionation reaction with the product from the preceding *E* step	$R_1 \rightleftharpoons O_1 + e^-$<br>$O_1 \rightarrow R_2$<br>$R_2 + O_1 \rightarrow O_2 + R_1$

[1] The listed equations in this Supplementary Table 6 consider an electro-oxidation reaction as the initiation step for each mechanism, meaning that the forward scan of the voltammogram is the anodic scan in the positive direction with a positive current following the IUPAC (International Union of Pure and Applied Chemistry) convention. To train the deep-learning model in a consistent manner, for any electrochemical process that is initiated by an electro-reduction reaction (e.g., an *EC* process between CoTPP and RX that is initiated by the reduction of Co^{II}TPP), the voltammogram is reformatted (Supplementary Note 2) so that the forward scan (the cathodic scan in this case) also points in the positive direction along the relative potential axis with a positive current along the relative current axis (see the voltammograms plotted in Supplementary Note 4 for example). Additional considerations regarding the training of the deep-learning model are detailed in our previous work[7].

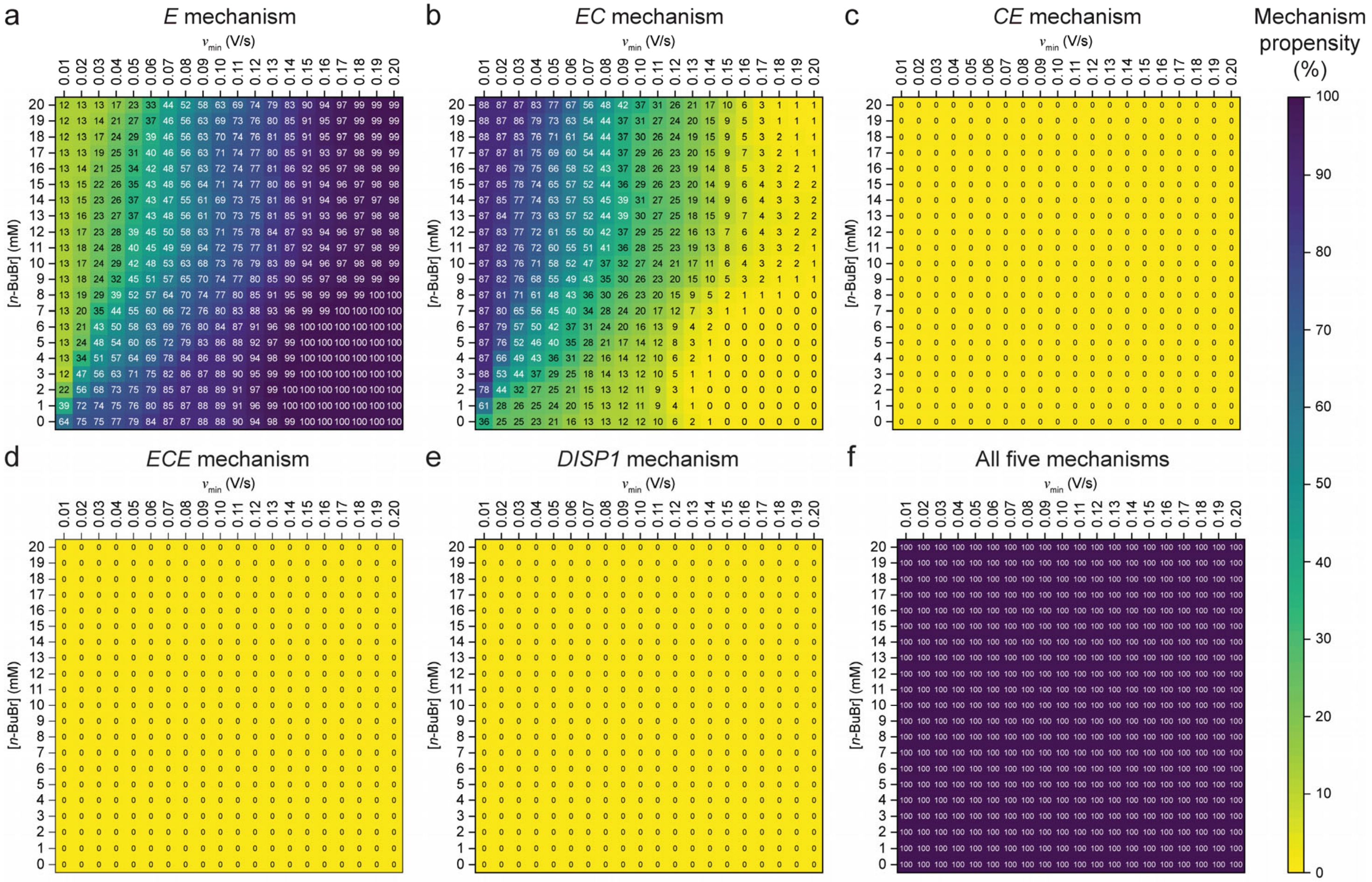

Supplementary Fig. 9. Summary of all the DL-generated mechanism propensity values obtained from automated exhaustive CV experiments of CoTPP with *n*-BuBr. The propensity values of (a) an *E* mechanism, (b) an *EC* mechanism (the same as Fig. 2b in the main text), (c) a *CE* mechanism, (d) an *ECE* mechanism, (e) a *DISP1* mechanism, and (f) the sum of the propensity values of all five mechanisms. Note that, over the entire parameter space ([*n*-BuBr] ∈ [0, 20] mM and v_{min} ∈ [0.01, 0.2] V/s), the propensity value of a *DISP1* mechanism is always < 0.02% so that it is displayed as 0% in panel (e), while the propensity values of a *CE* mechanism and an *ECE* mechanism are indeed always 0%.

Supplementary Note 4. Bayesian optimization of the DL-generated propensity of an *EC* mechanism for a model system of CoTPP with *n*-BuBr.

For a model system of CoTPP with *n*-BuBr, automated exhaustive CV experiments in the parameter space of [*n*-BuBr] ∈ [0, 20] mM and $v_{\min}$ ∈ [0.01, 0.2] V/s show that the DL-generated propensity of an *EC* mechanism, extracted from voltammogram features, provides a quantifiable value with sufficient detection sensitivity for discerning the possible presence of an *EC* mechanism (Fig. 2b in the main text). These results suggest the DL-generated propensity of an *EC* mechanism can serve as a numerical figure-of-merit for Bayesian optimization with a designated goal of maximizing the voltammograms' propensity toward an *EC* mechanism.

We set to demonstrate that, for such a model system of CoTPP with *n*-BuBr, the DL-generated propensity of an *EC* mechanism can be maximized in the parameter space of [*n*-BuBr] ∈ [0, 20] mM and $v_{\min}$ ∈ [0.01, 0.2] V/s by the use of a Dragonfly[2, 3] Bayesian optimization package (see the Section 5 of Supplementary Note 2). Shown below in Supplementary Fig. 10a is a 20-step campaign of Bayesian optimization, including 6 random sampling and 14 optimization steps, without any *a priori* knowledge of the parameter space. Each step inquires a combination of [*n*-BuBr] and $v_{\min}$ in the parameter space, yielding a DL-generated propensity value of an *EC* mechanism based on the measured voltammogram set. Supplementary Fig. 10b plots the obtained propensity value of an *EC* mechanism and the inquired [*n*-BuBr] and $v_{\min}$ values as a function of the step number. The desired maximized propensity of an *EC* mechanism is quickly reached within a finite number of optimization steps, meanwhile the maximal [*n*-BuBr] and the minimal $v_{\min}$ in the parameter space are approached, consistent with the ground-truth knowledge of the parameter space obtained from automated exhaustive CV experiments for this model system (see Fig. 2b in the main text).

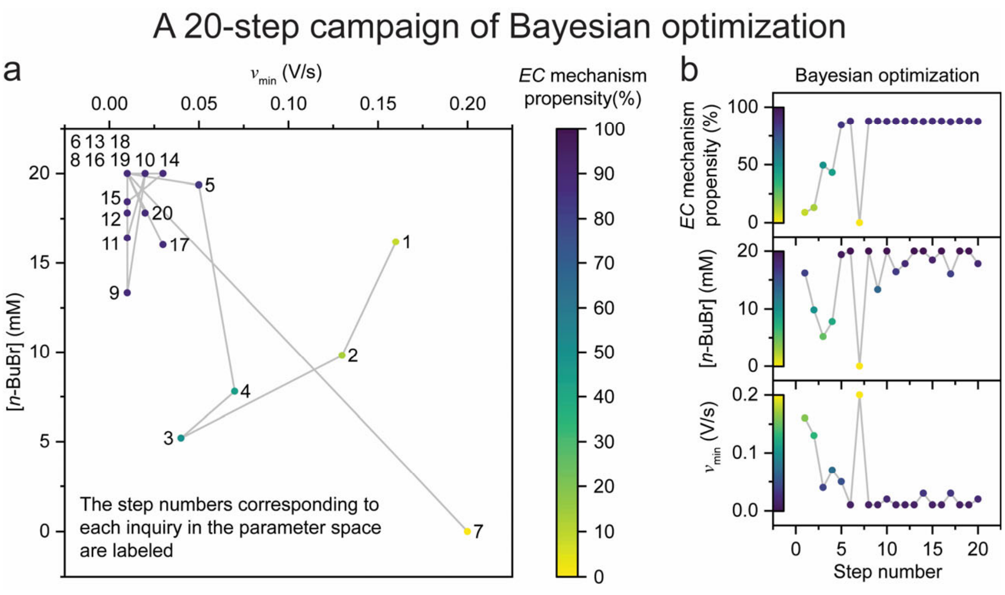

Supplementary Fig. 10. A 20-step campaign of Bayesian optimization. (a) The trajectory in the parameter space of [*n*-BuBr] ∈ [0, 20] mM and $v_{\min}$ ∈ [0.01, 0.2] V/s. (b) The DL-generated propensity of an *EC* mechanism, the inquired [*n*-BuBr], and the inquired $v_{\min}$ with respect to the step number.

Supplementary Fig. 11a plots the measured voltammogram sets under each of the inquired combinations of [*n*-BuBr] and v_{min} during the as-described 20-step campaign of Bayesian optimization (with the subscripts in the panel labels indicating the step numbers), and Supplementary Fig. 11b summarizes the DL-generated propensity values of an *EC* mechanism, together with those for the other four mechanisms, for each of all the 20 steps.

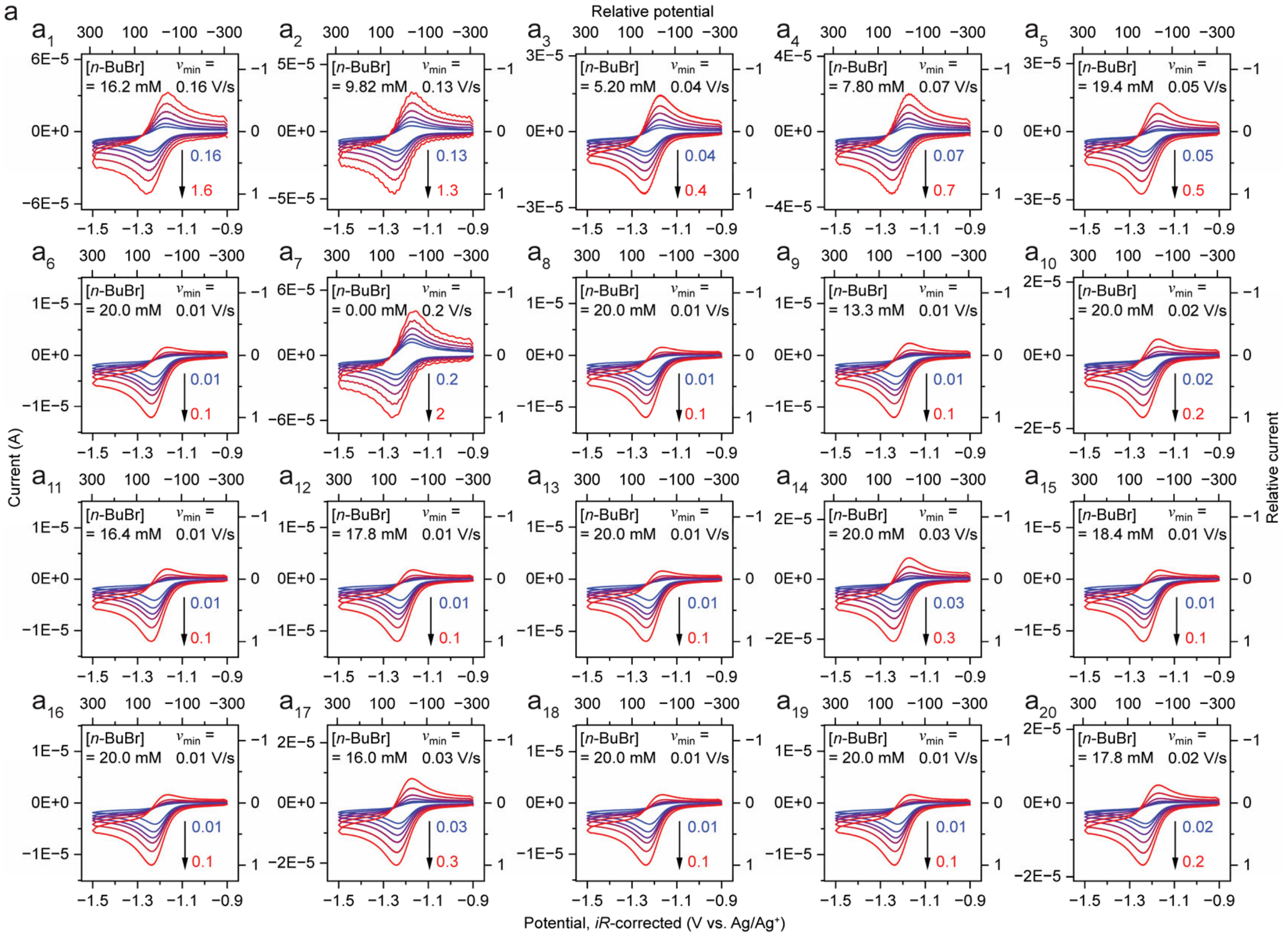

b

Potential range: between −0.9 and −1.5 V vs. Ag/Ag+							
Step	[*n*-BuBr] (mM)	v_{min} (V/s)	Mechanism propensity (%)				
			E	*EC*	*CE*	*DISP1*	*ECE*
1	16.2	0.16	91.1	8.9	0.0	0.0	0.0
2	9.82	0.13	86.9	13.1	0.0	0.0	0.0
3	5.20	0.04	50.5	49.5	0.0	0.0	0.0
4	7.80	0.07	56.8	43.2	0.0	0.0	0.0
5	19.4	0.05	15.5	84.5	0.0	0.0	0.0
6	20.0	0.01	12.3	87.7	0.0	0.0	0.0
7	0.00	0.2	100.0	0.0	0.0	0.0	0.0
8	20.0	0.01	12.3	87.7	0.0	0.0	0.0
9	13.3	0.01	12.2	87.8	0.0	0.0	0.0
10	20.0	0.02	12.5	87.5	0.0	0.0	0.0
11	16.4	0.01	12.2	87.8	0.0	0.0	0.0
12	17.8	0.01	12.3	87.7	0.0	0.0	0.0
13	20.0	0.01	12.3	87.7	0.0	0.0	0.0
14	20.0	0.03	12.6	87.4	0.0	0.0	0.0
15	18.4	0.01	12.2	87.8	0.0	0.0	0.0
16	20.0	0.01	12.2	87.8	0.0	0.0	0.0
17	16.0	0.03	12.8	87.2	0.0	0.0	0.0
18	20.0	0.01	12.2	87.8	0.0	0.0	0.0
19	20.0	0.01	12.2	87.8	0.0	0.0	0.0
20	17.8	0.02	12.6	87.4	0.0	0.0	0.0

Supplementary Fig. 11. Detailed results from a 20-step campaign of Bayesian optimization. (a) The measured voltammogram sets. (b) The inquired parameter combinations and the yielded mechanism propensity values.

From the as-described 20-step campaign of Bayesian optimization in the parameter space of [n-BuBr] ∈ [0, 20] mM and v_{min} ∈ [0.01, 0.2] V/s, it seems that a total of 20 steps may be an overkill for converging to the desired maximized propensity of an *EC* mechanism. Therefore, we further investigate the step-number-dependence of Bayesian optimization for such a model system of CoTPP and n-BuBr. Supplementary Figs. 12 and 13 show three replicates of a 15-step campaign of Bayesian optimization (6 random sampling and 9 optimization steps) and a 10-step campaign of Bayesian optimization (6 random sampling and 4 optimization steps), respectively.

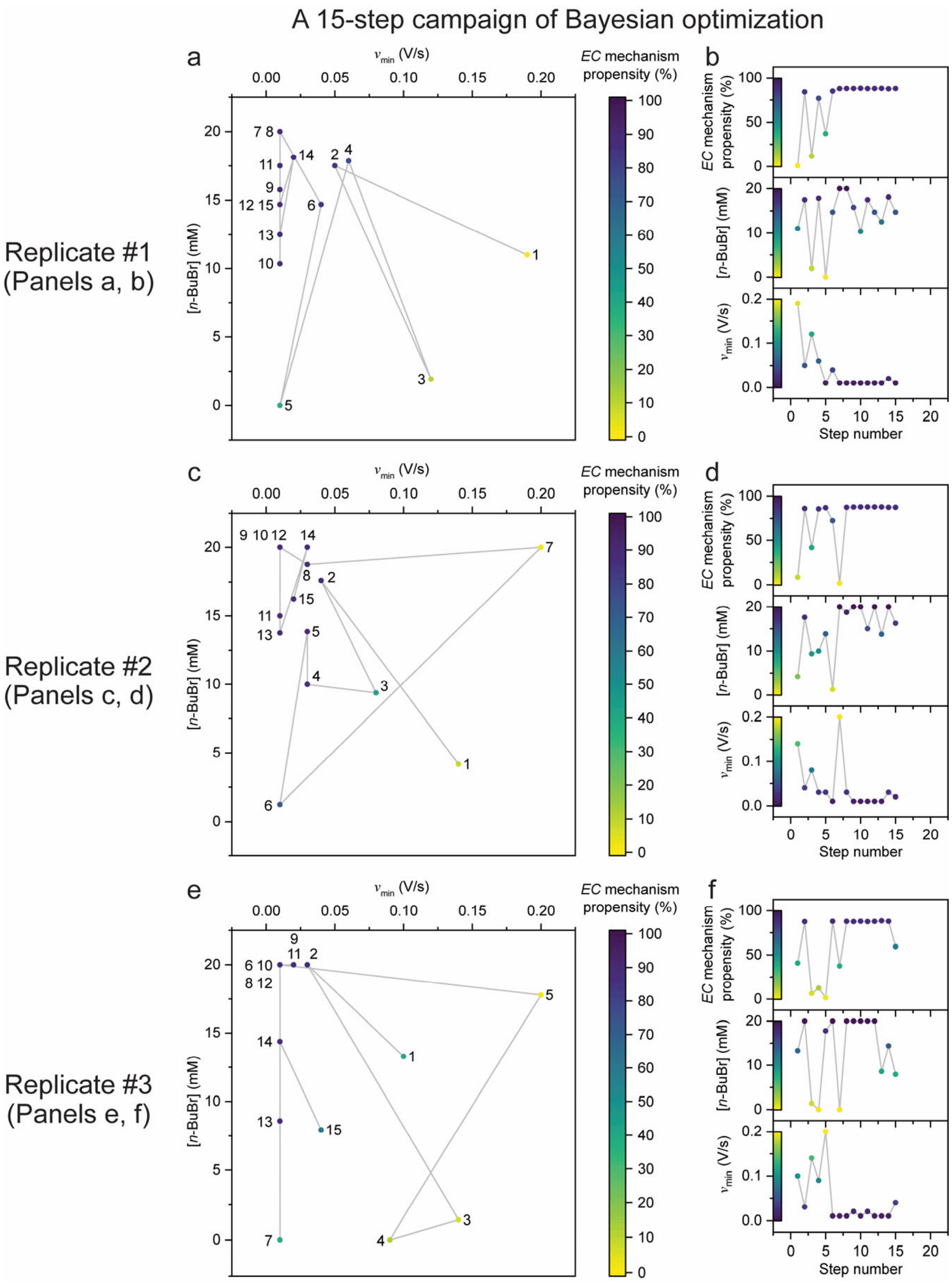

Supplementary Fig. 12. Three replicates of a 15-step campaign of Bayesian optimization. (a–c) The trajectory in the parameter space of [n-BuBr] ∈ [0, 20] mM and v_{min} ∈ [0.01, 0.2] V/s. (b–d) The DL-generated propensity of an *EC* mechanism, the inquired [n-BuBr], and the inquired v_{min} with respect to the step number.

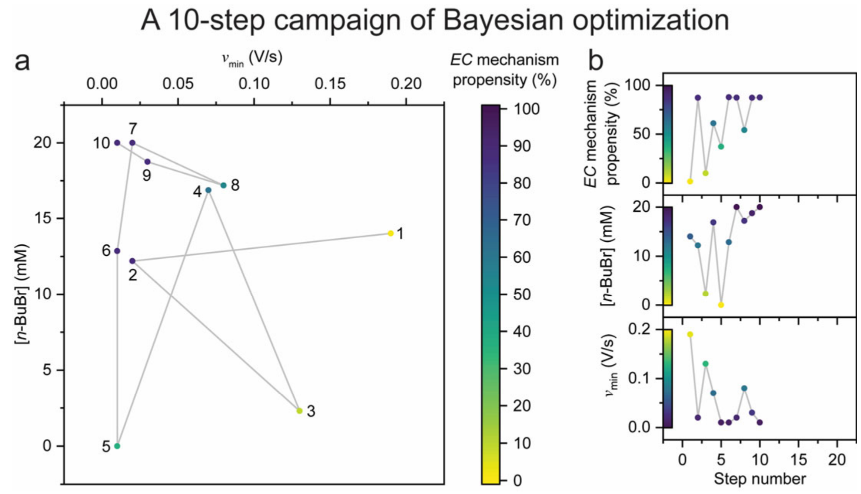

Supplementary Fig. 13. A 10-step campaign of Bayesian optimization. (a) The trajectory in the parameter space of [*n*-BuBr] ∈ [0, 20] mM and v_{min} ∈ [0.01, 0.2] V/s. (b) The DL-generated propensity of an *EC* mechanism, the inquired [*n*-BuBr], and the inquired v_{min} with respect to the step number.

These additional results of Bayesian optimization for such a model system of CoTPP and *n*-BuBr, as summarized below in Supplementary Fig. 14, suggest that a 15-step campaign can effectively converge to the desired maximized propensity of an *EC* mechanism with good statistical reproducibility, yet a 10-step campaign may not be sufficient.

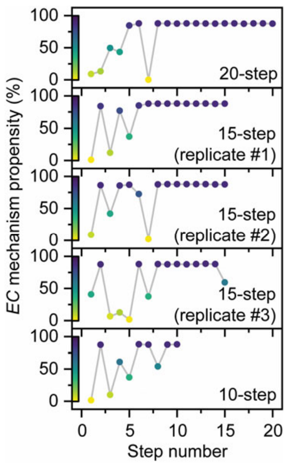

Supplementary Fig. 14. The step-number-dependence of Bayesian optimization. Summarized in this figure are the selected results from Supplementary Figs. 10, 12 and 13.

Therefore, subsequent design of an autonomous closed-loop workflow (Fig. 1f in the main text, Supplementary Fig. 16) is based on a 15-step campaign of Bayesian optimization (6 random sampling and 9 optimization steps) to maximize the voltammograms' propensity toward an *EC* mechanism. To make the closed-loop workflow generally applicable to a diverse scope of RX substrates, Bayesian optimization is deployed over a much wider parameter space of $\log_{10}$[RX] ∈ [$\log_{10}$(0.008 mM), $\log_{10}$(1000 mM)] and v_{min} ∈ [0.01, 0.2] V/s, where the accessible range of [RX] is expanded from [0, 20] mM to [0.008, 1000] mM (if RX can be obtained in plentiful quantities) by introducing additional RX reservoirs in the flow chemistry module of the autonomous electrochemical platform (Supplementary Note 6).

Supplementary Note 5. Extracting kinetic information of the *C* step in an *EC* mechanism from the reverse-to-forward peak current ratio of a voltammogram.

For an *EC* mechanism, a typical approach of extracting kinetic information of the *C* step is based on the reverse-to-forward peak current ratio of a voltammogram[8-10], when the *C* step is neither too slow nor too fast so that the redox feature is partially reversible and the reverse peak of the redox feature is still observed. In the case of an *EC* mechanism between CoTPP and RX, the reverse-to-forward peak current ratio is defined as the anodic-to-cathodic peak current ratio (i_{pa}/i_{pc}) between anodic (i_{pa}) and cathodic (i_{pc}) peak currents of the $Co^{II/I}$ redox. An *EC* mechanism between CoTPP and RX undergoes a bimolecular *C* step:

$$Co^{II}TPP + e^- \rightleftharpoons [Co^{I}TPP]^-$$

$$[Co^{I}TPP]^- + RX \rightarrow R\text{–}Co^{III}TPP + X^-$$

Comparison of an *EC* mechanism with a monomolecular *C* step versus that with a bimolecular *C* step suggests that the forward kinetic rate constant (k_f) of a monomolecular *C* step is equivalent to the observed kinetic rate constant (k_{obs}) of a bimolecular *C* step under a pseudo-first-order condition (see Supplementary Table 7 below).

Supplementary Table 7. An *EC* mechanism with a monomolecular versus a bimolecular *C* step.

	EC mechanism with a monomolecular *C* step	*EC* mechanism with a bimolecular *C* step
Reactions	$O + e^- \rightleftharpoons R$<br>$R \xrightarrow{k_f} A$	$O + e^- \rightleftharpoons R$<br>$R + B \xrightarrow{k_0} C$
Rate law [1]	rate = $k_f\,(C_R)_{x,t}$	rate = $k_0\,(C_R)_{x,t}\,(C_B)_{x,t} = k_{obs}\,(C_R)_{x,t}$

[1] For a monomolecular *C* step, k_f is the forward kinetic rate constant (s^{-1}). For a bimolecular *C* step, k_0 is the second-order kinetic rate constant ($M^{-1}\ s^{-1}$), and k_{obs} is the observed kinetic rate constant (s^{-1}) under a pseudo-first-order condition when B is in large excess so that its spatiotemporal concentration, $(C_B)_{x,t}$, can be considered as constant and is equal to [B], the bulk concentration of B.

The numerical calculations performed by Nicholson and Shain in ref. 8 (as reproduced in Supplementary Table 8 below) defined a working curve of i_{pa}/i_{pc} as a function of the product of k_f (the forward kinetic rate constant of a monomolecular *C* step) and τ (the time in seconds from the formal potential, $E_{1/2}$, of the redox feature to the switching potential, E_{ps}, at the end of the forward scan). Published plots of this working curve are in the form of either i_{pa}/i_{pc} versus $\log_{10}(k_f \cdot \tau)$ (ref. 8) or i_{pa}/i_{pc} versus $(k_f \cdot \tau)$ (ref. 10), but the exact equation of this working curve remains elusive.

Supplementary Table 8. A working curve for kinetic analysis of a monomolecular *C* step in an *EC* mechanism.

$(k_f \cdot \tau)$ [1]	0.004	0.023	0.035	0.066	0.105	0.195	0.350	0.525	0.550	0.778	1.050	1.168	1.557
i_{pa}/i_{pc} [1]	1.00	0.986	0.967	0.937	0.900	0.828	0.727	0.641	0.628	0.551	0.486	0.466	0.415

[1] Adapted from ref. 8.

In order to solve for k_f, an equation in the form of $(k_f \cdot \tau)$ versus i_{pa}/i_{pc} is required. To do so, we empirically applied the 6th order polynomial curve fitting of the as-tabulated data points (see the left panel of Supplementary Fig. 15)

to match with the curvature in a reported plot of the working curve in ref. 10 (see the right panel of Supplementary Fig. 15). The least square fitting results are shown in the following equation (Eq. S1):

$$(k_f \cdot \tau) = 105.82\,(i_{pa}/i_{pc})^6 - 511.14\,(i_{pa}/i_{pc})^5 + 1026.8\,(i_{pa}/i_{pc})^4 - 1100.5\,(i_{pa}/i_{pc})^3 + 667.25\,(i_{pa}/i_{pc})^2 - 220.17\,(i_{pa}/i_{pc}) + 31.962 \qquad \text{(Eq. S1)}$$

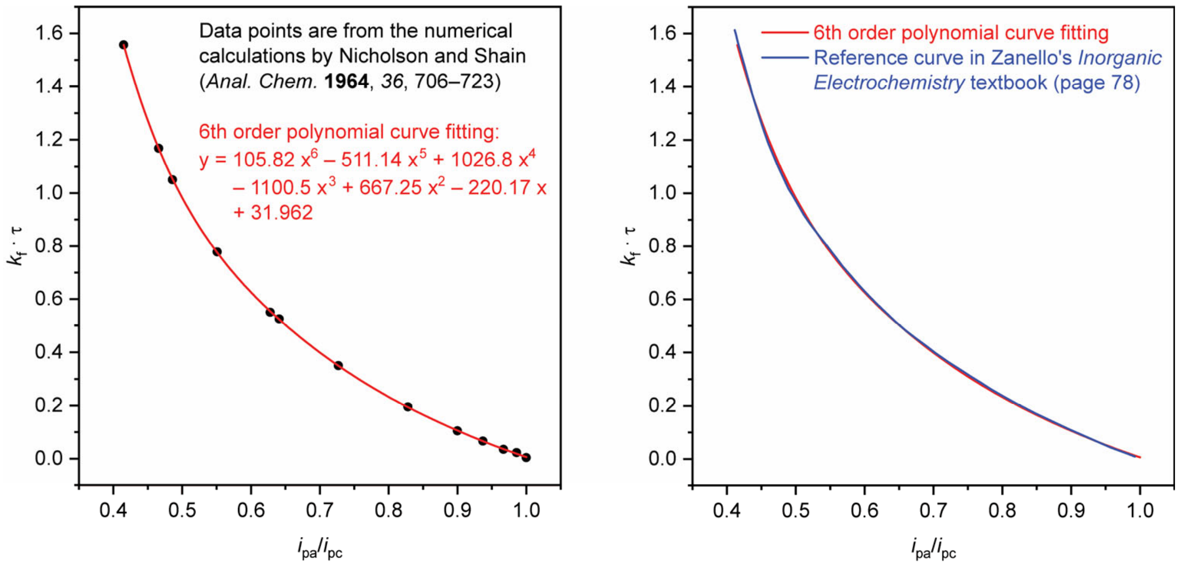

Supplementary Fig. 15. Generation of an empirical equation that matches with the working curve. The left panel shows the empirical curve fitting, which matches well with the working curve as shown in the right panel.

Therefore, Eq. S1 is suitable for deriving ($k_f \cdot \tau$) from experimentally obtained i_{pa}/i_{pc}. Note that, by definition[8, 9], both i_{pa} and i_{pc} need to be reported *with respect to an appropriate baseline* in order to obtain i_{pa}/i_{pc}, which may not be convenient without performing baseline fitting. To overcome this inconvenience, Nicholson introduced an semiempirical procedure[9] that gives an accurate representation of i_{pa}/i_{pc} as a function of readily measurable terms in the first CV cycle (Eq. S2):

$$i_{pa}/i_{pc} = (i_{pa})_0/(i_{pc})_0 + 0.485\,(i_{ps})_0/(i_{pc})_0 + 0.086 \qquad \text{(Eq. S2)}$$

where $(i_{pa})_0$, $(i_{pc})_0$, and $(i_{ps})_0$ are the absolute value of the current *with respect to the zero current axis* at the anodic peak potential, at the cathodic peak potential, and at the switching potential, respectively, all of which are readily measurable. Therefore, our derivation of ($k_f \cdot \tau$) is based on a combination of Eqs. S1 and S2.

To further extract k_f from ($k_f \cdot \tau$), the definition of τ is described as follows (Eq. S3):

$$\tau = |E_{1/2} - E_{ps}| \,/\, v \qquad \text{(Eq. S3)}$$

where $E_{1/2}$ is the formal potential, E_{ps} is the switching potential, and v is the scan rate. Since the redox feature in a voltammogram bearing an *EC* mechanism is complicated by the *C* step, the determination of $E_{1/2}$ from a *single* voltammogram also necessitates Nicholson's semiempirical procedure[9], which suggests a reasonable estimate of $E_{1/2}$ occurring at a point where the current equals 0.8517 $(i_{pc})_0$ for an *EC* mechanism bearing a fast preceding *E* step. Therefore, our derivation of τ is based on the following equations (Eqs. S4 and S5):

$$E_{1/2} = E \text{ at } 0.8517\ (i_{pc})_0 \qquad \text{(Eq. S4)}$$

$$\tau = |[E \text{ at } 0.8517\ (i_{pc})_0] - E_{ps}| / v \qquad \text{(Eq. S5)}$$

The discussions above are based on the forward kinetic rate constant (k_f) of a monomolecular *C* step. Since CoTPP and RX undergo an *EC* mechanism with a bimolecular *C* step, the observed kinetic rate constant (k_{obs}) under a pseudo-first-order condition when RX is in large excess is equivalent to k_f for a monomolecular *C* step (see page S26). Therefore, Eq. S1 can be re-written by substituting k_{obs} for k_f in the equation. To obtain the reaction order (n) of RX, if n = 1, a plot of k_{obs} versus [RX] should be linear (Eq. S6) or a plot of $\log_{10}(k_{obs})$ versus $\log_{10}$[RX] should be linear with a slope of 1 (Eq. S7). From Eqs. S6 and S7, the second-order kinetic rate constant (k_0) of the bimolecular *C* step between the electrogenerated Co^{I}TPP and RX can be extracted.

$$k_f = k_{obs} = k_0\ [\mathrm{RX}]^n \qquad \text{(Eq. S6)}$$

$$\log_{10}(k_f) = \log_{10}(k_{obs}) = \log_{10}(k_0) + n \log_{10}[\mathrm{RX}] \qquad \text{(Eq. S7)}$$

As a concluding remark of this Supplementary Note 5, for an *EC* mechanism, extracting kinetic information of the *C* step from i_{pa}/i_{pc} requires the *C* step being neither too fast nor too slow, corresponding to a partially reversible redox feature in a voltammogram where the reverse peak is still observed (see page S26). If the *C* step is too fast so that the reverse peak completely diminishes in a voltammogram, then kinetic information of the *C* step in an *EC* mechanism should be extracted using an alternative approach based on the forward peak position[11, 12]. In the context of an *EC* mechanism between CoTPP and RX, for certain RX substrates exhibiting very high reactivities toward the electrogenerated Co^{I}TPP, the i_{pa}/i_{pc} approach becomes inapplicable for kinetic analysis of the *C* step, and the as-mentioned alternative approach needs to be used (see more discussions in Supplementary Note 10).

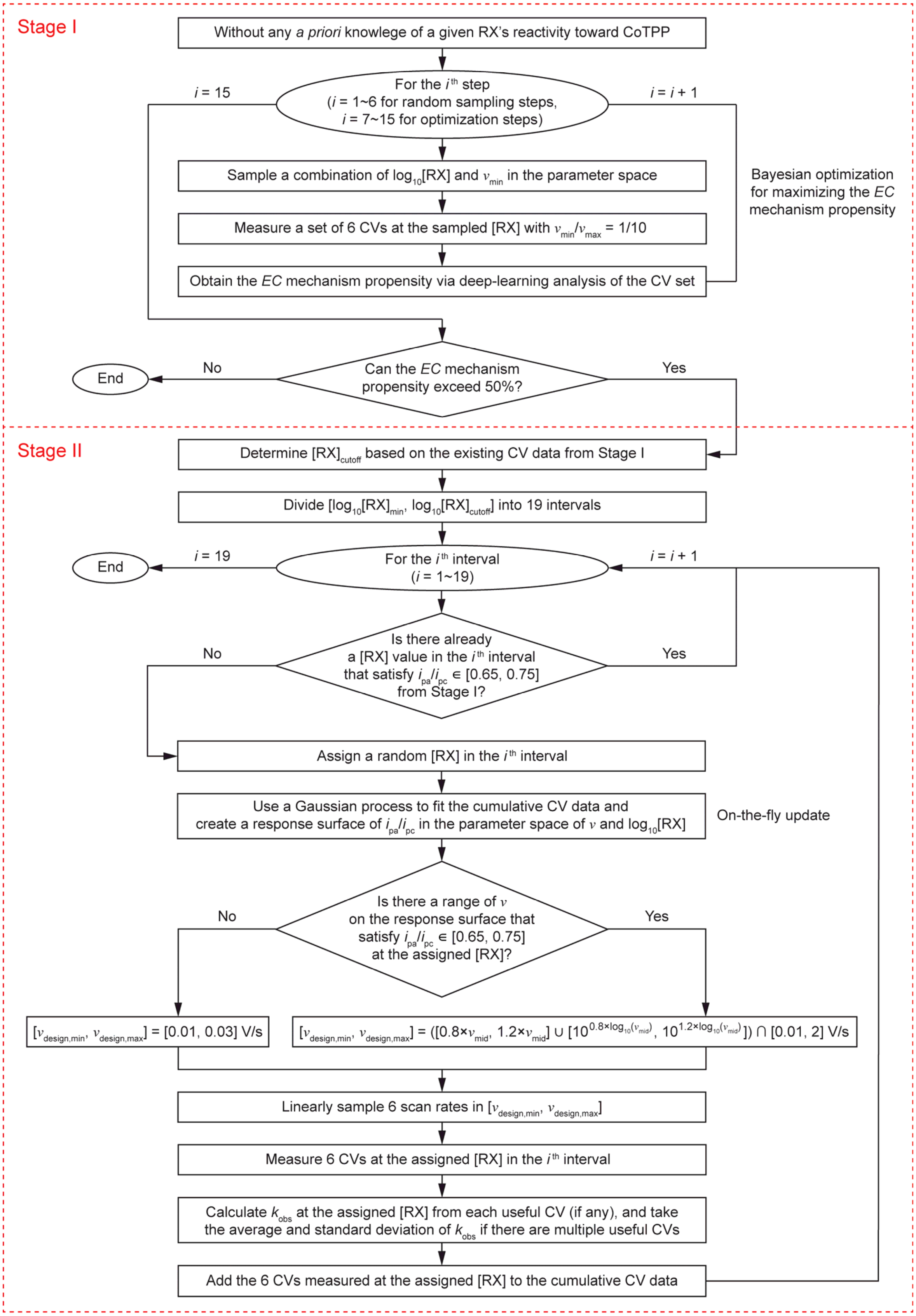

Supplementary Fig. 16. A flowchart illustrating the design of a closed-loop workflow for autonomous investigations of an *EC* mechanism between CoTPP and a library of organohalide (RX) substrates. Stage I for mechanism discernment and Stage II for electrokinetic analysis. Details about the workflow are provided in the rest part of this Supplementary Information.

Supplementary Note 6. Introduction of additional RX reservoirs in the flow chemistry module to expand the accessible range of [RX] for a generally applicable closed-loop workflow.

The as-described autonomous electrochemical platform in Supplementary Notes 1 and 2 (also in Figs. 1b and 1c in the main text) employs a single RX reservoir (40 mM RX in DMF with 0.1 M NBu_4PF_6) to access a finite range of [RX] ∈ [0, 20] mM for automated exhaustive CV experiments (Fig. 2 in the main text, Supplementary Note 3) and Bayesian optimization trials (Supplementary Note 4) when RX = *n*-BuBr.

To construct a generally applicable autonomous closed-loop workflow (Fig. 1f in the main text, Supplementary Fig. 16) for investigating an *EC* mechanism between CoTPP and a diverse scope of RX substrates whose reactivities may vary greatly, we introduce additional RX reservoirs in the flow chemistry module to expand the accessible range of [RX] from [0, 20] mM to [0.008, 1000] mM, as depicted below in Supplementary Fig. 17.

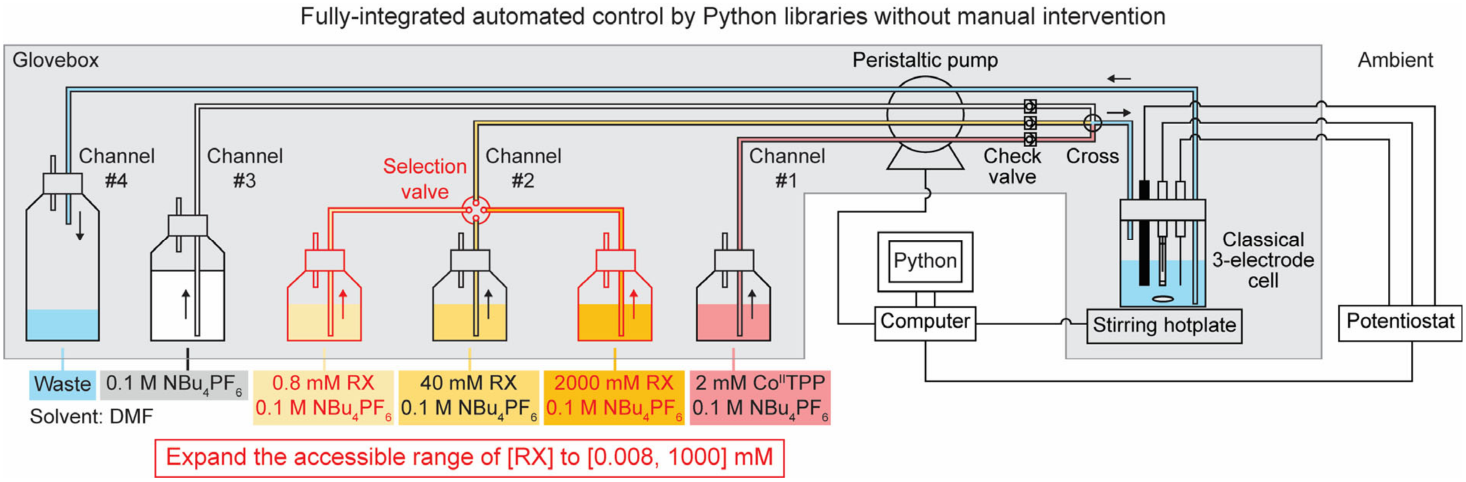

Supplementary Fig. 17. The modified autonomous electrochemical platform with additional RX reservoirs. The additional 0.8 mM and 2000 mM RX reservoirs expand the accessible range of [RX] to [0.008, 1000] mM.

Hardware modification of the flow chemistry module involves the addition of a selection valve, two more solution reservoirs, and associated fittings (highlighted in red in Supplementary Fig. 17 above and listed in Supplementary Table 9 below).

Supplementary Table 9. Hardware modification of the flow chemistry module.

Hardware	Manufacturer, Model, and Part Number	Quantity	Total Price
Modifications of the Flow System	IDEX Actuated Selection Valve, 6-Position, 7-Port, 6000 psi, DuraLife II/Titanium Wetting Materials (MXP7970-000)	1 Piece	US $1,900
	IDEX Fingertight One-Piece PEEK Fitting, 10-32 Coned Port, for 1/16"OD tubing (6000-282MC)	4 Pieces	US$ 26
	IDEX Super Flangeless Ferrule with Stainless Steel Ring, 1/4"-28 Flat-Bottom, for 1/16" OD tubing (P-250X)	4 Pieces	US$ 16
	IDEX Super Flangeless Nut, 1/4"-28 Flat-Bottom (P-255X)	4 Pieces	US$ 14
	IDEX Port Plug, Black Polypropylene, 1/4"-28 Flat-Bottom (D-324BLK)	4 Pieces	$US 8

Modifications of the Flow System (Continued)	Cole-Parmer VapLock Solvent Delivery Cap, with Four Stainless Steel 1/4”-28 Threaded Ports, GL45 (EW-12018-02)	2 Pieces	US$ 112
	Corning Pyrex 500 mL Medium Bottle, with GL45 Screw Cap (1395-500)	2 Pieces	US$ 32
The Autonomous Electrochemical Platform Prior to Modifications (See Supplementary Note 1)			US$ 21,306
Sum of Total Price			US$ 23,414

Below in Supplementary Fig. 18, further instructions are provided on how to modify the flow chemistry module using the additional parts (as listed in Supplementary Table 9 above), where hardware modification is highlighted inside a dashed red frame.

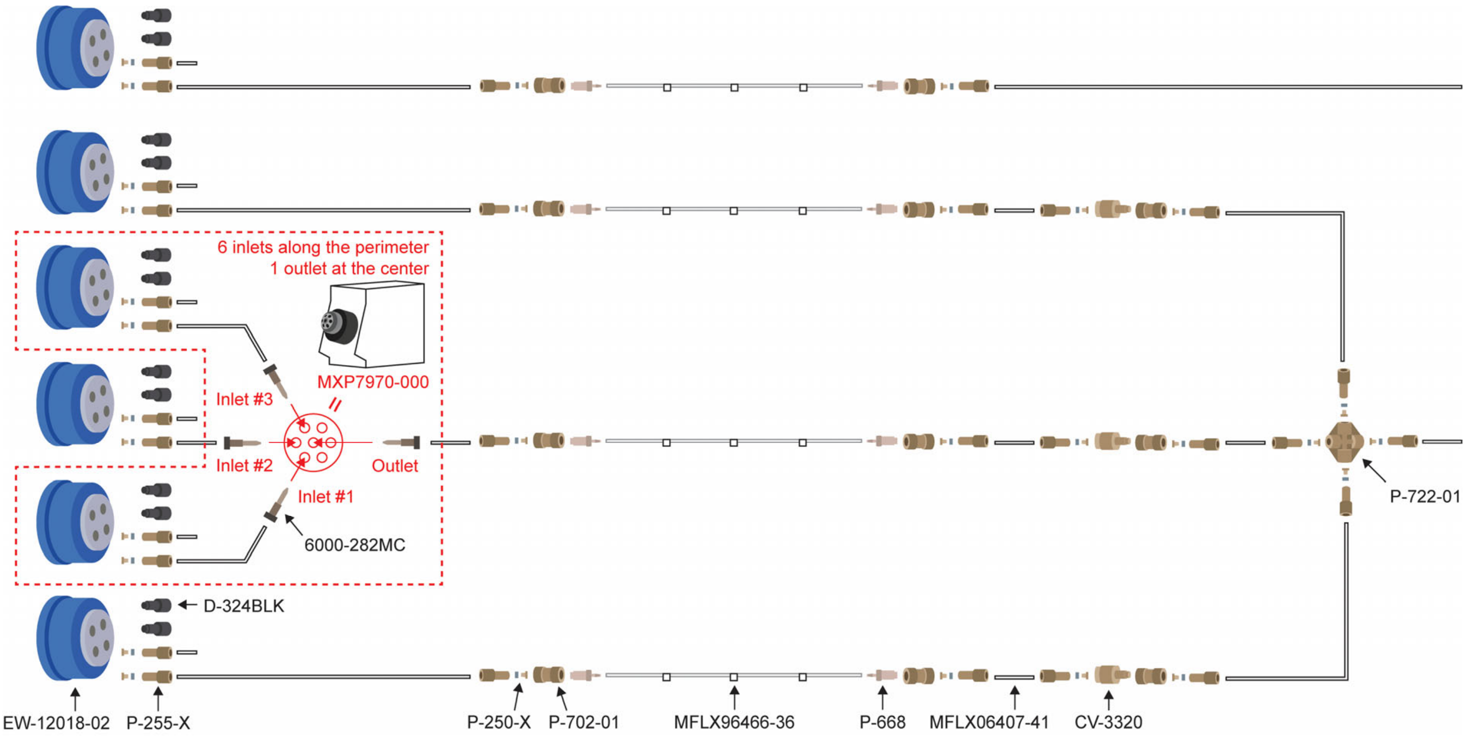

Supplementary Fig. 18. Instructions on how to modify the flow chemistry module. Hardware modification (as listed in Supplementary Table 9 above) is highlighted inside a dashed red frame. The selection valve used in this work has six inlets along the perimeter and one outlet at the center. The part numbers of the selection valve, the Masterflex tubings, the IDEX fittings, and other accessories (as listed in Supplementary Table 9 above as well as in Supplementary Table 1 in Supplementary Note 1) are annotated in this figure. The schematics of the parts are drawn to reflect their actual shapes.

The rationales behind using 0.8 mM, 40 mM, and 2000 mM RX reservoirs to access an expanded range of [RX] ∈ [0.008, 1000] mM are as follows. As described in Supplementary Note 3, [Co^{II}TPP] = 1 mM is kept constant and [RX] is varied during automated electrolyte formulation. The rotation speed of Channel #1 (which flows 2 mM Co^{II}TPP stock solution) is set to be 50 rpm, and the combined rotation speeds of Channel #2 (which flows RX stock solution) and Channel #3 (which flows NBu_4PF_6 stock solution) are set to be 50 rpm. Each running channel has a minimum rotation speed of 1.0 rpm and a step resolution of 0.01 rpm. The 0.8 mM, 40 mM, and 2000 mM RX reservoirs are connected to the selection valve inlets #1, #2, and #3, respectively. One of the three RX reservoirs is

selected and fed to Channel #2 via the selection valve outlet. The accessible range of [RX] and the step resolution of accessible [RX] are derived in Supplementary Table 10 below.

Supplementary Table 10. Derivation of the accessible range of [RX] for the modified platform.

Rotation speed settings	Selection valve inlet	Selected RX reservoir fed to Channel #2	Accessible range of [RX] [1]	Step resolution of accessible [RX]
Channel #1 = 50 rpm<br>Channel #2 = [1, 50] rpm<br>Channel #2 + Channel #3 = 50 rpm	#1	0.8 mM RX	[0.008, 0.4] mM	8×10^{-5} mM
	#2	40 mM RX	[0.4, 20] mM	0.004 mM
	#3	2000 mM RX	[20, 1000] mM	0.2 mM
Overall accessible range of [RX]			[0.008, 1000] mM	

[1] For a borderline [RX] = 0.4 or 20 mM that is accessible by feeding either a dilute RX reservoir at 50 rpm speed or a concentrated RX reservoir at 1 rpm, our algorithm arbitrarily assigns the latter option: feeding the 40 mM RX reservoir at 1 rpm to access [RX] = 0.4 mM, and feeding the 2000 mM RX reservoir at 1 rpm to access [RX] = 20 mM.

The annotated photographs of the modified platform with additional RX reservoirs are shown in Supplementary Fig. 19 below.

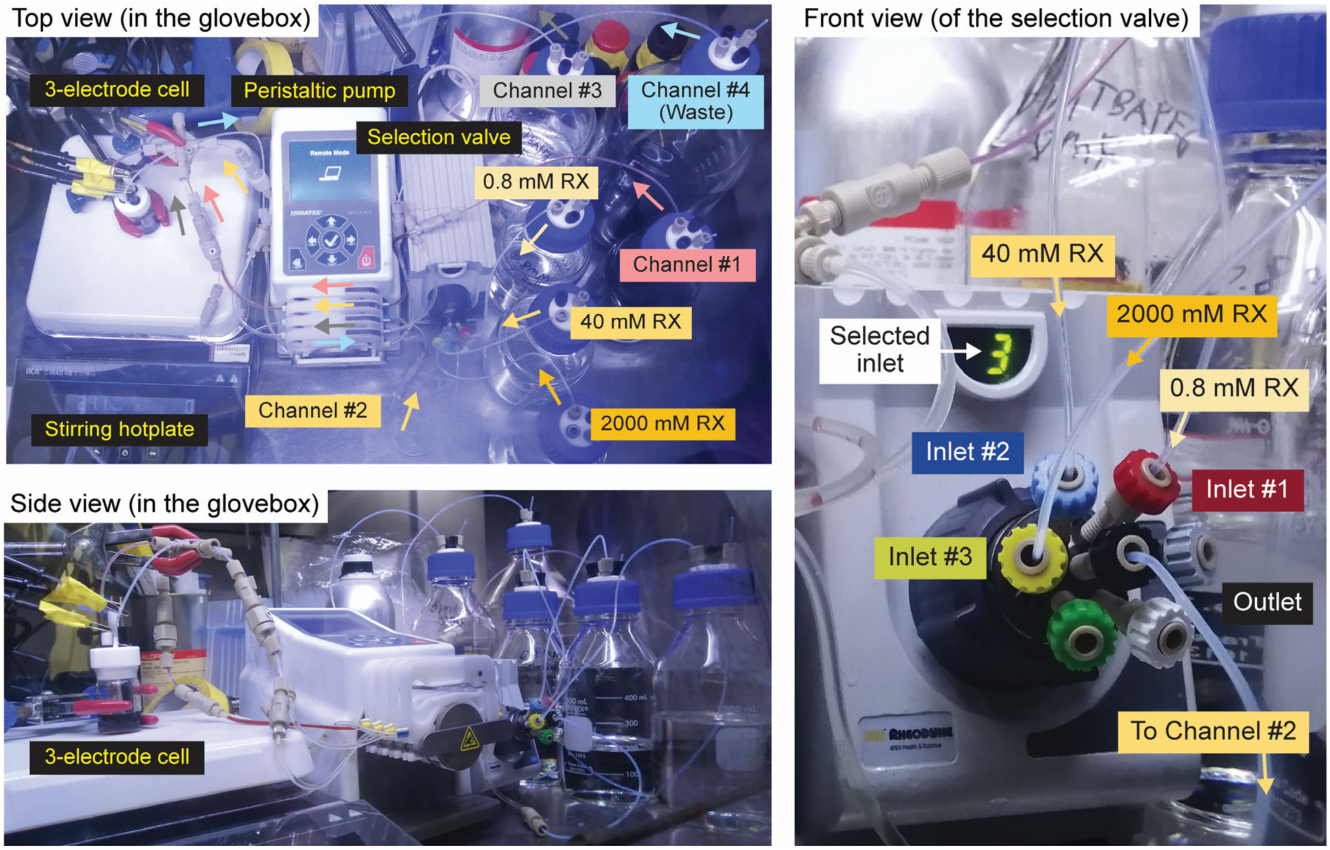

Supplementary Fig. 19. Annotated photographs of the modified platform. For comparison, photographs of the platform before modification are shown in Supplementary Fig. 2 in Supplementary Note 1.

In the modified platform, the three RX reservoirs share the same Channel #2, and there is a nontrivial volume of solution in the tubing section between the selection valve outlet and the cross body (see Supplementary Fig. 17 in this Supplementary Note 6). To avoid cross-contamination from different RX reservoirs and ensure an accurate

[RX] in the formulated electrolyte composition, we modified the protocols for automated electrolyte formulation and disposal (compared to those described in Supplementary Fig. 6 in Supplementary Note 2) by adding additional rinsing of Channel #2 using the selected RX reservoir. The modified protocols are shown below in Supplementary Fig. 20, with the changes highlighted in red.

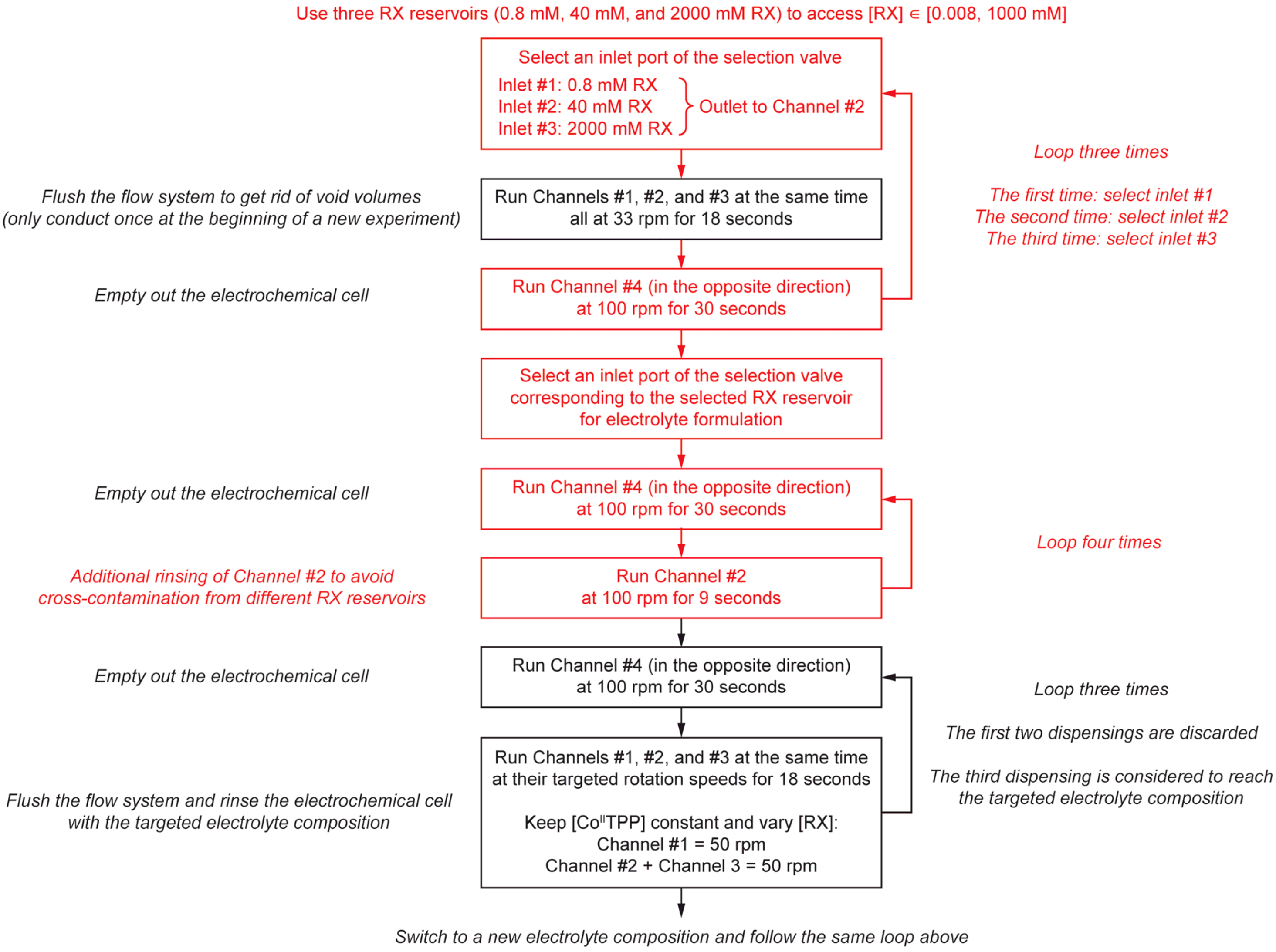

Supplementary Fig. 20. Modified protocols for automated electrolyte formulation and disposal when three RX reservoirs are used. The protocols in this flowchart are applicable to the modified platform (as depicted in Supplementary Fig. 17) when all three (0.8 mM, 40 mM, and 2000 mM) RX reservoirs are used. The changes made to accommodate the introduction of additional RX reservoirs (compared to those described in Supplementary Fig. 6 in Supplementary Note 2) are highlighted in red.

In this work, if a given RX substrate can be obtained in plentiful quantities (which applies to most of the studied RX substrates), autonomous investigation by the closed-loop workflow is conducted using three RX reservoirs (0.8 mM, 40 mM, and 2000 mM RX) to access [RX] ∈ [0.008, 1000] mM. If not, it becomes impractical to prepare the 2000 mM RX reservoir with a sufficient volume for autonomous investigation, and thus only the 0.8 mM and 40 mM RX reservoirs are used to access [RX] ∈ [0.008, 20] mM. For such a special case where only two of the three

RX reservoirs are used due to chemical shortage, the modified protocols for automated electrolyte formulation and disposal are shown below in Supplementary Fig. 21, with the additional changes highlighted in blue compared to a typical case where all three RX reservoirs are used.

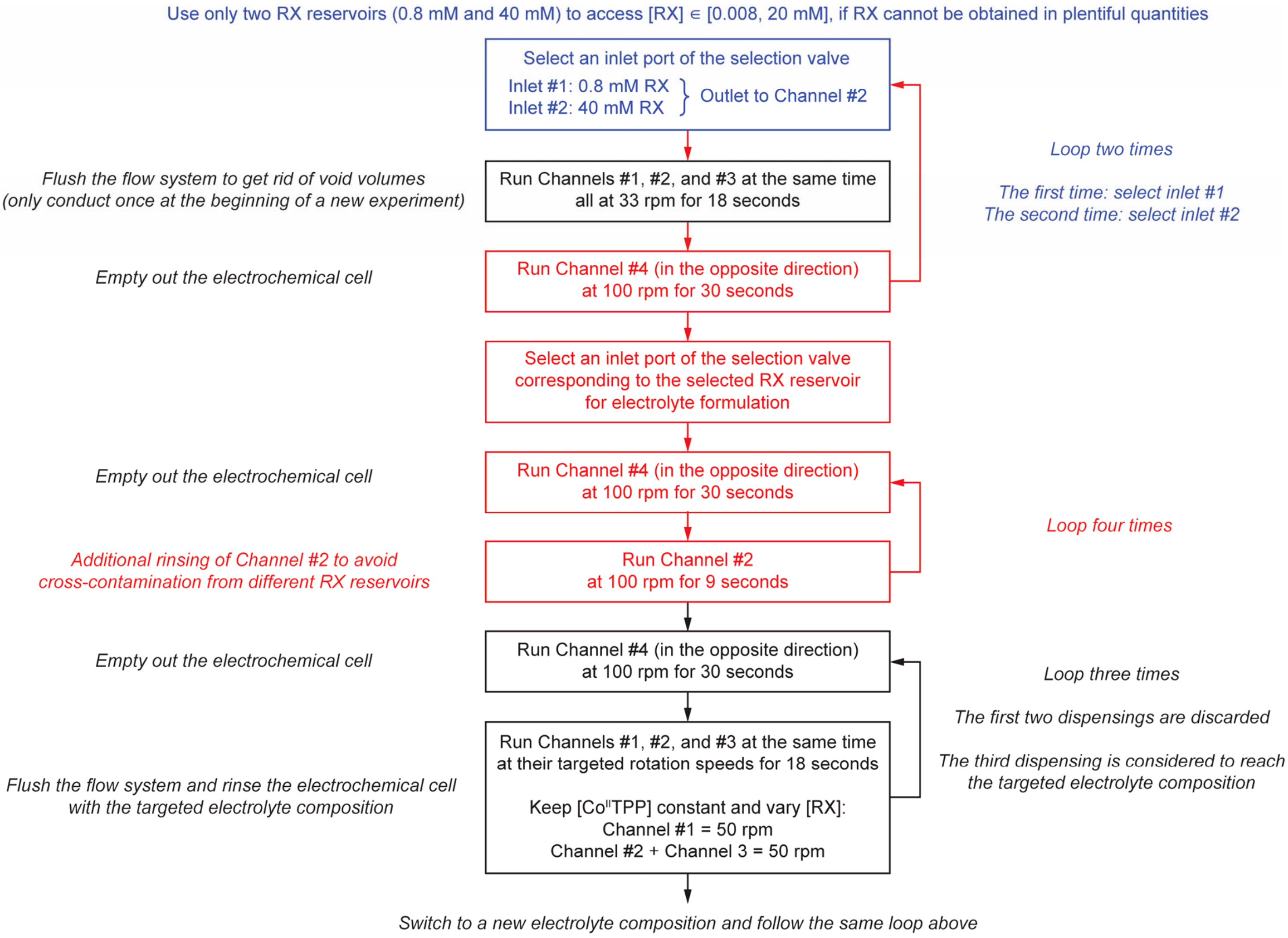

Supplementary Fig. 21. Modified protocols for automated electrolyte formulation and disposal when only two RX reservoirs are used. The protocols in this flowchart are applicable to the modified platform (as depicted in Supplementary Fig. 17) when only two (0.8 mM and 40 mM) RX reservoirs are used. The changes made to accommodate the introduction of additional RX reservoirs (compared to those described in Supplementary Fig. 6 in Supplementary Note 2) are highlighted in red. The additional changes (compared to a typical case where all three RX reservoirs are used as described in Supplementary Fig. 20) are highlighted in blue.

Supplementary Note 7. Determination of a cutoff RX concentration, $[RX]_{cutoff}$, for electrokinetic analysis in Stage II of the closed-loop workflow.

Since Bayesian optimization in Stage I of the closed-loop workflow aims at maximizing the voltammograms' propensity, obtained from the DL model, toward an *EC* mechanism, a majority of the CV data in Stage I are deemed to be not suitable for measuring the rate (k_0) of the *C* step because their i_{pa}/i_{pc} values are well below the range of [0.65, 0.75]. Therefore, right after Stage I, to increase the chance of locating suitable parameter combinations for electrokinetic analysis in Stage II, we developed a customized algorithm to determine a cutoff RX concentration, $[RX]_{cutoff}$, below which avoids accessing unnecessarily high [RX] values in future inquiries in Stage II.

Our customized algorithm applies a Gaussian process[2, 3] to fit the existing values of the DL-generated propensity of an *EC* mechanism, obtained from a 15-step campaign of Bayesian optimization in Stage I, under 15 combinations of v_{min} and $\log_{10}$[RX] (v_{min} ∈ [0.01. 0.2] V/s and $\log_{10}$[RX] ∈ [$\log_{10}$(0.008 mM), $\log_{10}$(1000 mM)]). As a result, a response surface of the *EC* mechanism propensity is created in the parameter space of v_{min} and $\log_{10}$[RX].

For example, right after Stage I of the closed-loop workflow when RX = *n*-BuBr (see Supplementary Fig. 24 for the corresponding 15-step campaign of Bayesian optimization), the created response surface of the *EC* mechanism propensity is shown below in Supplementary Fig. 22. Since a limited number of data points is fitted to a large parameter space, the local model uncertainty from the Gaussian process (the standard deviation of the modelled *EC* mechanism propensity) can be quite large in some regions. (Also, in retrospect, we should have constrained v_{min} ∈ [0.01. 0.2] V/s rather than [0.01, 2] V/s when creating the response surface of the *EC* mechanism propensity.)

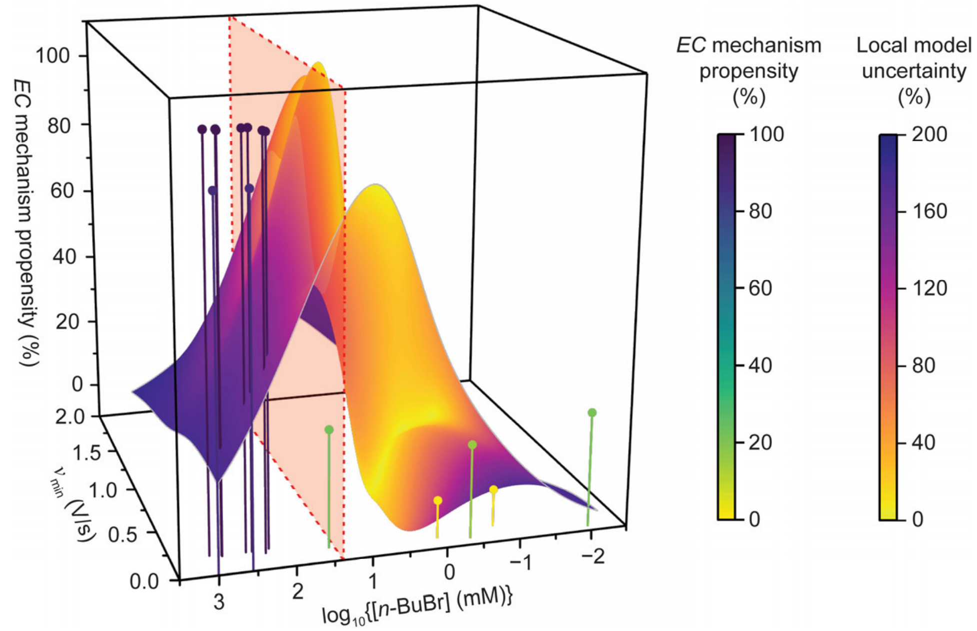

Supplementary Fig. 22. The created response surface of the *EC* mechanism propensity right after Stage I of the closed-loop workflow when RX = *n*-BuBr. The dots represent the obtained *EC* mechanism propensity values from Stage I of the workflow**.** The colors of the surface represent the local model uncertainty from the Gaussian process (the standard deviation of the modelled *EC* mechanism propensity).

After a response surface of the *EC* mechanism propensity is created, a number filter is then applied to only keep part of the response surface where the *EC* mechanism propensity ∈ (0%, 80%). From the filtered response surface, the point where the *EC* mechanism propensity is closest to 80% is identified, and the corresponding [RX] value is determined to be $[RX]_{cutoff}$. Following these rationales, in the as-mentioned case when RX = *n*-BuBr, $[n\text{-BuBr}]_{cutoff}$ is determined to be 24.3 mM (see the vertical red plane in Supplementary Fig. 22 on the last page).

Supplementary Note 8. Design of suitable parameter combinations for electrokinetic analysis in Stage II of the closed-loop workflow.

In Stage II of the closed-loop workflow, after determining $[RX]_{cutoff}$ (Supplementary Note 7), the concentration range between the minimal [RX] ($[RX]_{min}$ = 0.008 mM, Supplementary Note 6) and $[RX]_{cutoff}$ is equally divided into 19 intervals on the logarithmic scale, within each of which a randomly sampled [RX] value is assigned. In Stage II, these 19 assigned [RX] values are experimentally inquired in an incremental manner. For each assigned [RX] value, it is important to design suitable v values that may satisfy $i_{pa}/i_{pc} \in [0.65, 0.75]$ for measuring the rate (k_0) of the C step. Below we describe how our customized algorithm designs suitable v values under each assigned [RX] value for electrokinetic analysis in Stage II of the closed-loop workflow.

Prior to Stage II, a 15-step campaign of Bayesian optimization in Stage I measures a total of 90 voltammograms in the parameter space of v and $\log_{10}$[RX]. Right after Stage I, a Gaussian process[2, 3] is applied to fit the i_{pa}/i_{pc} values of these 90 voltammograms, creating an initial response surface of i_{pa}/i_{pc} in the parameter space of v and $\log_{10}$[RX] (see Fig. 3c in the main text for an example when RX = n-BuBr). As Stage II proceeds, this response surface of i_{pa}/i_{pc} is updated on-the-fly based on the cumulative CV data, every time after an assigned [RX] value is experimentally inquired (see Supplementary Fig. 25 for an example when RX = n-BuBr). Since there may or may not exist a range of v values on the response surface that satisfy $i_{pa}/i_{pc} \in [0.65, 0.75]$ under an assigned [RX] value, the following discussions are divided into two scenarios.

Scenario (1) where there exists a range of v values on the response surface that satisfy $i_{pa}/i_{pc} \in [0.65, 0.75]$ under an assigned [RX] value. If an assigned [RX] value falls in this scenario, from the range of v values on the response surface that satisfy $i_{pa}/i_{pc} \in [0.65, 0.75]$, the algorithm defines its midpoint as v_{mid} (V/s). To accommodate the local model uncertainty from the Gaussian process (the standard deviation of the modelled i_{pa}/i_{pc}), a slightly extended yet narrow range of v values surrounding the as-defined v_{mid} is designed by the algorithm to increase the chance of finding suitable v values for electrokinetic analysis. To do so, the algorithm first considers deviating from v_{mid} by ±20% either on the linear scale ($[0.8\times v_{mid}, 1.2\times v_{mid}]$) or on the logarithmic scale ($[10^{0.8\times\log_{10}(v_{mid})}, 10^{1.2\times\log_{10}(v_{mid})}]$), then take the union (∪) of these two ranges to make a slightly extended range, and finally take the intersection (∩) of this slightly extended range and [0.01, 2] V/s to constrain the designed range ($[v_{design,min}, v_{design,max}]$) within the parameter space:

$$[v_{design,min}, v_{design,max}] = ([0.8\times v_{mid}, 1.2\times v_{mid}] \cup [10^{0.8\times\log_{10}(v_{mid})}, 10^{1.2\times\log_{10}(v_{mid})}]) \cap [0.01, 2] \text{ V/s} \qquad \text{(Eq. S8)}$$

Afterwards, 6 proximate v values are linearly sampled from $[v_{design,min}, v_{design,max}]$, with $v_{design,min}$ and $v_{design,max}$ being the two end members. For an an assigned [RX] value that falls in this scenario, an experimental inquiry is conducted under 6 proximate v values. Upon completion, the newly measured 6 voltammograms are appended to the cumulative CV data, and the same Gaussian process is applied again to fit the cumulative CV data and update the

response surface of i_{pa}/i_{pc} on-the-fly. For the next assigned [RX] value, the design of [$v_{design,min}$, $v_{design,max}$] is based on the updated response surface of i_{pa}/i_{pc}, and so on and so forth.

Scenario (2) where there does not exist a range of *v* values on the response surface that satisfy $i_{pa}/i_{pc} \in [0.65, 0.75]$ under an assigned [RX] value. This scenario indicates that, under an assigned [RX] value, the *C* step is either too slow or too fast. Nevertheless, it is still meaningful to conduct experimental inquiry under such an assigned [RX] value. This is because measuring more voltammograms and appending them to the cumulative CV data are helpful for the Gaussian process to create a more accurate response surface of i_{pa}/i_{pc} through on-the-fly update. We reason that, when the *C* step is either too slow or too fast, it is useful to collect voltammograms under the lowest *v* value (0.01 V/s) in the parameter space: (1) for a slow *C* step, the lowest *v* value maximizes the chance (if any) to bring i_{pa}/i_{pc} below 0.75 for kinetic analysis using the i_{pa}/i_{pc} approach (Supplementary Note 5); (2) for a fast *C* step, even the highest *v* value in the parameter space cannot bring i_{pa}/i_{pc} above 0.65 so that kinetic analysis using the i_{pa}/i_{pc} approach becomes invalid, but on the other hand, the lowest *v* value allows for accurate measurement of the cathodic peak potential (E_{pc}) so that kinetic analysis using an alternative approach based on E_{pc} becomes possible (Supplementary Note 10). To design a narrow range of *v* values that contains the lowest *v* value (0.01 V/s), the algorithm still uses the same Eq. S8 shown above for the sake of consistency, but arbitrarily set v_{mid} = 0.0125 V/s (although v_{mid} does not exist in this scenario), yielding [$v_{design,min}$, $v_{design,max}$] = [0.01, 0.03] V/s. For an assigned [RX] value that falls in this scenario, an experimental inquiry is conducted under 6 proximate *v* values that are linearly sampled from the designed range (0.01, 0.014, 0.018, 0.022, 0.026, and 0.03 V/s). The on-the-fly update of the response surface of i_{pa}/i_{pc} also applies to this scenario.

Finally, we direct the readers to Supplementary Fig. 16 for a flowchart illustrating the design of the closed-loop workflow, which includes relevant information described in this Supplementary Note 8.

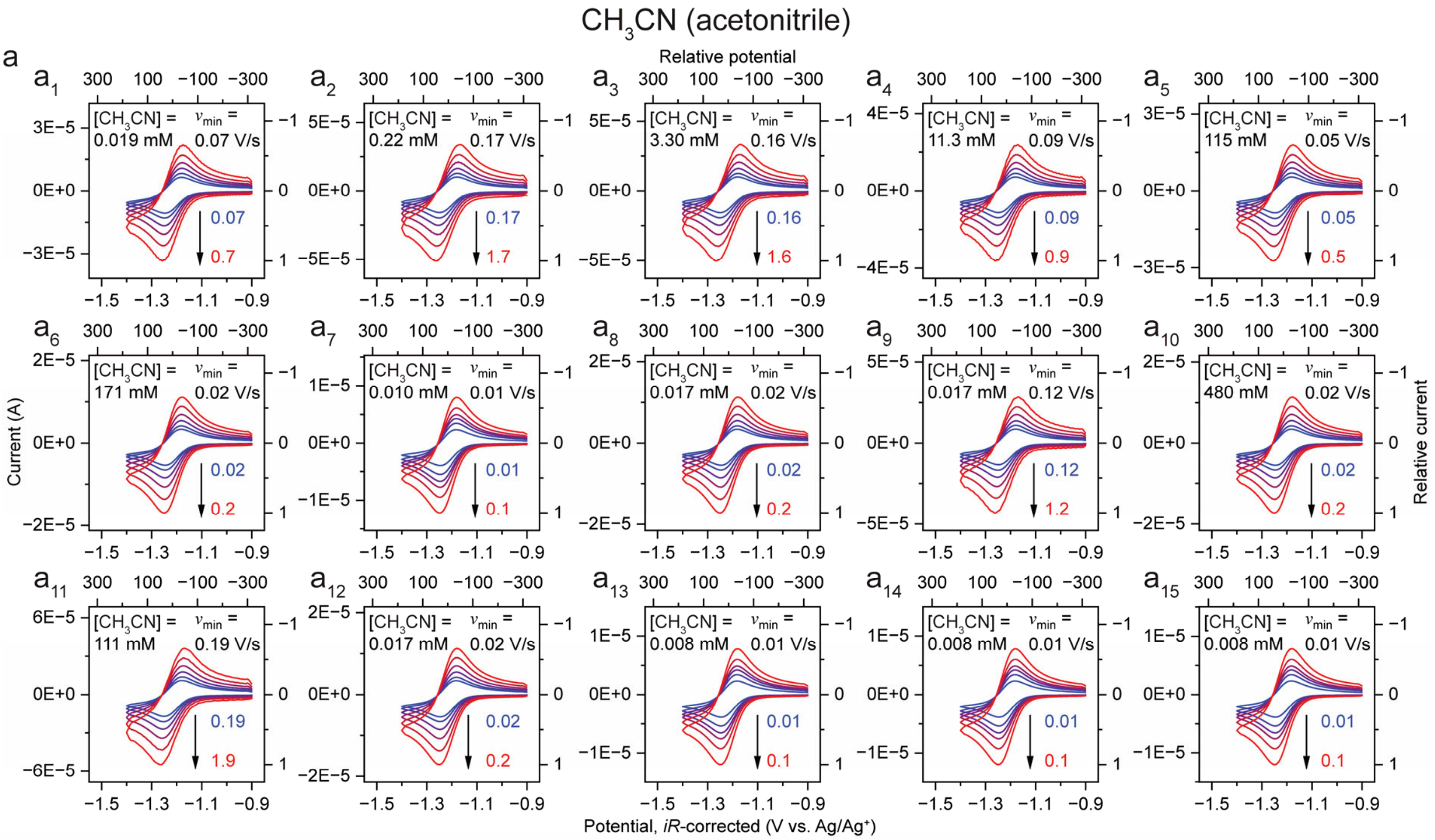

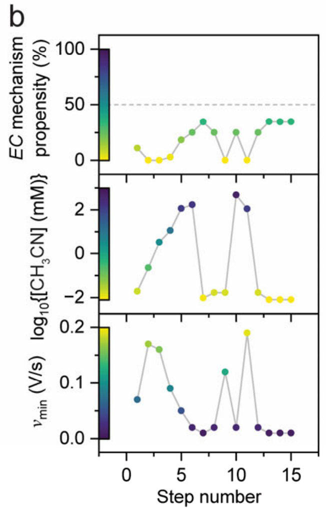

c

Potential range: between −0.9 and −1.4 V vs. Ag/Ag+

Step	$[CH_3CN]$ (mM)	v_{min} (V/s)	Mechanism propensity (%)				
			E	*EC*	*CE*	*DISP1*	*ECE*
1	0.019	0.07	89.0	11.0	0.0	0.0	0.0
2	0.22	0.17	100.0	0.0	0.0	0.0	0.0
3	3.30	0.16	100.0	0.0	0.0	0.0	0.0
4	11.3	0.09	97.3	2.7	0.0	0.0	0.0
5	115	0.05	81.7	18.3	0.0	0.0	0.0
6	171	0.02	75.0	25.0	0.0	0.0	0.0
7	0.010	0.01	65.5	34.5	0.0	0.0	0.0
8	0.017	0.02	75.0	25.0	0.0	0.0	0.0
9	0.017	0.12	100.0	0.0	0.0	0.0	0.0
10	480	0.02	75.0	25.0	0.0	0.0	0.0
11	111	0.19	100.0	0.0	0.0	0.0	0.0
12	0.017	0.02	75.0	25.0	0.0	0.0	0.0
13	0.008	0.01	65.2	34.8	0.0	0.0	0.0
14	0.008	0.01	65.4	34.6	0.0	0.0	0.0
15	0.008	0.01	65.3	34.7	0.0	0.0	0.0

Supplementary Fig. 23. Autonomous investigation of CH_3CN (acetonitrile) with an undetected reactivity toward CoTPP in the parameter space. (a) CV data (1 mM Co^{II}TPP in DMF with 0.1 M NBu_4PF_6, $[CH_3CN] \in [0.008, 1000]$ mM, $v_{min} \in [0.01, 0.2]$ V/s) measured from a 15-step campaign of Bayesian optimization in Stage I of the closed-loop workflow, with detailed parameters and Bayesian optimization results summarized in (b) and (c). As the DL-generated propensity of *EC* mechanism did not exceed the 50% threshold, the closed-loop workflow autonomously ended after Stage I and did not proceed to Stage II (see the flowchart in Supplementary Fig. 16).

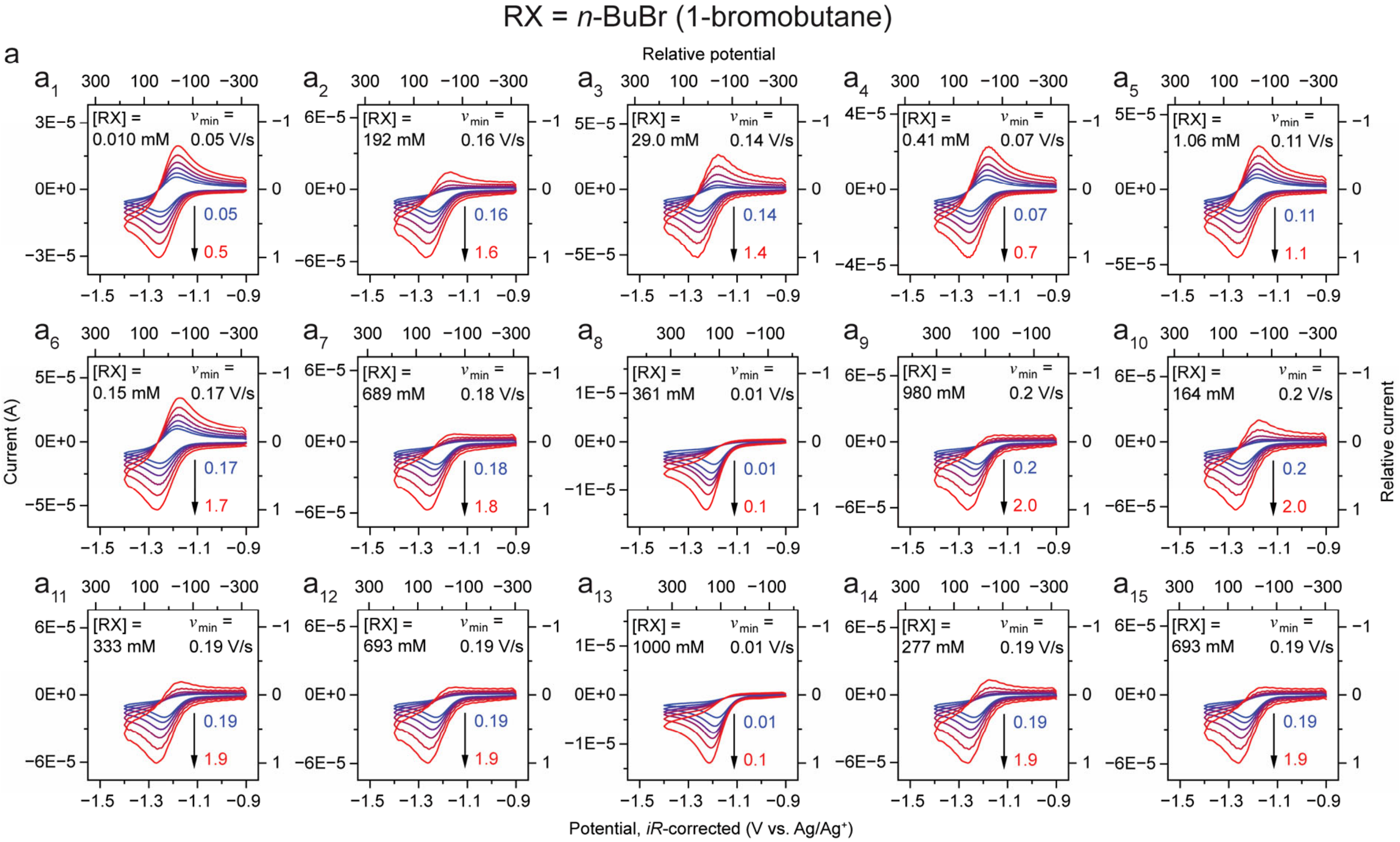

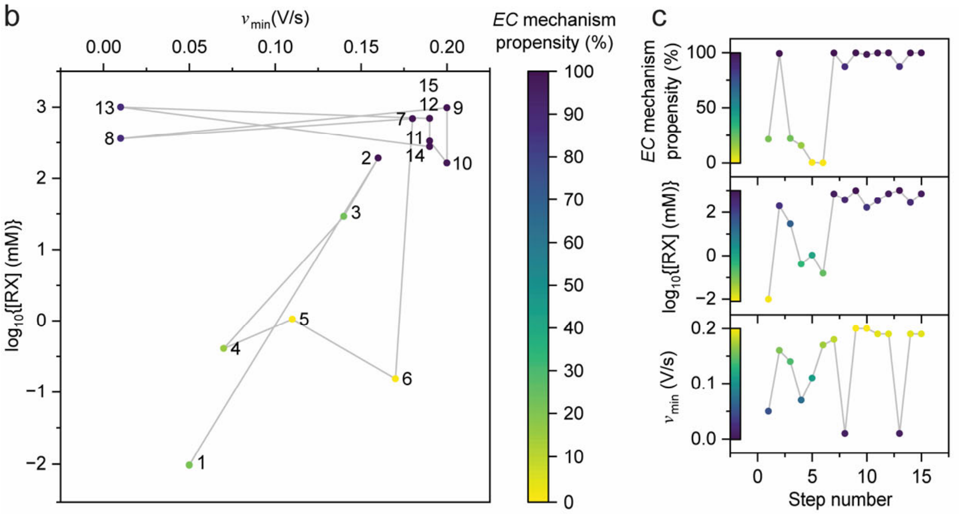

d

Potential range: between −0.9 and −1.4 V vs. Ag/Ag+

Step	[RX] (mM)	v_{min} (V/s)	Mechanism propensity (%)				
			E	*EC*	*CE*	*DISP1*	*ECE*
1	0.010	0.05	78.5	21.5	0.0	0.0	0.0
2	192	0.16	0.8	99.2	0.0	0.0	0.0
3	29.0	0.14	78.0	22.0	0.0	0.0	0.0
4	0.41	0.07	84.4	15.6	0.0	0.0	0.0
5	1.06	0.11	99.7	0.3	0.0	0.0	0.0
6	0.15	0.17	100.0	0.0	0.0	0.0	0.0
7	689	0.18	0.3	99.6	0.0	0.0	0.0
8	361	0.01	12.6	87.4	0.0	0.0	0.0
9	980	0.2	0.2	99.8	0.0	0.0	0.0
10	164	0.2	1.6	98.4	0.0	0.0	0.0
11	333	0.19	0.3	99.7	0.0	0.0	0.0
12	693	0.19	0.2	99.8	0.0	0.0	0.0
13	1000	0.01	12.5	87.5	0.0	0.0	0.0
14	277	0.19	0.3	99.7	0.0	0.0	0.0
15	693	0.19	0.2	99.8	0.0	0.0	0.0

Supplementary Fig. 24. Autonomous discernment of an *EC* mechanism between CoTPP and *n*-BuBr based on a 15-step campaign of Bayesian optimization in Stage I of the closed-loop workflow. (a) CV data (1 mM Co^{II}TPP in DMF with 0.1 M NBu_4PF_6, [RX] ∈ [0.008, 1000] mM, v_{min} ∈ [0.01, 0.2] V/s) measured from Stage I, with the trajectory of a 15-step campaign of Bayesian optimization shown in (b) and detailed parameters and Bayesian optimization results summarized in (c) and (d). As the DL-generated propensity of *EC* mechanism far exceeded the 50% threshold, the closed-loop workflow autonomously discerned an *EC* mechanism between CoTPP and *n*-BuBr from Stage I and proceeded to Stage II (see Supplementary Fig. 26 for CV data measured from Stage II).

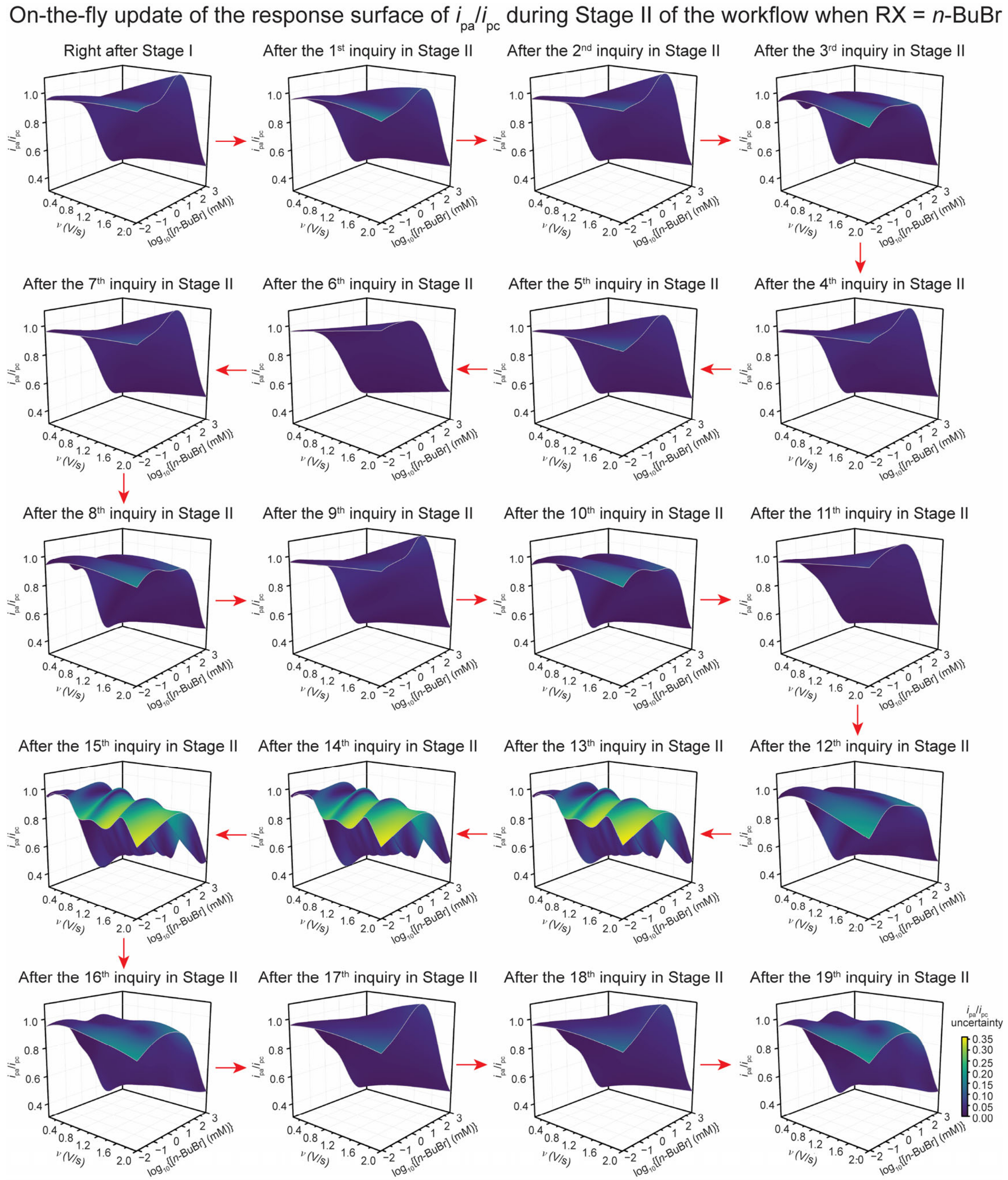

Supplementary Fig. 25. On-the-fly update of the response surface of i_{pa}/i_{pc} during Stage II of the closed-loop workflow when RX = *n*-BuBr. The colors of the surfaces represent the local model uncertainty from the Gaussian process (the standard deviation of the modelled i_{pa}/i_{pc}). The fluctuations in the surfaces and the non-convergence of the Gaussian process with additional data points could be ascribed to the fact that a small number of data points are fitted to a large parameter space. The corresponding CV data measured in Stage I and Stage II of the workflow are shown in Supplementary Figs. 24 and 26, respectively.

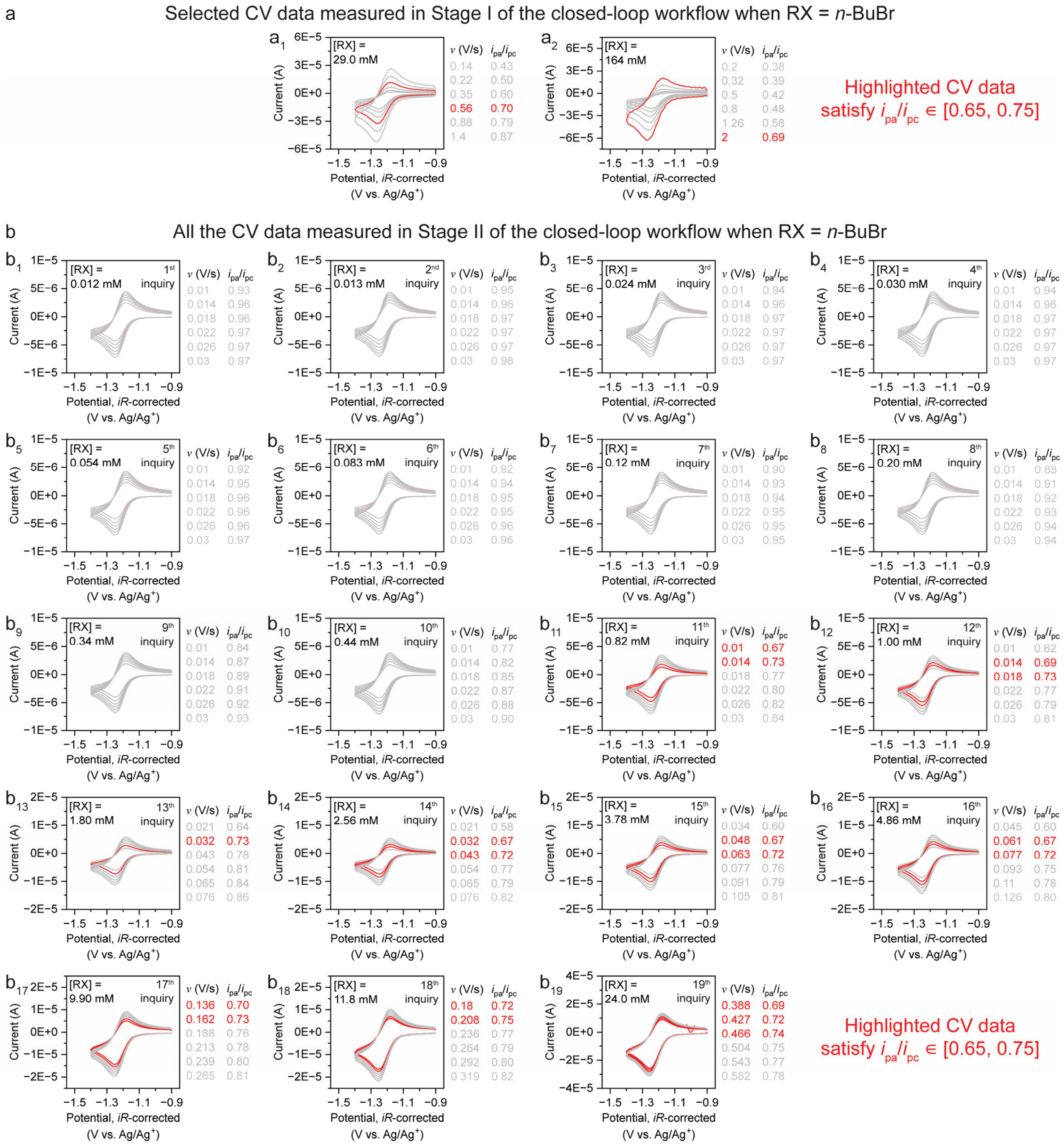

Supplementary Fig. 26. CV data measured in Stage I and Stage II of the closed-loop workflow when RX = *n*-BuBr. Among the 90 voltammograms measured from the 15-step campaign of Bayesian optimization in Stage I (Supplementary Fig. 24), there are only 2 voltammograms that satisfy $i_{pa}/i_{pc} \in [0.65, 0.75]$, as highlighted in red in panel (a). Among the 204 voltammograms measured under the 19 assigned [*n*-BuBr] values in Stage II, suitable v values that satisfy $i_{pa}/i_{pc} \in [0.65, 0.75]$ are found for the later 9 assigned [*n*-BuBr] values, as highlighted in red in panel (b).

Supplementary Note 9. *In silico* analysis of a hypothetical scenario where the response surface of i_{pa}/i_{pc} was not updated on-the-fly during Stage II of the closed-loop workflow when RX = *n*-BuBr.

For Stage II of the closed-loop workflow, we have described how the algorithm designs six proximate *v* values for an experimental inquiry under an assigned [RX] value, which is based on the response surface of i_{pa}/i_{pc} that is created via a Gaussian process (Supplementary Note 8). While inquiring the 19 assigned [RX] values in Stage II, the response surface of i_{pa}/i_{pc} is updated on-the-fly based on the cumulative CV data, every time after an assigned [RX] value is inquired with 6 newly measured voltammograms. In the case when RX = *n*-BuBr (Supplementary Fig. 26b), the first 10 assigned [RX] values are insufficient to trigger a noticeable *C* step even at the lowest *v* value (0.01 V/s) in the parameter space and the i_{pa}/i_{pc} value is always above 0.75, thus only the later 9 assigned [RX] values have the possibility to satisfy $i_{pa}/i_{pc} \in [0.65, 0.75]$ by finding suitable *v* values in the range of [0.01, 2] V/s. Thanks to on-the-fly update of the modelled response surface of i_{pa}/i_{pc}, suitable *v* values that satisfy $i_{pa}/i_{pc} \in [0.65, 0.75]$ are scored for all the later 9 assigned [RX] values (Supplementary Fig. 26b, panels (b_{11})–(b_{19})).

To demonstrate the importance of on-the-fly update of the response surface of i_{pa}/i_{pc}, here we perform *in silico* analysis of a hypothetical scenario where the response surface of i_{pa}/i_{pc} was not updated during Stage II of the workflow when RX = *n*-BuBr, and evaluate the success rate of finding suitable *v* values that satisfy $i_{pa}/i_{pc} \in [0.65, 0.75]$ for the later 9 assigned [RX] values.

Construction of a "ground-truth" response surface of i_{pa}/i_{pc} based on all the cumulative CV data from Stage I and Stage II of the closed-loop workflow when RX = *n*-BuBr. In the parameter space of *v* and $\log_{10}$[*n*-BuBr], 90 voltammograms are measured from a 15-step campaign of Bayesian optimization in Stage I (Supplementary Fig. 24a), and 114 voltammograms are measured from 19 iterative inquiries in Stage II (Supplementary Fig. 26b). By directly connecting the i_{pa}/i_{pc} values of these 204 voltammograms (instead of applying a Gaussian process to fit these i_{pa}/i_{pc} values), a "ground-truth" response surface of i_{pa}/i_{pc} can be constructed based on all the cumulative CV data measured in Stage I and Stage II, as shown below in Supplementary Fig. 27.

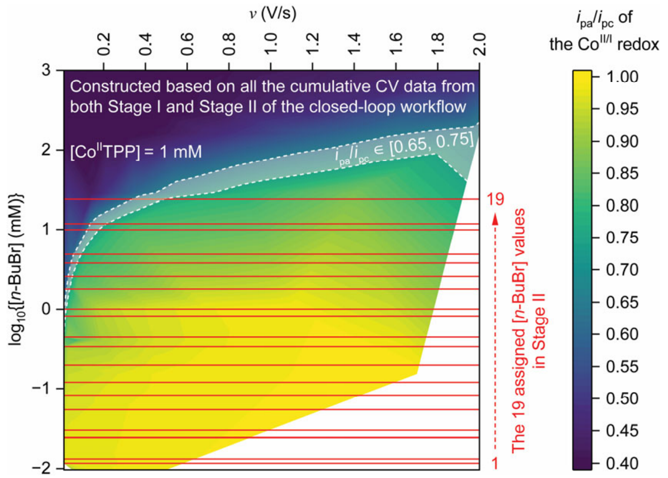

Supplementary Fig. 27. A "ground-truth" response surface of i_{pa}/i_{pc} constructed based on all the cumulative CV data measured in Stage I and Stage II. The colors indicate the i_{pa}/i_{pc} values of the measured voltammograms

in the parameter space of $v \in [0.01, 2]$ V/s and $\log_{10}$[*n*-BuBr] ∈ [$\log_{10}$(0.008 mM), $\log_{10}$(1000 mM)]. The shaded region indicates $i_{pa}/i_{pc} \in [0.65, 0.75]$. The horizontal red lines indicate the 19 assigned [*n*-BuBr] values in Stage II.

From the "ground-truth" response surface of i_{pa}/i_{pc}, the "ground-truth" range of v values to satisfy $i_{pa}/i_{pc} \in [0.65, 0.75]$ under each assigned [*n*-BuBr] value in Stage II can be determined, as summarized below in Supplementary Table 11.

Supplementary Table 11. Determination of the "ground-truth" range of v values to satisfy $i_{pa}/i_{pc} \in [0.65, 0.75]$ under each assigned [*n*-BuBr] value in Stage II.

Assigned [*n*-BuBr] (mM) [1]	"Ground-truth" range of v (V/s) to satisfy i_{pa}/i_{pc} ∈ [0.65, 0.75] [1]	Range of v (V/s) on the response surface of i_{pa}/i_{pc} that satisfy i_{pa}/i_{pc} ∈ [0.65, 0.75] → v_{mid} (V/s) [2] → Designed range of v (V/s) [2] → Score or miss i_{pa}/i_{pc} ∈ [0.65, 0.75]? [3]	
		On-the-fly update of the modelled response surface of i_{pa}/i_{pc}?	
		Yes (the real experimental case)	No (a hypothetical scenario for *in silico* analysis)
$10^{-1.93}$ = 0.012	N/A	N/A → N/A → [0.010, 0.030] → N/A	N/A → N/A → [0.010, 0.030] → N/A
$10^{-1.88}$ = 0.013	N/A	N/A → N/A → [0.010, 0.030] → N/A	N/A → N/A → [0.010, 0.030] → N/A
$10^{-1.61}$ = 0.024	N/A	N/A → N/A → [0.010, 0.030] → N/A	N/A → N/A → [0.010, 0.030] → N/A
$10^{-1.52}$ = 0.030	N/A	N/A → N/A → [0.010, 0.030] → N/A	N/A → N/A → [0.010, 0.030] → N/A
$10^{-1.27}$ = 0.054	N/A	N/A → N/A → [0.010, 0.030] → N/A	N/A → N/A → [0.010, 0.030] → N/A
$10^{-1.08}$ = 0.083	N/A	N/A → N/A → [0.010, 0.030] → N/A	N/A → N/A → [0.010, 0.030] → N/A
$10^{-0.92}$ = 0.12	N/A	N/A → N/A → [0.010, 0.030] → N/A	N/A → N/A → [0.010, 0.030] → N/A
$10^{-0.70}$ = 0.20	N/A	N/A → N/A → [0.010, 0.030] → N/A	N/A → N/A → [0.010, 0.030] → N/A
$10^{-0.47}$ = 0.34	N/A	N/A → N/A → [0.010, 0.030] → N/A	N/A → N/A → [0.010, 0.030] → N/A
$10^{-0.35}$ = 0.44	N/A	N/A → N/A → [0.010, 0.030] → N/A	N/A → N/A → [0.010, 0.030] → N/A
$10^{-0.09}$ = 0.82	[0.010, 0.015]	N/A → N/A → [0.010, 0.030] → Score	N/A → N/A → [0.010, 0.030] → Score
$10^{0.00}$ = 1.00	[0.012, 0.020]	N/A → N/A → [0.010, 0.030] → Score	N/A → N/A → [0.010, 0.030] → Score
$10^{0.26}$ = 1.80	[0.022, 0.037]	[0.01, 0.07] → 0.04 → [0.021, 0.076] → Score	N/A → N/A → [0.010, 0.030] → Score
$10^{0.41}$ = 2.56	[0.031, 0.049]	[0.03, 0.05] → 0.04 → [0.021, 0.076] → Score	N/A → N/A → [0.010, 0.030] → Miss
$10^{0.58}$ = 3.78	[0.044, 0.072]	[0.05, 0.07] → 0.06 → [0.034, 0.105] → Score	N/A → N/A → [0.010, 0.030] → Miss
$10^{0.69}$ = 4.86	[0.057, 0.092]	[0.06, 0.09] → 0.075 → [0.045, 0.126] → Score	[0.01, 0.02] → 0.015 → [0.010, 0.035] → Miss
$10^{1.00}$ = 9.90	[0.122, 0.176]	[0.14, 0.24] → 0.19 → [0.136, 0.265] → Score	[0.04, 0.38] → 0.21 → [0.154, 0.287] → Score
$10^{1.07}$ = 11.8	[0.136, 0.211]	[0.09, 0.39] → 0.24 → [0.180, 0.319] → Score	[0.13, 0.47] → 0.30 → [0.236, 0.382] → Miss
$10^{1.38}$ = 24.0	[0.338, 0.494]	[0.31, 0.66] → 0.485 → [0.388, 0.582] → Score	[0.49, 0.84] → 0.665 → [0.532, 0.798] → Miss

[1] At an assigned [*n*-BuBr] value, the "ground-truth" range of v values to satisfy $i_{pa}/i_{pc} \in [0.65, 0.75]$ is determined from the "ground-truth" response surface of i_{pa}/i_{pc}, which is constructed based on all the cumulative CV data from Stage I and Stage II of the closed-loop workflow when RX = *n*-BuBr (see Supplementary Fig. 27 on the last page).

[2] v_{mid} is the midpoint of the range of v values on the response surface of i_{pa}/i_{pc} that satisfy $i_{pa}/i_{pc} \in [0.65, 0.75]$, and the designed range of v values is defined as $[v_{design,min}, v_{design,max}] = ([0.8\times v_{mid}, 1.2\times v_{mid}] \cup [10^{0.8\times\log_{10}(v_{mid})}, 10^{1.2\times\log_{10}(v_{mid})}]) \cap [0.01, 2]$ V/s. If there does not exist such a v_{mid} on the response surface (denoted as N/A), the designed range of v is set as [0.010, 0.030] V/s (see Supplementary Note 8).

[3] At an assigned [*n*-BuBr] value, if there is a non-empty intersection between the designed range of v values and the "ground-truth" range of v values to satisfy $i_{pa}/i_{pc} \in [0.65, 0.75]$, $i_{pa}/i_{pc} \in [0.65, 0.75]$ will be scored. Otherwise, $i_{pa}/i_{pc} \in [0.65, 0.75]$ will be missed.

The real experimental case where the response surface of i_{pa}/i_{pc} is updated on-the-fly during Stage II of the closed-loop workflow when RX = *n*-BuBr. On-the-fly update of the response surface of i_{pa}/i_{pc} is visualized in Supplementary Fig. 25. The response surface of i_{pa}/i_{pc} updated after inquiring an assigned [*n*-BuBr] value is used to design a narrow range of *v* values for the experimental inquiry under the next assigned [*n*-BuBr] value. As a result, for all the later 9 assigned values of [*n*-BuBr] where the "ground-truth" range of *v* values to satisfy $i_{pa}/i_{pc} \in [0.65, 0.75]$ exists, the designed range of *v* values can always score $i_{pa}/i_{pc} \in [0.65, 0.75]$ (see Supplementary Table 11 on the last page), consistent with the experimental results obtained from Stage II of the closed-loop workflow when RX = *n*-BuBr (Supplementary Fig. 26b, panels (b_{11})–(b_{19})).

A hypothetical scenario for *in silico* analysis where the response surface of i_{pa}/i_{pc} was not updated on-the-fly during Stage II of the closed-loop workflow when RX = *n*-BuBr. In this hypothetical scenario, once the initial response surface of i_{pa}/i_{pc} was created from 90 voltammograms right after Stage I, it was not updated based on the cumulative CV data. That is, the initial response surface of i_{pa}/i_{pc} was used to design a narrow range of *v* values for experimentally inquiring all the 19 assigned [*n*-BuBr] values in Stage II. From *in silico* analysis, for the later 9 assigned [*n*-BuBr] values where the "ground-truth" range of *v* values to satisfy $i_{pa}/i_{pc} \in [0.65, 0.75]$ exists, there is a near 50% reduction in the success rate of scoring $i_{pa}/i_{pc} \in [0.65, 0.75]$ in the designed range of *v* values, which only works for 4 out of these 9 assigned [*n*-BuBr] values in Stage II (see Supplementary Table 11 on the last page).

Therefore, *in silico* analysis demonstrates the importance of updating the response surface of i_{pa}/i_{pc} on-the-fly based on the cumulative CV data during Stage II of the closed-loop workflow, as it keeps creating a more accurate response surface of i_{pa}/i_{pc} for better designing a narrow range of *v* values for experimentally inquiring the next assigned [RX] value, which is beneficial for acquiring as many usable data for electrokinetic analysis as possible.

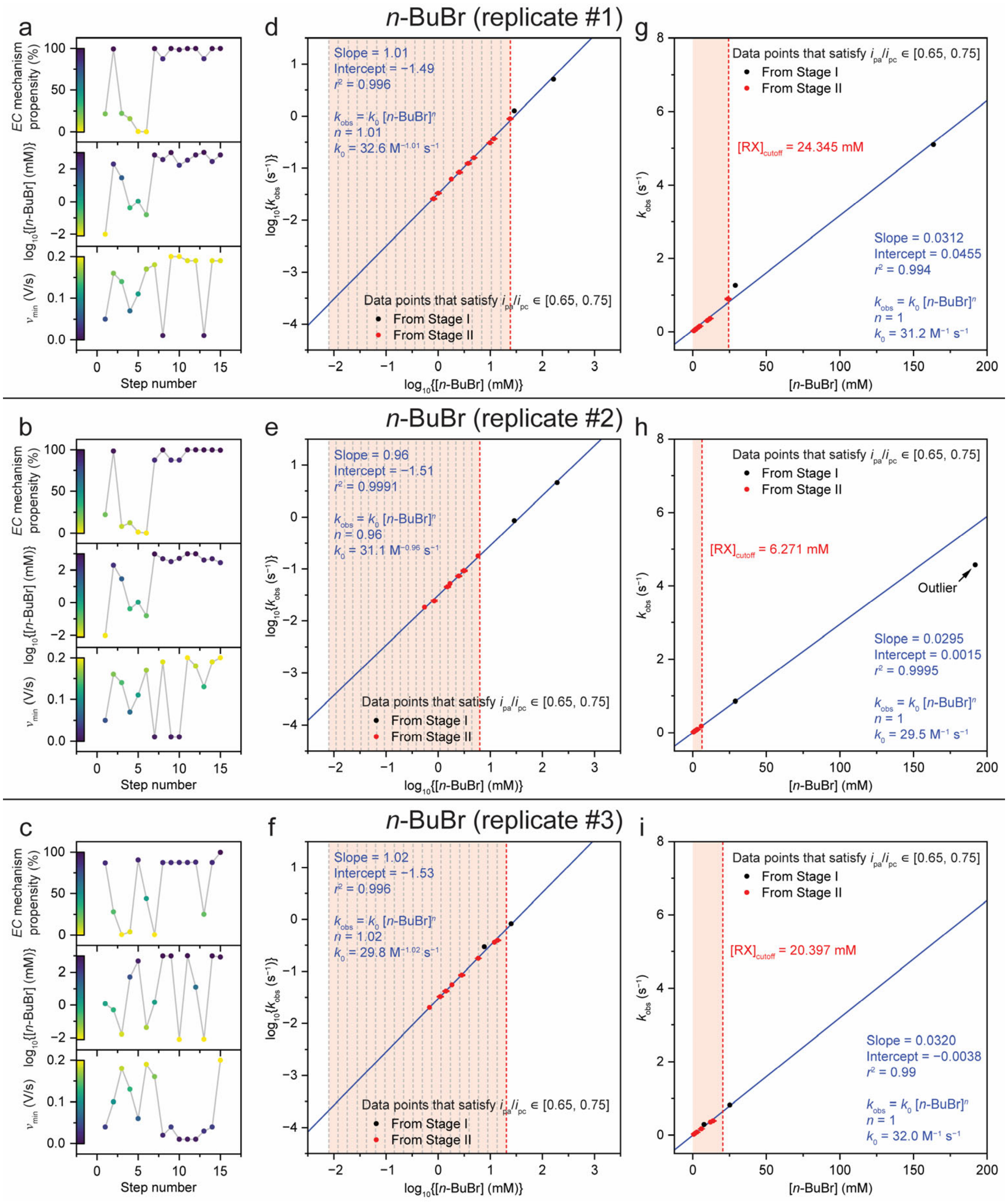

Supplementary Fig. 28. Three replicates of autonomous investigation of *n*-BuBr by the closed-loop workflow. (a–c) Bayesian optimization results in Stage I. (d–f) The $\log_{10}(k_{obs})$ versus $\log_{10}$[*n*-BuBr] plots and (g–i) the k_{obs} versus [*n*-BuBr] plots based on all the valid k_{obs} values derived from $i_{pa}/i_{pc} \in [0.65, 0.75]$ in Stage I (black dots) and Stage II (red dots). In panels (d–i), the dashed red lines indicate $[RX]_{cutoff}$ values (Supplementary Note 7), the dashed grey lines indicate 19 equally divided intervals between $[RX]_{min}$ and $[RX]_{cutoff}$ values on the logarithmic scale (Supplementary Note 8), and the vertical error bars represent the standard deviations of all the valid $\log_{10}(k_{obs})$ or k_{obs} values at each [RX] value. Cross-checking the logarithmic scale plot and the linear scale plot helps identify outliers in the kinetic data. The k_0 values presented in Fig. 4a in the main text are based on linear regressions of the linear scale plots, where the reaction order (n) of [RX] is strictly 1 and the unit of k_0 is strictly $M^{-1}\ s^{-1}$.

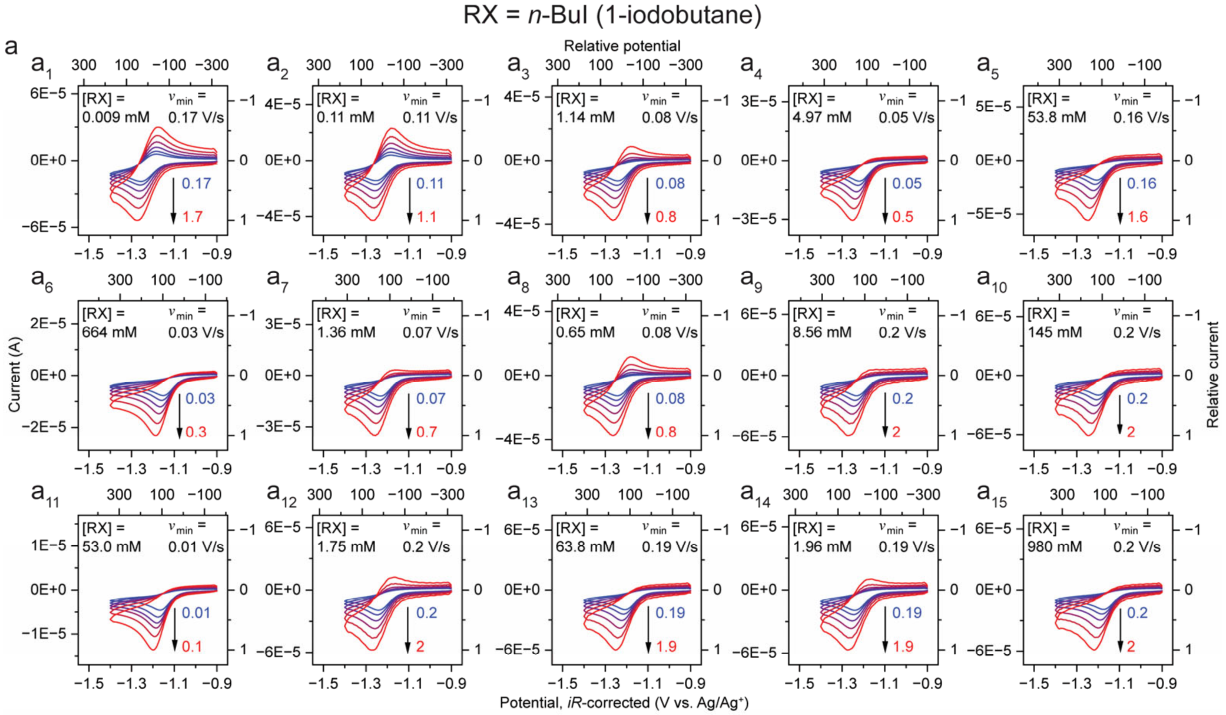

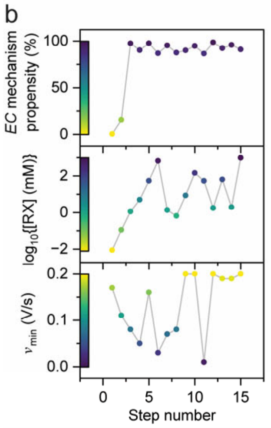

c

Potential range: between −0.9 and −1.4 V vs. Ag/Ag+							
Step	[RX] (mM)	v_{min} (V/s)	Mechanism propensity (%)				
			E	*EC*	*CE*	*DISP1*	*ECE*
1	0.009	0.17	99.6	0.4	0.0	0.0	0.0
2	0.11	0.11	84.5	15.5	0.0	0.0	0.0
3	1.14	0.08	2.4	97.6	0.0	0.0	0.0
4	4.97	0.05	9.6	90.4	0.0	0.0	0.0
5	53.8	0.16	0.4	97.7	0.0	0.0	1.9
6	664	0.03	13.1	86.9	0.0	0.0	0.0
7	1.36	0.07	4.6	95.4	0.0	0.0	0.0
8	0.65	0.08	12.3	87.7	0.0	0.0	0.0
9	8.56	0.2	0.2	90.1	0.0	0.0	9.7
10	145	0.2	0.8	94.8	0.0	0.0	4.4
11	53.0	0.01	13.4	86.6	0.0	0.0	0.0
12	1.75	0.2	0.3	98.7	0.0	0.0	1.0
13	63.8	0.19	0.4	92.4	0.0	0.0	7.1
14	1.96	0.19	0.3	96.0	0.0	0.0	3.8
15	980	0.2	2.4	91.1	0.0	0.0	6.4

d

$[RX]_{cutoff}$ = 2.589 mM		
CV data that satisfy $i_{pa}/i_{pc} \in [0.65, 0.75]$ from Stage I of the workflow		
[RX] (mM)	$\log_{10}$\{[RX] (mM)\}	v (V/s) that satisfy $i_{pa}/i_{pc} \in [0.65, 0.75]$
0.009	−2.059	0.17, 0.27, 0.43, 0.68, 1.07, 1.7
0.11	−0.954	0.11, 0.17, 0.28, 0.44, 0.69, 1.1
0.65	−0.186	0.08, 0.13, 0.2, 0.32, 0.5, 0.8
CV data that satisfy $i_{pa}/i_{pc} \in [0.65, 0.75]$ from Stage II of the workflow		
[RX] (mM)	$\log_{10}$\{[RX] (mM)\}	v (V/s) that satisfy $i_{pa}/i_{pc} \in [0.65, 0.75]$
0.015	−1.816	0.01, 0.014, 0.018, 0.022, 0.026, 0.03
0.18	−0.742	0.132, 0.157, 0.183, 0.208, 0.234, 0.259
0.48	−0.319	0.706, 0.776, 0.847, 0.917, 0.988, 1.06
0.89	−0.052	1.02, 1.12, 1.22, 1.32, 1.43, 1.53
1.38	0.141	1.57, 1.66, 1.74, 1.83, 1.91, 2

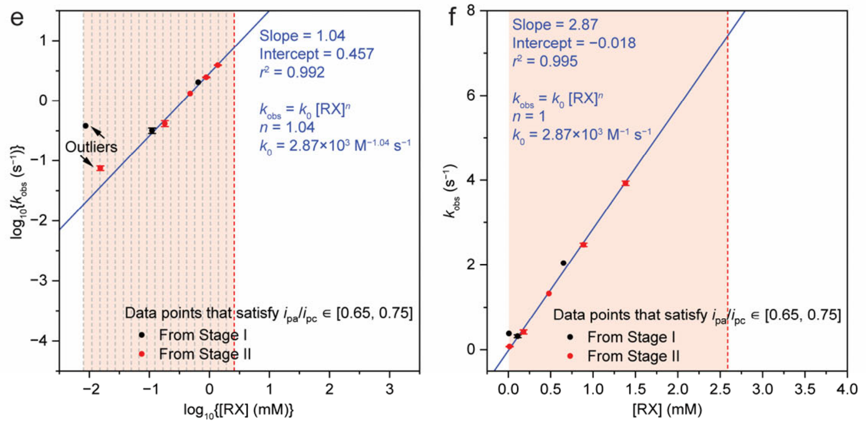

Supplementary Fig. 29. Autonomous investigation of *n*-BuI (1-iodobutane) that reacts with CoTPP following an *EC* mechanism. (a) CV data (1 mM Co^{II}TPP in DMF with 0.1 M NBu_4PF_6, [RX] ∈ [0.008, 1000] mM, v_{min} ∈ [0.01, 0.2] V/s) measured from Stage I, with detailed parameters and Bayesian optimization results summarized in (b) and (c). (d) Desired combinations of [RX] and v that satisfy $i_{pa}/i_{pc} \in [0.65, 0.75]$ from Stage I and Stage II. (e) The $\log_{10}(k_{obs})$ versus $\log_{10}$[RX] plot and (f) the k_{obs} versus [RX] plot based on all the valid k_{obs} values derived from $i_{pa}/i_{pc} \in [0.65, 0.75]$ in Stage I (black dots) and Stage II (red dots).

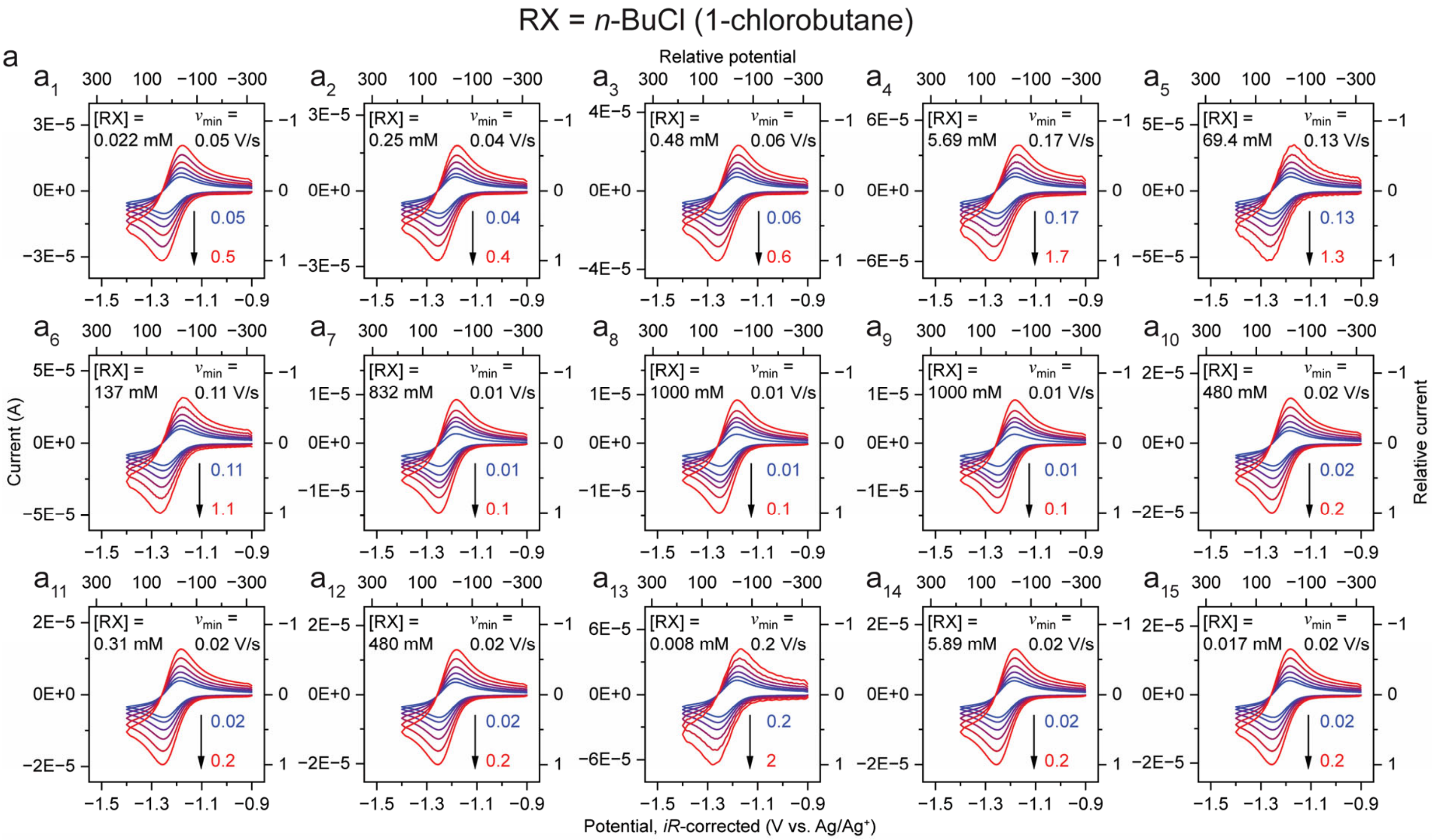

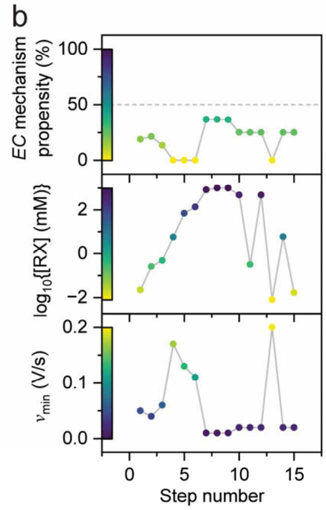

c

Potential range: between −0.9 and −1.4 V vs. Ag/Ag+							
Step	[RX] (mM)	v_{min} (V/s)	Mechanism propensity (%)				
			E	*EC*	*CE*	*DISP1*	*ECE*
1	0.022	0.05	81.2	18.8	0.0	0.0	0.0
2	0.25	0.04	78.6	21.4	0.0	0.0	0.0
3	0.48	0.06	86.6	13.4	0.0	0.0	0.0
4	5.69	0.17	100.0	0.0	0.0	0.0	0.0
5	69.4	0.13	100.0	0.0	0.0	0.0	0.0
6	137	0.11	100.0	0.0	0.0	0.0	0.0
7	832	0.01	63.3	36.7	0.0	0.0	0.0
8	1000	0.01	63.5	36.5	0.0	0.0	0.0
9	1000	0.02	63.3	36.7	0.0	0.0	0.0
10	480	0.02	74.9	25.1	0.0	0.0	0.0
11	0.31	0.02	75.0	25.0	0.0	0.0	0.0
12	480	0.02	75.0	25.0	0.0	0.0	0.0
13	0.008	0.2	100.0	0.0	0.0	0.0	0.0
14	5.89	0.02	75.0	25.0	0.0	0.0	0.0
15	0.017	0.02	75.0	25.0	0.0	0.0	0.0

Supplementary Fig. 30. Autonomous investigation of *n*-BuCl (1-chlorobutane) with an undetected reactivity toward CoTPP in the parameter space. (a) CV data (1 mM Co^{II}TPP in DMF with 0.1 M NBu_4PF_6, [RX] ∈ [0.008, 1000] mM, v_{min} ∈ [0.01, 0.2] V/s) measured from Stage I, with detailed parameters and Bayesian optimization results summarized in (b) and (c). As the DL-generated propensity of *EC* mechanism did not exceed the 50% threshold, the closed-loop workflow autonomously ended after Stage I and did not proceed to Stage II (see the flowchart in Supplementary Fig. 16).

RX = n-HexBr (1-bromohexane)

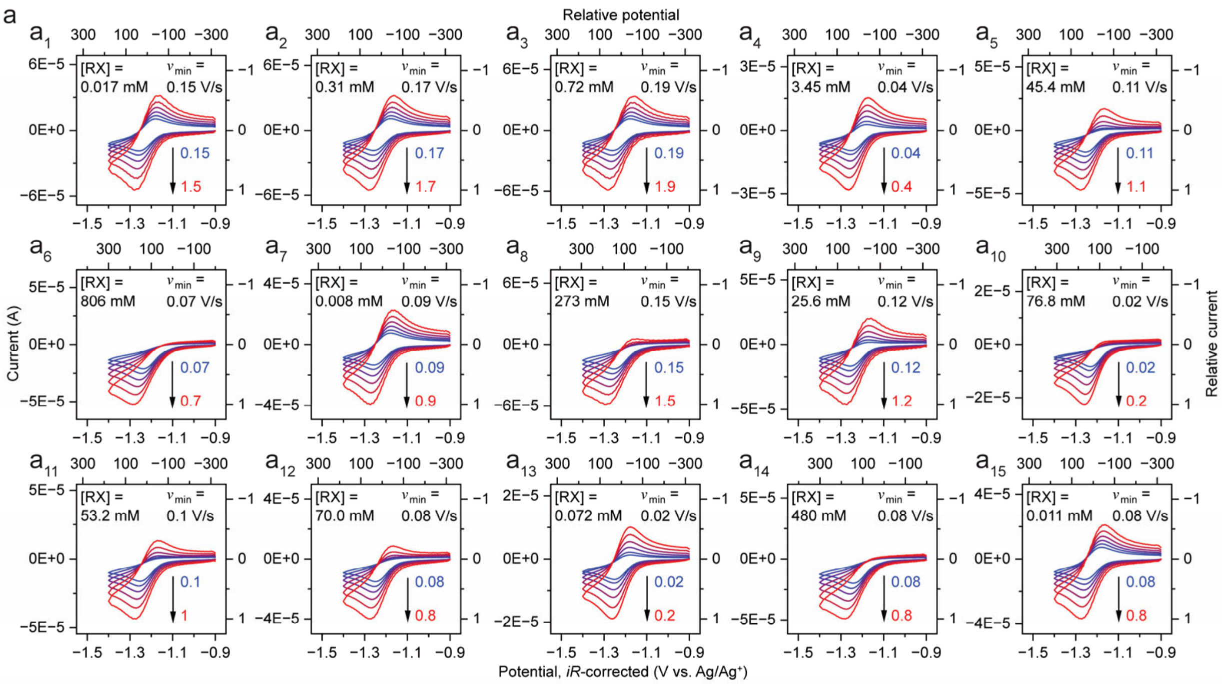

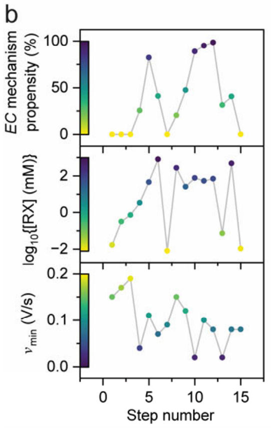

c

Step	[RX] (mM)	v_{min} (V/s)	Mechanism propensity (%)				
			E	*EC*	*CE*	*DISP1*	*ECE*
1	0.017	0.15	100.0	0.0	0.0	0.0	0.0
2	0.31	0.17	100.0	0.0	0.0	0.0	0.0
3	0.72	0.19	100.0	0.0	0.0	0.0	0.0
4	3.45	0.04	74.5	25.5	0.0	0.0	0.0
5	45.4	0.11	17.7	82.3	0.0	0.0	0.0
6	806	0.07	0.1	41.2	0.0	0.0	58.7
7	0.008	0.09	100.0	0.0	0.0	0.0	0.0
8	273	0.15	0.0	20.3	0.0	0.0	79.7
9	25.6	0.12	52.4	49.6	0.0	0.0	0.0
10	76.8	0.02	11.0	89.0	0.0	0.0	0.0
11	53.2	0.1	5.0	95.0	0.0	0.0	0.0
12	70.0	0.08	1.1	98.6	0.0	0.0	0.3
13	0.072	0.02	68.7	31.3	0.0	0.0	0.0
14	480	0.08	0.0	40.8	0.0	0.0	59.2
15	0.011	0.08	99.9	0.1	0.0	0.0	0.0

Potential range: between −0.9 and −1.4 V vs. Ag/Ag⁺

d

$[RX]_{cutoff}$ = 7.484 mM

CV data that satisfy i_{pa}/i_{pc} ∈ [0.65, 0.75] from Stage I of the workflow		
[RX] (mM)	log10{[RX] (mM)}	v (V/s) that satisfy i_{pa}/i_{pc} ∈ [0.65, 0.75]
3.45	0.538	0.04, 0.06, 0.1, 0.16, 0.25, 0.4
45.4	1.657	0.11, 0.17, 0.28, 0.44, 0.69, 1.1
25.6	1.408	0.12, 0.19, 0.3, 0.48, 0.76, 1.2
53.2	1.726	0.1, 0.16, 0.25, 0.4, 0.63, 1
0.072	−1.14	0.02, 0.03, 0.05, 0.08, 0.13, 0.2
**CV data that satisfy i_{pa}/i_{pc} ∈ [0.65, 0.75] from Stage II of the workflow**		
[RX] (mM)	log10{[RX] (mM)}	v (V/s) that satisfy i_{pa}/i_{pc} ∈ [0.65, 0.75]
0.82	−0.084	0.01, 0.014, 0.018, 0.022, 0.026, 0.03
0.96	−0.018	0.01, 0.014, 0.018, 0.022, 0.026, 0.03
1.56	0.192	0.01, 0.014, 0.018, 0.022, 0.026, 0.03
2.02	0.305	0.021, 0.032, 0.043, 0.054, 0.065, 0.076
3.86	0.587	0.018, 0.028, 0.038, 0.048, 0.058, 0.068

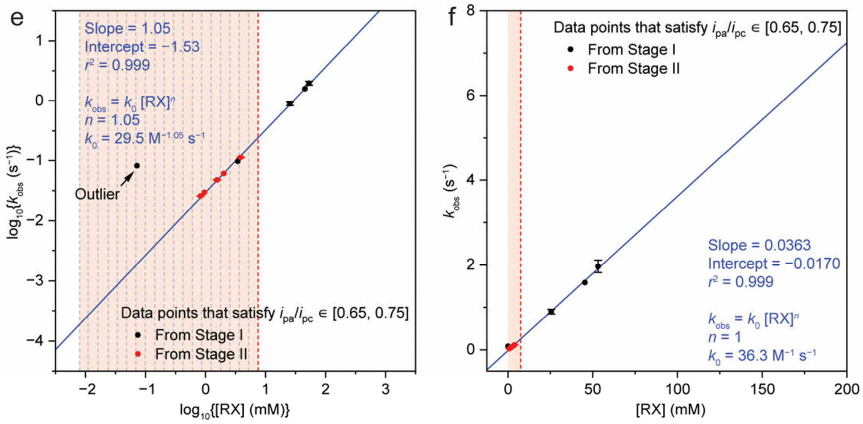

Supplementary Fig. 31. Autonomous investigation of *n*-HexBr (1-bromohexane) that reacts with CoTPP following an *EC* mechanism. (a) CV data (1 mM $Co^{II}TPP$ in DMF with 0.1 M NBu_4PF_6, [RX] ∈ [0.008, 1000] mM, v_{min} ∈ [0.01, 0.2] V/s) measured from Stage I, with detailed parameters and Bayesian optimization results summarized in (b) and (c). (d) Desired combinations of [RX] and v that satisfy i_{pa}/i_{pc} ∈ [0.65, 0.75] from Stage I and Stage II. (e) The $\log_{10}(k_{obs})$ versus $\log_{10}$[RX] plot and (f) the k_{obs} versus [RX] plot based on all the valid k_{obs} values derived from i_{pa}/i_{pc} ∈ [0.65, 0.75] in Stage I (black dots) and Stage II (red dots).

RX = *n*-OctBr (1-bromooctane)

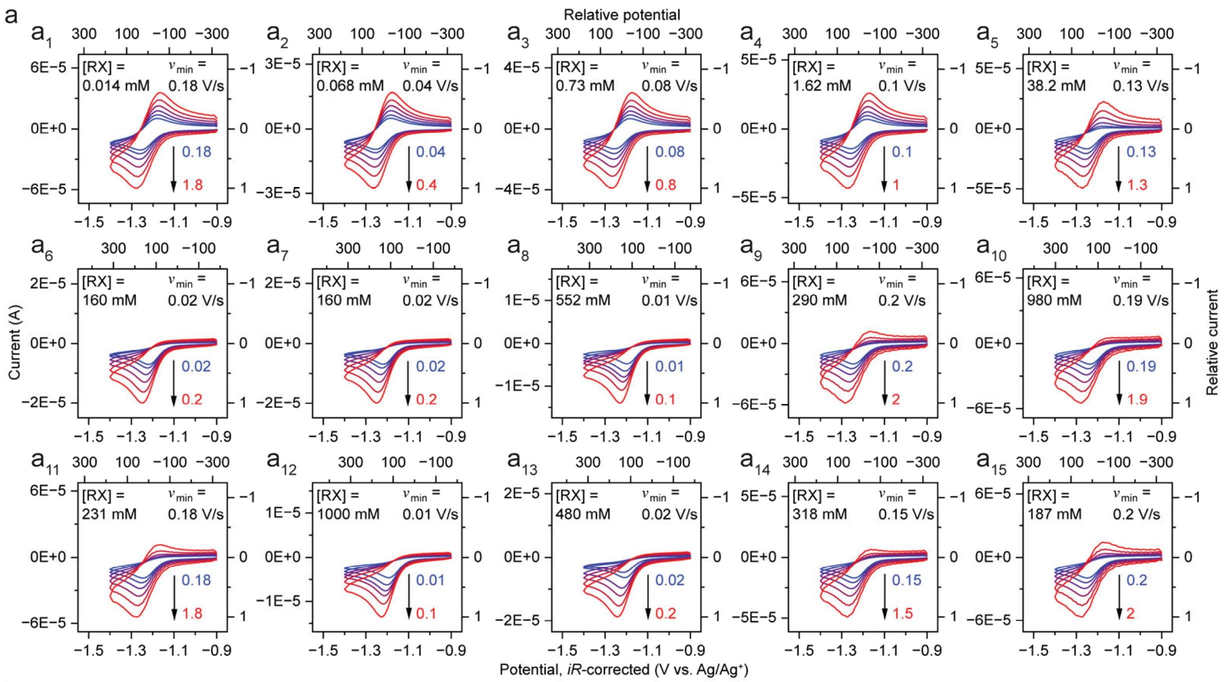

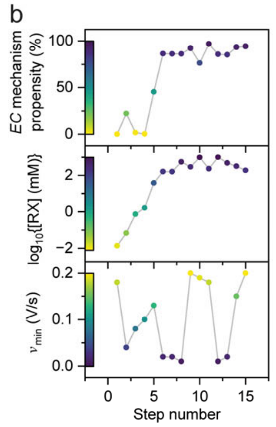

c

Step	[RX] (mM)	v_{min} (V/s)	Mechanism propensity (%)				
			E	*EC*	*CE*	*DISP1*	*ECE*
1	0.014	0.18	100.0	0.0	0.0	0.0	0.0
2	0.068	0.04	77.7	22.3	0.0	0.0	0.0
3	0.73	0.08	98.3	1.7	0.0	0.0	0.0
4	1.62	0.1	99.8	0.2	0.0	0.0	0.0
5	38.2	0.13	54.8	45.2	0.0	0.0	0.0
6	160	0.02	13.4	86.6	0.0	0.0	0.0
7	160	0.02	13.6	86.4	0.0	0.0	0.0
8	552	0.01	13.6	86.4	0.0	0.0	0.0
9	290	0.2	0.2	92.4	0.0	0.0	7.4
10	980	0.19	0.1	76.4	0.0	0.0	23.5
11	231	0.18	0.3	96.9	0.0	0.0	2.8
12	1000	0.01	14.0	86.0	0.0	0.0	0.0
13	480	0.02	14.6	85.4	0.0	0.0	0.0
14	318	0.15	0.3	93.5	0.0	0.0	6.2
15	187	0.2	0.4	94.4	0.0	0.0	5.2

Potential range: between −0.9 and −1.4 V vs. Ag/Ag⁺

d

$[RX]_{cutoff}$ = 6.652 mM

[RX] (mM)	$\log_{10}$\{[RX] (mM)\}	*v* (V/s) that satisfy i_{pa}/i_{pc} ∈ [0.65, 0.75]
CV data that satisfy i_{pa}/i_{pc} ∈ [0.65, 0.75] from Stage I of the workflow		
38.2	1.582	0.13, 0.21, 0.33, 0.52, 0.82, 1.3
187	2.272	0.2, 0.32, 0.5, 0.8, 1.26, 2
CV data that satisfy i_{pa}/i_{pc} ∈ [0.65, 0.75] from Stage II of the workflow		
[RX] (mM)	$\log_{10}$\{[RX] (mM)\}	*v* (V/s) that satisfy i_{pa}/i_{pc} ∈ [0.65, 0.75]
0.008	−2.088	0.01, 0.014, 0.018, 0.022, 0.026, 0.03
0.70	−0.152	0.01, 0.014, 0.018, 0.022, 0.026, 0.03
0.98	−0.009	0.01, 0.014, 0.018, 0.022, 0.026, 0.03
1.16	0.064	0.01, 0.014, 0.018, 0.022, 0.026, 0.03
1.80	0.254	0.021, 0.032, 0.043, 0.054, 0.065, 0.076
2.98	0.474	0.024, 0.036, 0.048, 0.06, 0.072, 0.084
3.34	0.524	0.031, 0.044, 0.058, 0.071, 0.085, 0.098
5.80	0.763	0.082, 0.104, 0.125, 0.147, 0.168, 0.189

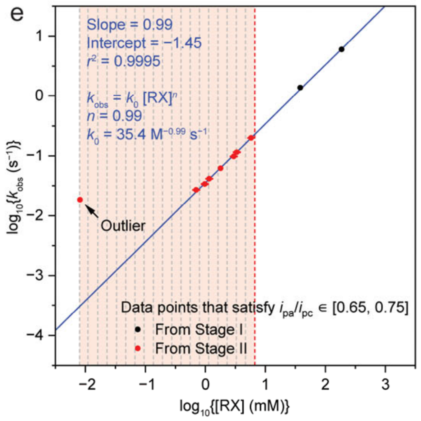

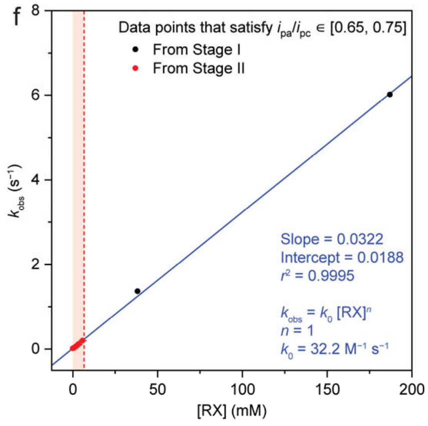

Supplementary Fig. 32. Autonomous investigation of *n*-OctBr (1-bromooctane) that reacts with CoTPP following an *EC* mechanism. (a) CV data (1 mM $Co^{II}TPP$ in DMF with 0.1 M NBu_4PF_6, [RX] ∈ [0.008, 1000] mM, v_{min} ∈ [0.01, 0.2] V/s) measured from Stage I, with detailed parameters and Bayesian optimization results summarized in (b) and (c). (d) Desired combinations of [RX] and v that satisfy i_{pa}/i_{pc} ∈ [0.65, 0.75] from Stage I and Stage II. (e) The $\log_{10}(k_{obs})$ versus $\log_{10}$[RX] plot and (f) the k_{obs} versus [RX] plot based on all the valid k_{obs} values derived from i_{pa}/i_{pc} ∈ [0.65, 0.75] in Stage I (black dots) and Stage II (red dots).

RX = *i*-BuBr (1-bromo-2-methylpropane)

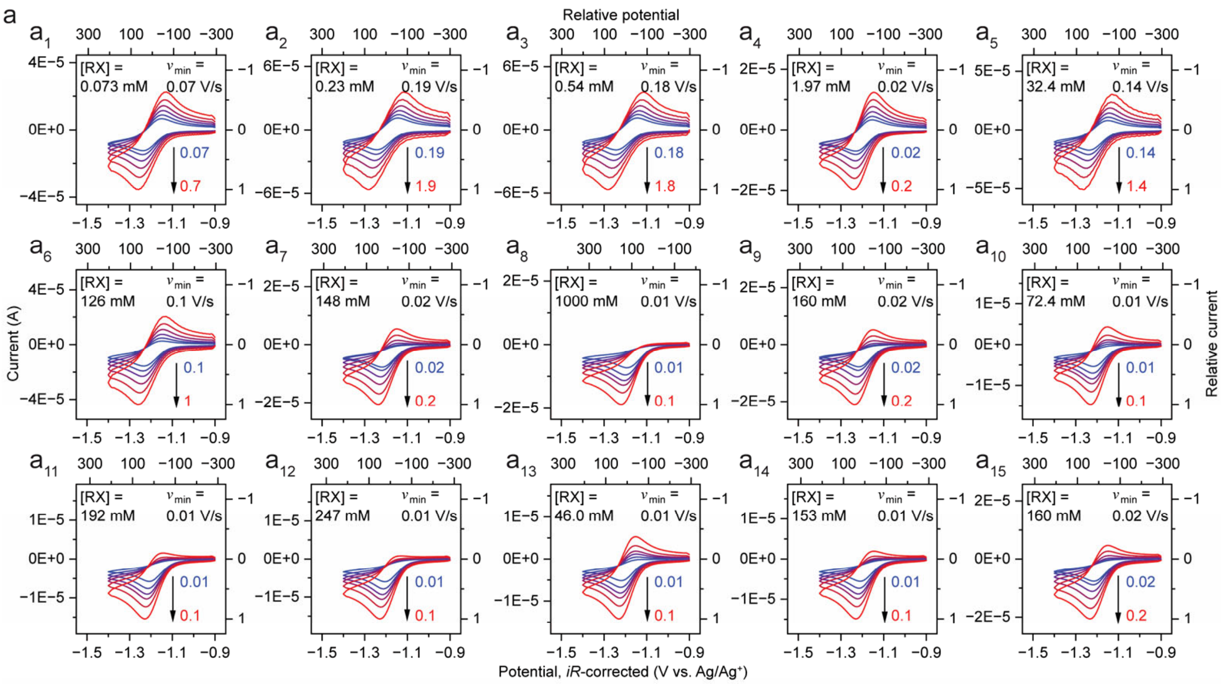

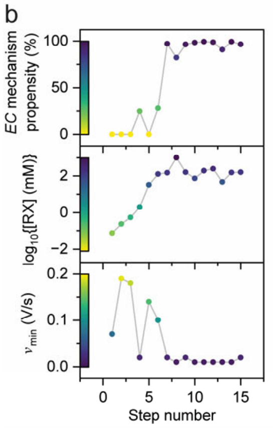

c

Potential range: between −0.9 and −1.4 V vs. Ag/Ag+							
Step	[RX] (mM)	v_{min} (V/s)	Mechanism propensity (%)				
			E	*EC*	*CE*	*DISP1*	*ECE*
1	0.073	0.07	100.0	0.0	0.0	0.0	0.0
2	0.23	0.19	100.0	0.0	0.0	0.0	0.0
3	0.54	0.18	100.0	0.0	0.0	0.0	0.0
4	1.97	0.02	75.3	24.7	0.0	0.0	0.0
5	32.4	0.14	100.0	0.0	0.0	0.0	0.0
6	126	0.1	72.0	28.0	0.0	0.0	0.0
7	148	0.02	2.8	97.2	0.0	0.0	0.0
8	1000	0.01	0.0	82.2	0.0	0.0	17.7
9	160	0.02	3.5	96.5	0.0	0.0	0.0
10	72.4	0.01	1.7	98.3	0.0	0.0	0.0
11	192	0.01	0.3	99.3	0.0	0.0	0.3
12	247	0.01	0.2	98.8	0.0	0.0	1.1
13	46.0	0.01	9.1	90.9	0.0	0.0	0.0
14	153	0.01	0.7	99.3	0.0	0.0	0.0
15	160	0.02	3.4	96.6	0.0	0.0	0.0

d

$[RX]_{cutoff}$ = 4.953 mM		
CV data that satisfy i_{pa}/i_{pc} ∈ [0.65, 0.75] from Stage I of the workflow		
[RX] (mM)	$\log_{10}${[RX] (mM)}	*v* (V/s) that satisfy i_{pa}/i_{pc} ∈ [0.65, 0.75]
126	2.10	0.1, 0.16, 0.25, 0.4, 0.63, 1
72.4	1.86	0.01, 0.02, 0.03, 0.04, 0.06, 0.1
46.0	1.66	0.01, 0.02, 0.03, 0.04, 0.06, 0.1
CV data that satisfy i_{pa}/i_{pc} ∈ [0.65, 0.75] from Stage II of the workflow		
[RX] (mM)	$\log_{10}${[RX] (mM)}	*v* (V/s) that satisfy i_{pa}/i_{pc} ∈ [0.65, 0.75]
None	None	None

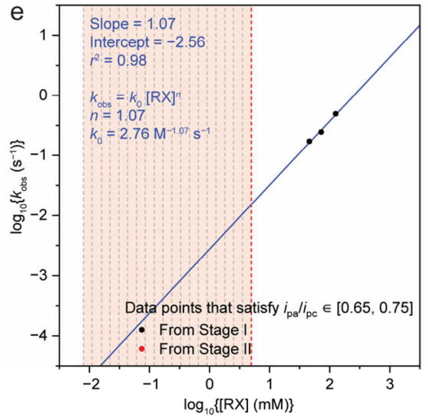

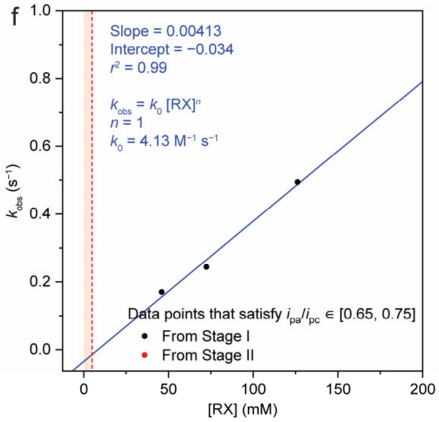

Supplementary Fig. 33. Autonomous investigation of *i*-BuBr (1-bromo-2-methylpropane) that reacts with CoTPP following an *EC* mechanism. (a) CV data (1 mM Co^{II}TPP in DMF with 0.1 M NBu_4PF_6, [RX] ∈ [0.008, 1000] mM, v_{min} ∈ [0.01, 0.2] V/s) measured from Stage I, with detailed parameters and Bayesian optimization results summarized in (b) and (c). (d) Desired combinations of [RX] and v that satisfy i_{pa}/i_{pc} ∈ [0.65, 0.75] from Stage I and Stage II. (e) The $\log_{10}(k_{obs})$ versus $\log_{10}$[RX] plot and (f) the k_{obs} versus [RX] plot based on all the valid k_{obs} values derived from i_{pa}/i_{pc} ∈ [0.65, 0.75] in Stage I (black dots) and Stage II (red dots).

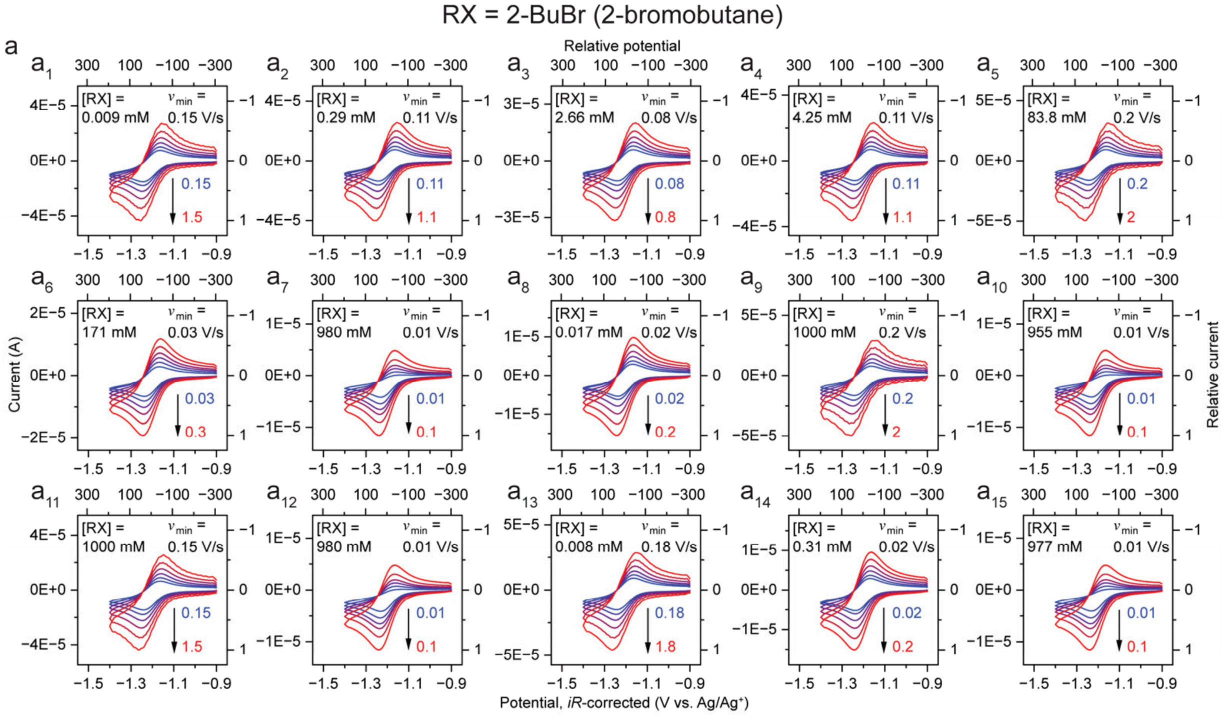

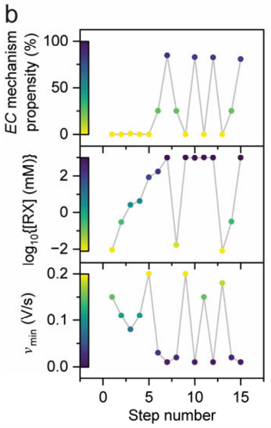

c

Step	[RX] (mM)	v_{min} (V/s)	Mechanism propensity (%)				
			E	*EC*	*CE*	*DISP1*	*ECE*
1	0.009	0.15	100.0	0.0	0.0	0.0	0.0
2	0.29	0.11	100.0	0.0	0.0	0.0	0.0
3	2.66	0.08	99.3	0.7	0.0	0.0	0.0
4	4.25	0.11	100.0	0.0	0.0	0.0	0.0
5	83.8	0.2	100.0	0.0	0.0	0.0	0.0
6	171	0.03	74.9	25.1	0.0	0.0	0.0
7	980	0.01	15.5	84.5	0.0	0.0	0.0
8	0.017	0.02	75.0	25.0	0.0	0.0	0.0
9	1000	0.2	100.0	0.0	0.0	0.0	0.0
10	955	0.01	17.3	82.7	0.0	0.0	0.0
11	1000	0.15	100.0	0.0	0.0	0.0	0.0
12	980	0.01	17.7	82.3	0.0	0.0	0.0
13	0.008	0.18	100.0	0.0	0.0	0.0	0.0
14	0.31	0.02	75.0	25.0	0.0	0.0	0.0
15	977	0.01	19.4	80.6	0.0	0.0	0.0

Potential range: between −0.9 and −1.4 V vs. Ag/Ag+

d

$[RX]_{cutoff}$ = 522.711 mM

[RX] (mM)	$\log_{10}$([RX] (mM))	v (V/s) that satisfy $i_{pa}/i_{pc} \in$ [0.65, 0.75]
**CV data that satisfy $i_{pa}/i_{pc} \in$ [0.65, 0.75] from Stage I of the workflow**		
980	2.99	0.01, 0.02, 0.03, 0.04, 0.06, 0.1
955	2.98	0.01, 0.02, 0.03, 0.04, 0.06, 0.1
980	2.99	0.01, 0.02, 0.03, 0.04, 0.06, 0.1
977	2.99	0.01, 0.02, 0.03, 0.04, 0.06, 0.1
**CV data that satisfy $i_{pa}/i_{pc} \in$ [0.65, 0.75] from Stage II of the workflow**		
157	2.20	0.01, 0.013, 0.016, 0.019, 0.022, 0.025
266	2.42	0.01, 0.015, 0.02, 0.025, 0.03, 0.035
382	2.58	0.012, 0.02, 0.028, 0.036, 0.044, 0.052

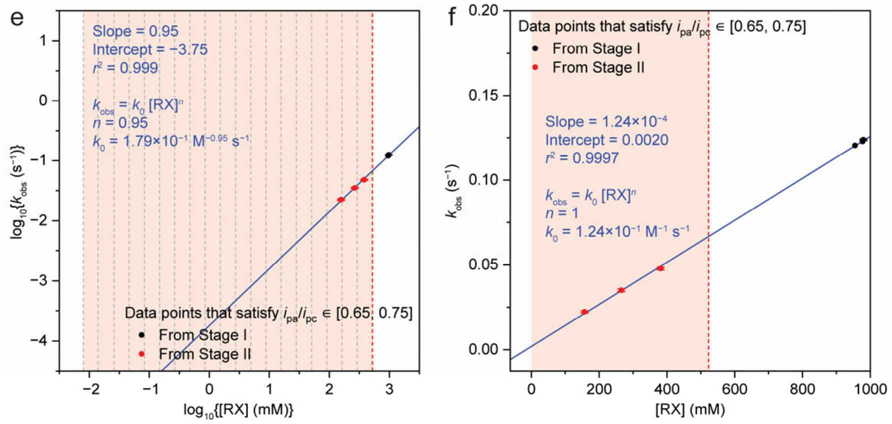

Supplementary Fig. 34. Autonomous investigation of 2-BuBr (2-bromobutane) that reacts with CoTPP following an *EC* mechanism. (a) CV data (1 mM Co^{II}TPP in DMF with 0.1 M NBu_4PF_6, [RX] ∈ [0.008, 1000] mM, v_{min} ∈ [0.01, 0.2] V/s) measured from Stage I, with detailed parameters and Bayesian optimization results summarized in (b) and (c). (d) Desired combinations of [RX] and v that satisfy i_{pa}/i_{pc} ∈ [0.65, 0.75] from Stage I and Stage II. (e) The $\log_{10}(k_{obs})$ versus $\log_{10}$[RX] plot and (f) the k_{obs} versus [RX] plot based on all the valid k_{obs} values derived from i_{pa}/i_{pc} ∈ [0.65, 0.75] in Stage I (black dots) and Stage II (red dots).

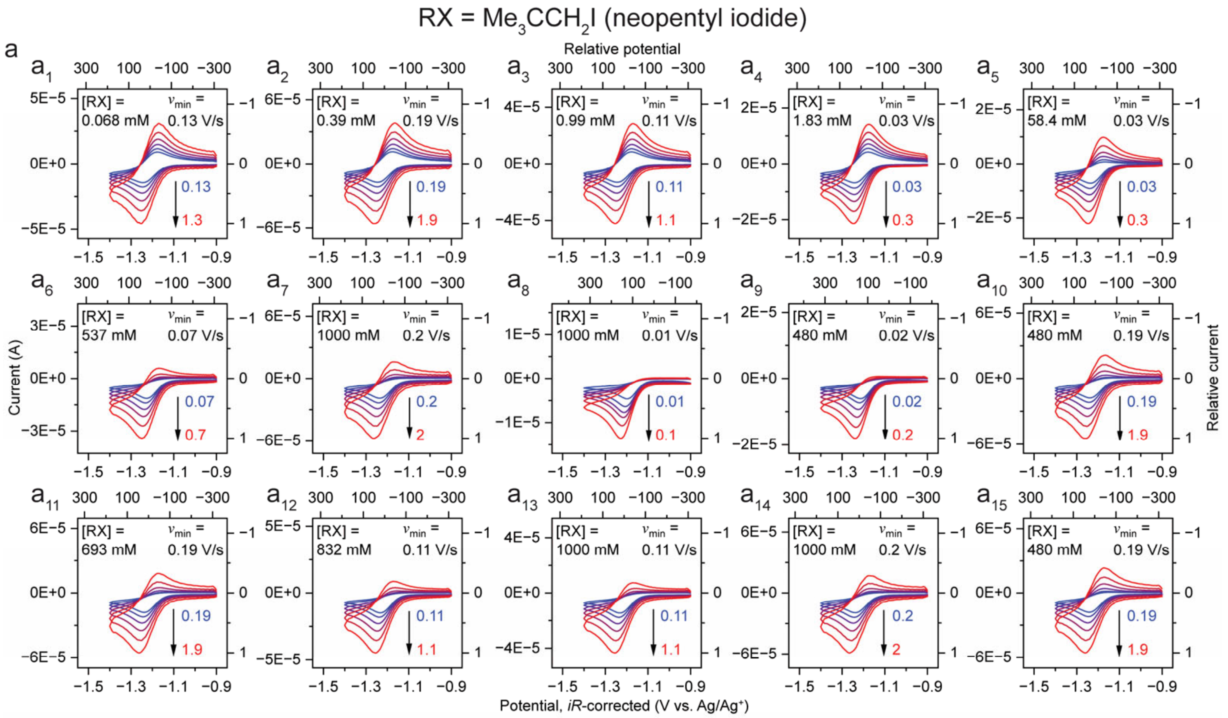

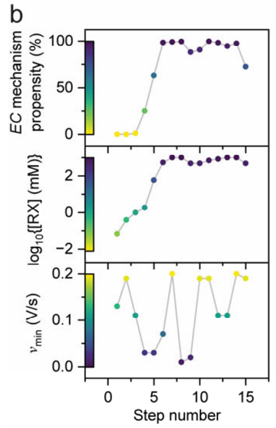

c

Potential range: between −0.9 and −1.4 V vs. Ag/Ag⁺							
Step	[RX] (mM)	v_{min} (V/s)	Mechanism propensity (%)				
			E	*EC*	*CE*	*DISP1*	*ECE*
1	0.068	0.13	99.9	0.1	0.0	0.0	0.0
2	0.39	0.19	100.0	0.0	0.0	0.0	0.0
3	0.99	0.11	98.9	1.1	0.0	0.0	0.0
4	1.83	0.03	75.0	25.0	0.0	0.0	0.0
5	58.4	0.03	36.8	63.2	0.0	0.0	0.0
6	537	0.07	1.3	98.5	0.0	0.0	0.2
7	1000	0.2	0.0	99.1	0.0	0.0	0.8
8	1000	0.01	0.1	99.6	0.0	0.0	0.3
9	480	0.02	11.6	88.1	0.0	0.0	0.3
10	480	0.19	9.3	90.7	0.0	0.0	0.0
11	693	0.19	0.2	99.8	0.0	0.0	0.0
12	832	0.11	0.0	98.2	0.0	0.0	1.7
13	1000	0.11	0.1	94.7	0.0	0.0	5.3
14	1000	0.2	0.0	97.6	0.0	0.0	2.4
15	480	0.19	27.4	72.5	0.0	0.0	0.1

d

$[RX]_{cutoff}$ = 160.697 mM		
CV data that satisfy i_{pa}/i_{pc} ∈ [0.65, 0.75] from Stage I of the workflow		
[RX] (mM)	$\log_{10}${[RX] (mM)}	v (V/s) that satisfy i_{pa}/i_{pc} ∈ [0.65, 0.75]
58.4	1.77	0.03, 0.05, 0.08, 0.12, 0.19, 0.3
480	2.68	0.19, 0.3, 0.48, 0.76, 1.2, 1.9
693	2.84	0.19, 0.3, 0.48, 0.76, 1.2, 1.9
480	2.68	0.19, 0.3, 0.48, 0.76, 1.2, 1.9
CV data that satisfy i_{pa}/i_{pc} ∈ [0.65, 0.75] from Stage II of the workflow		
[RX] (mM)	$\log_{10}${[RX] (mM)}	v (V/s) that satisfy i_{pa}/i_{pc} ∈ [0.65, 0.75]
None	None	None

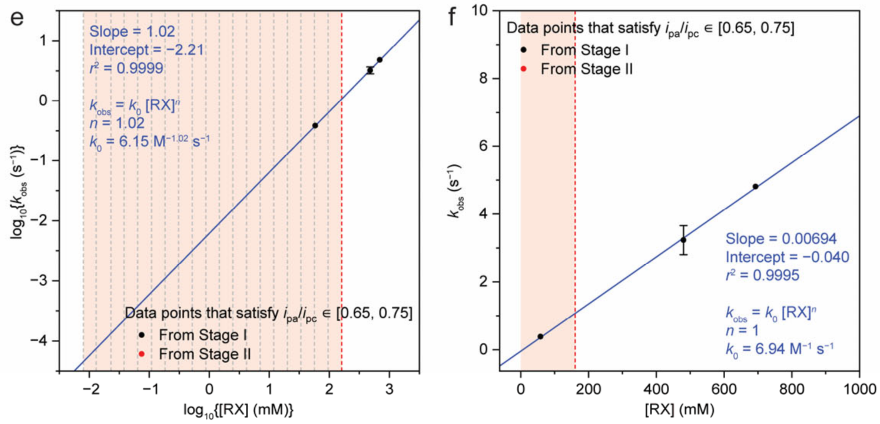

Supplementary Fig. 35. Autonomous investigation of Me_3CCH_2I (neopentyl iodide) that reacts with CoTPP following an *EC* mechanism. (a) CV data (1 mM Co^{II}TPP in DMF with 0.1 M NBu_4PF_6, [RX] ∈ [0.008, 1000] mM, v_{min} ∈ [0.01, 0.2] V/s) measured from Stage I, with detailed parameters and Bayesian optimization results summarized in (b) and (c). (d) Desired combinations of [RX] and v that satisfy i_{pa}/i_{pc} ∈ [0.65, 0.75] from Stage I and Stage II. (e) The $\log_{10}(k_{obs})$ versus $\log_{10}$[RX] plot and (f) the k_{obs} versus [RX] plot based on all the valid k_{obs} values derived from i_{pa}/i_{pc} ∈ [0.65, 0.75] in Stage I (black dots) and Stage II (red dots).

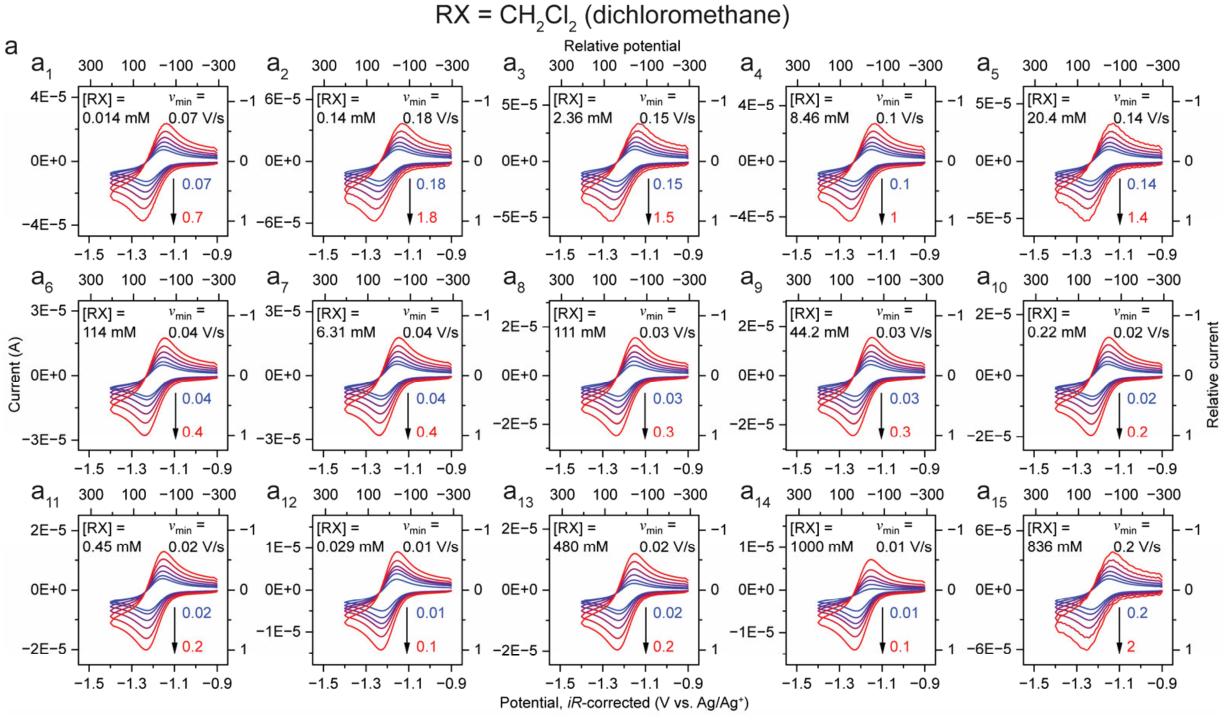

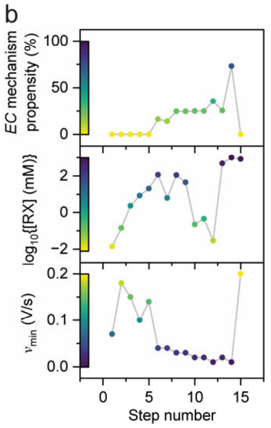

c

Potential range: between −0.9 and −1.4 V vs. Ag/Ag+							
Step	[RX] (mM)	v_{min} (V/s)	Mechanism propensity (%)				
			E	*EC*	*CE*	*DISP1*	*ECE*
1	0.014	0.07	100.0	0.0	0.0	0.0	0.0
2	0.14	0.18	100.0	0.0	0.0	0.0	0.0
3	2.36	0.15	100.0	0.0	0.0	0.0	0.0
4	8.46	0.1	100.0	0.0	0.0	0.0	0.0
5	20.4	0.14	100.0	0.0	0.0	0.0	0.0
6	114	0.04	83.8	16.2	0.0	0.0	0.0
7	6.31	0.04	86.0	14.0	0.0	0.0	0.0
8	111	0.03	75.3	24.7	0.0	0.0	0.0
9	44.2	0.03	75.5	24.5	0.0	0.0	0.0
10	0.22	0.02	75.1	24.9	0.0	0.0	0.0
11	0.45	0.02	75.1	24.9	0.0	0.0	0.0
12	0.029	0.01	64.4	35.6	0.0	0.0	0.0
13	480	0.02	74.6	25.4	0.0	0.0	0.0
14	1000	0.01	26.9	73.1	0.0	0.0	0.0
15	836	0.2	100.0	0.0	0.0	0.0	0.0

d

$[RX]_{cutoff}$ = 10.051 mM		
CV data that satisfy i_{pa}/i_{pc} ∈ [0.65, 0.75] from Stage I of the workflow		
[RX] (mM)	log_{10}{[RX] (mM)}	*v* (V/s) that satisfy i_{pa}/i_{pc} ∈ [0.65, 0.75]
480	2.68	0.02, 0.03, 0.05, 0.08, 0.13, 0.2
1000	3	0.01, 0.02, 0.03, 0.04, 0.06, 1
CV data that satisfy i_{pa}/i_{pc} ∈ [0.65, 0.75] from Stage II of the workflow		
[RX] (mM)	log_{10}{[RX] (mM)}	*v* (V/s) that satisfy i_{pa}/i_{pc} ∈ [0.65, 0.75]
None	None	None

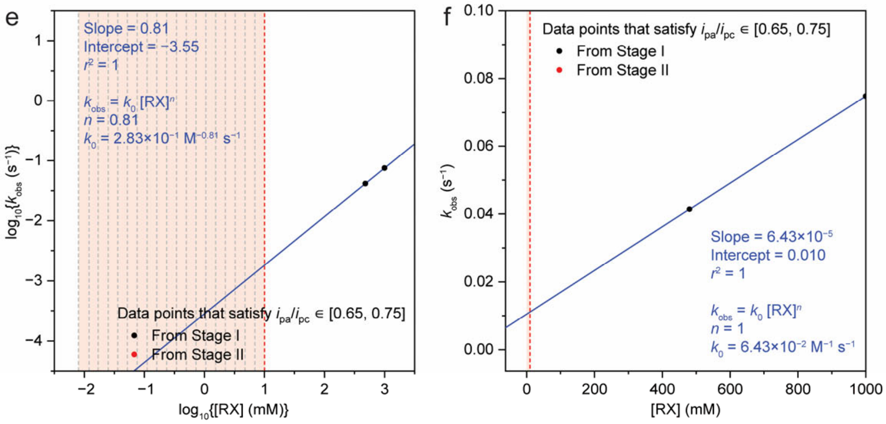

Supplementary Fig. 36. Autonomous investigation of CH_2Cl_2 (dichloromethane) that reacts with CoTPP following an *EC* mechanism. (a) CV data (1 mM Co^{II}TPP in DMF with 0.1 M NBu_4PF_6, [RX] ∈ [0.008, 1000] mM, v_{min} ∈ [0.01, 0.2] V/s) measured from Stage I, with detailed parameters and Bayesian optimization results summarized in (b) and (c). (d) Desired combinations of [RX] and v that satisfy i_{pa}/i_{pc} ∈ [0.65, 0.75] from Stage I and Stage II. (e) The $log_{10}(k_{obs})$ versus log_{10}[RX] plot and (f) the k_{obs} versus [RX] plot based on all the valid k_{obs} values derived from i_{pa}/i_{pc} ∈ [0.65, 0.75] in Stage I (black dots) and Stage II (red dots).

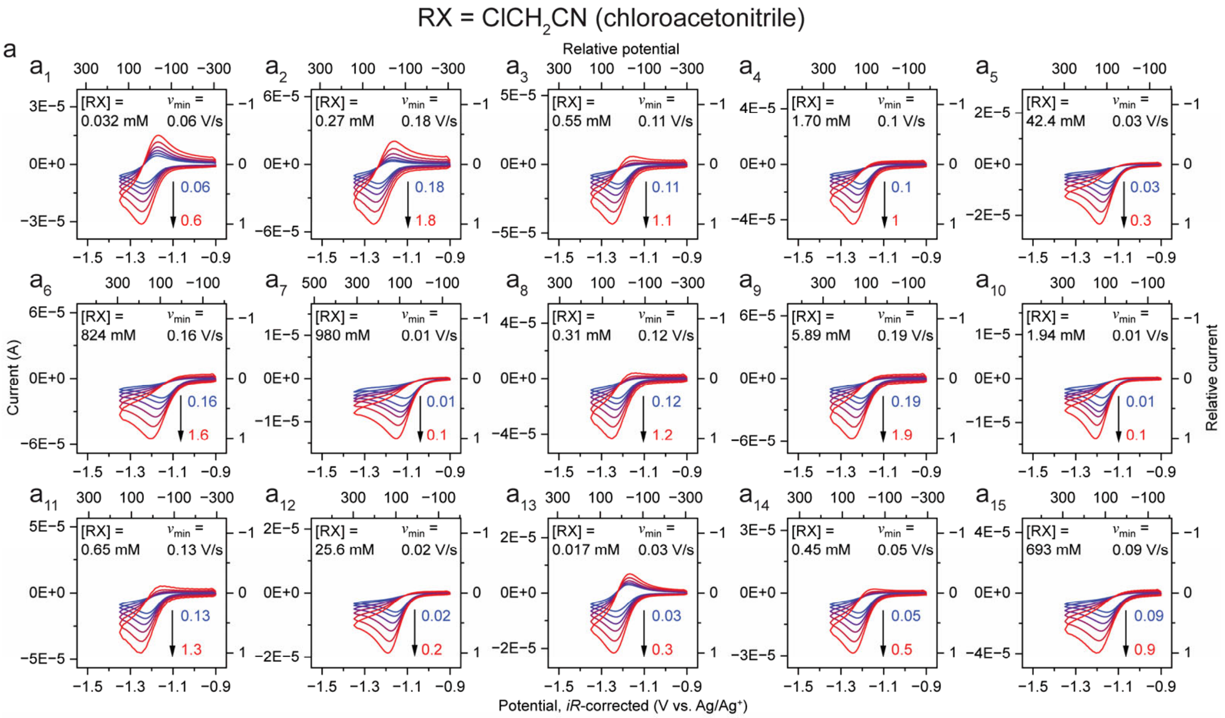

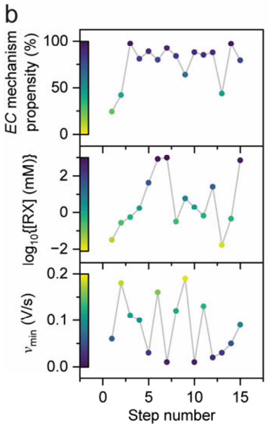

c

Potential range: between −0.9 and −1.35 V vs. Ag/Ag⁺							
Step	[RX] (mM)	v_{min} (V/s)	Mechanism propensity (%)				
			E	*EC*	*CE*	*DISP1*	*ECE*
1	0.032	0.06	75.7	24.3	0.0	0.0	0.0
2	0.27	0.18	57.8	42.2	0.0	0.0	0.0
3	0.55	0.11	0.1	97.5	0.0	0.0	2.4
4	1.70	0.1	0.0	81.0	0.0	0.0	19.0
5	42.4	0.03	11.0	88.9	0.0	0.0	0.1
6	824	0.16	0.1	79.8	0.0	0.0	20.0
7	980	0.01	6.9	92.6	0.0	0.0	0.1
8	0.31	0.12	0.0	83.9	0.0	0.0	16.1
9	5.89	0.19	0.0	63.9	0.0	0.0	36.1
10	1.94	0.01	12.0	88.0	0.0	0.0	0.0
11	0.65	0.13	0.0	85.1	0.0	0.0	14.9
12	25.6	0.02	12.2	87.7	0.0	0.0	0.0
13	0.017	0.03	56.2	43.8	0.0	0.0	0.0
14	0.45	0.05	2.2	97.3	0.0	0.0	0.5
15	693	0.09	0.2	79.3	0.0	0.0	20.5

d

$[RX]_{cutoff}$ = 4.402 mM		
CV data that satisfy $i_{pa}/i_{pc} \in [0.65, 0.75]$ from Stage I of the workflow		
[RX] (mM)	$\log_{10}$\{[RX] (mM)\}	v (V/s) that satisfy $i_{pa}/i_{pc} \in [0.65, 0.75]$
0.27	−0.572	0.18, 0.29, 0.45, 0.72, 1.14, 1.8
0.017	−1.779	0.03, 0.05, 0.08, 0.12, 0.19, 0.3
CV data that satisfy $i_{pa}/i_{pc} \in [0.65, 0.75]$ from Stage II of the workflow		
[RX] (mM)	$\log_{10}$\{[RX] (mM)\}	v (V/s) that satisfy $i_{pa}/i_{pc} \in [0.65, 0.75]$
0.22	−0.656	0.84, 0.924, 1.01, 1.09, 1.18, 1.26
0.38	−0.421	1.29, 1.42, 1.55, 1.68, 1.81, 1.94

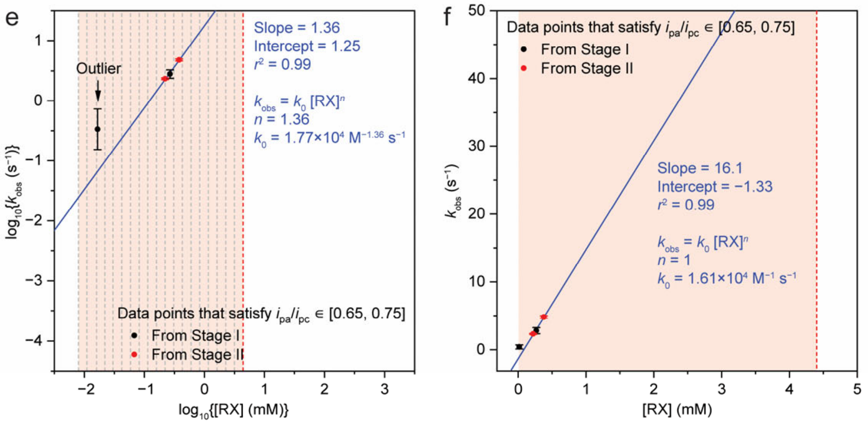

Supplementary Fig. 37. Autonomous investigation of ClCH$_2$CN (chloroacetonitrile) that reacts with CoTPP following an *EC* mechanism. (a) CV data (1 mM Co^{II}TPP in DMF with 0.1 M NBu$_4$PF$_6$, [RX] ∈ [0.008, 1000] mM, v_{min} ∈ [0.01, 0.2] V/s) measured from Stage I, with detailed parameters and Bayesian optimization results summarized in (b) and (c). (d) Desired combinations of [RX] and v that satisfy i_{pa}/i_{pc} ∈ [0.65, 0.75] from Stage I and Stage II. (e) The $\log_{10}(k_{obs})$ versus $\log_{10}$[RX] plot and (f) the k_{obs} versus [RX] plot based on all the valid k_{obs} values derived from i_{pa}/i_{pc} ∈ [0.65, 0.75] in Stage I (black dots) and Stage II (red dots).

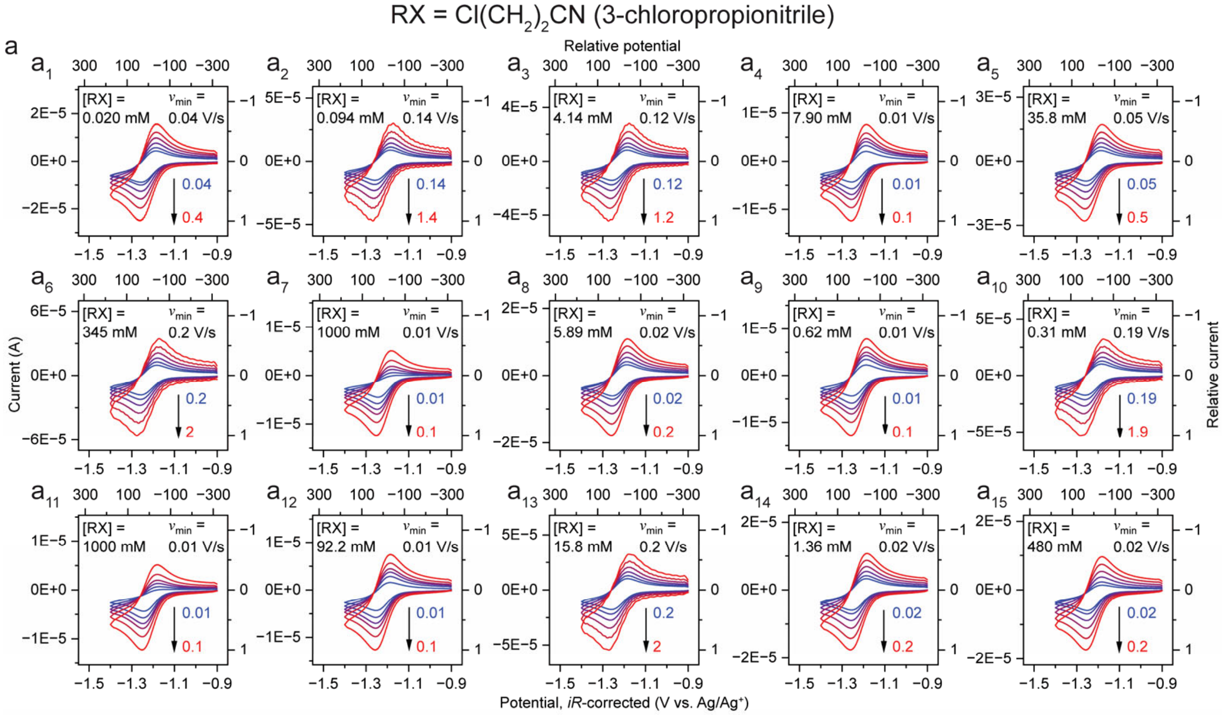

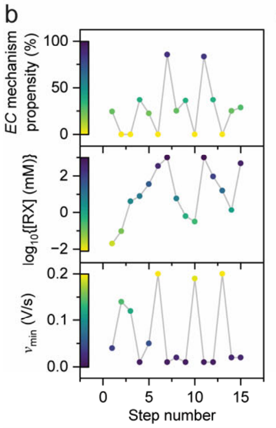

c

Potential range: between −0.9 and −1.4 V vs. Ag/Ag+							
Step	[RX] (mM)	v_{min} (V/s)	Mechanism propensity (%)				
			E	*EC*	*CE*	*DISP1*	*ECE*
1	0.020	0.04	75.7	24.3	0.0	0.0	0.0
2	0.094	0.14	100.0	0.0	0.0	0.0	0.0
3	4.14	0.12	100.0	0.0	0.0	0.0	0.0
4	7.90	0.01	62.9	37.1	0.0	0.0	0.0
5	35.8	0.05	77.7	22.3	0.0	0.0	0.0
6	345	0.2	100.0	0.0	0.0	0.0	0.0
7	1000	0.01	14.6	85.4	0.0	0.0	0.0
8	5.89	0.02	74.9	25.1	0.0	0.0	0.0
9	0.62	0.01	63.8	36.2	0.0	0.0	0.0
10	0.31	0.19	100.0	0.0	0.0	0.0	0.0
11	1000	0.01	16.7	83.3	0.0	0.0	0.0
12	92.2	0.01	63.0	37.0	0.0	0.0	0.0
13	15.8	0.2	100.0	0.0	0.0	0.0	0.0
14	1.36	0.02	75.0	25.0	0.0	0.0	0.0
15	480	0.02	71.4	28.6	0.0	0.0	0.0

d

$[RX]_{cutoff}$ = 242.827 mM		
CV data that satisfy i_{pa}/i_{pc} ∈ [0.65, 0.75] from Stage I of the workflow		
[RX] (mM)	$\log_{10}${[RX] (mM)}	*v* (V/s) that satisfy i_{pa}/i_{pc} ∈ [0.65, 0.75]
1000	3	0.01, 0.02, 0.03, 0.04, 0.06, 0.1
1000	3	0.01, 0.02, 0.03, 0.04, 0.06, 0.1
92.2	1.96	0.01, 0.02, 0.03, 0.04, 0.06, 0.1
480	2.68	0.02, 0.03, 0.05, 0.08, 0.13, 0.2
CV data that satisfy i_{pa}/i_{pc} ∈ [0.65, 0.75] from Stage II of the workflow		
[RX] (mM)	$\log_{10}${[RX] (mM)}	*v* (V/s) that satisfy i_{pa}/i_{pc} ∈ [0.65, 0.75]
173	2.24	0.01, 0.013, 0.016, 0.019, 0.022, 0.025

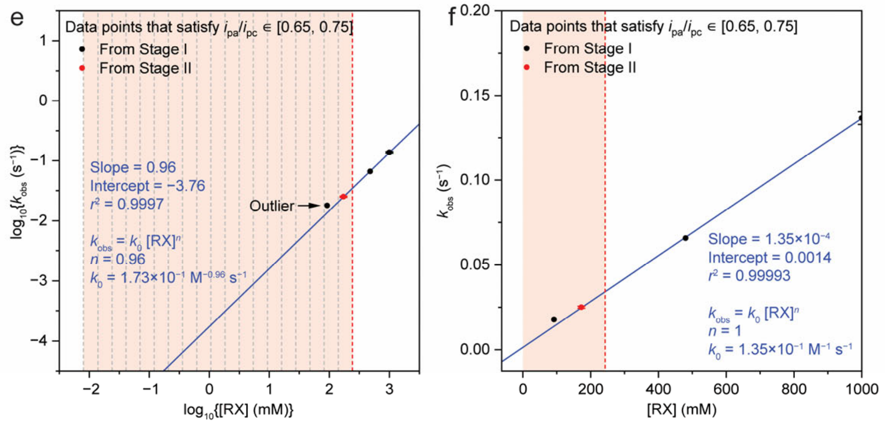

Supplementary Fig. 38. Autonomous investigation of $Cl(CH_2)_2CN$ (3-chloropropionitrile) that reacts with CoTPP following an *EC* mechanism. (a) CV data (1 mM $Co^{II}TPP$ in DMF with 0.1 M NBu_4PF_6, [RX] ∈ [0.008, 1000] mM, v_{min} ∈ [0.01, 0.2] V/s) measured from Stage I, with detailed parameters and Bayesian optimization results summarized in (b) and (c). (d) Desired combinations of [RX] and v that satisfy i_{pa}/i_{pc} ∈ [0.65, 0.75] from Stage I and Stage II. (e) The $\log_{10}(k_{obs})$ versus $\log_{10}$[RX] plot and (f) the k_{obs} versus [RX] plot based on all the valid k_{obs} values derived from i_{pa}/i_{pc} ∈ [0.65, 0.75] in Stage I (black dots) and Stage II (red dots).

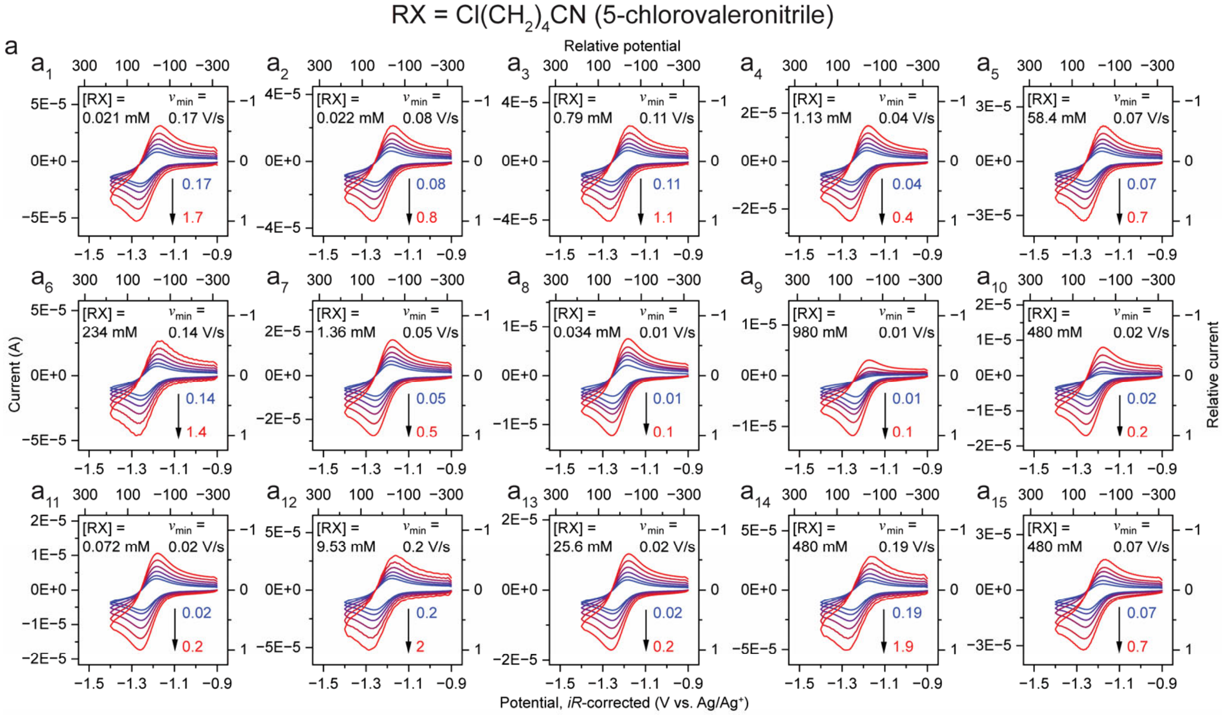

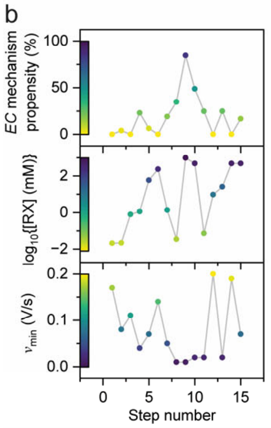

c

Step	[RX] (mM)	v_{min} (V/s)	Mechanism propensity (%) *E*	*EC*	*CE*	*DISP1*	*ECE*
1	0.021	0.17	100.0	0.0	0.0	0.0	0.0
2	0.022	0.08	96.1	3.9	0.0	0.0	0.0
3	0.79	0.11	100.0	0.0	0.0	0.0	0.0
4	1.13	0.04	77.0	23.0	0.0	0.0	0.0
5	58.4	0.07	93.7	6.3	0.0	0.0	0.0
6	234	0.14	100.0	0.0	0.0	0.0	0.0
7	1.36	0.05	80.9	19.1	0.0	0.0	0.0
8	0.034	0.01	65.3	34.7	0.0	0.0	0.0
9	980	0.01	15.3	84.7	0.0	0.0	0.0
10	480	0.02	51.2	48.8	0.0	0.0	0.0
11	0.072	0.02	75.2	24.8	0.0	0.0	0.0
12	9.53	0.2	100.0	0.0	0.0	0.0	0.0
13	25.6	0.02	75.1	24.9	0.0	0.0	0.0
14	480	0.19	100.0	0.0	0.0	0.0	0.0
15	480	0.07	83.3	16.7	0.0	0.0	0.0

Potential range: between −0.9 and −1.4 V vs. Ag/Ag^+

d

$[RX]_{cutoff}$ = 942.73 mM

CV data that satisfy i_{pa}/i_{pc} ∈ [0.65, 0.75] from Stage I of the workflow

[RX] (mM)	$\log_{10}${[RX] (mM)}	v (V/s) that satisfy i_{pa}/i_{pc} ∈ [0.65, 0.75]
480	2.68	0.02, 0.03, 0.05, 0.08, 0.13, 0.2
480	2.68	0.07, 0.11, 0.18, 0.28, 0.44, 0.7

CV data that satisfy i_{pa}/i_{pc} ∈ [0.65, 0.75] from Stage II of the workflow

[RX] (mM)	$\log_{10}${[RX] (mM)}	v (V/s) that satisfy i_{pa}/i_{pc} ∈ [0.65, 0.75]
73.0	1.86	0.01, 0.014, 0.018, 0.022, 0.026, 0.030
113	2.05	0.01, 0.013, 0.016, 0.019, 0.022, 0.025
158	2.20	0.01, 0.017, 0.024, 0.03, 0.037, 0.044
684	2.83	0.063, 0.082, 0.101, 0.12, 0.139, 0.158

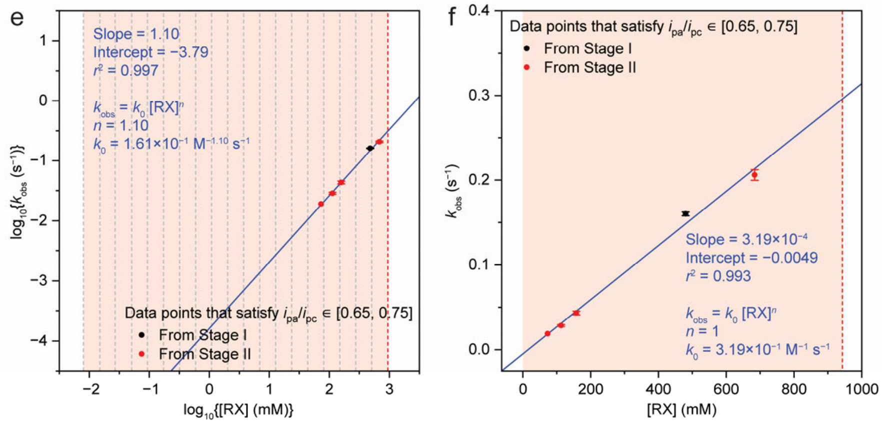

Supplementary Fig. 39. Autonomous investigation of $Cl(CH_2)_4CN$ (5-chlorovaleronitrile) that reacts with CoTPP following an *EC* mechanism. (a) CV data (1 mM Co^{II}TPP in DMF with 0.1 M NBu_4PF_6, [RX] ∈ [0.008, 1000] mM, v_{min} ∈ [0.01, 0.2] V/s) measured from Stage I, with detailed parameters and Bayesian optimization results summarized in (b) and (c). (d) Desired combinations of [RX] and v that satisfy i_{pa}/i_{pc} ∈ [0.65, 0.75] from Stage I and Stage II. (e) The $\log_{10}(k_{obs})$ versus $\log_{10}$[RX] plot and (f) the k_{obs} versus [RX] plot based on all the valid k_{obs} values derived from i_{pa}/i_{pc} ∈ [0.65, 0.75] in Stage I (black dots) and Stage II (red dots).

RX = n-BuBr-d_9 (1-bromobutane-d_9)

a

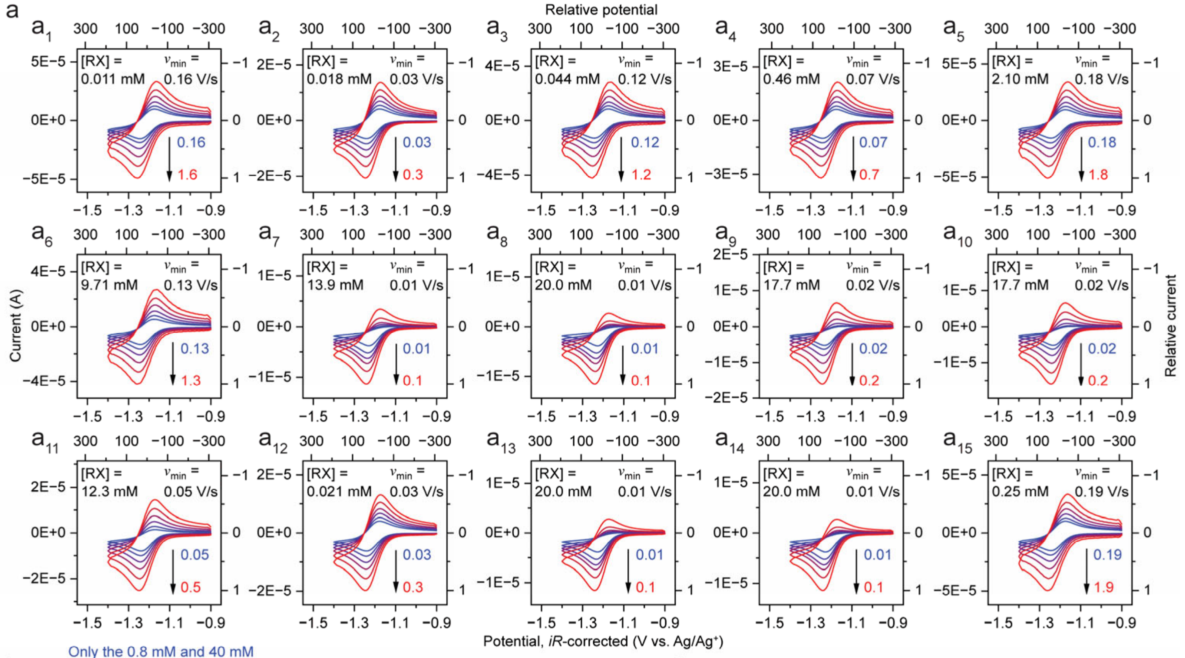

b

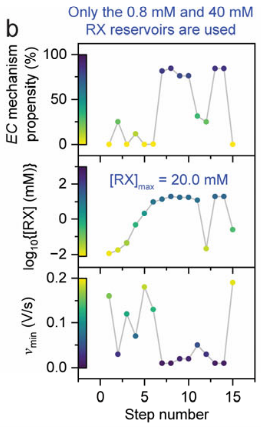

c

Potential range: between −0.9 and −1.4 V vs. Ag/Ag⁺							
Step	[RX] (mM)	v_{min} (V/s)	Mechanism propensity (%)				
			E	*EC*	*CE*	*DISP1*	*ECE*
1	0.011	0.16	100.0	0.0	0.0	0.0	0.0
2	0.018	0.03	75.1	24.9	0.0	0.0	0.0
3	0.044	0.12	99.9	0.1	0.0	0.0	0.0
4	0.46	0.07	88.6	11.4	0.0	0.0	0.0
5	2.10	0.18	100.0	0.0	0.0	0.0	0.0
6	9.71	0.13	99.7	0.3	0.0	0.0	0.0
7	13.9	0.01	18.5	81.5	0.0	0.0	0.0
8	20.0	0.01	15.6	84.4	0.0	0.0	0.0
9	17.7	0.02	24.0	76.0	0.0	0.0	0.0
10	17.7	0.02	23.9	76.1	0.0	0.0	0.0
11	12.3	0.05	68.7	31.3	0.0	0.0	0.0
12	0.021	0.03	75.2	24.8	0.0	0.0	0.0
13	20.0	0.01	15.8	84.2	0.0	0.0	0.0
14	20.0	0.01	15.7	84.3	0.0	0.0	0.0
15	0.25	0.19	100.0	0.0	0.0	0.0	0.0

d

$[RX]_{cutoff}$ = 9.11 mM		
CV data that satisfy $i_{pa}/i_{pc} \in [0.65, 0.75]$ from Stage I of the workflow		
[RX] (mM)	$\log_{10}$\{[RX] (mM)\}	v (V/s) that satisfy $i_{pa}/i_{pc} \in [0.65, 0.75]$
17.7	1.25	0.02, 0.03, 0.05, 0.08, 0.13, 0.2
17.7	1.25	0.02, 0.03, 0.05, 0.08, 0.13, 0.2
12.3	1.09	0.05, 0.08, 0.13, 0.2, 0.32, 0.5
CV data that satisfy $i_{pa}/i_{pc} \in [0.65, 0.75]$ from Stage II of the workflow		
[RX] (mM)	$\log_{10}$\{[RX] (mM)\}	v (V/s) that satisfy $i_{pa}/i_{pc} \in [0.65, 0.75]$
0.79	−0.101	0.01, 0.014, 0.018, 0.022, 0.026, 0.03
1.22	0.086	0.01, 0.014, 0.018, 0.022, 0.026, 0.03
1.90	0.278	0.01, 0.017, 0.024, 0.03, 0.037, 0.044
2.36	0.374	0.015, 0.024, 0.033, 0.042, 0.051, 0.06
3.62	0.559	0.024, 0.036, 0.048, 0.06, 0.072, 0.084
6.14	0.788	0.041, 0.057, 0.072, 0.088, 0.104, 0.119
7.43	0.871	0.048, 0.065, 0.082, 0.099, 0.116, 0.133

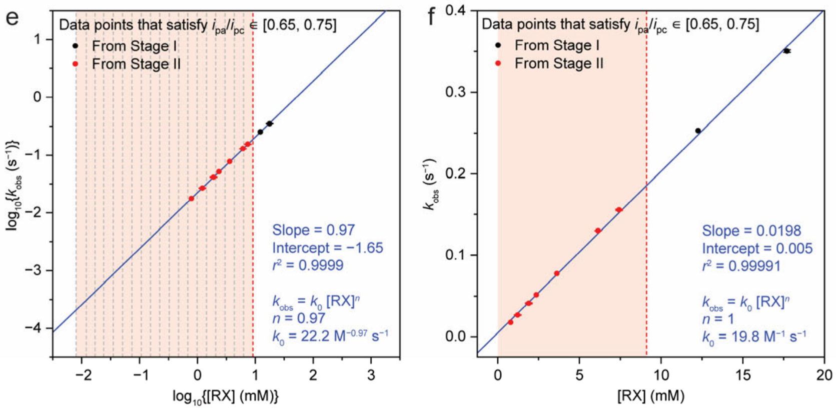

g Secondary α-deuterium kinetic isotope effect (2° KIE *per* α-D)

k_0(n-BuBr) = 31.2, 29.5, 32.0 $M^{-1} s^{-1}$
(see Supplementary Fig. 28 for 3 replicates)

k_0(n-BuBr-d_9) = 19.8 $M^{-1} s^{-1}$

n-BuBr-d_9 has two α-deuterium atoms

2° KIE *per* α-deuterium atom (2° KIE *per* α-D)
= $[k_0(n\text{-BuBr}) / k_0(n\text{-BuBr-}d_9)]^{1/2}$
= 1.25 ± 0.03

Supplementary Fig. 40. Autonomous investigation of *n*-BuBr-d_9 that reacts with CoTPP following an *EC* mechanism. (a) CV data (1 mM Co^{II}TPP in DMF with 0.1 M NBu_4PF_6, [RX] ∈ [0.008, 20] mM because only the 0.8 mM and 40 mM RX reservoirs are used, $v_{min} \in [0.01, 0.2]$ V/s) measured from Stage I, with detailed parameters and Bayesian optimization results summarized in (b) and (c). (d) Desired combinations of [RX] and v that satisfy $i_{pa}/i_{pc} \in [0.65, 0.75]$ from Stage I and Stage II. (e) The $\log_{10}(k_{obs})$ versus $\log_{10}$[RX] plot and (f) the k_{obs} versus [RX] plot based on all the valid k_{obs} values derived from $i_{pa}/i_{pc} \in [0.65, 0.75]$ in Stage I (black dots) and Stage II (red dots). (g) Secondary α-deuterium kinetic isotope effect (2° KIE *per* α-D) for n-BuBr relative to n-BuBr-d_9.

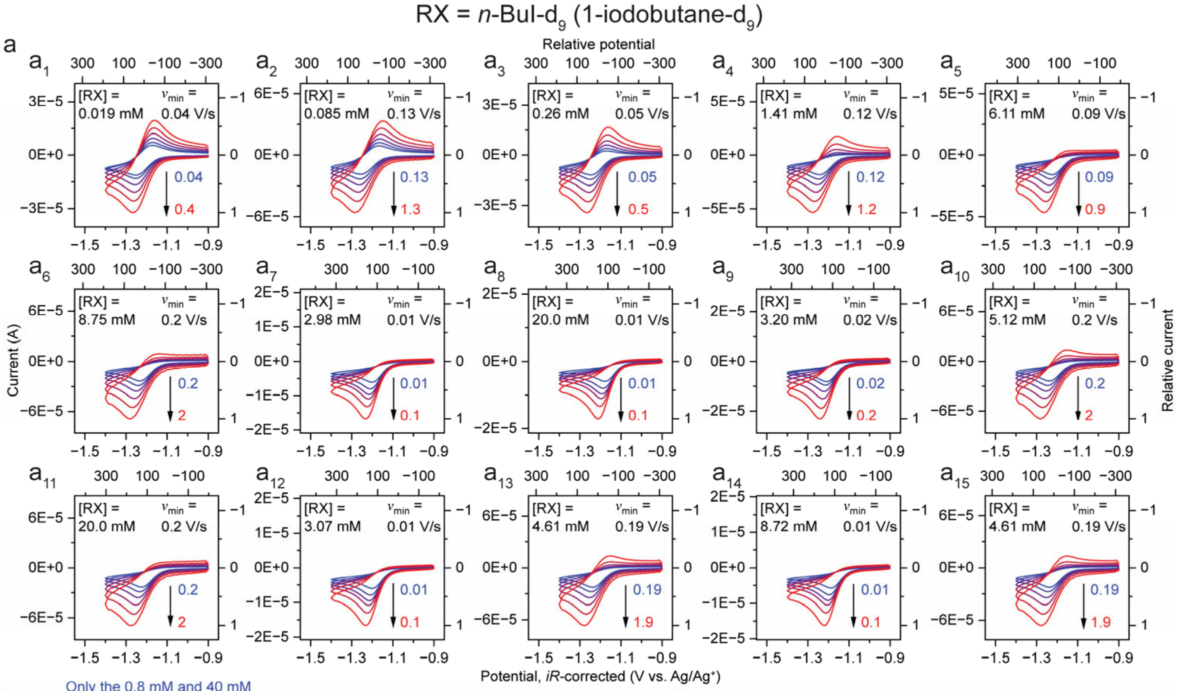

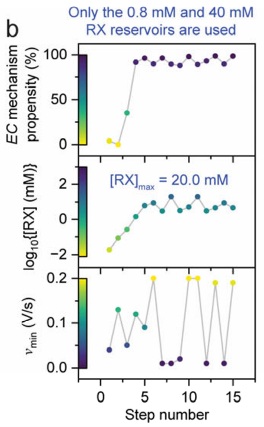

c

Step	[RX] (mM)	v_{min} (V/s)	Mechanism propensity (%)				
			E	*EC*	*CE*	*DISP1*	*ECE*
Potential range: between −0.9 and −1.4 V vs. Ag/Ag+							
1	0.019	0.04	96.2	3.8	0.0	0.0	0.0
2	0.085	0.13	100.0	0.0	0.0	0.0	0.0
3	0.26	0.05	64.9	35.1	0.0	0.0	0.0
4	1.41	0.12	8.5	91.5	0.0	0.0	0.0
5	6.11	0.09	0.4	96.1	0.0	0.0	3.5
6	8.75	0.2	0.1	89.7	0.0	0.0	10.2
7	2.98	0.01	3.5	96.3	0.0	0.0	0.2
8	20.0	0.01	10.7	89.3	0.0	0.0	0.0
9	3.20	0.02	12.1	87.8	0.0	0.0	0.0
10	5.12	0.2	0.3	98.0	0.0	0.0	1.7
11	20.0	0.2	0.3	89.0	0.0	0.0	10.7
12	3.07	0.01	6.9	93.1	0.0	0.0	0.0
13	4.61	0.19	0.3	98.6	0.0	0.0	1.0
14	8.72	0.01	10.4	89.6	0.0	0.0	0.0
15	4.61	0.19	0.4	98.6	0.0	0.0	1.1

d

$[RX]_{cutoff}$ = 3.836 mM		
CV data that satisfy i_{pa}/i_{pc} ∈ [0.65, 0.75] from Stage I of the workflow		
[RX] (mM)	$\log_{10}$\{[RX] (mM)\}	v (V/s) that satisfy i_{pa}/i_{pc} ∈ [0.65, 0.75]
0.26	−0.590	0.05, 0.08, 0.13, 0.2, 0.32, 0.5
1.41	0.149	0.12, 0.19, 0.3, 0.48, 0.76, 1.2
CV data that satisfy i_{pa}/i_{pc} ∈ [0.65, 0.75] from Stage II of the workflow		
[RX] (mM)	$\log_{10}$\{[RX] (mM)\}	v (V/s) that satisfy i_{pa}/i_{pc} ∈ [0.65, 0.75]
0.14	−0.861	0.01, 0.013, 0.016, 0.019, 0.022, 0.025
0.19	−0.717	0.048, 0.065, 0.082, 0.099, 0.116, 0.133
0.38	−0.415	0.226, 0.255, 0.284, 0.313, 0.342, 0.371
0.47	−0.330	0.304, 0.335, 0.367, 0.398, 0.43, 0.461
0.58	−0.240	0.428, 0.471, 0.514, 0.556, 0.599, 0.642
0.81	−0.090	0.556, 0.612, 0.667, 0.723, 0.778, 0.834
2.00	0.302	1.32, 1.45, 1.58, 1.72, 1.85, 1.98
2.53	0.403	1.38, 1.5, 1.63, 1.75, 1.88, 2
2.83	0.451	1.42, 1.54, 1.65, 1.77, 1.88, 2

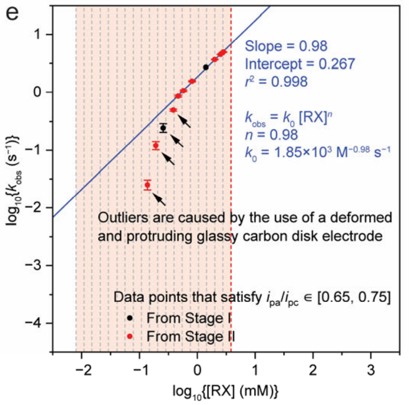

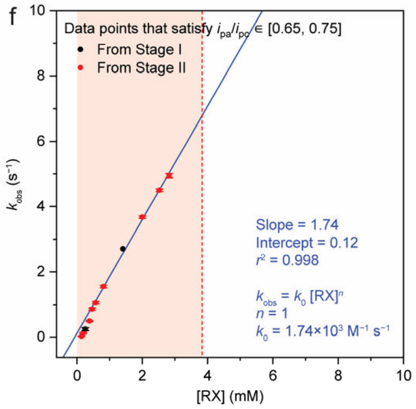

g Secondary α-deuterium kinetic isotope effect (2° KIE *per* α-D)

k_0(*n*-BuI) = 2.87 × 10³ M⁻¹ s⁻¹
(see Supplementary Fig. 29)

k_0(*n*-BuI-d_9) = 1.74 × 10³ M⁻¹ s⁻¹

n-BuI-d_9 has two α-deuterium atoms

2° KIE *per* α-deuterium atom (2° KIE *per* α-D)
= $[k_0(n\text{-BuI}) / k_0(n\text{-BuI-}d_9)]^{1/2}$
= 1.28

Supplementary Fig. 41. Autonomous investigation of *n*-BuI-d_9 that reacts with CoTPP following an *EC* mechanism. (a) CV data (1 mM Co^{II}TPP in DMF with 0.1 M NBu_4PF_6, [RX] ∈ [0.008, 20] mM because only the 0.8 mM and 40 mM RX reservoirs are used, v_{min} ∈ [0.01, 0.2] V/s) measured from Stage I, with detailed parameters and Bayesian optimization results summarized in (b) and (c). (d) Desired combinations of [RX] and v that satisfy i_{pa}/i_{pc} ∈ [0.65, 0.75] from Stage I and Stage II. (e) The $\log_{10}(k_{obs})$ versus $\log_{10}$[RX] plot and (f) the k_{obs} versus [RX] plot based on all the valid k_{obs} values derived from i_{pa}/i_{pc} ∈ [0.65, 0.75] in Stage I (black dots) and Stage II (red dots). (g) Secondary α-deuterium kinetic isotope effect (2° KIE *per* α-D) for *n*-BuI relative to *n*-BuI-d_9.

Supplementary Note 10. Extracting kinetic information of the *C* step between CoTPP and benzyl bromide substrates with fast rates using an alternative approach based on the forward peak position.

As mentioned in the concluding remark of Supplementary Note 5, if the *C* step in an *EC* mechanism is too fast so that the reverse peak of the redox feature completely diminishes in a voltammogram, then kinetic information of the *C* step should be extracted using an alternative approach based on the forward peak position[11, 12]. In the context of an *EC* mechanism between CoTPP and RX, the forward peak position stands for the cathodic peak potential (E_{pc}) of the $Co^{II/I}$ redox. Note that, in this work, our preferred approach for kinetic analysis of the *C* step is based on i_{pa}/i_{pc} (Supplementary Note 5), unless the i_{pa}/i_{pc} approach becomes inapplicable when the studied RX substrate is highly reactive toward the electrogenerated Co^{I}TPP.

Below we first introduce the alternative E_{pc} approach for kinetic analysis of the *C* step in an *EC* mechanism, and then indicate why it is less preferred in this work unless needed.

In an *EC* mechanism between CoTPP and RX, if the *C* step is too fast, the reverse peak of the $Co^{II/I}$ redox feature completely diminishes, accompanied with the shift of E_{pc} in the anodic direction (Eq. S9)[11, 12]:

$$E_{pc} = E_{1/2} - 0.78\frac{RT}{F} + \frac{RT\ln(10)}{2F}\log_{10}\left(\frac{RT\,k_0\,[RX]}{Fv}\right) \qquad \text{(Eq. S9)}$$

where R is the ideal gas constant (8.314 J mol^{-1} K^{-1}), T is the temperature (K), F is the Faraday's constant (96485 C mol^{-1}), $E_{1/2}$ is the formal potential of the $Co^{II/I}$ redox, k_0 is the second-order kinetic rate constant (M^{-1} s^{-1}) of the *C* step between Co^{I}TPP and RX under a pseudo-first-order condition when RX is in large excess, [RX] is the RX concentration, and v is the scan rate. Eq. S9 indicates that the E_{pc} approach for kinetic analysis of the *C* step should be performed at a certain scan rate.

The E_{pc} approach for kinetic analysis of the *C* step is less preferred in this work unless needed, because several factors can affect the accuracy/validity of the measured E_{pc}: (1) any undercompensation of the uncompensated resistance (R_u) between the working and reference electrodes can deviate the measured E_{pc}, and such deviation is more prominent at higher v as the measured current increases; (2) if the preceding *E* step is not fast enough, the peak separation (when uncomplicated by the *C* step) deviates from the ideal 59 mV as v increases, causing a mismatch between the measured E_{pc} and that defined in Eq. S9. Moreover, since there is a notable shift of E_{pc} in the anodic direction, $E_{1/2}$ can no longer be estimated from a single voltammogram (unlike the i_{pa}/i_{pc} approach in Supplementary Note 5), but rather needs to be obtained from a separate voltammogram of the *E* step itself when uncomplicated by the *C* step. Therefore, the E_{pc} approach for kinetic analysis of the *C* step is less preferred in this work unless needed.

Nevertheless, the E_{pc} approach is needed for kinetic analysis of the *C* step between Co^{I}TPP and the benzyl bromide substrates studied in this work because these substrates are highly reactive toward Co^{I}TPP. The i_{pa}/i_{pc} approach becomes inapplicable for these highly reactive substrates because $i_{pa}/i_{pc} \in [0.65, 0.75]$ needs to be satisfied under a low [RX] value that may no longer fulfill a pseudo-first-order condition assuming RX is in large access, meaning

the i_{pa}/i_{pc} approach may be invalid to use. Therefore, the E_{pc} approach is needed for these highly reactive substrates. Kinetic analysis of the C step using the E_{pc} approach is based on voltammograms measured at the lowest v value (0.01 V/s) in the parameter space for the sake of accuracy in measuring E_{pc} (as discussed above).

To verify that the E_{pc} approach can indeed be used to determine k_0 of the C step in an EC mechanism, the k_0 values determined using the i_{pa}/i_{pc} approach versus the E_{pc} approach are compared for six representative RX substrates (n-BuI, n-BuI-d$_9$, $ClCH_2CN$, $Br(CH_2)_3CN$, $I(CH_2)_3CN$, and $PhCH(CH_3)Br$). These six RX substrates are not too reactive toward Co^{I}TPP so that the autonomous workflow can determine their k_0 values using the i_{pa}/i_{pc} approach (the determined k_0 values fall in the range of $[1 \times 10^2, 2 \times 10^4]$ M^{-1} s^{-1}). Meanwhile, these six RX substrates are reactive enough toward Co^{I}TPP to cause a notable anodic shift of E_{pc} at high [RX] values, which validates the use of the E_{pc} approach. Therefore, all the voltammograms measured at 0.01 V/s from the autonomous workflow are selected for determining the k_0 values of these six RX substrates using the E_{pc} approach (see Supplementary Figs. 42 and 43 below): n-BuI, n-BuI-d$_9$, $ClCH_2CN$, $Br(CH_2)_3CN$, $I(CH_2)_3CN$, and $PhCH(CH_3)Br$. The measured E_{pc} values (V vs. Ag/Ag^+, iR-corrected) are plotted against $\log_{10}$[RX] (where the unit [RX] is M). A linear fitting is performed among those anodically shifted E_{pc} values with a fixed slope of 0.02958 (which is the value for $\frac{RT\ln(10)}{2F}$ at 25 °C, see Eq. S9). The k_0 value is extracted from the slope of the linear fitting (which equals $E_{1/2} - 0.78\frac{RT}{F} + \frac{RT\ln(10)}{2F}\log_{10}\left(\frac{RTk_0}{Fv}\right)$, see Eq. S9).

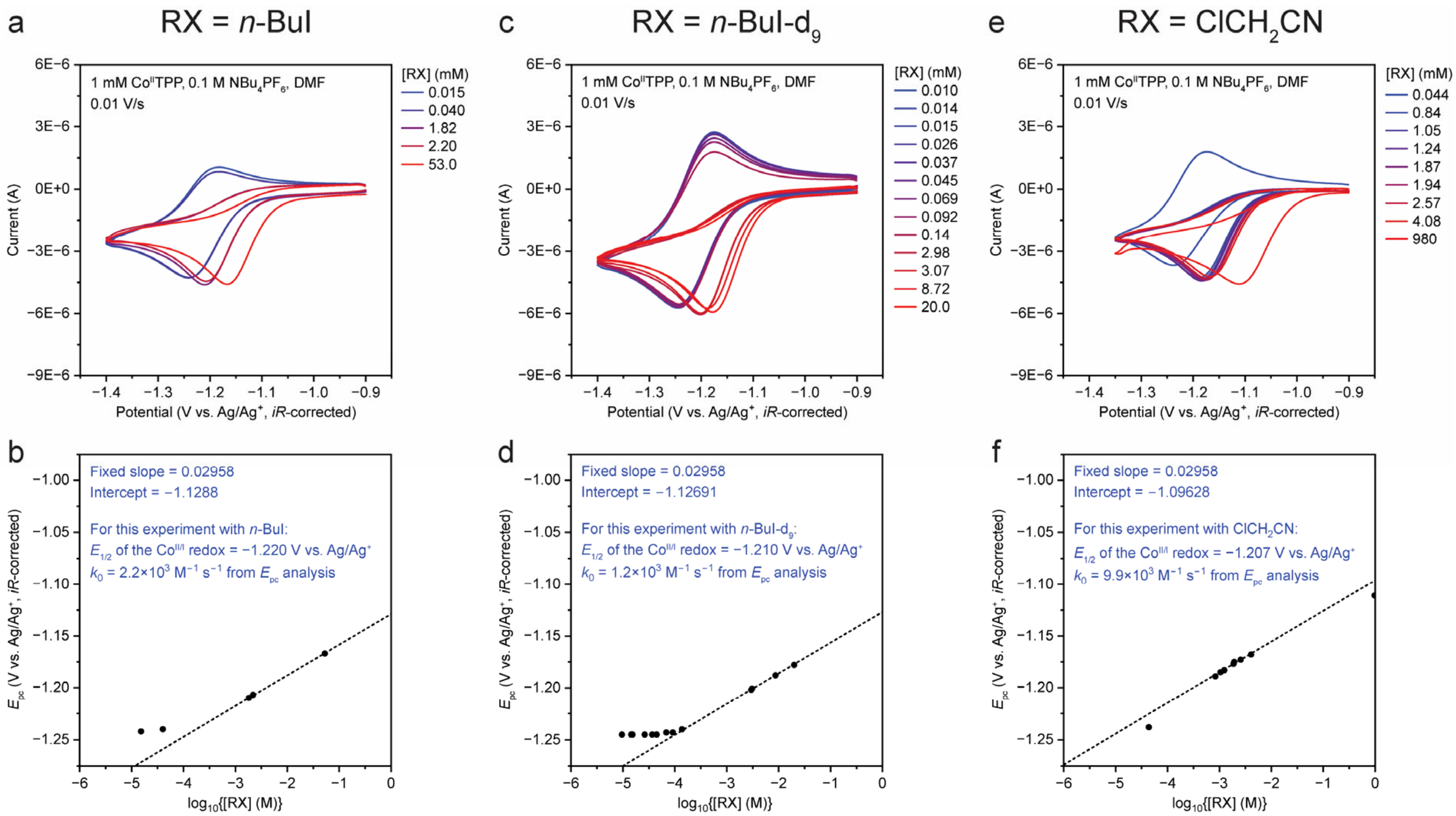

Supplementary Fig. 42. Method validation for determination of k_0 using the E_{pc} approach (part 1). Selected CV data measured at lowest v value (0.01 V/s) in the parameter space from Stage I and Stage II of the workflow,

and the resultant E_{pc} versus $\log_{10}$[RX] plots: (a, b) *n*-BuI; (c, d) *n*-BuI-d$_9$; (e, f) $ClCH_2CN$. Detailed results from the corresponding autonomous investigations of *n*-BuI, *n*-BuI-d$_9$, and $ClCH_2CN$ can be found in Supplementary Figs. 29, 41, and 37, respectively.

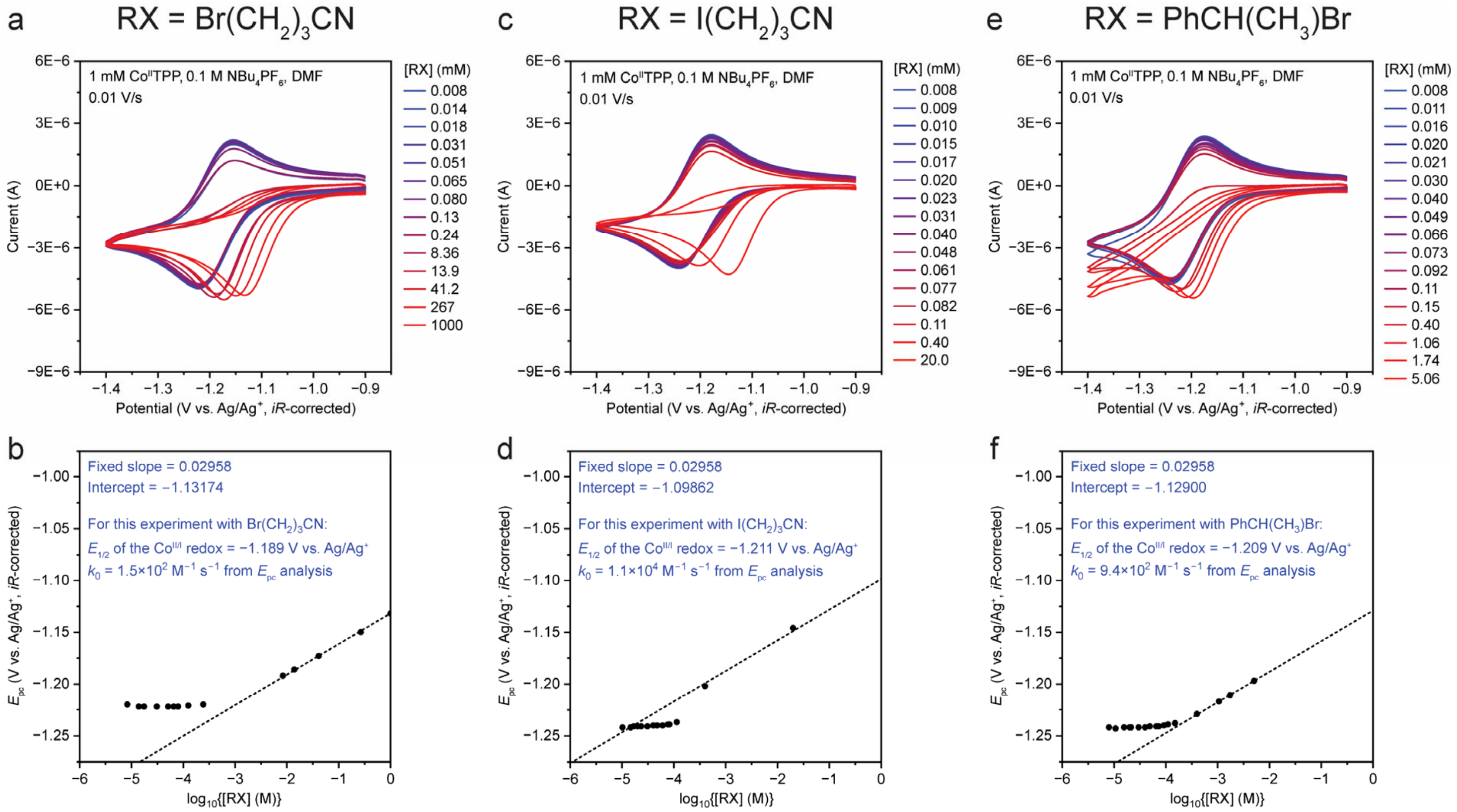

Supplementary Fig. 43. Method validation for determination of k_0 using the E_{pc} approach (part 2). Selected CV data measured at lowest *v* value (0.01 V/s) in the parameter space from Stage I and Stage II of the workflow, and the resultant E_{pc} versus $\log_{10}$[RX] plots: (a, b) $Br(CH_2)_3CN$; (c, d) $I(CH_2)_3CN$; (e, f) $PhCH(CH_3)Br$. Detailed results from the corresponding autonomous investigations of $Br(CH_2)_3CN$, $I(CH_2)_3CN$, and $PhCH(CH_3)Br$ can be found in Supplementary Figs. 66, 67, and 63, respectively.

As shown below in Supplementary Table 12, the ratio of k_0 determined by the E_{pc} approach to k_0 determined by the i_{pa}/i_{pc} approach falls in a narrow statistical range (0.71 ± 0.07) for these six representative RX substrates. Although there exists a systematic error between these two approaches, k_0 values determined by the E_{pc} approach are still comparable across different RX substrates. Therefore, k_0 values of all the benzyl bromide substrates studied in this work (Fig. 4a in the main text) are determined using the E_{pc} approach.

Supplementary Table 12. Comparisons of k_0 determined by the E_{pc} approach with k_0 determined by the i_{pa}/i_{pc} approach for six representative RX substrates.

RX substrate	k_0 (M^{-1} s^{-1})		$\frac{k_0 \text{ determined by the } E_{pc} \text{ approach}}{k_0 \text{ determined by the } i_{pa}/i_{pc} \text{ approach}}$
	The E_{pc} approach	The i_{pa}/i_{pc} approach [1]	
n-BuI	2.2×10^3	2.9×10^3	0.78
n-BuI-d$_9$	1.2×10^3	1.7×10^3	0.69

$ClCH_2CN$	9.9×10^3	1.6×10^4	0.61
$PhCH(CH_3)Br$	9.4×10^2	1.4×10^3	0.67
$Br(CH_2)_3CN$	1.5×10^2	2.1×10^2	0.72
$I(CH_2)_3CN$	1.1×10^4	1.4×10^4	0.81
Average ± standard deviation (among all six RX substrates in this table)			0.71 ± 0.07

[1] The k_0 values determined by the i_{pa}/i_{pc} approach can be found in Supplementary Figs. 29 (*n*-BuI), 41 (*n*-BuI-d_9), 37 ($ClCH_2CN$), 66 ($Br(CH_2)_3CN$), 67 ($I(CH_2)_3CN$), and 63 ($PhCH(CH_3)Br$).

No matter which approach (the i_{pa}/i_{pc} approach vs. the E_{pc} approach) is used for kinetic analysis of the *C* step in an *EC* mechanism, the same autonomous workflow shown in Fig. 1f in the main text (also see a detailed flowchart in Supplementary Fig. 16) is used for experiment execution and data generation. The only difference lies in data analysis to extract k_0. For all the benzyl bromide substrates studied in this work, upon the completion of all the experiment execution and data generation by the autonomous workflow, all the voltammograms measured at the lowest scan rate (0.01 V/s) in the parameter space from both Stage I and Stage II are selected for extracting k_0 from the shift of E_{pc}. Selecting the lowest scan rate for kinetic analysis using the E_{pc} approach is for the sake of accuracy in measuring E_{pc}, as discussed earlier in this Supplementary Note 10. For clarity, the revised workflow for kinetic analysis using the E_{pc} approach is shown below in Supplementary Fig. 44.

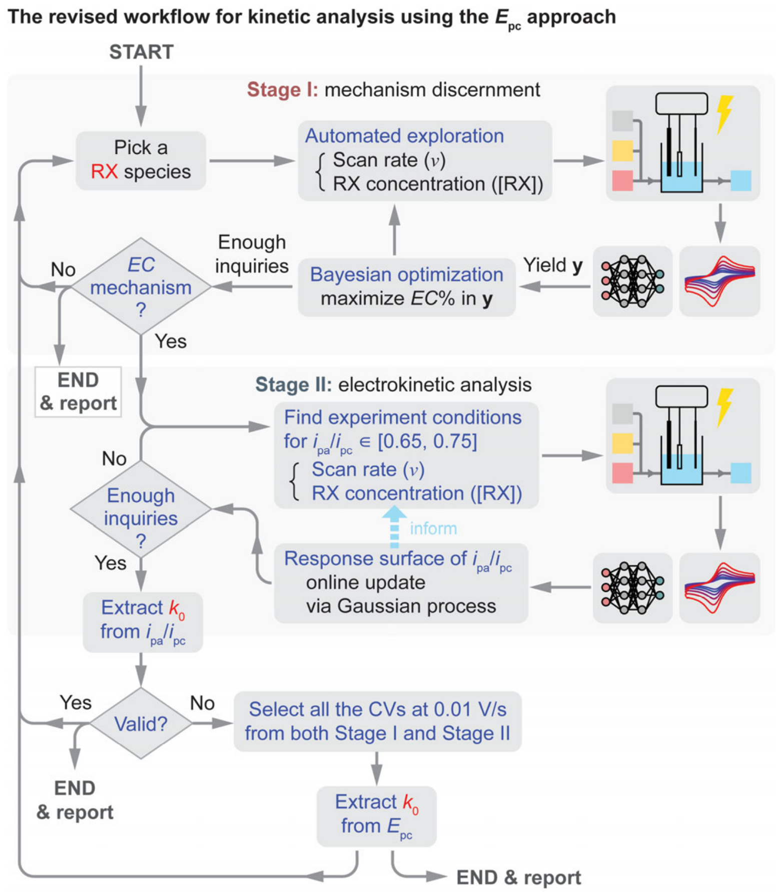

Supplementary Fig. 44. The revised workflow for kinetic analysis of the *C* step between CoTPP and highly reactive RX substrates using the E_{pc} approach. Upon the completion of all the experiment execution and data generation, if the i_{pa}/i_{pc} approach becomes inapplicable for kinetic analysis, the E_{pc} approach is used instead.

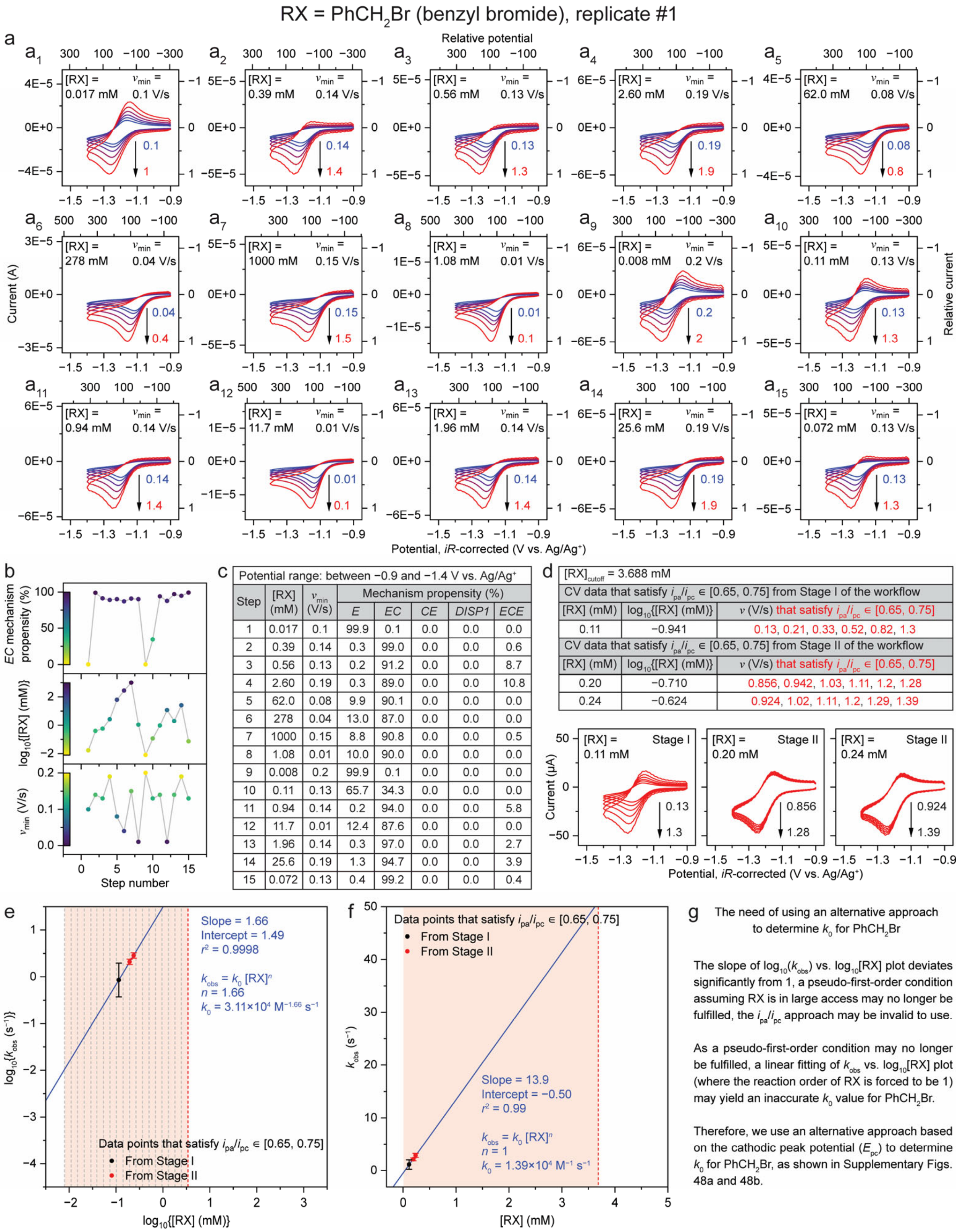

c

Potential range: between −0.9 and −1.4 V vs. Ag/Ag⁺							
Step	[RX] (mM)	v_{min} (V/s)	Mechanism propensity (%)				
			E	*EC*	*CE*	*DISP1*	*ECE*
1	0.017	0.1	99.9	0.1	0.0	0.0	0.0
2	0.39	0.14	0.3	99.0	0.0	0.0	0.6
3	0.56	0.13	0.2	91.2	0.0	0.0	8.7
4	2.60	0.19	0.3	89.0	0.0	0.0	10.8
5	62.0	0.08	9.9	90.1	0.0	0.0	0.0
6	278	0.04	13.0	87.0	0.0	0.0	0.0
7	1000	0.15	8.8	90.8	0.0	0.0	0.5
8	1.08	0.01	10.0	90.0	0.0	0.0	0.0
9	0.008	0.2	99.9	0.1	0.0	0.0	0.0
10	0.11	0.13	65.7	34.3	0.0	0.0	0.0
11	0.94	0.14	0.2	94.0	0.0	0.0	5.8
12	11.7	0.01	12.4	87.6	0.0	0.0	0.0
13	1.96	0.14	0.3	97.0	0.0	0.0	2.7
14	25.6	0.19	1.3	94.7	0.0	0.0	3.9
15	0.072	0.13	0.4	99.2	0.0	0.0	0.4

d

$[RX]_{cutoff}$ = 3.688 mM		
CV data that satisfy $i_{pa}/i_{pc} \in [0.65, 0.75]$ from Stage I of the workflow		
[RX] (mM)	$\log_{10}$\{[RX] (mM)\}	v (V/s) that satisfy $i_{pa}/i_{pc} \in [0.65, 0.75]$
0.11	−0.941	0.13, 0.21, 0.33, 0.52, 0.82, 1.3
CV data that satisfy $i_{pa}/i_{pc} \in [0.65, 0.75]$ from Stage II of the workflow		
[RX] (mM)	$\log_{10}$\{[RX] (mM)\}	v (V/s) that satisfy $i_{pa}/i_{pc} \in [0.65, 0.75]$
0.20	−0.710	0.856, 0.942, 1.03, 1.11, 1.2, 1.28
0.24	−0.624	0.924, 1.02, 1.11, 1.2, 1.29, 1.39

g The need of using an alternative approach to determine k_0 for $PhCH_2Br$

The slope of $\log_{10}(k_{obs})$ vs. $\log_{10}$[RX] plot deviates significantly from 1, a pseudo-first-order condition assuming RX is in large access may no longer be fulfilled, the i_{pa}/i_{pc} approach may be invalid to use.

As a pseudo-first-order condition may no longer be fulfilled, a linear fitting of k_{obs} vs. $\log_{10}$[RX] plot (where the reaction order of RX is forced to be 1) may yield an inaccurate k_0 value for $PhCH_2Br$.

Therefore, we use an alternative approach based on the cathodic peak potential (E_{pc}) to determine k_0 for $PhCH_2Br$, as shown in Supplementary Figs. 48a and 48b.

Supplementary Fig. 45. Autonomous investigation of $PhCH_2Br$ (benzyl bromide) that reacts with CoTPP following an *EC* mechanism (replicate #1). (a) CV data (1 mM Co^{II}TPP in DMF with 0.1 M NBu_4PF_6, [RX] ∈ [0.008, 1000] mM, v_{min} ∈ [0.01, 0.2] V/s) measured from Stage I, with detailed parameters and Bayesian optimization results summarized in (b) and (c). (d) Desired combinations of [RX] and v that satisfy i_{pa}/i_{pc} ∈ [0.65, 0.75] from Stage I and Stage II. (e) The $\log_{10}(k_{obs})$ versus $\log_{10}$[RX] plot and (f) the k_{obs} versus [RX] plot based on all the valid k_{obs} values derived from i_{pa}/i_{pc} ∈ [0.65, 0.75] in Stage I (black dots) and Stage II (red dots). (g) The need of using an alternative approach to determine k_0.

RX = $PhCH_2Br$ (benzyl bromide), replicate #2

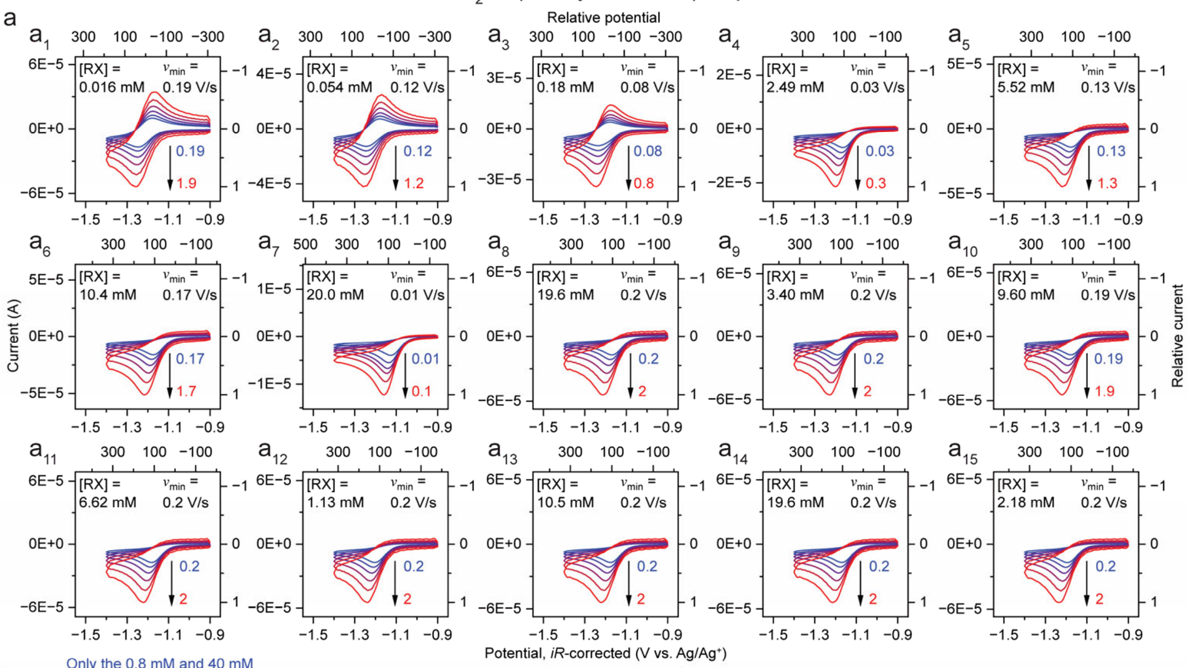

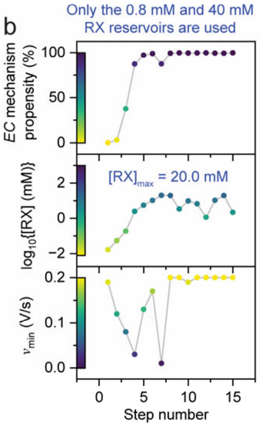

c

Potential range: between −0.9 and −1.4 V vs. Ag/Ag⁺							
Step	[RX] (mM)	v_{min} (V/s)	Mechanism propensity (%)				
			E	*EC*	*CE*	*DISP1*	*ECE*
1	0.016	0.19	100.0	0.0	0.0	0.0	0.0
2	0.054	0.12	97.0	3.0	0.0	0.0	0.0
3	0.18	0.08	62.4	37.6	0.0	0.0	0.0
4	2.49	0.03	12.6	87.4	0.0	0.0	0.0
5	5.52	0.13	2.8	97.2	0.0	0.0	0.0
6	10.4	0.17	1.1	98.9	0.0	0.0	0.0
7	20.0	0.01	12.5	87.5	0.0	0.0	0.0
8	19.6	0.2	0.7	99.3	0.0	0.0	0.0
9	3.40	0.2	0.3	99.7	0.0	0.0	0.0
10	9.60	0.19	0.5	99.5	0.0	0.0	0.0
11	6.62	0.2	0.4	99.6	0.0	0.0	0.0
12	1.13	0.2	0.2	99.8	0.0	0.0	0.0
13	10.5	0.2	0.6	99.4	0.0	0.0	0.0
14	19.6	0.2	0.6	99.4	0.0	0.0	0.0
15	2.18	0.2	0.2	99.8	0.0	0.0	0.0

d

$[RX]_{cutoff}$ = 3.409 mM		
CV data that satisfy $i_{pa}/i_{pc} \in [0.65, 0.75]$ from Stage I of the workflow		
[RX] (mM)	log_{10}\{[RX] (mM)\}	v (V/s) that satisfy $i_{pa}/i_{pc} \in [0.65, 0.75]$
0.18	−0.735	0.08, 0.13, 0.2, 0.32, 0.5, 0.8
CV data that satisfy $i_{pa}/i_{pc} \in [0.65, 0.75]$ from Stage II of the workflow		
[RX] (mM)	log_{10}\{[RX] (mM)\}	v (V/s) that satisfy $i_{pa}/i_{pc} \in [0.65, 0.75]$
0.25	−0.596	0.804, 0.884, 0.965, 1.05, 1.13, 1.21
0.27	−0.563	0.78, 0.858, 0.936, 1.01, 1.09, 1.17

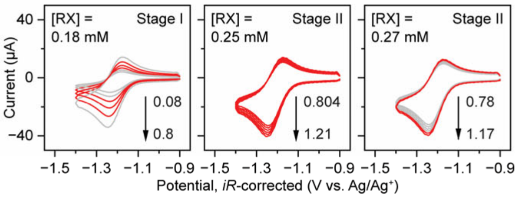

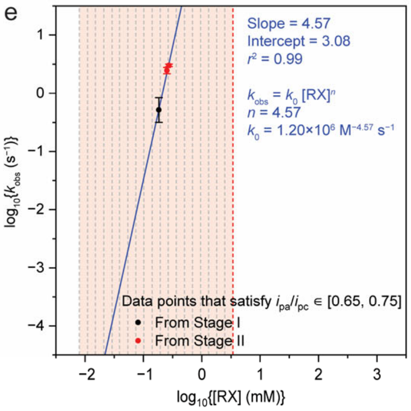

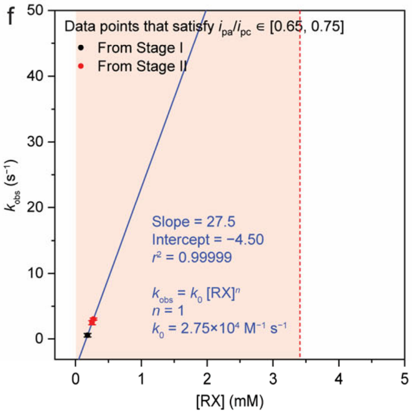

g

The need of using an alternative approach to determine k_0 for $PhCH_2Br$

The slope of $log_{10}(k_{obs})$ vs. log_{10}[RX] plot deviates significantly from 1, a pseudo-first-order condition assuming RX is in large access may no longer be fulfilled, the i_{pa}/i_{pc} approach may be invalid to use.

As a pseudo-first-order condition may no longer be fulfilled, a linear fitting of k_{obs} vs. log_{10}[RX] plot (where the reaction order of RX is forced to be 1) may yield an inaccurate k_0 value for $PhCH_2Br$.

Therefore, we use an alternative approach based on the cathodic peak potential (E_{pc}) to determine k_0 for $PhCH_2Br$, as shown in Supplementary Figs. 48c and 48d.

Supplementary Fig. 46. Autonomous investigation of $PhCH_2Br$ (benzyl bromide) that reacts with CoTPP following an *EC* mechanism (replicate #2). (a) CV data (1 mM Co^{II}TPP in DMF with 0.1 M NBu_4PF_6, [RX] ∈ [0.008, 20] mM because only the 0.8 mM and 40 mM RX reservoirs are used, v_{min} ∈ [0.01, 0.2] V/s) measured from Stage I, with detailed parameters and Bayesian optimization results summarized in (b) and (c). (d) Desired combinations of [RX] and v that satisfy i_{pa}/i_{pc} ∈ [0.65, 0.75] from Stage I and Stage II. (e) The $log_{10}(k_{obs})$ versus log_{10}[RX] plot and (f) the k_{obs} versus [RX] plot based on all the valid k_{obs} values derived from i_{pa}/i_{pc} ∈ [0.65, 0.75] in Stage I (black dots) and Stage II (red dots). (g) The need of using an alternative approach to determine k_0.

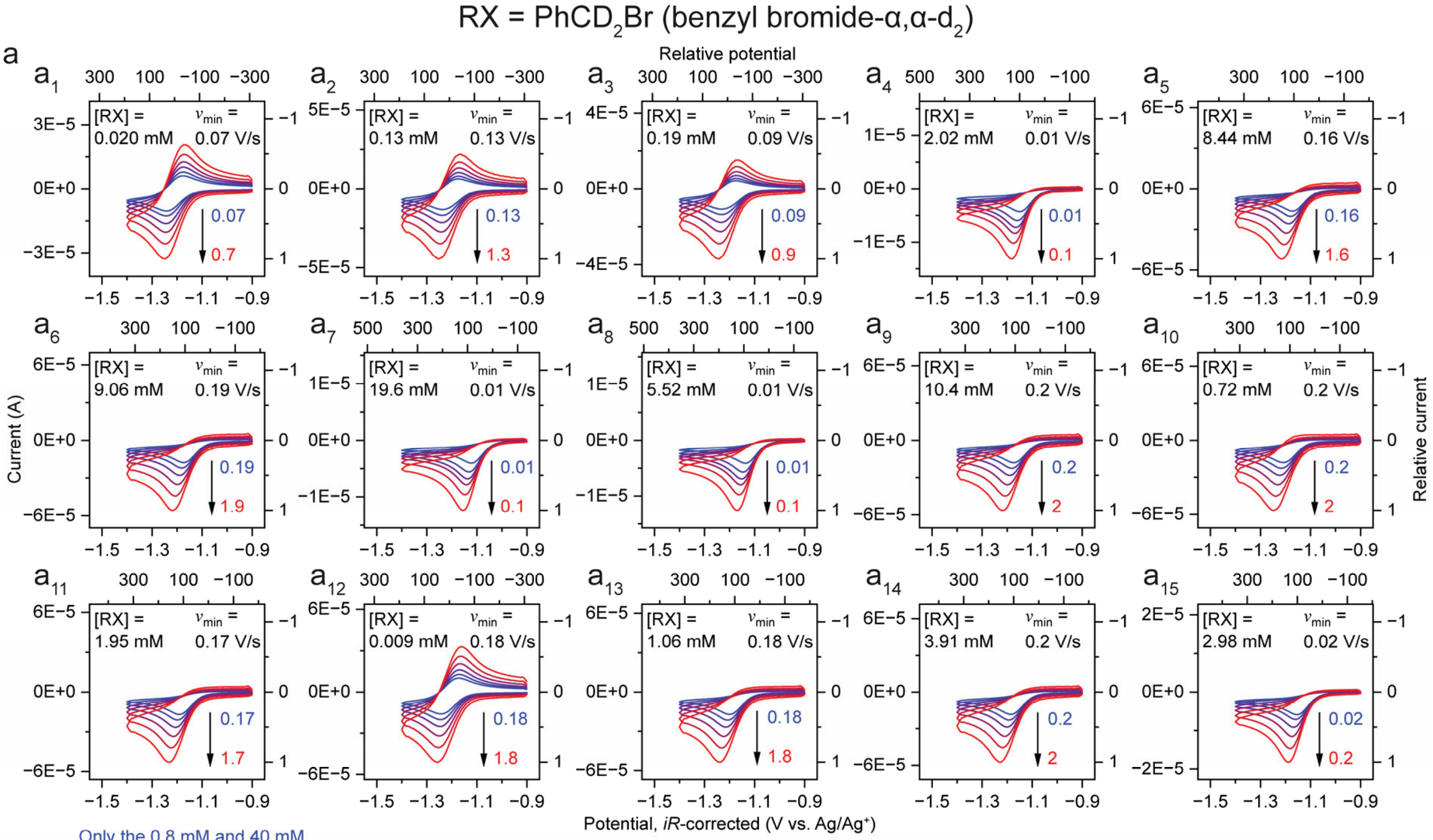

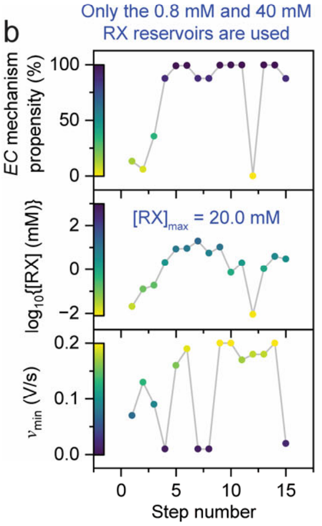

c

Potential range: between −0.9 and −1.4 V vs. Ag/Ag$^+$							
Step	[RX] (mM)	v_{min} (V/s)	Mechanism propensity (%)				
			E	*EC*	*CE*	*DISP1*	*ECE*
1	0.020	0.07	86.9	13.1	0.0	0.0	0.0
2	0.13	0.13	94.1	5.9	0.0	0.0	0.0
3	0.19	0.09	64.4	35.6	0.0	0.0	0.0
4	2.02	0.01	12.5	87.5	0.0	0.0	0.0
5	8.44	0.16	1.0	99.0	0.0	0.0	0.0
6	9.06	0.19	0.7	99.3	0.0	0.0	0.0
7	19.6	0.01	12.5	87.5	0.0	0.0	0.0
8	5.52	0.01	12.5	87.5	0.0	0.0	0.0
9	10.4	0.2	0.4	99.6	0.0	0.0	0.0
10	0.72	0.2	0.2	99.8	0.0	0.0	0.0
11	1.95	0.17	0.3	99.7	0.0	0.0	0.0
12	0.009	0.18	100.0	0.0	0.0	0.0	0.0
13	1.06	0.18	0.2	99.8	0.0	0.0	0.0
14	3.91	0.2	0.3	99.7	0.0	0.0	0.0
15	2.98	0.02	12.6	87.4	0.0	0.0	0.0

d

[RX]$_{cutoff}$ = 16.431 mM		
CV data that satisfy i_{pa}/i_{pc} ∈ [0.65, 0.75] from Stage I of the workflow		
[RX] (mM)	log$_{10}${[RX] (mM)}	v (V/s) that satisfy i_{pa}/i_{pc} ∈ [0.65, 0.75]
0.19	−0.728	0.09, 0.14, 0.23, 0.36, 0.57, 0.9
CV data that satisfy i_{pa}/i_{pc} ∈ [0.65, 0.75] from Stage II of the workflow		
[RX] (mM)	log$_{10}${[RX] (mM)}	v (V/s) that satisfy i_{pa}/i_{pc} ∈ [0.65, 0.75]
0.26	−0.582	1.13, 1.25, 1.36, 1.47, 1.58, 1.7

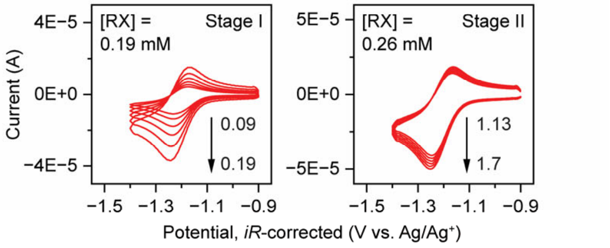

Supplementary Fig. 47. Autonomous investigation of PhCD$_2$Br (benzyl bromide-α,α-d$_2$) that reacts with CoTPP following an *EC* mechanism. (a) CV data (1 mM Co^{II}TPP in DMF with 0.1 M NBu$_4$PF$_6$, [RX] ∈ [0.008, 20] mM because only the 0.8 mM and 40 mM RX reservoirs are used, v_{min} ∈ [0.01, 0.2] V/s) measured from Stage I, with detailed parameters and Bayesian optimization results summarized in (b) and (c). (d) Desired combinations of [RX] and v that satisfy i_{pa}/i_{pc} ∈ [0.65, 0.75] from Stage I and Stage II. There seems to be usable CV data under two [RX] values for determining k_0 of the *C* step using the i_{pa}/i_{pc} approach. However, the corresponding CV responses in (d) may no longer fulfill a pseudo-first-order condition assuming RX is in large excess, meaning the i_{pa}/i_{pc} approach may be invalid to use. Instead, k_0 of the *C* step is determined by the E_{pc} approach (Supplementary Note 10) based on the selected CV data measured at the lowest v value (0.01 V/s) in the parameter space from Stage I and Stage II (Supplementary Figs. 48e and 48f).

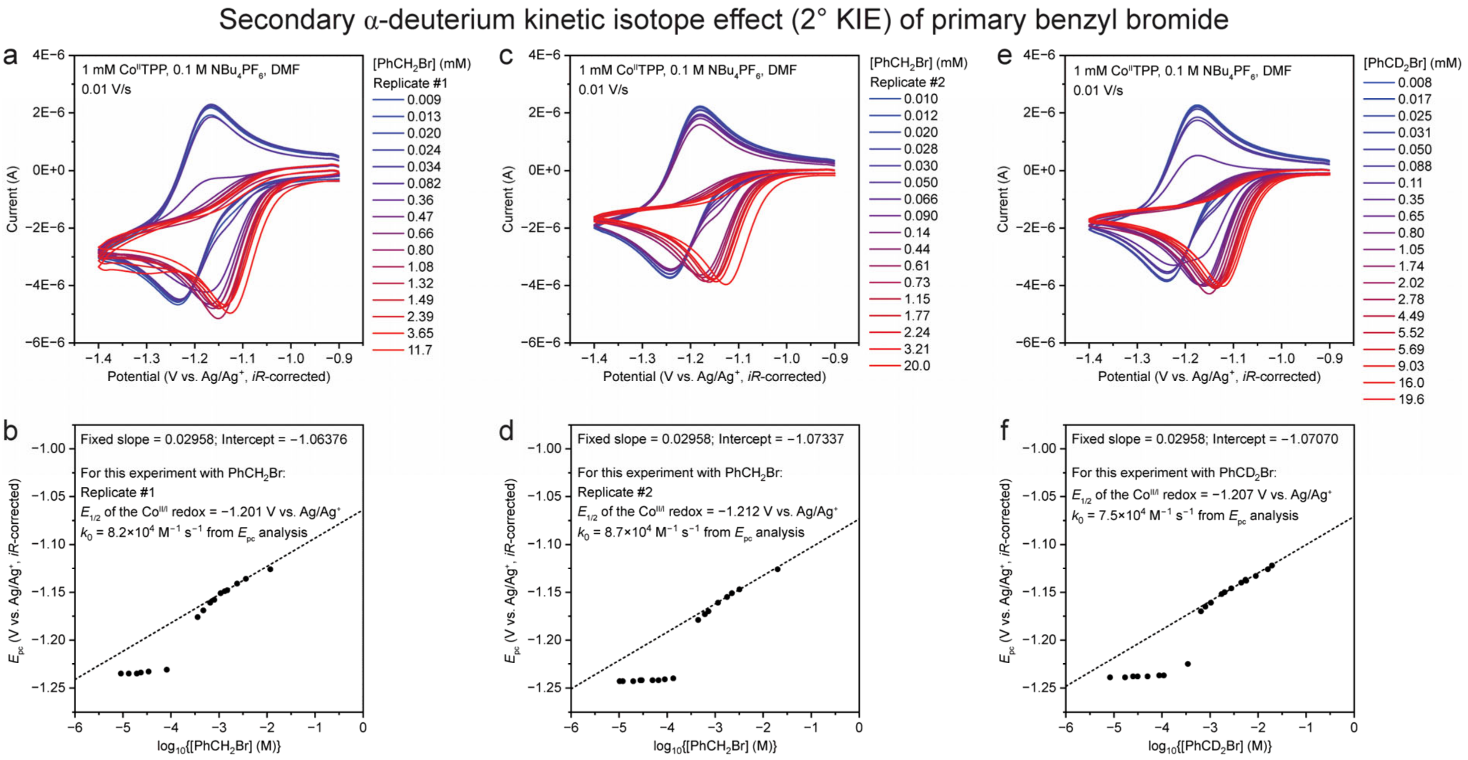

Supplementary Fig. 48. Determination of k_0 for $PhCH_2Br$ and $PhCD_2Br$ using the E_{pc} approach. Selected CV data measured at lowest *v* value (0.01 V/s) in the parameter space from Stage I and Stage II of the workflow, and the resultant E_{pc} versus $\log_{10}$[RX] plots: (a, b) $PhCH_2Br$ (replicate #1); (c, d) $PhCH_2Br$ (replicate #2); (e, f) $PhCD_2Br$. Detailed results from the corresponding autonomous investigations of $PhCH_2Br$ and $PhCD_2Br$ can be found in Supplementary Figs. 45–47.

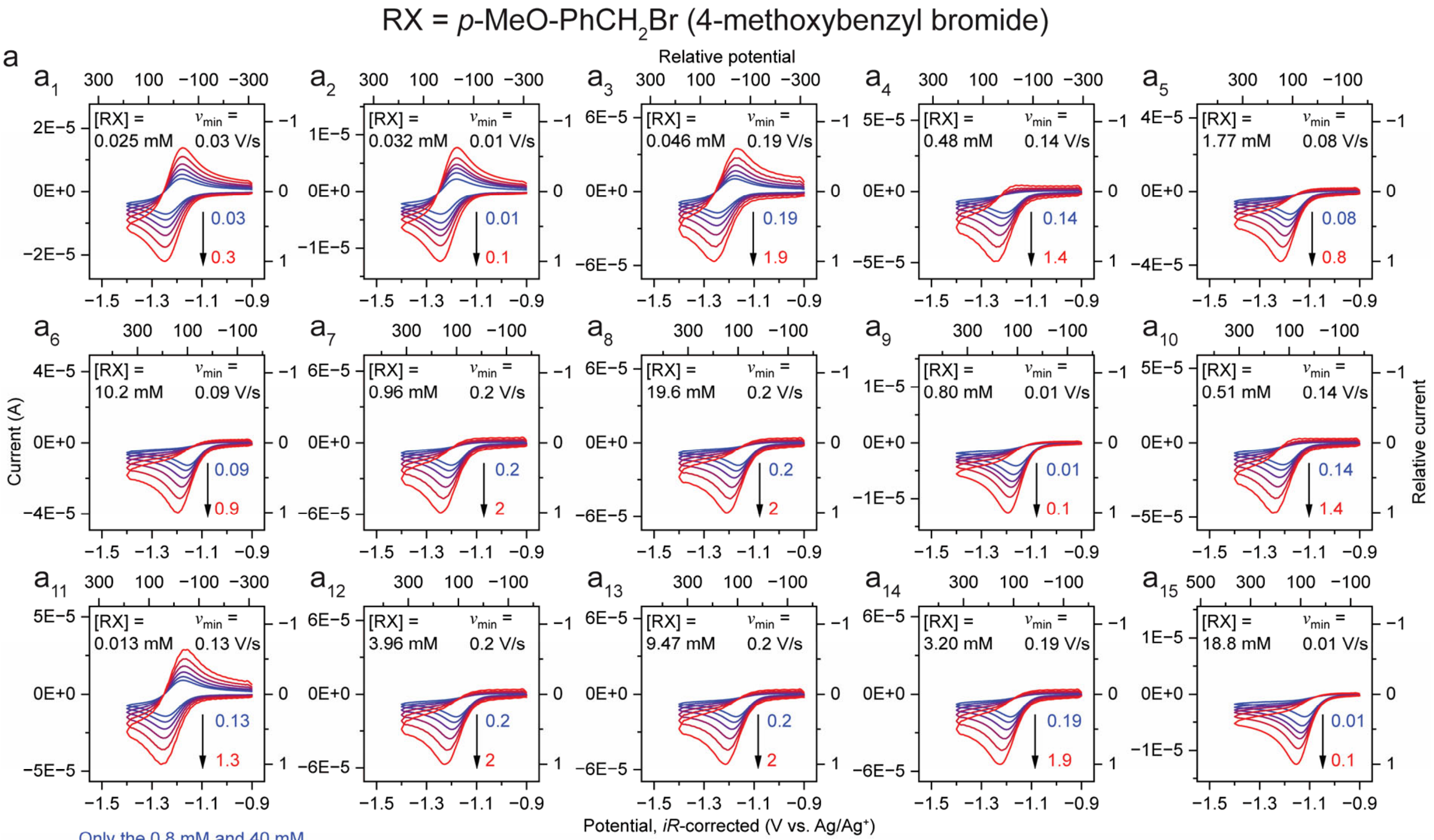

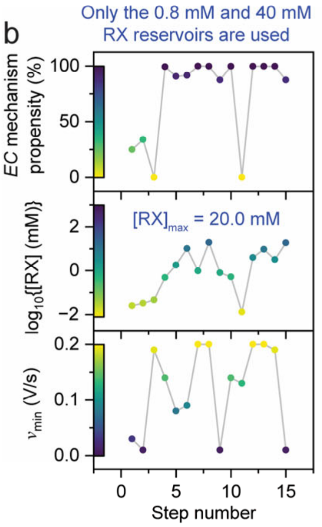

c

Potential range: between −0.9 and −1.4 V vs. Ag/Ag+							
Step	[RX] (mM)	v_{min} (V/s)	Mechanism propensity (%)				
			E	*EC*	*CE*	*DISP1*	*ECE*
1	0.025	0.03	75.1	24.9	0.0	0.0	0.0
2	0.032	0.01	66.2	33.8	0.0	0.0	0.0
3	0.046	0.19	100.0	0.0	0.0	0.0	0.0
4	0.48	0.14	0.7	99.3	0.0	0.0	0.0
5	1.77	0.08	9.2	90.8	0.0	0.0	0.0
6	10.2	0.09	8.4	91.6	0.0	0.0	0.0
7	0.96	0.2	0.1	99.8	0.0	0.0	0.0
8	19.6	0.2	0.2	99.8	0.0	0.0	0.0
9	0.80	0.01	12.5	87.5	0.0	0.0	0.0
10	0.51	0.14	0.2	99.8	0.0	0.0	0.0
11	0.013	0.13	100.0	0.0	0.0	0.0	0.0
12	3.96	0.2	0.2	99.8	0.0	0.0	0.0
13	9.47	0.2	0.2	99.8	0.0	0.0	0.0
14	3.20	0.19	0.2	99.8	0.0	0.0	0.0
15	18.8	0.01	12.5	87.5	0.0	0.0	0.0

d

$[RX]_{cutoff}$ = 3.151 mM		
CV data that satisfy i_{pa}/i_{pc} ∈ [0.65, 0.75] from Stage I of the workflow		
[RX] (mM)	log_{10}{[RX] (mM)}	*v* (V/s) that satisfy i_{pa}/i_{pc} ∈ [0.65, 0.75]
None	None	None
CV data that satisfy i_{pa}/i_{pc} ∈ [0.65, 0.75] from Stage II of the workflow		
[RX] (mM)	log_{10}{[RX] (mM)}	*v* (V/s) that satisfy i_{pa}/i_{pc} ∈ [0.65, 0.75]
None	None	None

Supplementary Fig. 49. Autonomous investigation of *p*-MeO-PhCH$_2$Br (4-methoxybenzyl bromide) that reacts with CoTPP following an *EC* mechanism. (a) CV data (1 mM Co^{II}TPP in DMF with 0.1 M NBu$_4$PF$_6$, [RX] ∈ [0.008, 20] mM because only the 0.8 mM and 40 mM RX reservoirs are used, v_{min} ∈ [0.01, 0.2] V/s) measured from Stage I, with detailed parameters and Bayesian optimization results summarized in (b) and (c). (d) Desired combinations of [RX] and *v* that satisfy i_{pa}/i_{pc} ∈ [0.65, 0.75] from Stage I and Stage II. There is no usable CV data for determining k_0 of the *C* step using the i_{pa}/i_{pc} approach. Instead, k_0 of the *C* step is determined by the E_{pc} approach (Supplementary Note 10) based on the selected CV data measured at the lowest *v* value (0.01 V/s) in the parameter space from Stage I and Stage II (Supplementary Figs. 59a and 60a).

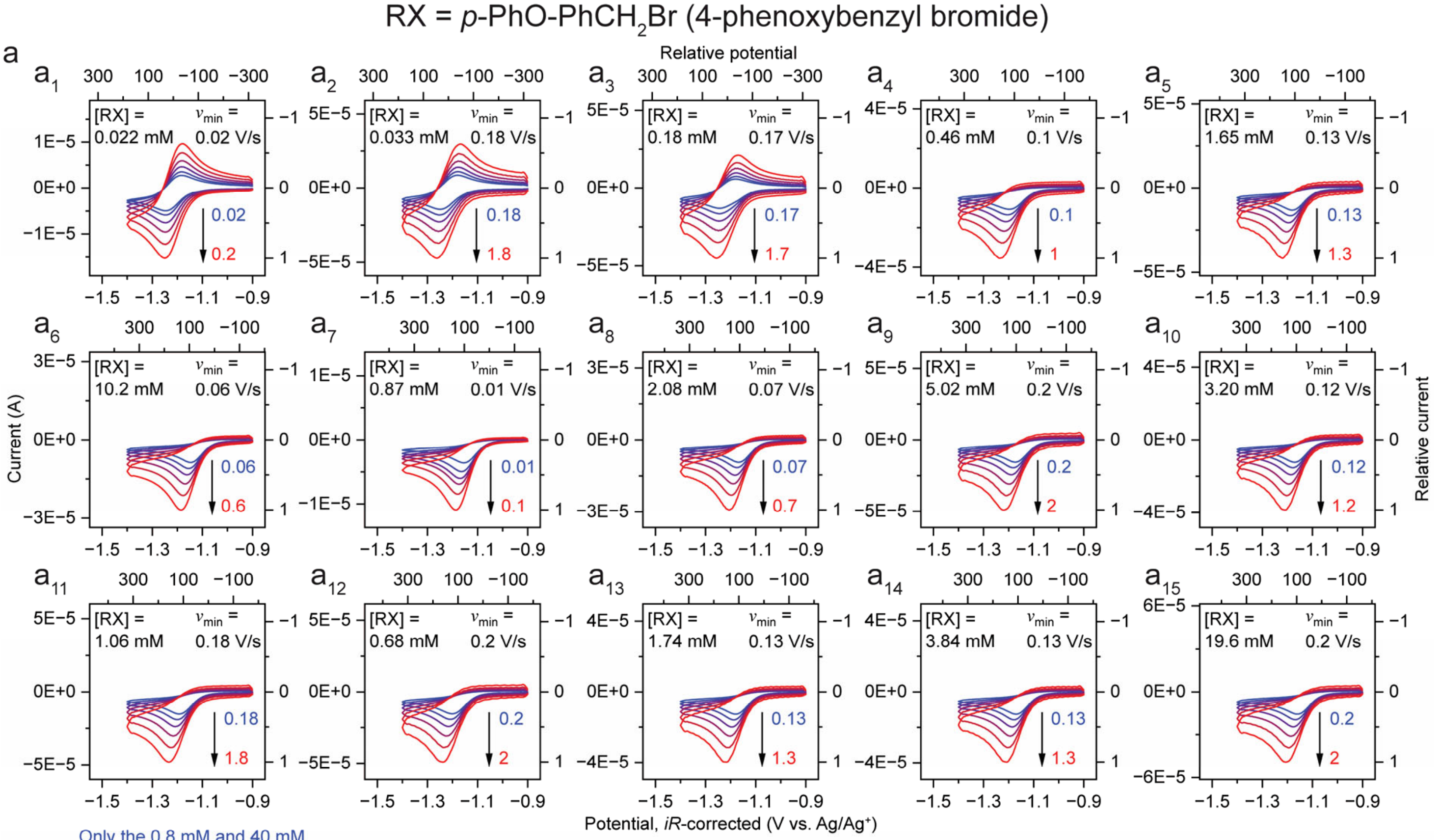

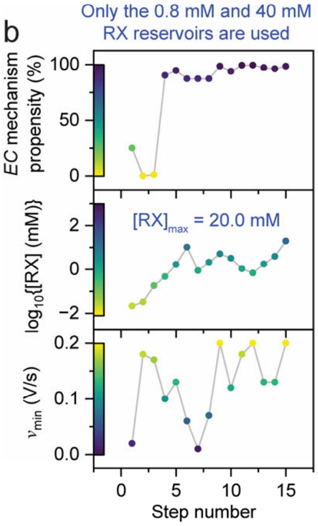

c

Potential range: between −0.9 and −1.4 V vs. Ag/Ag+							
Step	[RX] (mM)	v_{min} (V/s)	Mechanism propensity (%)				
			E	*EC*	*CE*	*DISP1*	*ECE*
1	0.022	0.02	75.0	25.0	0.0	0.0	0.0
2	0.033	0.18	100.0	0.0	0.0	0.0	0.0
3	0.18	0.17	98.9	1.1	0.0	0.0	0.0
4	0.46	0.1	9.5	90.5	0.0	0.0	0.0
5	1.65	0.13	5.2	94.8	0.0	0.0	0.0
6	10.2	0.06	12.6	87.4	0.0	0.0	0.0
7	0.87	0.01	12.6	87.4	0.0	0.0	0.0
8	2.08	0.07	12.6	87.4	0.0	0.0	0.0
9	5.02	0.2	1.5	98.5	0.0	0.0	0.0
10	3.20	0.12	6.0	94.0	0.0	0.0	0.0
11	1.06	0.18	0.7	99.3	0.0	0.0	0.0
12	0.68	0.2	0.6	99.4	0.0	0.0	0.0
13	1.74	0.13	2.8	97.2	0.0	0.0	0.0
14	3.84	0.13	3.7	96.3	0.0	0.0	0.0
15	19.6	0.2	1.6	98.4	0.0	0.0	0.0

d

$[RX]_{cutoff}$ = 1.89 mM		
CV data that satisfy $i_{pa}/i_{pc} \in [0.65, 0.75]$ from Stage I of the workflow		
[RX] (mM)	$\log_{10}$\{[RX] (mM)\}	v (V/s) that satisfy $i_{pa}/i_{pc} \in [0.65, 0.75]$
None	None	None
CV data that satisfy $i_{pa}/i_{pc} \in [0.65, 0.75]$ from Stage II of the workflow		
[RX] (mM)	$\log_{10}$\{[RX] (mM)\}	v (V/s) that satisfy $i_{pa}/i_{pc} \in [0.65, 0.75]$
0.32	−0.494	0.01, 0.014, 0.018, 0.022, 0.026, 0.03
0.34	−0.471	1.13, 1.25, 1.36, 1.47, 1.58, 1.7

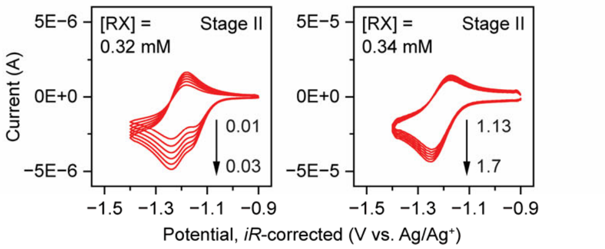

Supplementary Fig. 50. Autonomous investigation of *p*-PhO-PhCH$_2$Br (4-phenoxybenzyl bromide) that reacts with CoTPP following an *EC* mechanism. (a) CV data (1 mM Co^{II}TPP in DMF with 0.1 M NBu$_4$PF$_6$, [RX] ∈ [0.008, 20] mM because only the 0.8 mM and 40 mM RX reservoirs are used, v_{min} ∈ [0.01, 0.2] V/s) measured from Stage I, with detailed parameters and Bayesian optimization results summarized in (b) and (c). (d) Desired combinations of [RX] and v that satisfy i_{pa}/i_{pc} ∈ [0.65, 0.75] from Stage I and Stage II. There seems to be usable CV data under two [RX] values for determining k_0 of the *C* step using the i_{pa}/i_{pc} approach. However, the corresponding CV responses in (d) may no longer fulfill a pseudo-first-order condition assuming RX is in large excess, meaning the i_{pa}/i_{pc} approach may be invalid to use. Instead, k_0 of the *C* step is determined by the E_{pc} approach (Supplementary Note 10) based on the selected CV data measured at the lowest v value (0.01 V/s) in the parameter space from Stage I and Stage II (Supplementary Figs. 59b and 60b).

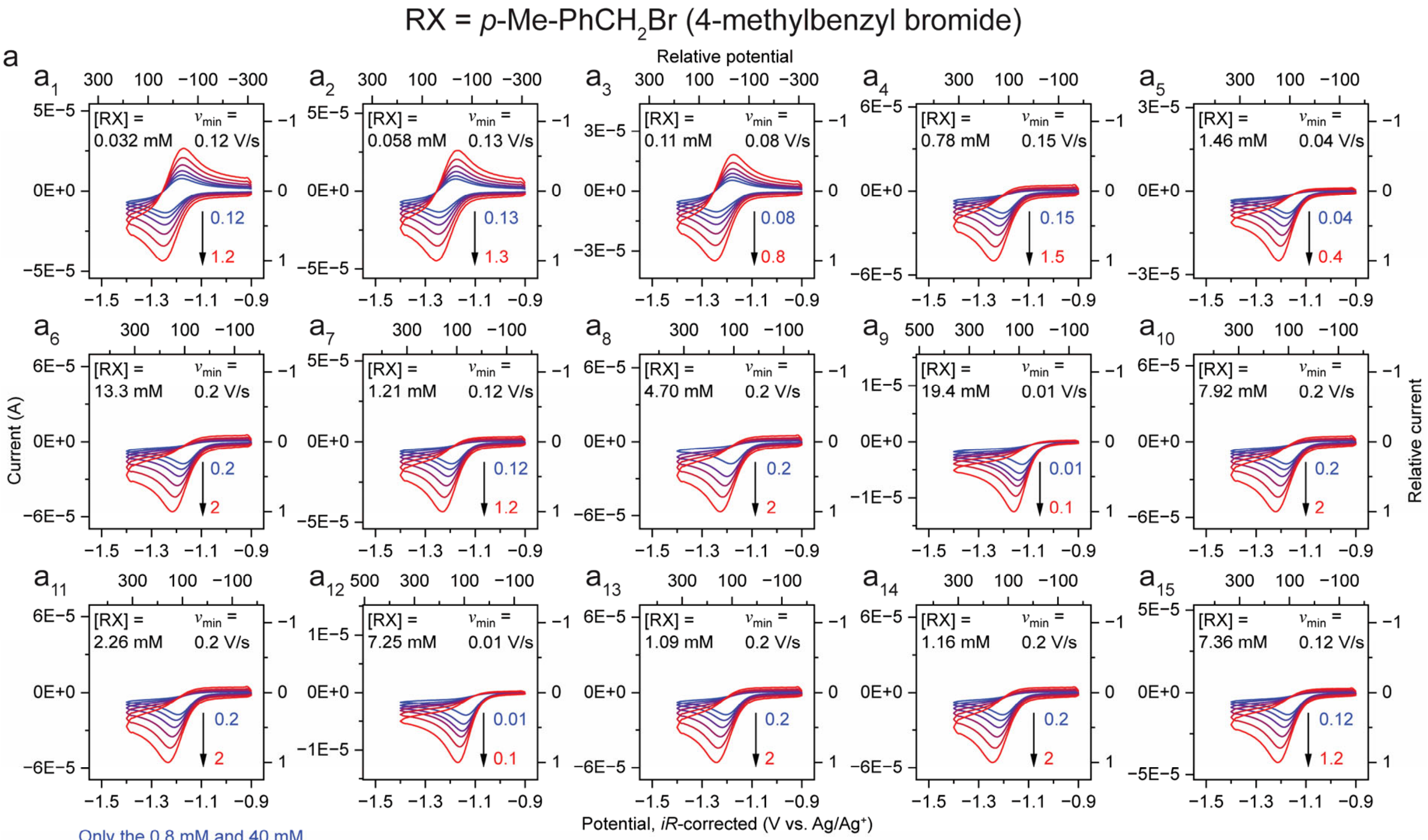

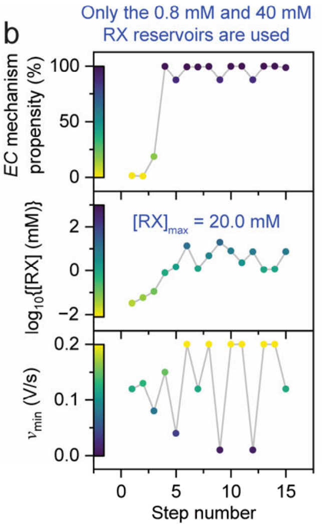

c

Potential range: between −0.9 and −1.4 V vs. Ag/Ag⁺							
Step	[RX] (mM)	v_{min} (V/s)	Mechanism propensity (%)				
			E	*EC*	*CE*	*DISP1*	*ECE*
1	0.032	0.12	98.6	1.4	0.0	0.0	0.0
2	0.058	0.13	99.0	1.0	0.0	0.0	0.0
3	0.11	0.08	81.4	18.6	0.0	0.0	0.0
4	0.78	0.15	0.2	99.8	0.0	0.0	0.0
5	1.46	0.04	12.6	87.4	0.0	0.0	0.0
6	13.3	0.2	0.6	99.4	0.0	0.0	0.0
7	1.21	0.12	0.7	99.3	0.0	0.0	0.0
8	4.70	0.2	0.3	99.7	0.0	0.0	0.0
9	19.4	0.01	12.5	87.5	0.0	0.0	0.0
10	7.92	0.2	0.2	99.8	0.0	0.0	0.0
11	2.26	0.2	0.2	99.8	0.0	0.0	0.0
12	7.25	0.01	12.5	87.5	0.0	0.0	0.0
13	1.09	0.2	0.1	99.9	0.0	0.0	0.0
14	1.16	0.2	0.1	99.9	0.0	0.0	0.0
15	7.36	0.12	1.3	98.7	0.0	0.0	0.0

d

$[RX]_{cutoff}$ = 14.603 mM		
CV data that satisfy i_{pa}/i_{pc} ∈ [0.65, 0.75] from Stage I of the workflow		
[RX] (mM)	$\log_{10}${[RX] (mM)}	v (V/s) that satisfy i_{pa}/i_{pc} ∈ [0.65, 0.75]
None	None	None
CV data that satisfy i_{pa}/i_{pc} ∈ [0.65, 0.75] from Stage II of the workflow		
[RX] (mM)	$\log_{10}${[RX] (mM)}	v (V/s) that satisfy i_{pa}/i_{pc} ∈ [0.65, 0.75]
0.27	−0.576	0.132, 0.157, 0.183, 0.208, 0.234, 0.259

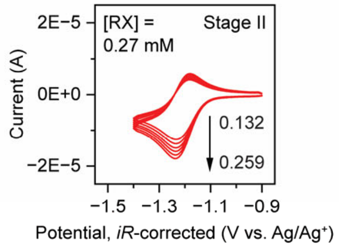

Supplementary Fig. 51. Autonomous investigation of *p*-Me-PhCH₂Br (4-methylbenzyl bromide) that reacts with CoTPP following an *EC* mechanism. (a) CV data (1 mM Co^{II}TPP in DMF with 0.1 M NBu_4PF_6, [RX] ∈ [0.008, 20] mM because only the 0.8 mM and 40 mM RX reservoirs are used, v_{min} ∈ [0.01, 0.2] V/s) measured from Stage I, with detailed parameters and Bayesian optimization results summarized in (b) and (c). (d) Desired combinations of [RX] and v that satisfy i_{pa}/i_{pc} ∈ [0.65, 0.75] from Stage I and Stage II. There seems to be usable CV data under only one [RX] value, insufficient for determining k_0 of the *C* step using the i_{pa}/i_{pc} approach. Also, the corresponding CV responses in (d) may no longer fulfill a pseudo-first-order condition assuming RX is in large excess, meaning the i_{pa}/i_{pc} approach may be invalid to use. Instead, k_0 of the *C* step is determined by the E_{pc} approach (Supplementary Note 10) based on the selected CV data measured at the lowest v value (0.01 V/s) in the parameter space from Stage I and Stage II (Supplementary Figs. 59c and 60c).

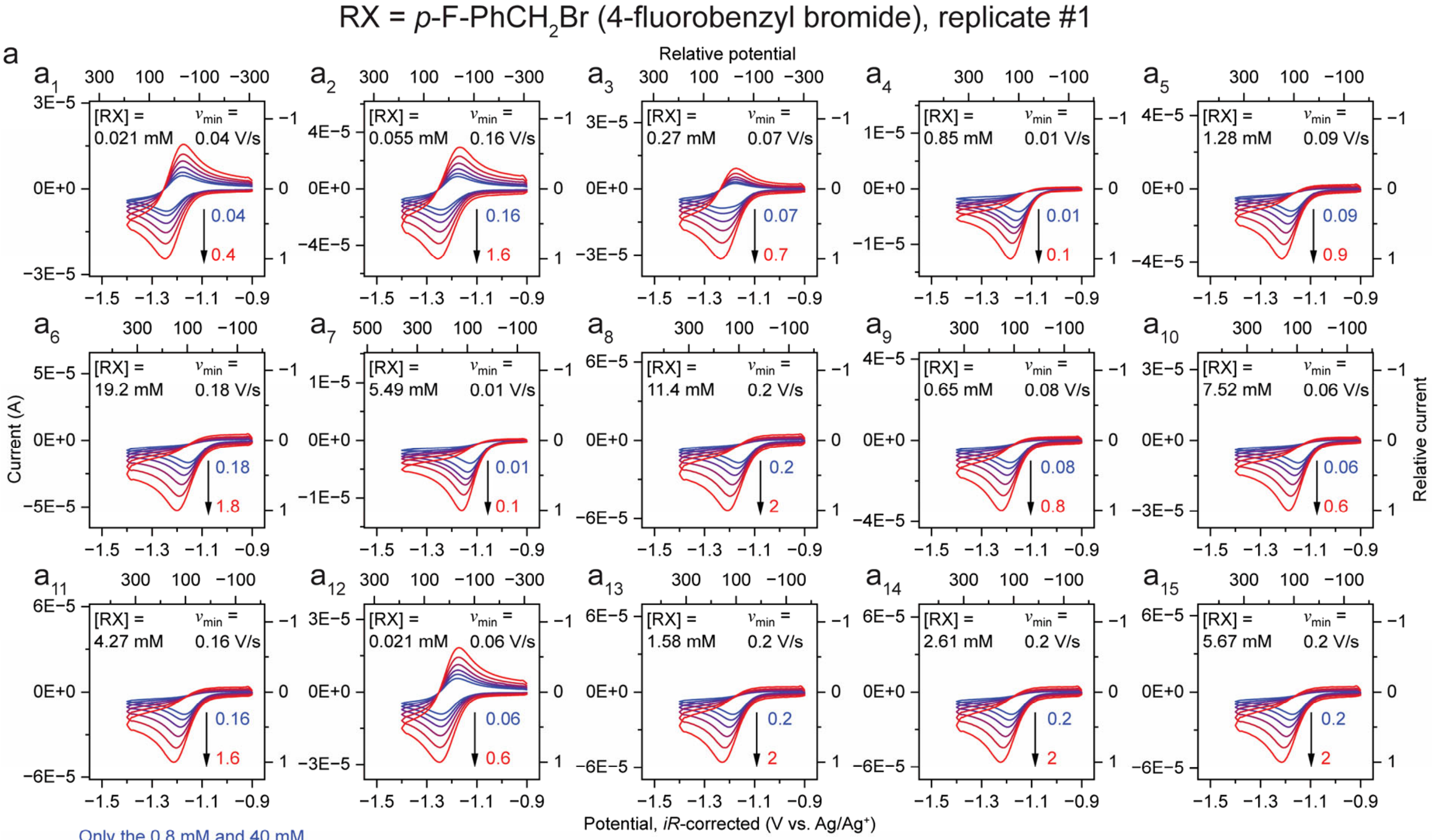

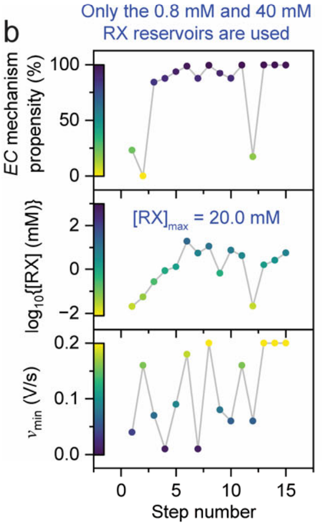

c

Potential range: between −0.9 and −1.4 V vs. Ag/Ag⁺							
Step	[RX] (mM)	v_{min} (V/s)	Mechanism propensity (%)				
			E	*EC*	*CE*	*DISP1*	*ECE*
1	0.021	0.04	77.0	23.0	0.0	0.0	0.0
2	0.055	0.16	100.0	0.0	0.0	0.0	0.0
3	0.27	0.07	15.9	84.1	0.0	0.0	0.0
4	0.85	0.01	12.5	87.5	0.0	0.0	0.0
5	1.28	0.09	6.4	93.6	0.0	0.0	0.0
6	19.2	0.18	1.3	98.7	0.0	0.0	0.0
7	5.49	0.01	12.5	87.5	0.0	0.0	0.0
8	11.4	0.2	0.5	99.5	0.0	0.0	0.0
9	0.65	0.08	7.8	92.2	0.0	0.0	0.0
10	7.52	0.06	12.4	87.6	0.0	0.0	0.0
11	4.27	0.16	0.3	99.7	0.0	0.0	0.0
12	0.021	0.06	82.8	17.2	0.0	0.2	0.0
13	1.58	0.2	0.2	99.8	0.0	0.0	0.0
14	2.61	0.2	0.2	99.8	0.0	0.0	0.0
15	5.67	0.2	0.3	99.7	0.0	0.0	0.0

d

$[RX]_{cutoff}$ = 2.693 mM		
CV data that satisfy i_{pa}/i_{pc} ∈ [0.65, 0.75] from Stage I of the workflow		
[RX] (mM)	$\log_{10}${[RX] (mM)}	v (V/s) that satisfy i_{pa}/i_{pc} ∈ [0.65, 0.75]
None	None	None
CV data that satisfy i_{pa}/i_{pc} ∈ [0.65, 0.75] from Stage II of the workflow		
[RX] (mM)	$\log_{10}${[RX] (mM)}	v (V/s) that satisfy i_{pa}/i_{pc} ∈ [0.65, 0.75]
0.19	−0.713	0.804, 0.884, 0.965, 1.05, 1.13, 1.21

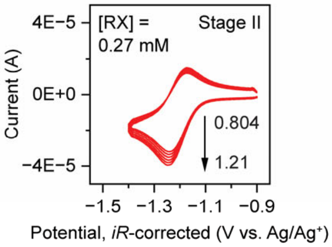

Supplementary Fig. 52. Autonomous investigation of *p*-F-PhCH₂Br (4-fluorobenzyl bromide) that reacts with CoTPP following an *EC* mechanism (replicate #1). (a) CV data (1 mM Co^{II}TPP in DMF with 0.1 M NBu_4PF_6, [RX] ∈ [0.008, 20] mM because only the 0.8 mM and 40 mM RX reservoirs are used, v_{min} ∈ [0.01, 0.2] V/s) measured from Stage I, with detailed parameters and Bayesian optimization results summarized in (b) and (c). (d) Desired combinations of [RX] and v that satisfy i_{pa}/i_{pc} ∈ [0.65, 0.75] from Stage I and Stage II. There seems to be usable CV data under only one [RX] value, insufficient for determining k_0 of the *C* step using the i_{pa}/i_{pc} approach. Also, the corresponding CV responses in (d) may no longer fulfill a pseudo-first-order condition assuming RX is in large excess, meaning the i_{pa}/i_{pc} approach may be invalid to use. Instead, k_0 of the *C* step is determined by the E_{pc} approach (Supplementary Note 10) based on the selected CV data measured at the lowest v value (0.01 V/s) in the parameter space from Stage I and Stage II (Supplementary Figs. 59d and 60d).

RX = *p*-F-PhCH$_2$Br (4-fluorobenzyl bromide), replicate #2

a

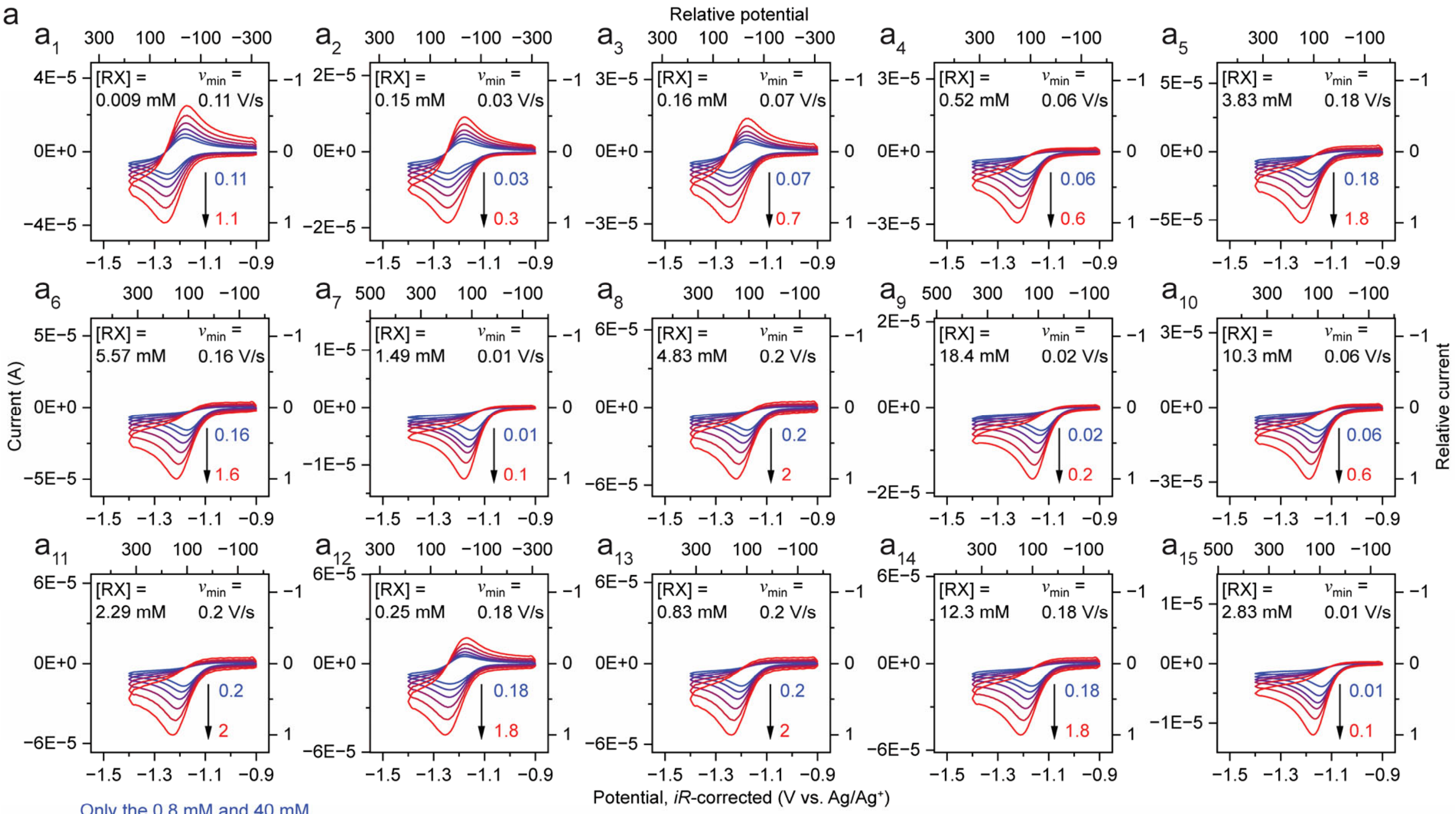

b

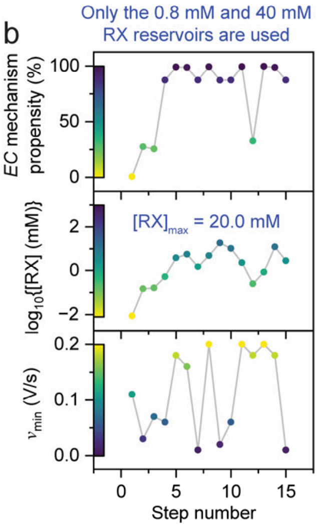

c

Potential range: between −0.9 and −1.4 V vs. Ag/Ag⁺							
Step	[RX] (mM)	v_{min} (V/s)	Mechanism propensity (%)				
			E	*EC*	*CE*	*DISP1*	*ECE*
1	0.009	0.11	99.5	0.5	0.0	0.0	0.0
2	0.15	0.03	72.6	27.4	0.0	0.0	0.0
3	0.16	0.07	74.6	25.4	0.0	0.0	0.0
4	0.52	0.06	12.6	87.4	0.0	0.0	0.0
5	3.83	0.18	0.9	99.1	0.0	0.0	0.0
6	5.57	0.16	1.2	98.8	0.0	0.0	0.0
7	1.49	0.01	12.6	87.4	0.0	0.0	0.0
8	4.83	0.2	0.7	99.3	0.0	0.0	0.0
9	18.4	0.02	12.7	87.3	0.0	0.0	0.0
10	10.3	0.06	12.6	87.4	0.0	0.0	0.0
11	2.29	0.2	0.5	99.5	0.0	0.0	0.0
12	0.25	0.18	67.3	32.5	0.0	0.2	0.0
13	0.83	0.2	0.3	99.7	0.0	0.0	0.0
14	12.3	0.18	1.2	98.8	0.0	0.0	0.0
15	2.83	0.01	12.6	87.4	0.0	0.0	0.0

d

$[RX]_{cutoff}$ = 12.978 mM		
CV data that satisfy i_{pa}/i_{pc} ∈ [0.65, 0.75] from Stage I of the workflow		
[RX] (mM)	log_{10}{[RX] (mM)}	*v* (V/s) that satisfy i_{pa}/i_{pc} ∈ [0.65, 0.75]
0.25	−0.610	0.18, 0.29, 0.45, 0.72, 1.14, 1.8
CV data that satisfy i_{pa}/i_{pc} ∈ [0.65, 0.75] from Stage II of the workflow		
[RX] (mM)	log_{10}{[RX] (mM)}	*v* (V/s) that satisfy i_{pa}/i_{pc} ∈ [0.65, 0.75]
None	None	None

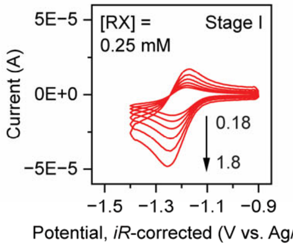

Supplementary Fig. 53. Autonomous investigation of *p*-F-PhCH$_2$Br (4-fluorobenzyl bromide) that reacts with CoTPP following an *EC* mechanism (replicate #2). (a) CV data (1 mM Co^{II}TPP in DMF with 0.1 M NBu$_4$PF$_6$, [RX] ∈ [0.008, 20] mM because only the 0.8 mM and 40 mM RX reservoirs are used, v_{min} ∈ [0.01, 0.2] V/s) measured from Stage I, with detailed parameters and Bayesian optimization results summarized in (b) and (c). (d) Desired combinations of [RX] and *v* that satisfy i_{pa}/i_{pc} ∈ [0.65, 0.75] from Stage I and Stage II. There seems to be usable CV data under only one [RX] value, insufficient for determining k_0 of the *C* step using the i_{pa}/i_{pc} approach. Also, the corresponding CV responses in (d) may no longer fulfill a pseudo-first-order condition assuming RX is in large excess, meaning the i_{pa}/i_{pc} approach may be invalid to use. Instead, k_0 of the *C* step is determined by the E_{pc} approach (Supplementary Note 10) based on the selected CV data measured at the lowest *v* value (0.01 V/s) in the parameter space from Stage I and Stage II (Supplementary Figs. 59e and 60e).

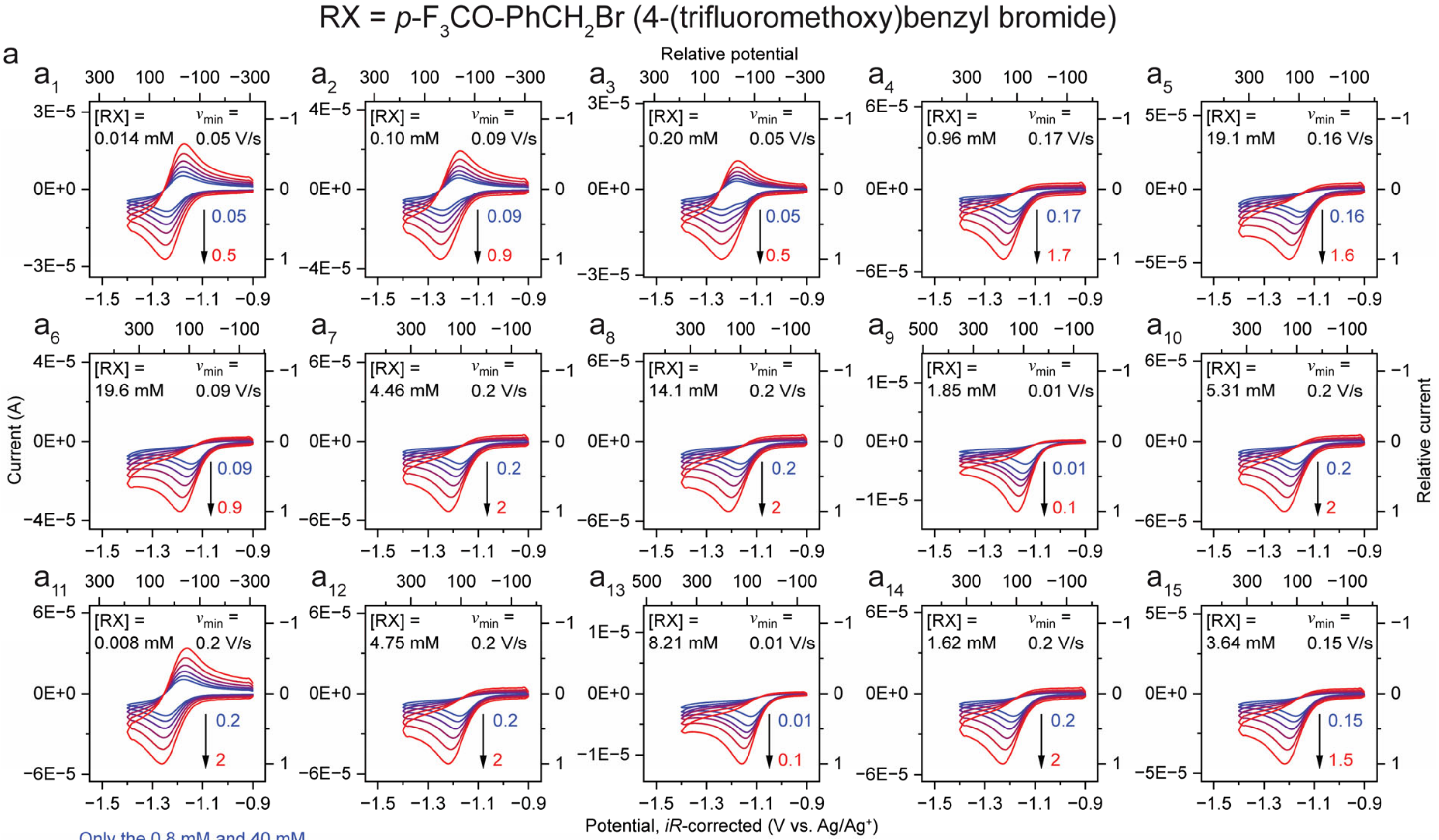

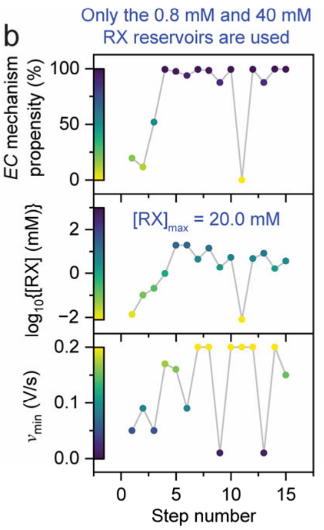

c

Potential range: between −0.9 and −1.4 V vs. Ag/Ag⁺							
Step	[RX] (mM)	v_{min} (V/s)	Mechanism propensity (%)				
			E	*EC*	*CE*	*DISP1*	*ECE*
1	0.014	0.05	80.6	19.4	0.0	0.0	0.0
2	0.10	0.09	88.5	11.5	0.0	0.0	0.0
3	0.20	0.05	48.0	52.0	0.0	0.0	0.0
4	0.96	0.17	0.6	99.4	0.0	0.0	0.0
5	19.1	0.16	2.5	97.5	0.0	0.0	0.0
6	19.6	0.09	6.3	93.7	0.0	0.0	0.0
7	4.46	0.2	0.6	99.4	0.0	0.0	0.0
8	14.1	0.2	1.5	98.5	0.0	0.0	0.0
9	1.85	0.01	12.6	87.4	0.0	0.0	0.0
10	5.31	0.2	0.6	99.4	0.0	0.0	0.0
11	0.008	0.2	100.0	0.0	0.0	0.0	0.0
12	4.75	0.2	0.6	99.4	0.0	0.0	0.0
13	8.21	0.01	12.6	87.4	0.0	0.0	0.0
14	1.62	0.2	0.4	99.6	0.0	0.0	0.0
15	3.64	0.15	0.5	99.5	0.0	0.0	0.0

d

$[RX]_{cutoff}$ = 3.151 mM		
CV data that satisfy $i_{pa}/i_{pc} \in [0.65, 0.75]$ from Stage I of the workflow		
[RX] (mM)	$\log_{10}$\{[RX] (mM)\}	v (V/s) that satisfy $i_{pa}/i_{pc} \in [0.65, 0.75]$
0.20	−0.689	0.05, 0.08, 0.13, 0.2, 0.32, 0.5
CV data that satisfy $i_{pa}/i_{pc} \in [0.65, 0.75]$ from Stage II of the workflow		
[RX] (mM)	$\log_{10}$\{[RX] (mM)\}	v (V/s) that satisfy $i_{pa}/i_{pc} \in [0.65, 0.75]$
None	None	None

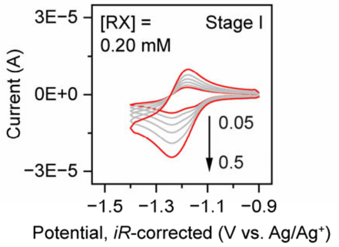

Supplementary Fig. 54. Autonomous investigation of *p*-F_3CO-$PhCH_2Br$ (4-(trifluoromethoxy)benzyl bromide) that reacts with CoTPP following an *EC* mechanism. (a) CV data (1 mM Co^{II}TPP in DMF with 0.1 M NBu_4PF_6, [RX] ∈ [0.008, 20] mM because only the 0.8 mM and 40 mM RX reservoirs are used, v_{min} ∈ [0.01, 0.2] V/s) measured from Stage I, with detailed parameters and Bayesian optimization results summarized in (b) and (c). (d) Desired combinations of [RX] and v that satisfy i_{pa}/i_{pc} ∈ [0.65, 0.75] from Stage I and Stage II. There seems to be usable CV data under only one [RX] value, insufficient for determining k_0 of the *C* step using the i_{pa}/i_{pc} approach. Also, the corresponding CV responses in (d) may no longer fulfill a pseudo-first-order condition assuming RX is in large excess, meaning the i_{pa}/i_{pc} approach may be invalid to use. Instead, k_0 of the *C* step is determined by the E_{pc} approach (Supplementary Note 10) based on the selected CV data measured at the lowest v value (0.01 V/s) in the parameter space from Stage I and Stage II (Supplementary Figs. 59h and 60h).

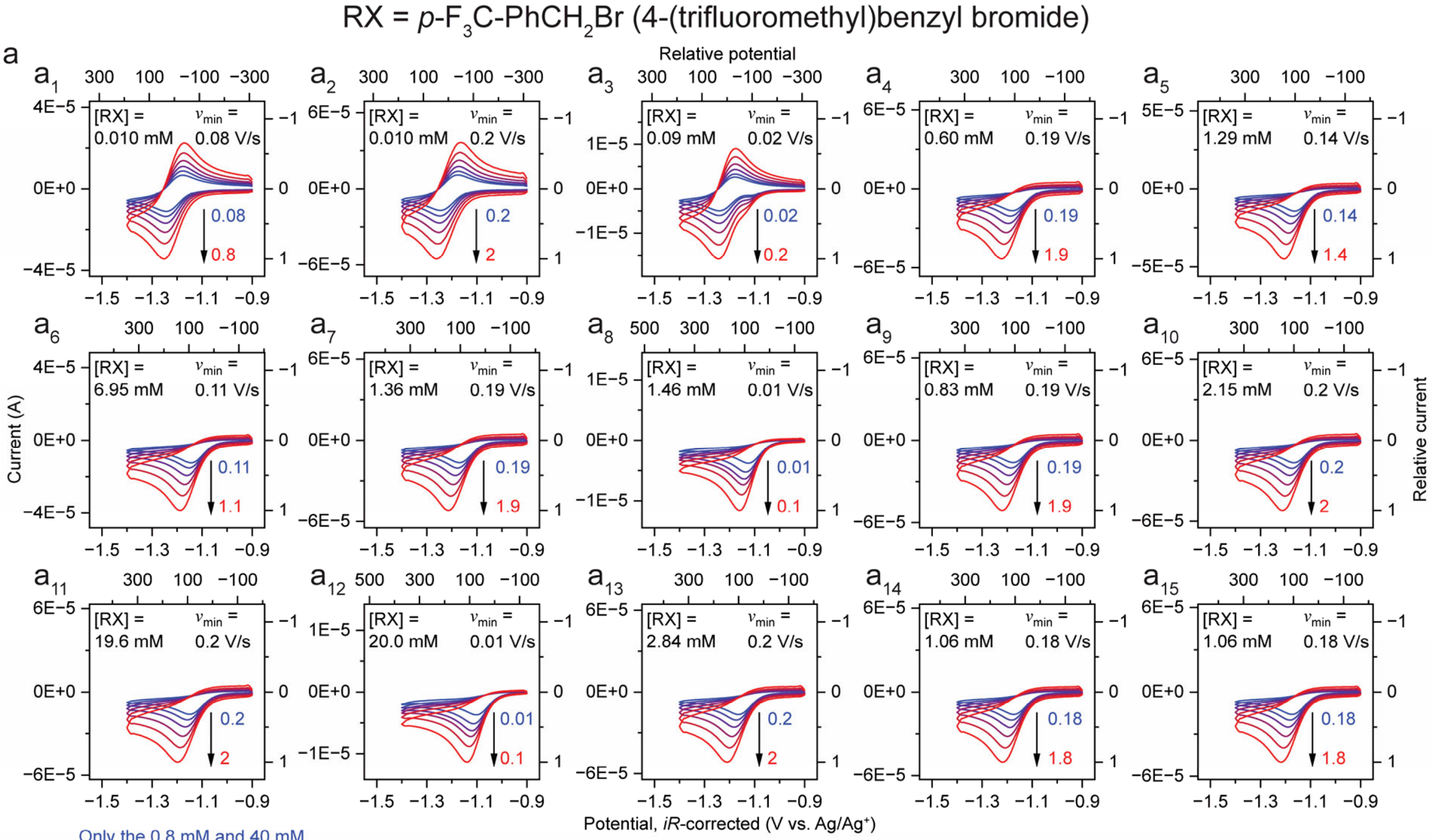

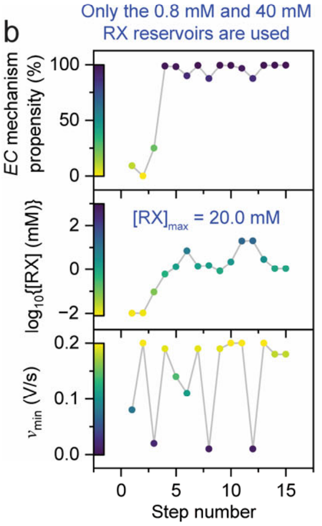

c

Potential range: between −0.9 and −1.4 V vs. Ag/Ag+							
Step	[RX] (mM)	v_{min} (V/s)	Mechanism propensity (%)				
			E	*EC*	*CE*	*DISP1*	*ECE*
1	0.010	0.08	90.9	9.1	0.0	0.0	0.0
2	0.010	0.2	100.0	0.0	0.0	0.0	0.0
3	0.09	0.02	75.1	24.9	0.0	0.0	0.0
4	0.60	0.19	1.2	98.8	0.0	0.0	0.0
5	1.29	0.14	1.8	98.2	0.0	0.0	0.0
6	6.95	0.11	10.2	89.8	0.0	0.0	0.0
7	1.36	0.19	0.7	99.3	0.0	0.0	0.0
8	1.46	0.01	12.6	87.4	0.0	0.0	0.0
9	0.83	0.19	0.6	99.4	0.0	0.0	0.0
10	2.15	0.2	0.6	99.3	0.0	0.0	0.0
11	19.6	0.2	3.4	96.6	0.0	0.0	0.0
12	20.0	0.01	12.6	87.4	0.0	0.0	0.0
13	2.84	0.2	0.5	99.5	0.0	0.0	0.0
14	1.06	0.18	0.4	99.6	0.0	0.0	0.0
15	1.06	0.18	0.4	99.6	0.0	0.0	0.0

d

$[RX]_{cutoff}$ = 3.03 mM		
CV data that satisfy i_{pa}/i_{pc} ∈ [0.65, 0.75] from Stage I of the workflow		
[RX] (mM)	$\log_{10}$\{[RX] (mM)\}	v (V/s) that satisfy i_{pa}/i_{pc} ∈ [0.65, 0.75]
None	None	None
CV data that satisfy i_{pa}/i_{pc} ∈ [0.65, 0.75] from Stage II of the workflow		
[RX] (mM)	$\log_{10}$\{[RX] (mM)\}	v (V/s) that satisfy i_{pa}/i_{pc} ∈ [0.65, 0.75]
0.22	−0.655	0.804, 0.884, 0.965, 1.05, 1.13, 1.21

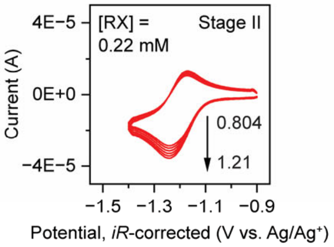

Supplementary Fig. 55. Autonomous investigation of *p*-F_3C-$PhCH_2Br$ (4-trifluoromethylbenzyl bromide) that reacts with CoTPP following an *EC* mechanism. (a) CV data (1 mM Co^{II}TPP in DMF with 0.1 M NBu_4PF_6, [RX] ∈ [0.008, 20] mM because only the 0.8 mM and 40 mM RX reservoirs are used, v_{min} ∈ [0.01, 0.2] V/s) measured from Stage I, with detailed parameters and Bayesian optimization results summarized in (b) and (c). (d) Desired combinations of [RX] and v that satisfy i_{pa}/i_{pc} ∈ [0.65, 0.75] from Stage I and Stage II. There seems to be usable CV data under only one [RX] value, insufficient for determining k_0 of the *C* step using the i_{pa}/i_{pc} approach. Also, the corresponding CV responses in (d) may no longer fulfill a pseudo-first-order condition assuming RX is in large excess, meaning the i_{pa}/i_{pc} approach may be invalid to use. Instead, k_0 of the *C* step is determined by the E_{pc} approach (Supplementary Note 10) based on the selected CV data measured at the lowest v value (0.01 V/s) in the parameter space from Stage I and Stage II (Supplementary Figs. 59i and 60i).

RX = *p*-MeO$_2$C-PhCH$_2$Br (methyl 4-(bromomethyl)benzoate)

a

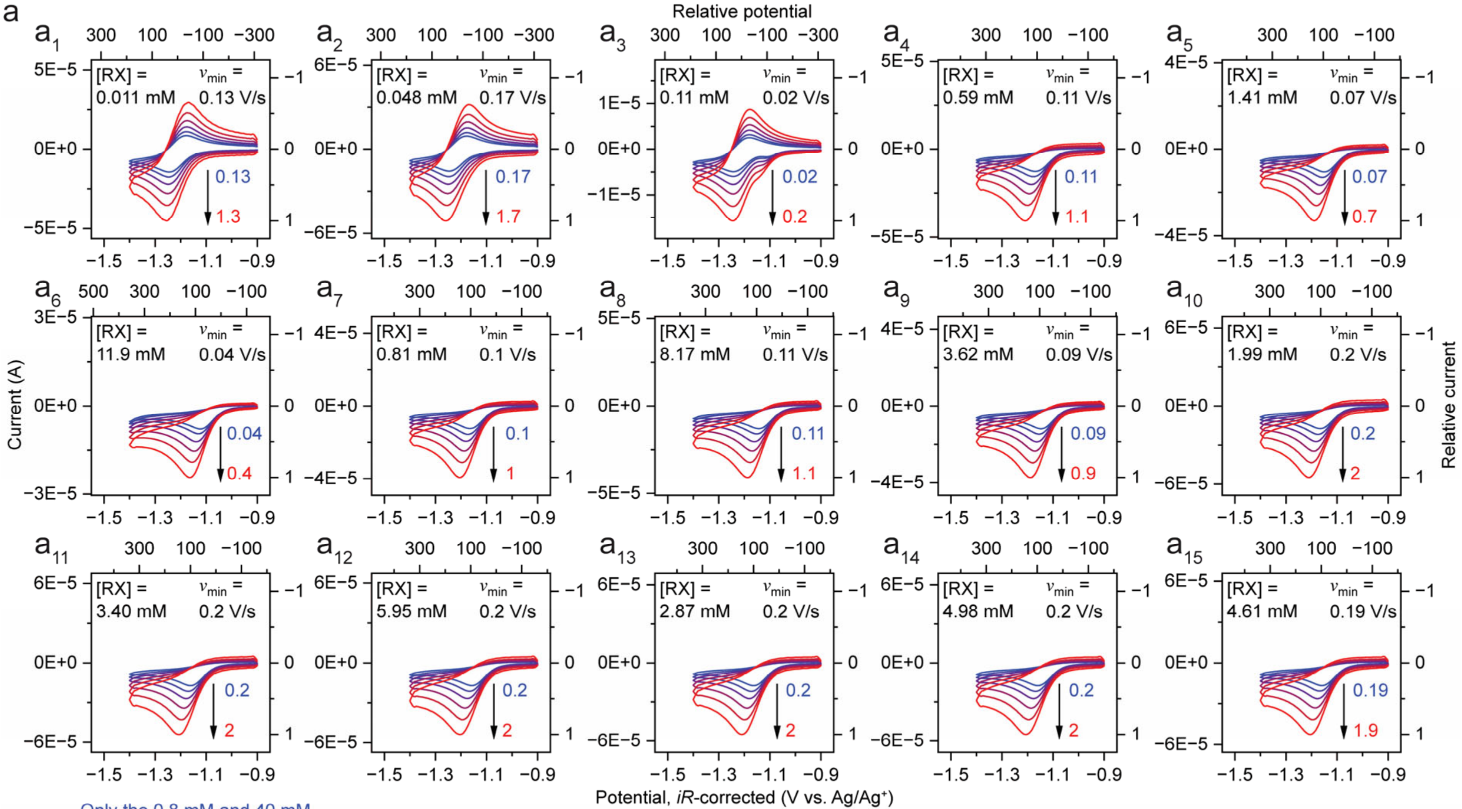

b

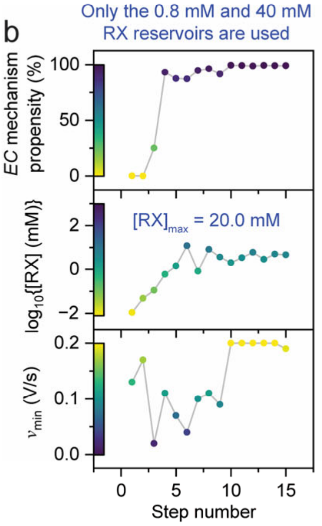

c

Potential range: between −0.9 and −1.4 V vs. Ag/Ag$^+$

Step	[RX] (mM)	v_{min} (V/s)	Mechanism propensity (%)				
			E	*EC*	*CE*	*DISP1*	*ECE*
1	0.011	0.13	99.9	0.1	0.0	0.0	0.0
2	0.048	0.17	100.0	0.0	0.0	0.0	0.0
3	0.11	0.02	75.0	25.0	0.0	0.0	0.0
4	0.59	0.11	6.8	93.2	0.0	0.0	0.0
5	1.41	0.07	12.5	87.5	0.0	0.0	0.0
6	11.9	0.04	12.8	87.2	0.0	0.0	0.0
7	0.81	0.1	5.3	94.7	0.0	0.0	0.0
8	8.17	0.11	3.7	96.3	0.0	0.0	0.0
9	3.62	0.09	8.4	91.6	0.0	0.0	0.0
10	1.99	0.2	0.5	99.5	0.0	0.0	0.0
11	3.40	0.2	0.8	99.2	0.0	0.0	0.0
12	5.95	0.2	1.2	98.8	0.0	0.0	0.0
13	2.87	0.2	0.6	99.4	0.0	0.0	0.0
14	4.98	0.2	0.9	99.1	0.0	0.0	0.0
15	4.61	0.19	0.9	99.1	0.0	0.0	0.0

d

$[RX]_{cutoff}$ = 3.278 mM		
CV data that satisfy $i_{pa}/i_{pc} \in [0.65, 0.75]$ from Stage I of the workflow		
[RX] (mM)	log_{10}{[RX] (mM)}	*v* (V/s) that satisfy $i_{pa}/i_{pc} \in [0.65, 0.75]$
None	None	None
CV data that satisfy $i_{pa}/i_{pc} \in [0.65, 0.75]$ from Stage II of the workflow		
[RX] (mM)	log_{10}{[RX] (mM)}	*v* (V/s) that satisfy $i_{pa}/i_{pc} \in [0.65, 0.75]$
0.34	−0.469	1.14, 1.25, 1.37, 1.48, 1.59, 1.71

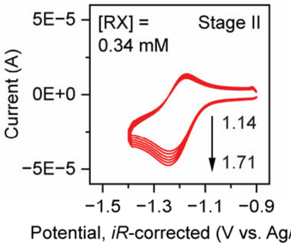

Supplementary Fig. 56. Autonomous investigation of *p*-MeO$_2$C-PhCH$_2$Br (methyl 4-(bromomethyl)benzoate) that reacts with CoTPP following an *EC* mechanism. (a) CV data (1 mM Co^{II}TPP in DMF with 0.1 M NBu$_4$PF$_6$, [RX] ∈ [0.008, 20] mM because only the 0.8 mM and 40 mM RX reservoirs are used, v_{min} ∈ [0.01, 0.2] V/s) measured from Stage I, with detailed parameters and Bayesian optimization results summarized in (b) and (c). (d) Desired combinations of [RX] and *v* that satisfy i_{pa}/i_{pc} ∈ [0.65, 0.75] from Stage I and Stage II. There seems to be usable CV data under only one [RX] value, insufficient for determining k_0 of the *C* step using the i_{pa}/i_{pc} approach. Also, the corresponding CV responses in (d) may no longer fulfill a pseudo-first-order condition assuming RX is in large excess, meaning the i_{pa}/i_{pc} approach may be invalid to use. Instead, k_0 of the *C* step is determined by the E_{pc} approach (Supplementary Note 10) based on the selected CV data measured at the lowest *v* value (0.01 V/s) in the parameter space from Stage I and Stage II (Supplementary Figs. 59j and 60j).

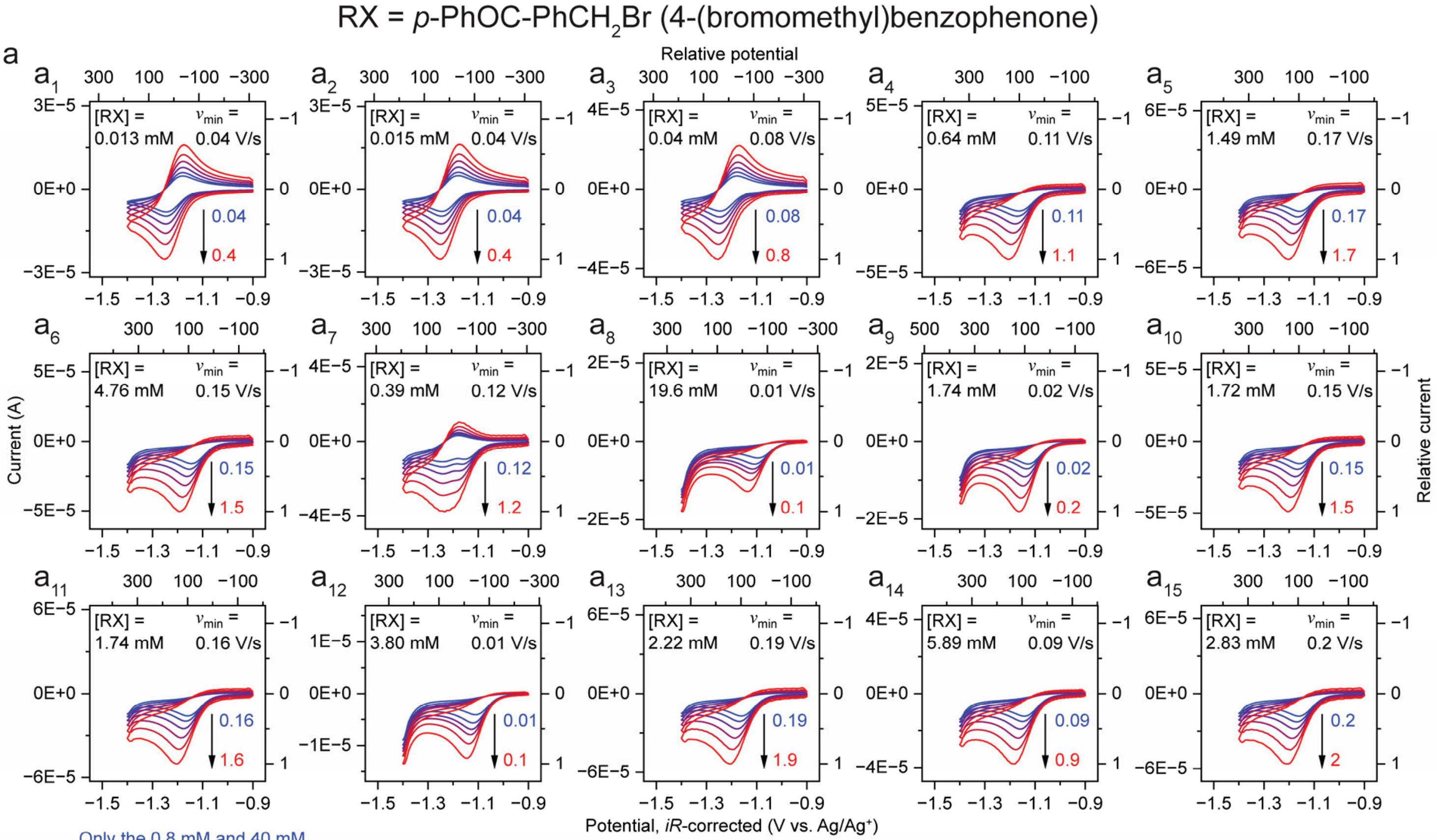

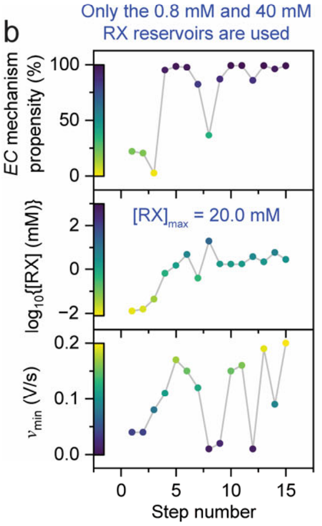

c

Potential range: between −0.9 and −1.4 V vs. Ag/Ag+							
Step	[RX] (mM)	v_{min} (V/s)	Mechanism propensity (%)				
			E	*EC*	*CE*	*DISP1*	*ECE*
1	0.013	0.04	78.1	21.9	0.0	0.0	0.0
2	0.015	0.04	79.6	20.4	0.0	0.0	0.0
3	0.04	0.08	97.5	2.5	0.0	0.0	0.0
4	0.64	0.11	5.0	95.0	0.0	0.0	0.0
5	1.49	0.17	1.5	98.5	0.0	0.0	0.0
6	4.76	0.15	2.4	97.6	0.0	0.0	0.0
7	0.39	0.12	17.8	82.2	0.0	0.0	0.0
8	19.6	0.01	12.5	36.5	0.0	14.7	36.3
9	1.74	0.02	13.2	86.8	0.0	0.0	0.0
10	1.72	0.15	0.7	99.2	0.0	0.0	0.0
11	1.74	0.16	0.7	99.3	0.0	0.0	0.0
12	3.80	0.01	14.2	85.8	0.0	0.0	0.0
13	2.22	0.19	0.9	99.1	0.0	0.0	0.0
14	5.89	0.09	4.0	96.0	0.0	0.0	0.0
15	2.83	0.2	0.9	99.1	0.0	0.0	0.0

d

$[RX]_{cutoff}$ = 3.836 mM		
CV data that satisfy i_{pa}/i_{pc} ∈ [0.65, 0.75] from Stage I of the workflow		
[RX] (mM)	log_{10}{[RX] (mM)}	v (V/s) that satisfy i_{pa}/i_{pc} ∈ [0.65, 0.75]
0.39	−0.407	0.12, 0.19, 0.3, 0.48, 0.76, 1.2
CV data that satisfy i_{pa}/i_{pc} ∈ [0.65, 0.75] from Stage II of the workflow		
[RX] (mM)	log_{10}{[RX] (mM)}	v (V/s) that satisfy i_{pa}/i_{pc} ∈ [0.65, 0.75]
None	None	None

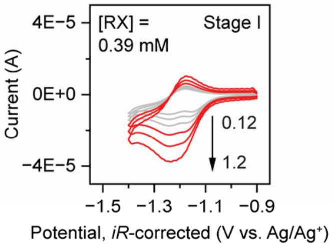

Supplementary Fig. 57. Autonomous investigation of *p*-PhOC-PhCH$_2$Br (4-(bromomethyl)benzophenone) that reacts with CoTPP following an *EC* mechanism. (a) CV data (1 mM Co^{II}TPP in DMF with 0.1 M NBu$_4$PF$_6$, [RX] ∈ [0.008, 20] mM because only the 0.8 mM and 40 mM RX reservoirs are used, v_{min} ∈ [0.01, 0.2] V/s) measured from Stage I, with detailed parameters and Bayesian optimization results summarized in (b) and (c). (d) Desired combinations of [RX] and v that satisfy i_{pa}/i_{pc} ∈ [0.65, 0.75] from Stage I and Stage II. There seems to be usable CV data under only one [RX] value, insufficient for determining k_0 of the *C* step using the i_{pa}/i_{pc} approach. Also, the corresponding CV responses in (d) may no longer fulfill a pseudo-first-order condition assuming RX is in large excess, meaning the i_{pa}/i_{pc} approach may be invalid to use. Instead, k_0 of the *C* step is determined by the E_{pc} approach (Supplementary Note 10) based on the selected CV data measured at the lowest v value (0.01 V/s) in the parameter space from Stage I and Stage II (Supplementary Figs. 59k and 60k).

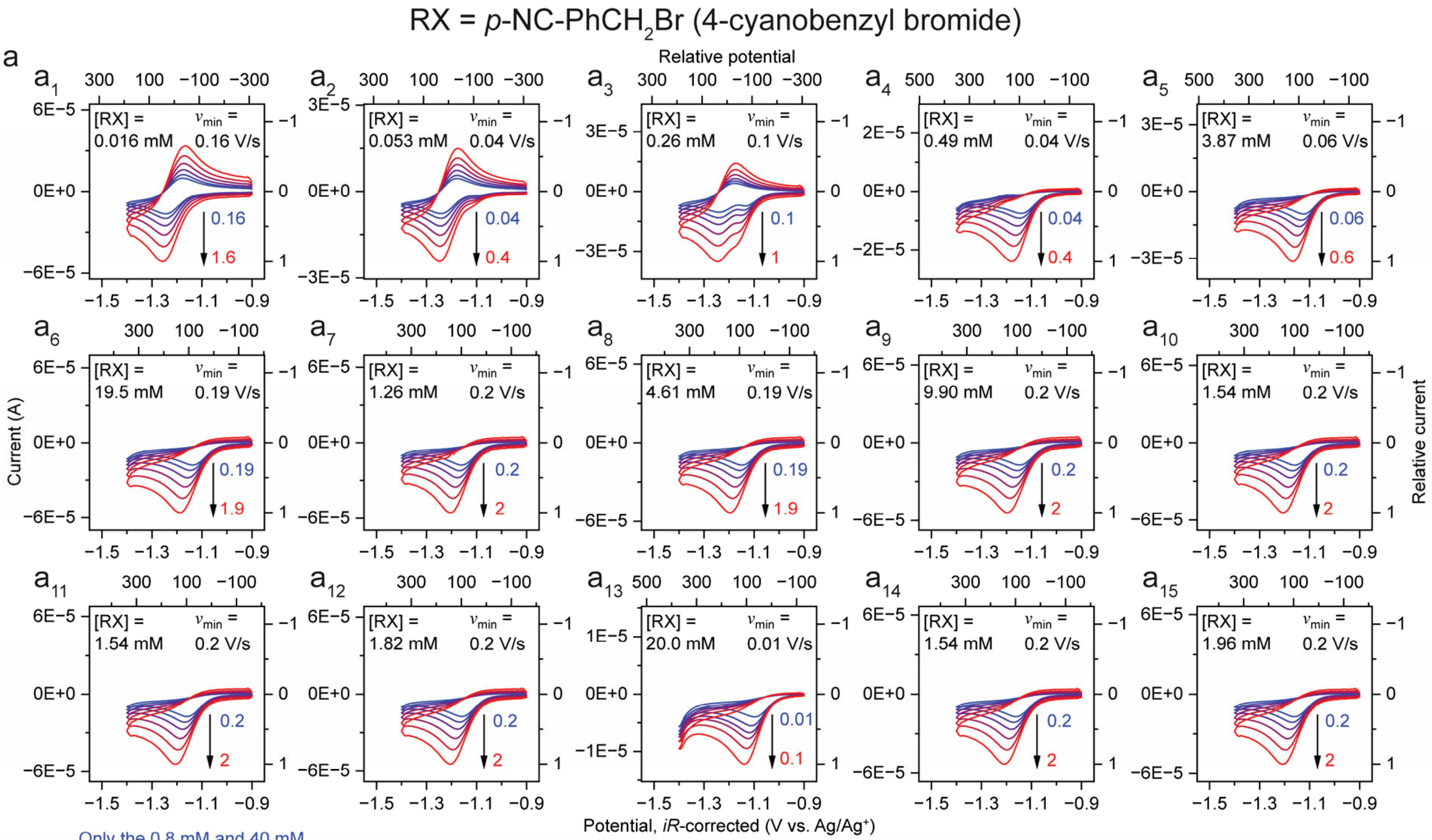

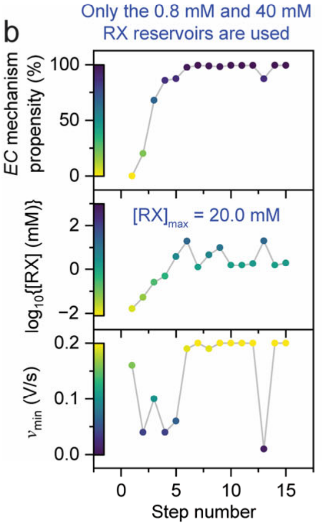

c

Potential range: between −0.9 and −1.4 V vs. Ag/Ag+							
Step	[RX] (mM)	v_{min} (V/s)	Mechanism propensity (%)				
			E	*EC*	*CE*	*DISP1*	*ECE*
1	0.016	0.16	100.0	0.0	0.0	0.0	0.0
2	0.053	0.04	79.9	20.1	0.0	0.0	0.0
3	0.26	0.1	32.0	68.0	0.0	0.0	0.0
4	0.49	0.04	14.3	85.7	0.0	0.0	0.0
5	3.87	0.06	12.7	87.3	0.0	0.0	0.0
6	19.5	0.19	2.4	97.6	0.0	0.0	0.0
7	1.26	0.2	0.5	99.4	0.0	0.0	0.0
8	4.61	0.19	1.1	98.9	0.0	0.0	0.0
9	9.90	0.2	1.7	98.2	0.0	0.0	0.0
10	1.54	0.2	0.6	99.4	0.0	0.0	0.0
11	1.54	0.2	0.6	99.4	0.0	0.0	0.0
12	1.82	0.2	0.6	99.4	0.0	0.0	0.0
13	20.0	0.01	12.8	87.2	0.0	0.0	0.0
14	1.54	0.2	0.4	99.6	0.0	0.0	0.0
15	1.96	0.2	0.4	99.5	0.0	0.0	0.1

d

$[RX]_{cutoff}$ = 3.151 mM		
CV data that satisfy i_{pa}/i_{pc} ∈ [0.65, 0.75] from Stage I of the workflow		
[RX] (mM)	$\log_{10}${[RX] (mM)}	v (V/s) that satisfy i_{pa}/i_{pc} ∈ [0.65, 0.75]
None	None	None
CV data that satisfy i_{pa}/i_{pc} ∈ [0.65, 0.75] from Stage II of the workflow		
[RX] (mM)	$\log_{10}${[RX] (mM)}	v (V/s) that satisfy i_{pa}/i_{pc} ∈ [0.65, 0.75]
None	None	None

Supplementary Fig. 58. Autonomous investigation of *p*-NC-PhCH₂Br (4-cyanobenzyl bromide) that reacts with CoTPP following an *EC* mechanism. (a) CV data (1 mM Co^{II}TPP in DMF with 0.1 M NBu_4PF_6, [RX] ∈ [0.008, 20] mM because only the 0.8 mM and 40 mM RX reservoirs are used, v_{min} ∈ [0.01, 0.2] V/s) measured from Stage I, with detailed parameters and Bayesian optimization results summarized in (b) and (c). (d) Desired combinations of [RX] and v that satisfy i_{pa}/i_{pc} ∈ [0.65, 0.75] from Stage I and Stage II. There is no usable CV data for determining k_0 of the *C* step using the i_{pa}/i_{pc} approach. Instead, k_0 of the *C* step is determined by the E_{pc} approach (Supplementary Note 10) based on the selected CV data measured at the lowest v value (0.01 V/s) in the parameter space from Stage I and Stage II (Supplementary Figs. 59l and 60l).

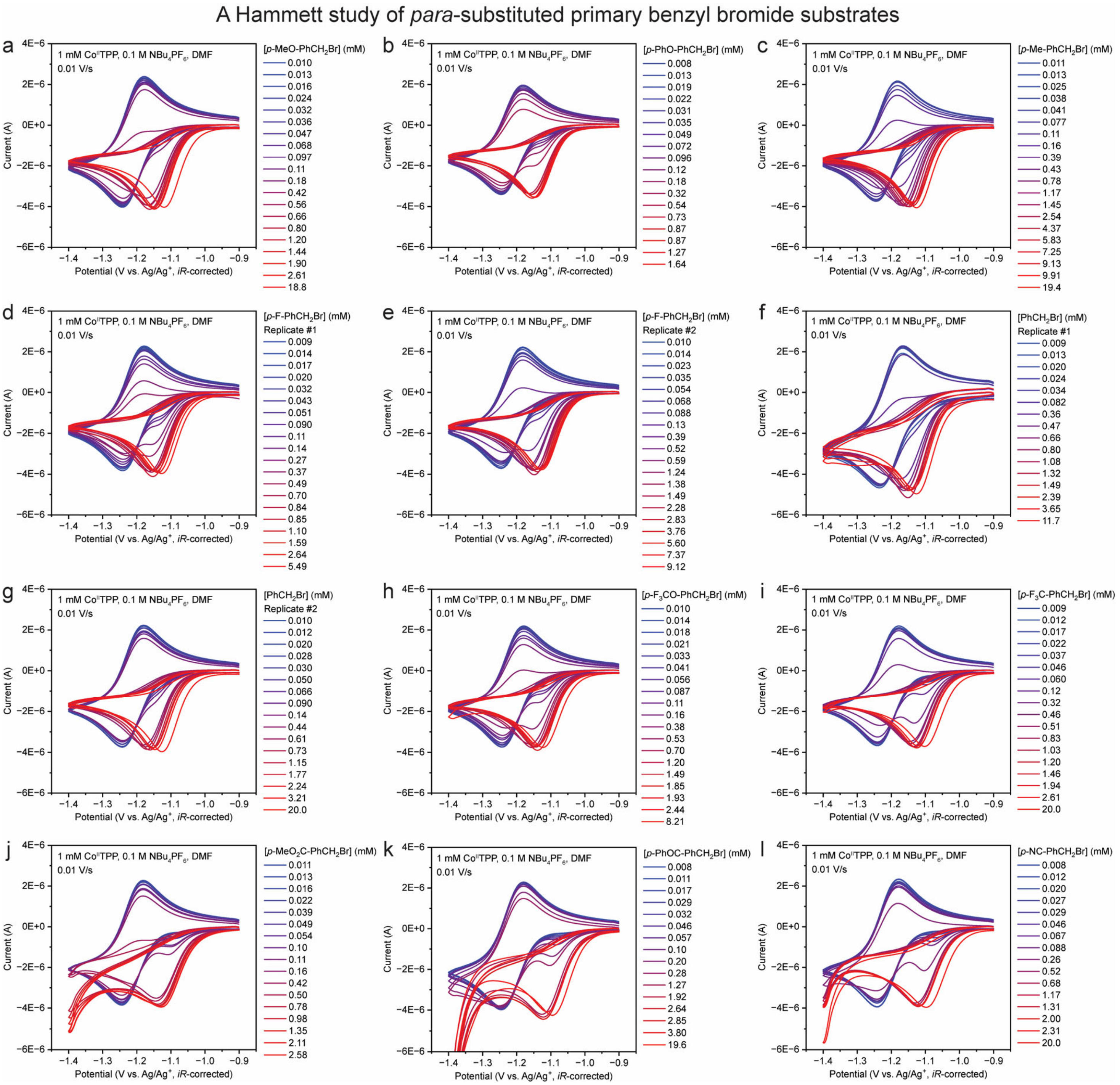

Supplementary Fig. 59. Determination of k_0 for *para*-substituted primary benzyl bromide substrates using the E_{pc} approach (part 1). Selected CV data measured at the lowest *v* value (0.01 V/s) in the parameter space from Stage I and Stage II of the workflow: (a) *p*-MeO-$PhCH_2Br$; (b) *p*-PhO-$PhCH_2Br$; (c) *p*-Me-$PhCH_2Br$; (d) *p*-F-$PhCH_2Br$ (replicate #1); (e) *p*-F-$PhCH_2Br$ (replicate #2); (f) $PhCH_2Br$ (replicate #1); (g) $PhCH_2Br$ (replicate #2); (h) *p*-F_3CO-$PhCH_2Br$; (i) *p*-F_3C-$PhCH_2Br$; (j) *p*-MeO_2C-$PhCH_2Br$; (k) *p*-PhOC-$PhCH_2Br$; (l) *p*-NC-$PhCH_2Br$. Detailed results from the corresponding autonomous investigations of $PhCH_2Br$ and *para*-substituted primary benzyl bromide substrates can be found in Supplementary Figs. 45, 46, and 49–58.

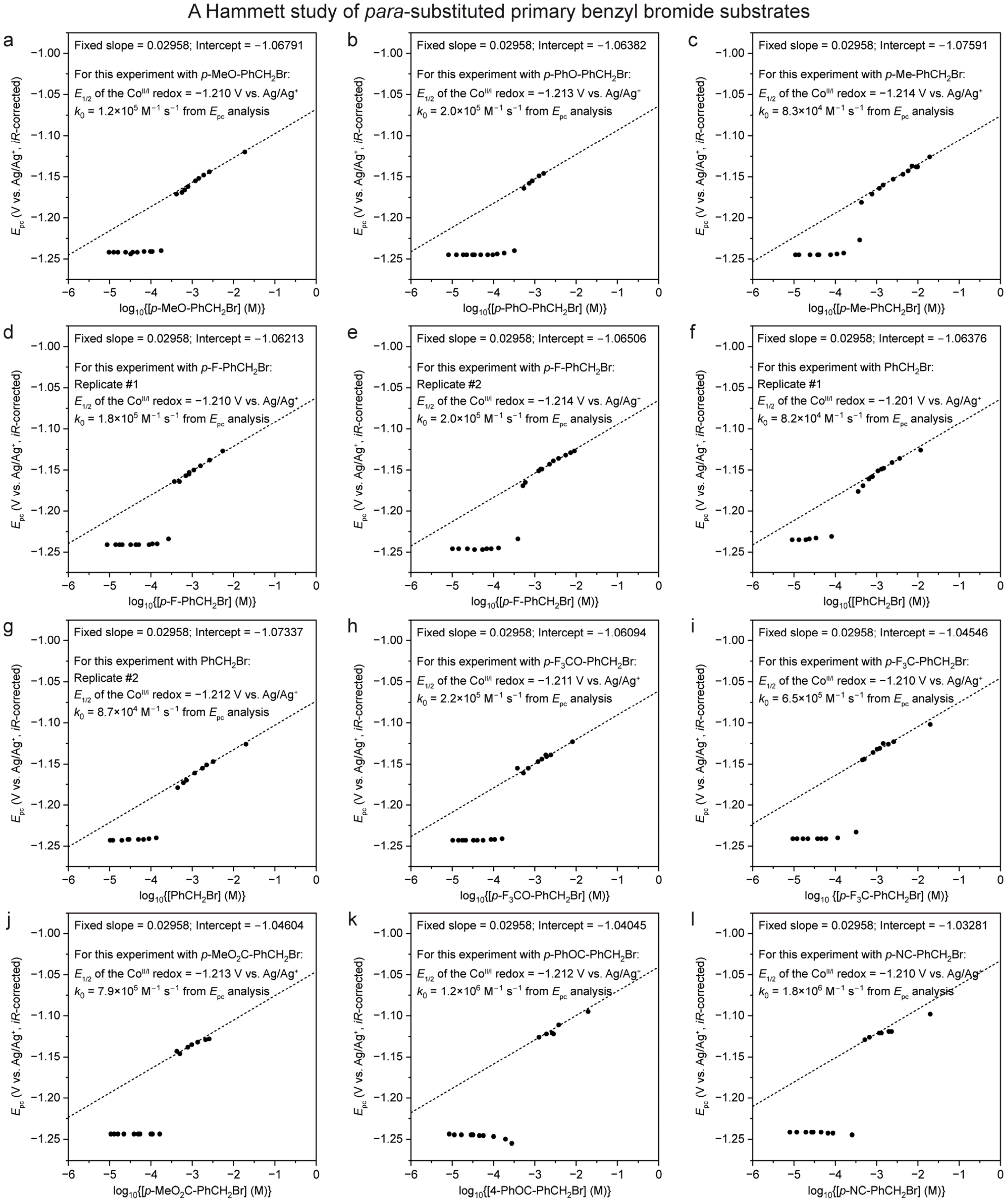

Supplementary Fig. 60. Determination of k_0 for *para*-substituted primary benzyl bromide substrates using the E_{pc} approach (part 2). The resultant E_{pc} versus $\log_{10}$[RX] plots based on the CV data shown in Supplementary Fig. 59.

Supplementary Table 13. Various polar- or radical-derived Hammett parameters for the *para*-substituted primary benzyl bromide substrates studied in this work.

Substituent	σ [1]	σ^+ [1]	σ^- [1]	$\lvert\sigma^\pm\rvert$ [1]	σ_{mb} [1]	$\sigma^\bullet_{JJ}$	$\sigma^\bullet_{J}$	$\sigma^\bullet_{\alpha}$	$\sigma^\bullet_{C}$	$\sigma^\bullet_{F}$	n	$\lvert\sigma^\pm - \sigma\rvert/n$
p-MeO	–0.27	–0.76	–0.26	0.76	–0.77	0.23	0.43	0.034	0.27	–0.12	2	0.25
p-PhO	–0.03	–0.50	–0.10	0.50	–0.46							
p-Me	–0.17	–0.31	–0.17	0.31	–0.29	0.15	0.39	0.015	0.16	–0.02	1	0.14
p-F	0.06	–0.07	–0.03	0.07	–0.24	–0.02	0.12	–0.011	–0.06	–0.25	2	0.07
p-H	0.00	0.00	0.00	0.00	0.00	0.00	0.00	0.000	0.00	0.00	1	0.00
p-F_3CO	0.35		0.27	0.35							2	0.04
p-F_3C	0.54	0.61	0.65	0.65	0.49	–0.01		0.001	0.05		1	0.11
p-MeO_2C	0.45	0.49	0.75	0.75	0.48	0.33		0.048	0.40		1	0.30
p-PhOC	0.45	0.51	0.83	0.83								
p-NC	0.66	0.66	1.00	1.00	0.86	0.42	0.41	0.043	0.47	0.34	1	0.34

[1] Polar-derived Hammett parameters are collected from the following sources: σ (based on the acidity of benzoic acid; not accounting for the stabilization of charge via resonance)[13, 14], σ^+ (based on the heterolysis reaction of *para*-substituted phenyldimethyl chloromethanes; accounting for the stabilization of positive charge via resonance)[13, 14], σ^- (based on the ionization of *para*-substituted phenols; accounting for the stabilization of negative charge via resonance)[13, 14], $\lvert\sigma^\pm\rvert$ (the magnitude of the larger value of σ^+ or σ^-, as a measurement of the stabilization of either positive or negative charge), σ_{mb} (polar σ-scale designed by Jiang and Ji, measured from the fluorine nuclear magnetic resonance (^{19}F NMR) chemical shifts of trifluorostyrenes)[15].

[2] Radical-derived Hammett parameters are collected from the following sources: $\sigma^\bullet_{JJ}$ (Jiang and Ji radical scale, measured from the cyclodimerization of styrenes)[15], $\sigma^\bullet_{J}$ (Jackson radical scale, measured from the decomposition of dibenzyl mercurials)[16], $\sigma^\bullet_{\alpha}$ (Arnold radical scale, measured from electron spin resonance hyperfine coupling constants)[17], $\sigma^\bullet_{C}$ (Creary radical scale, measured from the rearrangement of methylenecyclopropanes)[18], $\sigma^\bullet_{F}$ (Fisher radical scale, measured from bromination of 3-cyanotoluenes)[19], $\lvert(\sigma^\pm - \sigma)/n\rvert$ (a measure of radical stabilization, where n is a classification term used to determine the number of stabilization electrons: $n = 2$ for a *para*-substituent with a lone pair, else $n = 1$)[20].

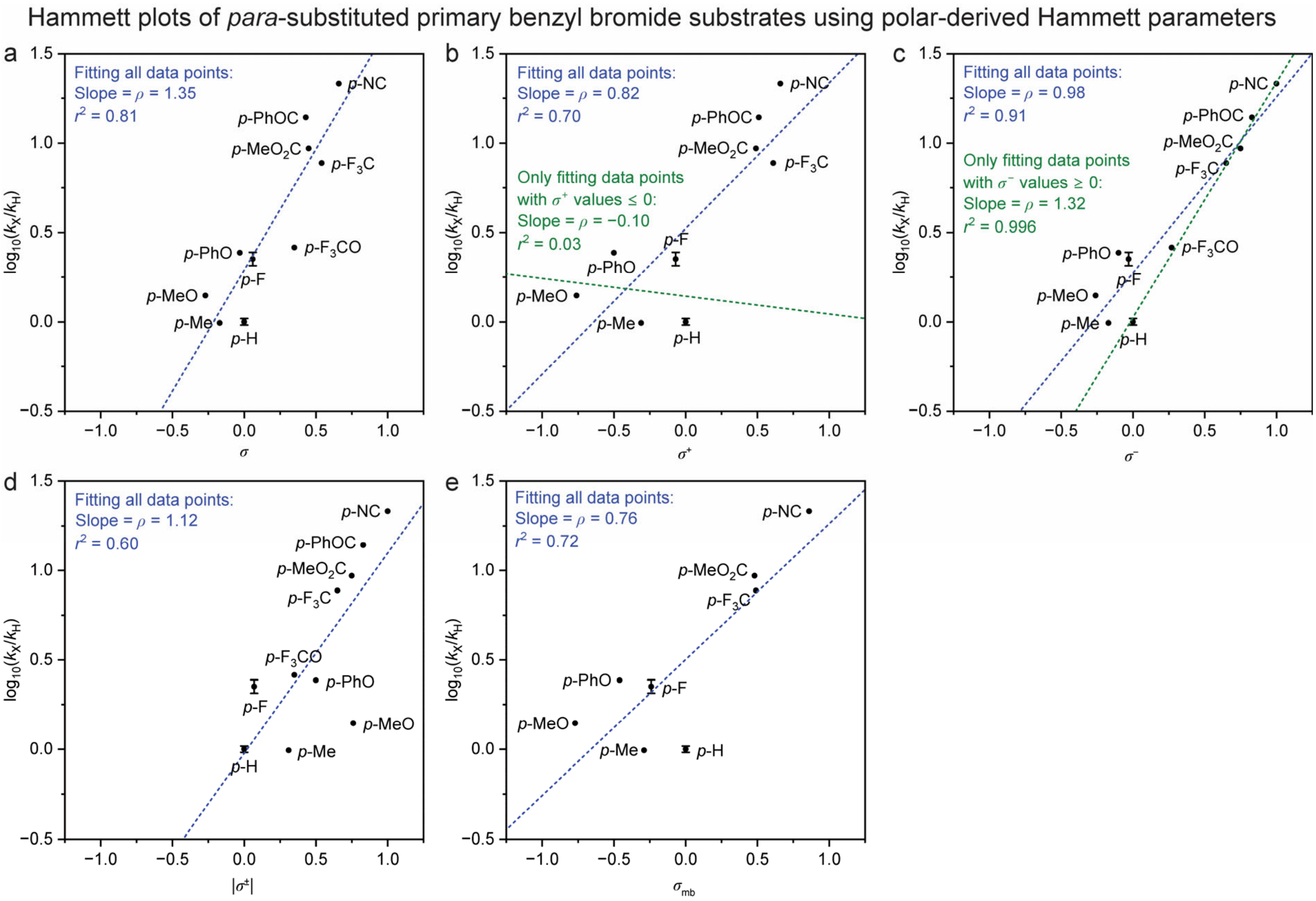

Supplementary Fig. 61. Hammett plots of *para*-substituted primary benzyl bromide substrates using polar-derived Hammett parameters. Correlations of (a) σ, (b) σ^+, (c) σ^-, (d) $|\sigma^\pm|$, and (e) σ_{mb} (defined in Supplementary Table 13) with $\log_{10}(k_X/k_H)$, where k_X and k_H are the k_0 values of *para*-substituted (*p*-X-PhCH$_2$Br, denoted as *p*-X, where X represents a *para*-substituent) and unsubstituted (PhCH$_2$Br, denoted as *p*-H) substrates, respectively.

On the σ scale, a linear correlation with a positive slope among all substrates (r^2 = 0.81, ρ = 1.35) indicates a negative charge buildup in the transition state, consistent with the anionic nature of the Co^{I}TPP nucleophile. The observed deviation from a perfect linear correlation on the σ scale could be ascribed to structural variations in the transition state that have been often observed for S$_N$2 reactions at benzylic sites[21-25], or different *para*-substituents acting to stabilize the transition state with a differing balance of inductive and resonance effects[13].

A slightly better linear correlation among all substrates is observed on the σ^- scale (r^2 = 0.91, ρ = 0.98), suggesting that resonance with the *para*-substituent plays a role in stabilizing negative charge buildup in the transition state[13], as the stabilization of negative charge via resonance is accounted for by σ^- but not by σ (Supplementary Table 13).

The Hammett study of *para*-substituted primary benzyl bromide substrates support a S$_N$2-type pathway, out of other possible alternative pathways[26-28], for the *C* step between Co^{I}TPP and RX electrophiles, as further discussed in Supplementary Note 11.

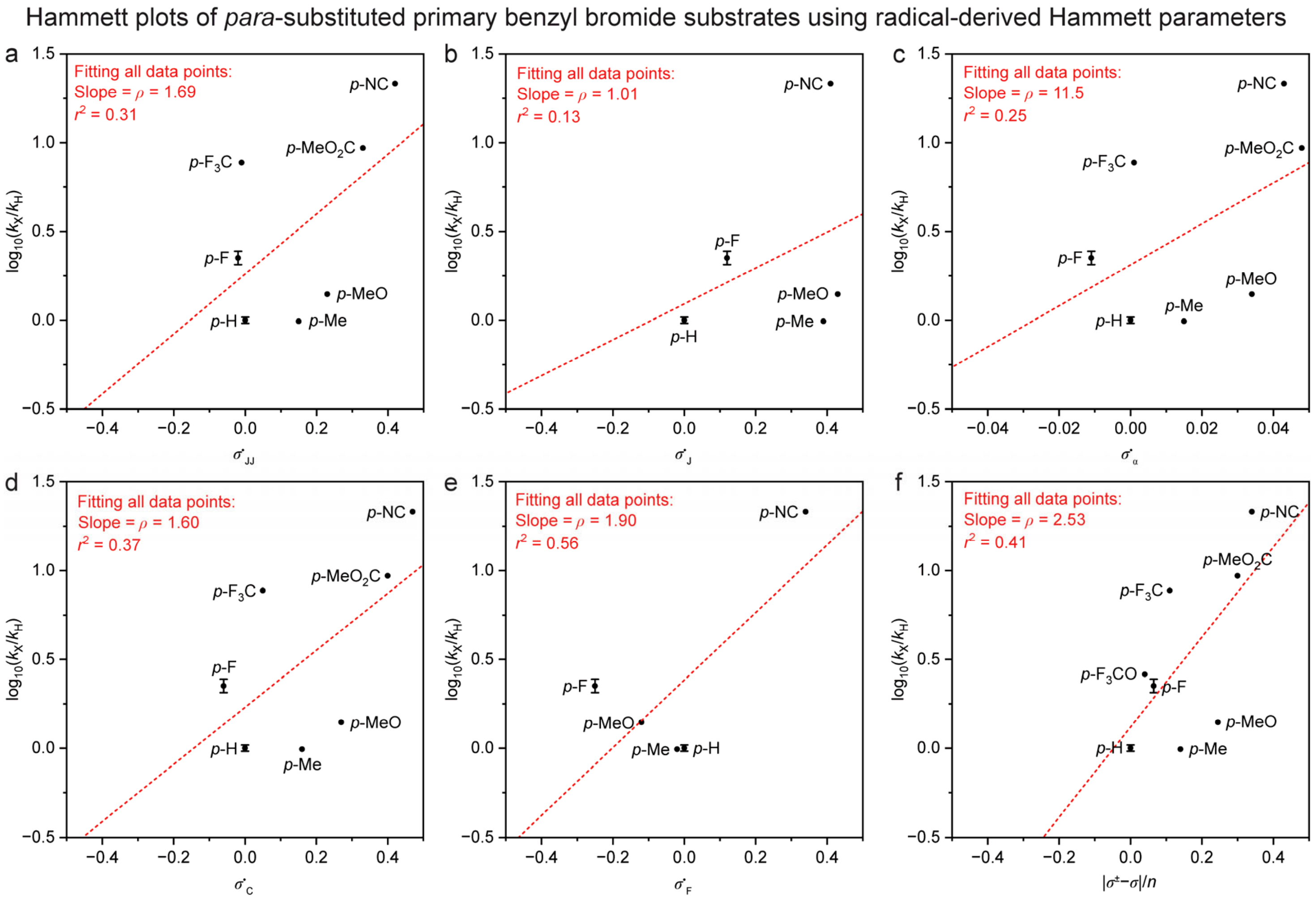

Supplementary Fig. 62. Hammett plots of *para*-substituted primary benzyl bromide substrates using radical-derived Hammett parameters. Correlations of (a) $\sigma^{\bullet}_{\mathrm{JJ}}$, (b) $\sigma^{\bullet}_{\mathrm{J}}$, (c) $\sigma^{\bullet}_{\alpha}$, (d) $\sigma^{\bullet}_{\mathrm{C}}$, (e) $\sigma^{\bullet}_{\mathrm{F}}$, and (f) $|\sigma^{\pm}-\sigma|/n$ (defined in Supplementary Table 13) with $\log_{10}(k_X/k_H)$, where k_X and k_H are the k_0 values of *para*-substituted (*p*-X-$PhCH_2Br$, denoted as *p*-X, where X represents a *para*-substituent) and unsubstituted ($PhCH_2Br$, denoted as *p*-H) substrates, respectively.

Generally poor correlations are observed when using a variety of radical-derived Hammett parameters, suggesting against singular stabilization of a radical intermediate[29].

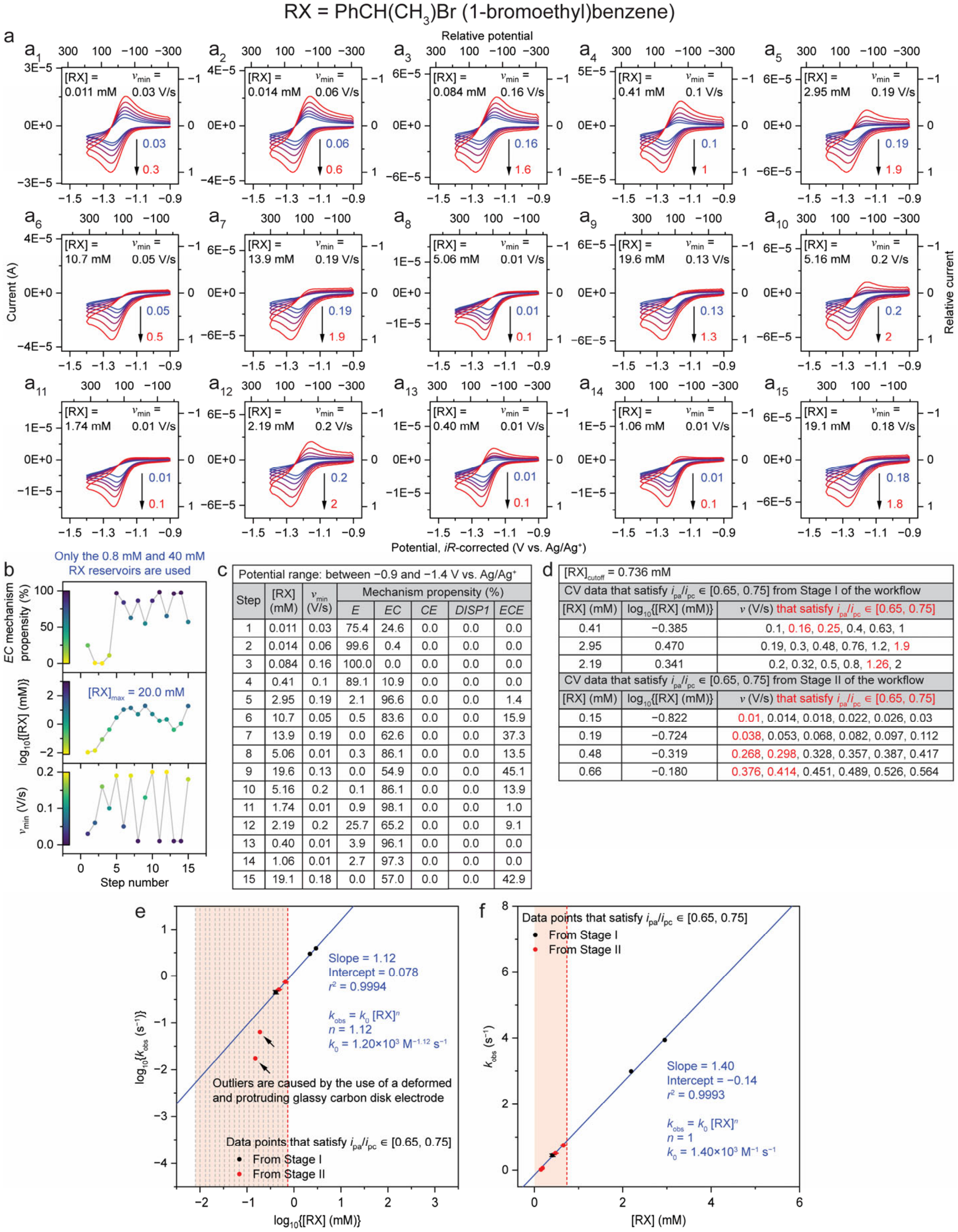

c

Potential range: between −0.9 and −1.4 V vs. Ag/Ag+							
Step	[RX] (mM)	v_{min} (V/s)	Mechanism propensity (%)				
			E	*EC*	*CE*	*DISP1*	*ECE*
1	0.011	0.03	75.4	24.6	0.0	0.0	0.0
2	0.014	0.06	99.6	0.4	0.0	0.0	0.0
3	0.084	0.16	100.0	0.0	0.0	0.0	0.0
4	0.41	0.1	89.1	10.9	0.0	0.0	0.0
5	2.95	0.19	2.1	96.6	0.0	0.0	1.4
6	10.7	0.05	0.5	83.6	0.0	0.0	15.9
7	13.9	0.19	0.0	62.6	0.0	0.0	37.3
8	5.06	0.01	0.3	86.1	0.0	0.0	13.5
9	19.6	0.13	0.0	54.9	0.0	0.0	45.1
10	5.16	0.2	0.1	86.1	0.0	0.0	13.9
11	1.74	0.01	0.9	98.1	0.0	0.0	1.0
12	2.19	0.2	25.7	65.2	0.0	0.0	9.1
13	0.40	0.01	3.9	96.1	0.0	0.0	0.0
14	1.06	0.01	2.7	97.3	0.0	0.0	0.0
15	19.1	0.18	0.0	57.0	0.0	0.0	42.9

d

$[RX]_{cutoff}$ = 0.736 mM		
CV data that satisfy $i_{pa}/i_{pc} \in [0.65, 0.75]$ from Stage I of the workflow		
[RX] (mM)	$\log_{10}$\{[RX] (mM)\}	v (V/s) that satisfy $i_{pa}/i_{pc} \in [0.65, 0.75]$
0.41	−0.385	0.1, 0.16, 0.25, 0.4, 0.63, 1
2.95	0.470	0.19, 0.3, 0.48, 0.76, 1.2, 1.9
2.19	0.341	0.2, 0.32, 0.5, 0.8, 1.26, 2
CV data that satisfy $i_{pa}/i_{pc} \in [0.65, 0.75]$ from Stage II of the workflow		
[RX] (mM)	$\log_{10}$\{[RX] (mM)\}	v (V/s) that satisfy $i_{pa}/i_{pc} \in [0.65, 0.75]$
0.15	−0.822	0.01, 0.014, 0.018, 0.022, 0.026, 0.03
0.19	−0.724	0.038, 0.053, 0.068, 0.082, 0.097, 0.112
0.48	−0.319	0.268, 0.298, 0.328, 0.357, 0.387, 0.417
0.66	−0.180	0.376, 0.414, 0.451, 0.489, 0.526, 0.564

Supplementary Fig. 63. Autonomous investigation of PhCH(CH_3)Br (1-bromoethyl)benzene) that reacts with CoTPP following an *EC* mechanism. (a) CV data (1 mM Co^{II}TPP in DMF with 0.1 M NBu_4PF_6, [RX] ∈ [0.008, 20] mM because only the 0.8 mM and 40 mM RX reservoirs are used, $v_{min} \in [0.01, 0.2]$ V/s) measured from Stage I, with detailed parameters and Bayesian optimization results summarized in (b) and (c). (d) Desired combinations of [RX] and v that satisfy $i_{pa}/i_{pc} \in [0.65, 0.75]$ from Stage I and Stage II. (e) The $\log_{10}(k_{obs})$ versus $\log_{10}$[RX] plot and (f) the k_{obs} versus [RX] plot based on all the valid k_{obs} values derived from $i_{pa}/i_{pc} \in [0.65, 0.75]$ in Stage I (black dots) and Stage II (red dots). Although the i_{pa}/i_{pc} approach is valid to use, k_0 of PhCH(CH_3)Br is determined by the E_{pc} approach (Supplementary Fig. 43f in Supplementary Note 10) for comparisons with other benzyl bromides.

Supplementary Note 11. Possible pathways for oxidative addition of RX electrophiles to Co^{I}TPP, out of which a S$_N$2-type pathway is supported by our studies of benzyl bromide substrates and beyond.

Typically, oxidative addition of RX electrophiles to an electron-rich low-valent metal center can operate in several possible pathways[29, 30]: (1) three-centered concerted oxidative addition, (2) S$_N$2-type oxidative addition, (3) outer-sphere electron transfer, and (4) halogen-atom abstraction. Note that (1) and (2) are two-electron processes forging a metal–alkyl bond, whereas (3) and (4) are one-electron processes yielding an alkyl radical intermediate followed by radical rebound with the metal center. In the context of oxidative addition of RX electrophiles to Co^{I}TPP, all the four possible pathways are depicted in Supplementary Fig. 64, regardless of whether they could be operational or not for this system.

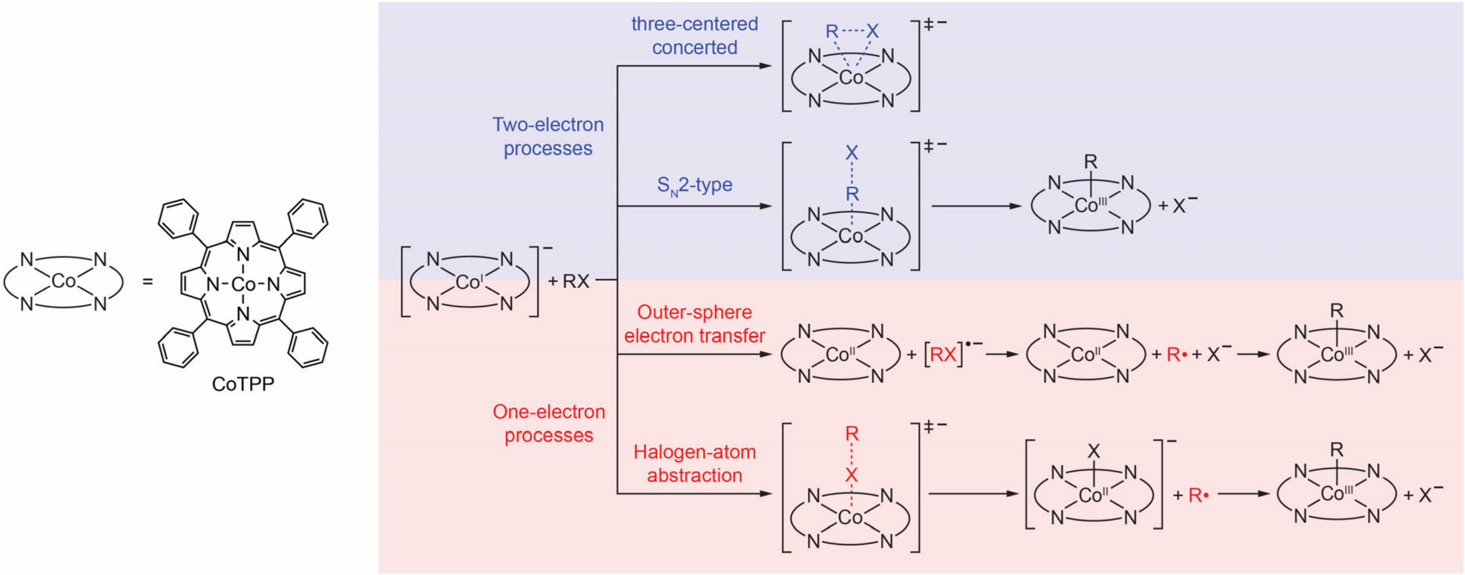

Supplementary Fig. 64. Possible pathways for oxidative addition of RX electrophiles to Co^{I}TPP. The blue shaded region indicates two electron processes forging a metal–alkyl bond (a three-centered concerted pathway or a S$_N$2-type pathway), whereas the red shaded region indicates one-electron processes yielding an alkyl radical intermediate followed by radical rebound with the metal center (an outer-sphere electron transfer pathway or a halogen-atom abstraction pathway).

We reason that CoTPP is unlikely to undergo a three-centered concerted pathway, as the rigidness of the square-planar TPP ligand is unlikely to afford six-coordinate octahedral complexes via *cis*-addition of RX electrophiles (see Supplementary Fig. 64 above). Therefore, the following discussions focus on discerning a S$_N$2-type pathway versus a pathway involving radical intermediates (either outer-sphere electron transfer or halogen-atom abstraction).

The Hammett study of *para*-substituted primary benzyl bromide substrates. Our results from the Hammett study disfavor a pathway involving radical intermediates for several reasons. First, generally poor correlations are observed when using a variety of radical-derived Hammett parameters (Supplementary Fig. 62), suggesting against singular stabilization of a radical intermediate[29]. Next, a singular linear correlation with σ or σ^{-} among both electron-rich (positive σ or σ^{-}) and electron-deficient (negative σ or σ^{-}) substrates is expected for an outer-sphere

electron transfer pathway[31-33]; however, our results suggest that the linear correlation among all substrates deviates from a perfect linear correlation on either the σ scale ($r^2 = 0.81$, Supplementary Fig. 61a) or the σ^- scale ($r^2 = 0.91$, Supplementary Fig. 61c). Last, a halogen-atom abstraction pathway at benzylic sites has been described by an unusual broken Hammett relationship where electron-rich substrates are best correlated with σ^+ but electron-deficient substrates are best correlated with σ^- (refs. 29, 33, 34); however, our results show a poor correlation with σ^+ among electron-rich substrates ($r^2 = 0.03$ when only fitting the data points with $\sigma^+ \leq 0$, Supplementary Fig. 61b) despite of an excellent correlation with σ^- among electron-rich substrates ($r^2 = 0.996$ when only fitting the data points with $\sigma^- \geq 0$, Supplementary Fig. 61c).

On the other hand, our results from the Hammett study support a S_N2-type pathway, which has been discussed in Supplementary Fig. 61. On the σ scale, a linear correlation with a positive slope among all substrates ($r^2 = 0.81$, $\rho = 1.35$, Supplementary Fig. 61a) indicates a negative charge buildup in the transition state, consistent with the anionic nature of the $Co^{I}TPP$ nucleophile. The observed deviation from a perfect linear correlation on the σ scale could be ascribed to structural variations in the transition state that have been often observed for S_N2 reactions at benzylic sites[21-25], or different *para*-substituents acting to stabilize the transition state with a differing balance of inductive and resonance effects[13]. A slightly better linear correlation among all substrates is observed on the σ^- scale ($r^2 = 0.91$, $\rho = 0.98$, Supplementary Fig. 61c), suggesting that resonance with the *para*-substituent plays a role in stabilizing negative charge buildup in the transition state[13], as the stabilization of negative charge via resonance is accounted for by σ^- but not by σ (Supplementary Table 13).

Secondary benzyl bromide substrate. Our observation of $PhCH(CH_3)Br$ being less reactive toward $Co^{I}TPP$ than $PhCH_2Br$ (see their k_0 values summarized in Fig. 4a in the main text) suggests against a halogen-atom abstraction pathway, as α-methyl substitution can stabilize the benzylic radical through hyperconjugation and enhance the rate of halogen-atom abstraction for secondary substrates[29, 33]. In fact, the relative reaction rate for $PhCH(CH_3)Br$ versus $PhCH_2Br$ is aligned with typical organic S_N2 reactions (see Supplementary Table 15).

Other RX substrates. The observed reactivity trends for other RX substrates studied in this work also support a S_N2-type pathway (see their k_0 values summarized in Fig. 4a in the main text), namely an increased reactivity with a better leaving group (*n*-BuI > *n*-BuBr > *n*-BuCl; $I(CH_2)_3CN$ > $Br(CH_2)_3CN$) and with less steric hindrance (*n*-BuBr > *i*-BuBr > 2-BuBr; *n*-BuI > Me_3CCH_2I), and a similar reactivity with respect to linear alkyl chain length (*n*-BuBr ≈ *n*-HexBr ≈ *n*-OctBr; $Cl(CH_2)_2CN \approx Cl(CH_2)_4CN$)[35]. Comparisons of relative reaction rates between $Co^{I}TPP$ and RX substrates with respect to leaving group (the same alkyl group but different leaving groups) and with respect to alkyl group (the same leaving group but different alkyl groups) are also aligned with typical organic S_N2 reactions[35, 36] (Supplementary Tables 14 and 15).

Supplementary Table 14. Comparisons of relative reaction rates between $Co^{I}TPP$ and RX substrates with respect to leaving group (the same alkyl group but different leaving groups).

Reaction type	Reaction system (temperature, solvent)	R group	k_{Cl}/k_{Br} [1]	k_{I}/k_{Br} [1]	Reference
S_N2	Typical organic S_N2 reactions on average		2×10^{-2}	~3	Table 16 in ref. 36
S_N2	Tributylphosphine–cobaloxime(I) + RX (25 °C, methanol)	CH_3–	3.9×10^{-3}	10	Table III in ref. 35
		CH_3CH_2–	5.7×10^{-3}		
		$CH_3CH_2CH_2$–	4.2×10^{-3}		
		$(CH_3)_2CH$–	2.9×10^{-3}	29	
		$(CH_3)_2CHCH_2$–	2.8×10^{-3}	30	
		$C_6H_5CH_2$–	2.3×10^{-2}		
		$^{-}OOCCH_2$–		7.4	
S_N2	Cob(I)alamin (Vitamin B_{12s}) + RX (25 °C, methanol)	CH_3–	3.1×10^{-3}	21	Table III in ref. 35
		CH_3CH_2–	1.5×10^{-3}		
		$CH_3CH_2CH_2$–	2.6×10^{-3}		
		$(CH_3)_2CHCH_2$–	1.9×10^{-3}		
Halogen-atom abstraction	Pentacyanocobalt(II) ($Co(CN)_5^{3-}$) + RX (25 °C, water) [2]	$^{-}OOCCH_2$–	7.1×10^{-4}	3.2×10^{3}	Table III in ref. 35 (source data are from ref. 37)
		$H_3COOCCH_2$–	2.9×10^{-5}	2.6×10^{3}	
		$H_2NC(O)CH_2$–	3.7×10^{-5}	1.8×10^{3}	
		$^{-}OOCCH_2CH_2$–		6.8×10^{2}	
**Aligned with S_N2**	Cobalt(I) tetraphenylporphyrin ($Co^{I}TPP$) + RX (room temperature ~25 °C, anhydrous DMF)	$CH_3(CH_2)_3$–		93 ± 4	This work
		$CD_3(CD_2)_3$–		88	
		$NC(CH_2)_3$–		65	

[1] k_{Cl}, k_{Br}, and k_{I} stand for the kinetic rate constants for chloride, bromide, and iodide substrates with the same alkyl group. In this work, the kinetic rate constants are reported as the second-order kinetic rate constants (k_0 in the unit of $M^{-1}\ s^{-1}$) between $Co^{I}TPP$ and RX substrates. The k_0 values used for calculations in this table can be found in Supplementary Figs. 28 (three replicates of *n*-BuBr), 29 (*n*-BuI), 40 (*n*-BuBr-d_9), 41 (*n*-BuI-d_9), 66 ($Br(CH_2)_3CN$), and 67 ($I(CH_2)_3CN$), all of which are determined by the i_{pa}/i_{pc} approach (Supplementary Note 5).

[2] Pentacyanocobalt(II) ($Co(CN)_5^{3-}$) reacts with RX substrates via halogen-atom abstraction mechanism (ref. 37):

$$Co(CN)_5^{3-} + RX \rightarrow X\text{–}Co(CN)_5^{3-} + R\cdot \text{ (rate-determining)}$$

$$Co(CN)_5^{3-} + R\cdot \rightarrow R\text{–}Co(CN)_5^{3-} \text{ (fast)}$$

Supplementary Table 15. Comparisons of relative reaction rates between $Co^{I}TPP$ and RX substrates with respect to alkyl group (the same alkyl group but different leaving groups).

Reaction type	Reaction system (temperature, solvent)	R group or RX substrate	R' group or R'X substrate	$k_R/k_{R'}$ [1]	Reference
S_N2	Typical organic S_N2 reactions on average	$(CH_3)_2CHCH_2$–	$CH_3(CH_2)_3$–	0.03	Table 5 in ref. 36
		Me_3CCH_2–	$CH_3(CH_2)_3$–	10^{-5}	
		$PhCH_2$–	$CH_3(CH_2)_3$–	1.2×10^{2}	
S_N2	Tributylphosphine–cobaloxime(I) + RX (25 °C, methanol)	$(CH_3)_2CHCH_2Cl$	$CH_3(CH_2)_3Cl$	0.11	Tables I and IV in ref. 35
		$(CH_3)_2CHCH_2I$	$CH_3CH_2CH(CH_3)I$	10	
		$(CH_3)_2CHCH_2Cl$	$PhCH(CH_3)Cl$	3.2×10^{-3}	
		$PhCH_2$–	$PhCH(CH_3)$–	2.0×10^{2}	
S_N2	Cob(I)alamin (Vitamin B_{12s}) + RX (25 °C, methanol)	$(CH_3)_2CHCH_2Cl$	$CH_3(CH_2)_3Cl$	0.15	Table II in ref. 35
**Aligned with S_N2**	Cobalt(I) tetraphenylporphyrin ($Co^{I}TPP$) + RX (room temperature ~25 °C, anhydrous DMF)	$(CH_3)_2CHCH_2Br$	$CH_3(CH_2)_3Br$	0.13 ± 0.01	This work
		$(CH_3)_2CHCH_2Br$	$CH_3CH_2CH(CH_3)Br$	33	
		Me_3CCH_2I	$CH_3(CH_2)_3I$	2.4×10^{-3}	
		$PhCH_2Br$	$CH_3(CH_2)_3Br$	$(2.7 \pm 0.1) \times 10^{3}$	
		$(CH_3)_2CHCH_2Br$	$PhCH(CH_3)Br$	4.4×10^{-3}	
		$PhCH_2Br$	$PhCH(CH_3)Br$	90 ± 4	

[1] k_R and $k_{R'}$ stand for the kinetic rate constants for RX substrates with different alkyl groups (R vs. R') but the same leaving group (Cl, or Br, or I). In this work, the kinetic rate constants are reported as the second-order kinetic rate constants (k_0 in the unit of M^{-1} s^{-1}) between $Co^{I}TPP$ and RX substrates. The k_0 values used for calculations in this table can be found in Supplementary Figs. 28 (three replicates of *n*-BuBr), 29 (*n*-BuI), 33 (*i*-BuBr), 34 (2-BuBr), 35 (Me_3CCH_2I), 48 (two replicates of $PhCH_2Br$), and 43f ($PhCH(CH_3)Br$). The k_0 values of *n*-BuBr, *n*-BuI, *i*-BuBr, *i*-BuBr, and Me_3CCH_2I are determined by the i_{pa}/i_{pc} approach (Supplementary Note 5), whereas those of $PhCH_2Br$ and $PhCH(CH_3)Br$ are determined by the E_{pc} approach (Supplementary Note 10).

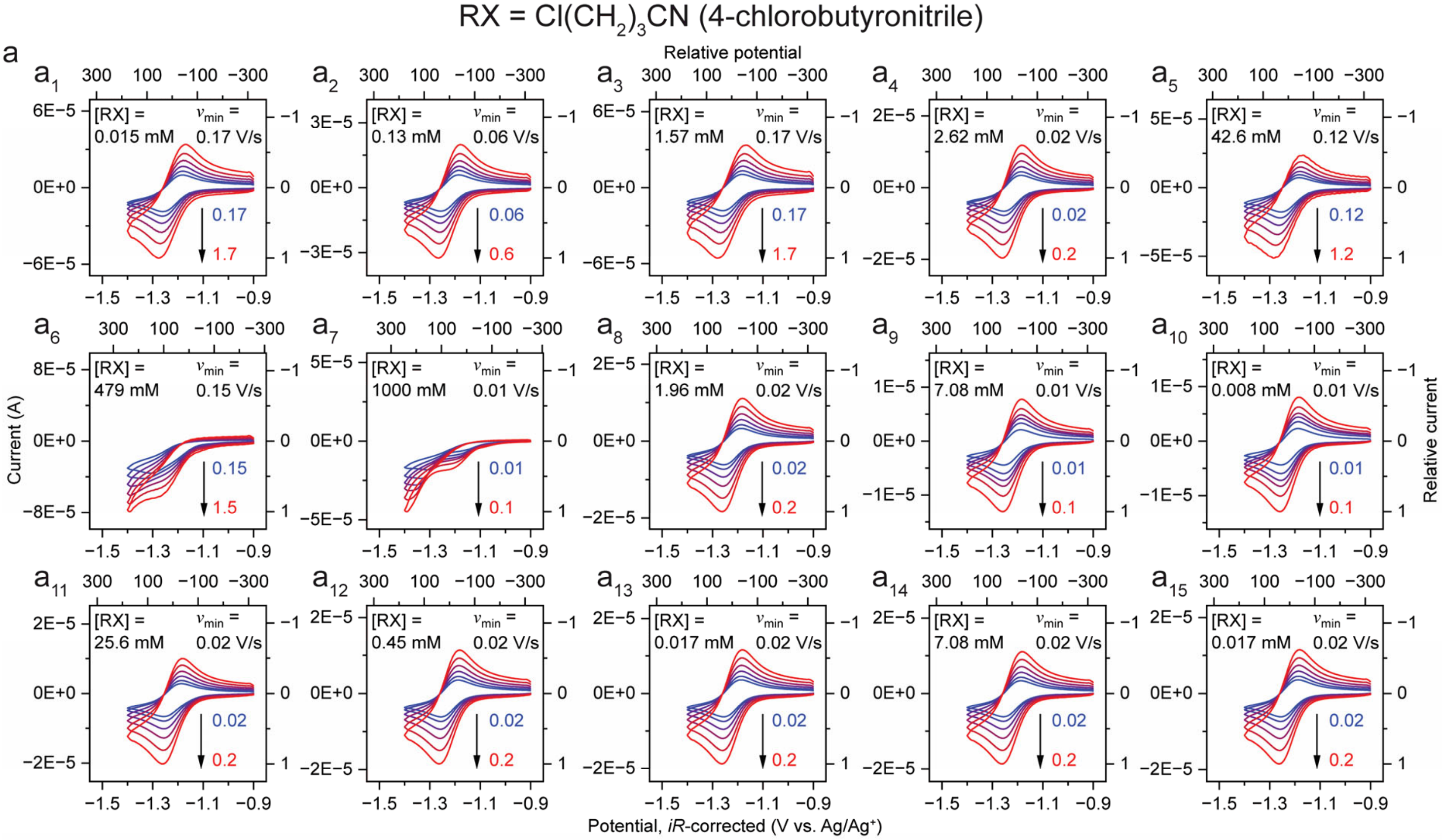

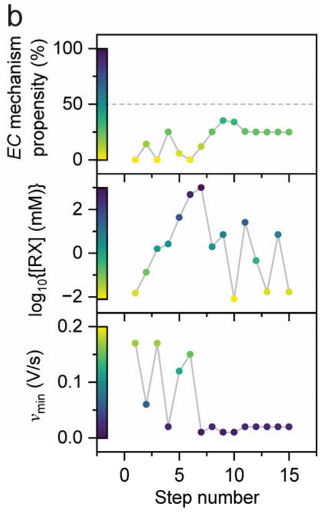

c

Potential range: between −0.9 and −1.4 V vs. Ag/Ag+							
Step	[RX] (mM)	v_{min} (V/s)	Mechanism propensity (%)				
			E	*EC*	*CE*	*DISP1*	*ECE*
1	0.015	0.17	100.0	0.0	0.0	0.0	0.0
2	0.13	0.06	85.9	14.1	0.0	0.0	0.0
3	1.57	0.17	100.0	0.0	0.0	0.0	0.0
4	2.62	0.02	75.0	25.0	0.0	0.0	0.0
5	42.6	0.12	94.2	5.8	0.0	0.0	0.0
6	479	0.15	0.0	0.0	0.0	0.3	99.7
7	1000	0.01	0.0	11.8	0.0	14.1	74.1
8	1.96	0.02	75.0	25.0	0.0	0.0	0.0
9	7.08	0.01	64.8	35.2	0.0	0.0	0.0
10	0.008	0.01	66.2	33.8	0.0	0.0	0.0
11	25.6	0.02	74.6	25.4	0.0	0.0	0.0
12	0.45	0.02	75.1	24.9	0.0	0.0	0.0
13	0.017	0.02	75.2	24.8	0.0	0.0	0.0
14	7.08	0.02	75.1	24.9	0.0	0.0	0.0
15	0.017	0.02	75.2	24.8	0.0	0.0	0.0

Supplementary Fig. 65. Autonomous investigation of $Cl(CH_2)_3CN$ (3-chlorobutyronitrile) that reacts with CoTPP but does not follow an *EC* mechanism. (a) CV data (1 mM Co^{II}TPP in DMF with 0.1 M NBu_4PF_6, [RX] ∈ [0.008, 1000] mM, v_{min} ∈ [0.01, 0.2] V/s) measured from Stage I, with detailed parameters and Bayesian optimization results summarized in (b) and (c). Under a high [RX] such as 479 mM in (a6) and 1000 mM in (a_7), the CV responses deviate from a prototypical *EC* mechanism, and the DL model correctly identified the existence of alternative mechanisms as shown in (c). As the DL-generated propensity of *EC* mechanism did not exceed the 50% threshold, the closed-loop workflow autonomously ended after Stage I and did not proceed to Stage II (see the flowchart in Supplementary Fig. 16). This example indicates that our autonomous platform is sensitive enough to detect outliers that bear previously unexpected mechanisms.

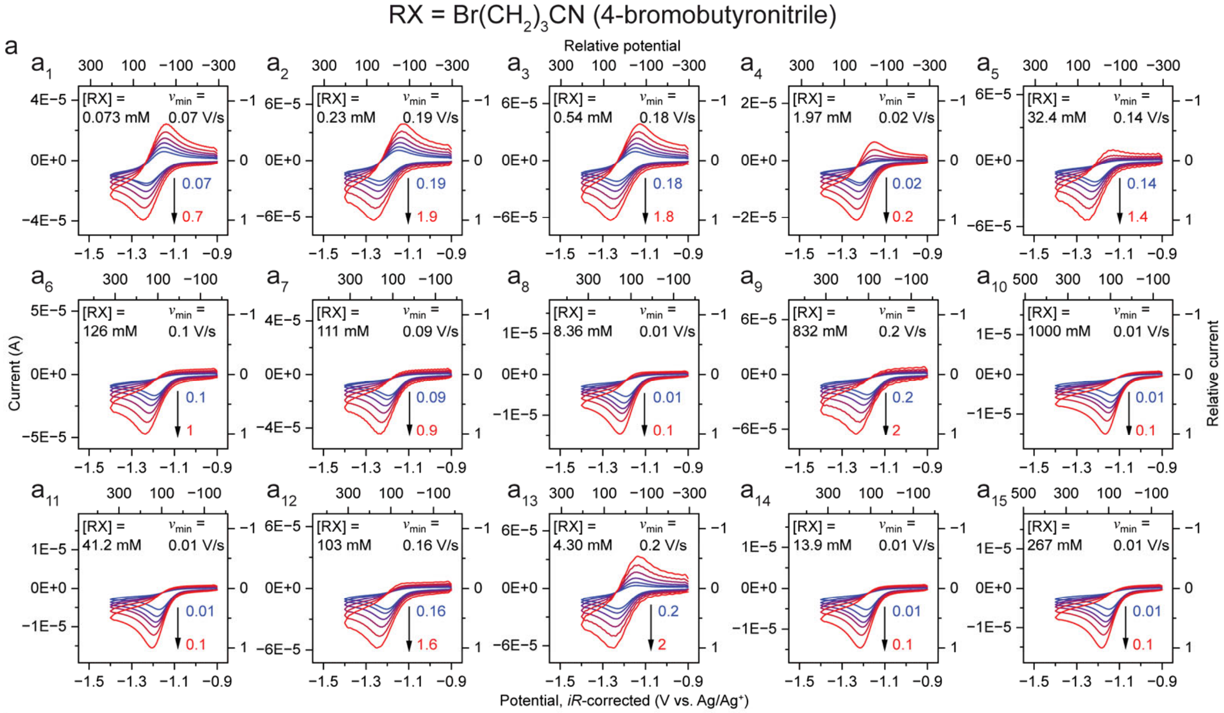

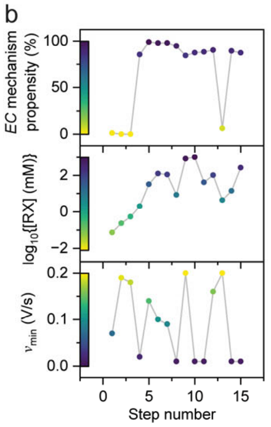

c

Potential range: between −0.9 and −1.4 V vs. Ag/Ag⁺							
Step	[RX] (mM)	v_{min} (V/s)	Mechanism propensity (%)				
			E	*EC*	*CE*	*DISP1*	*ECE*
1	0.073	0.07	98.8	1.2	0.0	0.0	0.0
2	0.23	0.19	100.0	0.0	0.0	0.0	0.0
3	0.54	0.18	100.0	0.0	0.0	0.0	0.0
4	1.97	0.02	14.5	85.5	0.0	0.0	0.0
5	32.4	0.14	0.3	99.0	0.0	0.0	0.6
6	126	0.1	1.0	97.9	0.0	0.0	1.1
7	111	0.09	1.4	98.0	0.0	0.0	0.6
8	8.36	0.01	5.3	94.7	0.0	0.0	0.0
9	832	0.2	2.8	84.5	0.0	0.0	12.7
10	1000	0.01	12.5	87.5	0.0	0.0	0.0
11	41.2	0.01	11.6	88.4	0.0	0.0	0.0
12	103	0.16	0.3	90.4	0.0	0.0	9.3
13	4.30	0.2	93.8	6.2	0.0	0.0	0.0
14	13.9	0.01	10.5	89.5	0.0	0.0	0.0
15	267	0.01	12.6	87.4	0.0	0.0	0.0

d

$[RX]_{cutoff}$ = 11.309 mM		
CV data that satisfy i_{pa}/i_{pc} ∈ [0.65, 0.75] from Stage I of the workflow		
[RX] (mM)	log_{10}\{[RX] (mM)\}	v (V/s) that satisfy i_{pa}/i_{pc} ∈ [0.65, 0.75]
1.97	0.295	0.02, 0.03, 0.05, 0.08, 0.13, 0.2
4.30	0.633	0.2, 0.32, 0.5, 0.8, 1.26, 2
CV data that satisfy i_{pa}/i_{pc} ∈ [0.65, 0.75] from Stage II of the workflow		
[RX] (mM)	log_{10}\{[RX] (mM)\}	v (V/s) that satisfy i_{pa}/i_{pc} ∈ [0.65, 0.75]
0.008	−2.08	0.01, 0.014, 0.018, 0.022, 0.026, 0.03
0.13	−0.902	0.01, 0.014, 0.018, 0.022, 0.026, 0.03
0.24	−0.614	0.01, 0.014, 0.018, 0.022, 0.026, 0.03
0.27	−0.570	0.021, 0.032 0.043, 0.054, 0.065, 0.76
0.46	−0.341	0.048, 0.065, 0.082, 0.099, 0.116, 0.133
0.58	−0.240	0.052, 0.069, 0.087, 0.104, 0.122, 0.139
0.95	−0.021	0.098, 0.122, 0.144, 0.167, 0.19, 0.213
1.52	0.182	0.154, 0.18, 0.207, 0.234, 0.26, 0.287
3.36	0.526	0.352, 0.387, 0.422, 0.458, 0.493, 0.528
6.08	0.784	0.592, 0.651, 0.71, 0.77, 0.829, 0.888
8.35	0.922	0.772, 0.849, 0.926, 1, 1.08, 1.16

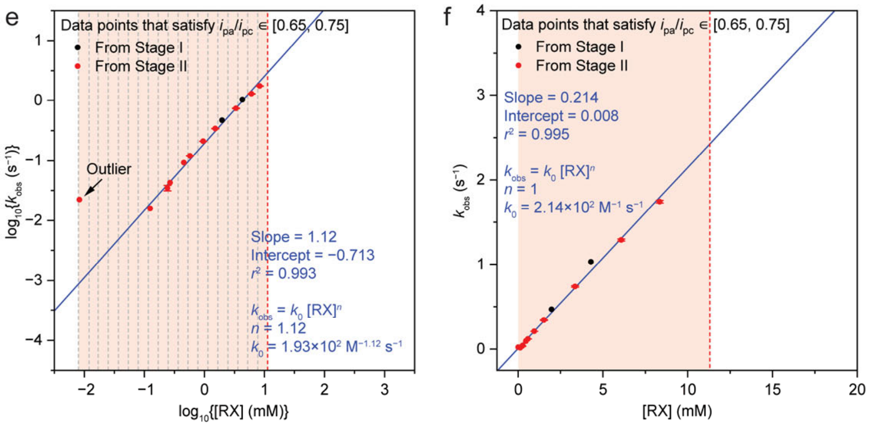

Supplementary Fig. 66. Autonomous investigation of $Br(CH_2)_3CN$ (4-bromobutyronitrile) that reacts with CoTPP following an *EC* mechanism. (a) CV data (1 mM Co^{II}TPP in DMF with 0.1 M NBu_4PF_6, [RX] ∈ [0.008, 1000] mM, v_{min} ∈ [0.01, 0.2] V/s) measured from Stage I, with detailed parameters and Bayesian optimization results summarized in (b) and (c). (d) Desired combinations of [RX] and v that satisfy i_{pa}/i_{pc} ∈ [0.65, 0.75] from Stage I and Stage II. (e) The $log_{10}(k_{obs})$ versus log_{10}[RX] plot and (f) the k_{obs} versus [RX] plot based on all the valid k_{obs} values derived from i_{pa}/i_{pc} ∈ [0.65, 0.75] in Stage I (black dots) and Stage II (red dots).

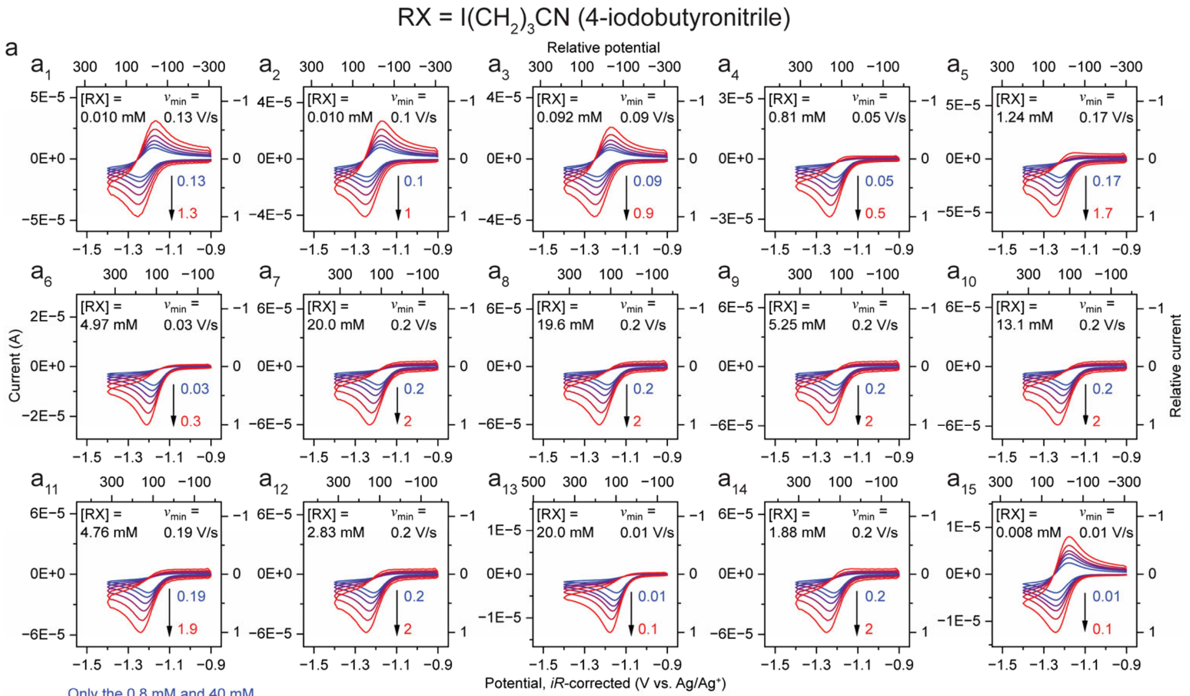

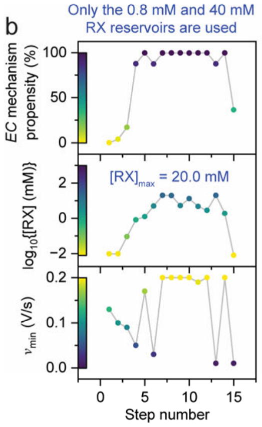

c

Potential range: between −0.9 and −1.4 V vs. Ag/Ag+							
Step	[RX] (mM)	v_{min} (V/s)	Mechanism propensity (%)				
			E	*EC*	*CE*	*DISP1*	*ECE*
1	0.010	0.13	99.9	0.1	0.0	0.0	0.0
2	0.010	0.1	96.0	4.0	0.0	0.0	0.0
3	0.092	0.09	83.0	17.0	0.0	0.0	0.0
4	0.81	0.05	12.4	87.6	0.0	0.0	0.0
5	1.24	0.17	0.2	99.8	0.0	0.0	0.0
6	4.97	0.03	12.6	87.4	0.0	0.0	0.0
7	20.0	0.2	0.3	99.7	0.0	0.0	0.0
8	19.6	0.2	0.3	99.7	0.0	0.0	0.0
9	5.25	0.2	0.2	99.8	0.0	0.0	0.0
10	13.1	0.2	0.3	99.7	0.0	0.0	0.0
11	4.76	0.19	0.2	99.8	0.0	0.0	0.0
12	2.83	0.2	0.2	99.8	0.0	0.0	0.0
13	20.0	0.01	12.5	87.5	0.0	0.0	0.0
14	1.88	0.2	0.2	99.8	0.0	0.0	0.0
15	0.008	0.01	63.8	36.2	0.0	0.0	0.0

d

$[RX]_{cutoff}$ = 0.408 mM		
CV data that satisfy i_{pa}/i_{pc} ∈ [0.65, 0.75] from Stage I of the workflow		
[RX] (mM)	$\log_{10}${[RX] (mM)}	v (V/s) that satisfy i_{pa}/i_{pc} ∈ [0.65, 0.75]
None	None	None
CVs that satisfy i_{pa}/i_{pc} ∈ [0.65, 0.75] from Stage II of the workflow		
[RX] (mM)	$\log_{10}${[RX] (mM)}	v (V/s) that satisfy i_{pa}/i_{pc} ∈ [0.65, 0.75]
0.20	−0.698	0.62, 0.682, 0.744, 0.806, 0.868, 0.93
0.24	−0.612	0.912, 1, 1.09, 1.19, 1.28, 1.37
0.27	−0.567	1.05, 1.16, 1.26, 1.37, 1.47, 1.58

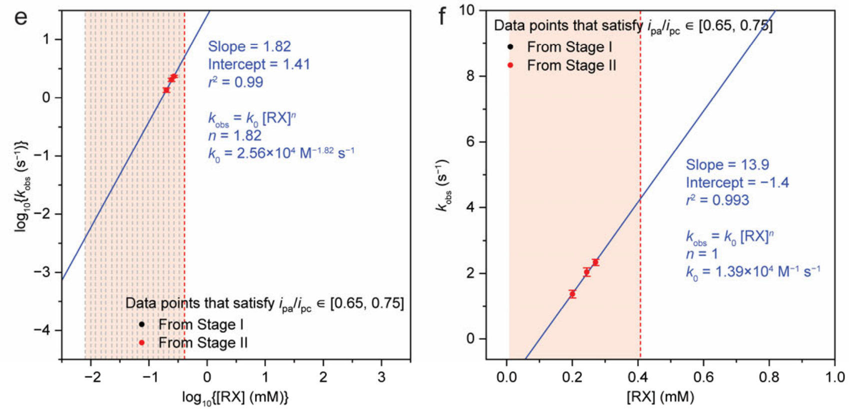

Supplementary Fig. 67. Autonomous investigation of $I(CH_2)_3CN$ (4-iodobutyronitrile) that reacts with CoTPP following an *EC* mechanism. (a) CV data (1 mM Co^{II}TPP in DMF with 0.1 M NBu_4PF_6, [RX] ∈ [0.008, 20] mM because only the 0.8 mM and 40 mM RX reservoirs are used, v_{min} ∈ [0.01, 0.2] V/s) measured from Stage I, with detailed parameters and Bayesian optimization results summarized in (b) and (c). (d) Desired combinations of [RX] and v that satisfy i_{pa}/i_{pc} ∈ [0.65, 0.75] from Stage I and Stage II. (e) The $\log_{10}(k_{obs})$ versus $\log_{10}$[RX] plot and (f) the k_{obs} versus [RX] plot based on all the valid k_{obs} values derived from i_{pa}/i_{pc} ∈ [0.65, 0.75] in Stage I (black dots) and Stage II (red dots).

Supplementary Note 12. Additional manual experiments to confirm $Cl(CH_2)_3CN$ as a mechanistic outlier, with the exclusion of potential artifacts.

As shown in Supplementary Fig. 65, autonomous investigation suggests $Cl(CH_2)_3CN$ as a mechanistic outlier that reacts with CoTPP but does not follow an *EC* mechanism. Selected CV data from this autonomous investigation are reproduced below in Supplementary Figs. 68a and 68b, where the CV responses deviate from a prototypical *EC* mechanism under a high [RX] such as 1000 mM. Prior to use for this autonomous investigation, as-received $Cl(CH_2)_3CN$ (TCI America, >97.0%) was transferred in a Schlenk flask, evacuated under vacuum on a Schlenk line, brought into the glovebox, and dried over 3 Å molecular sieves.

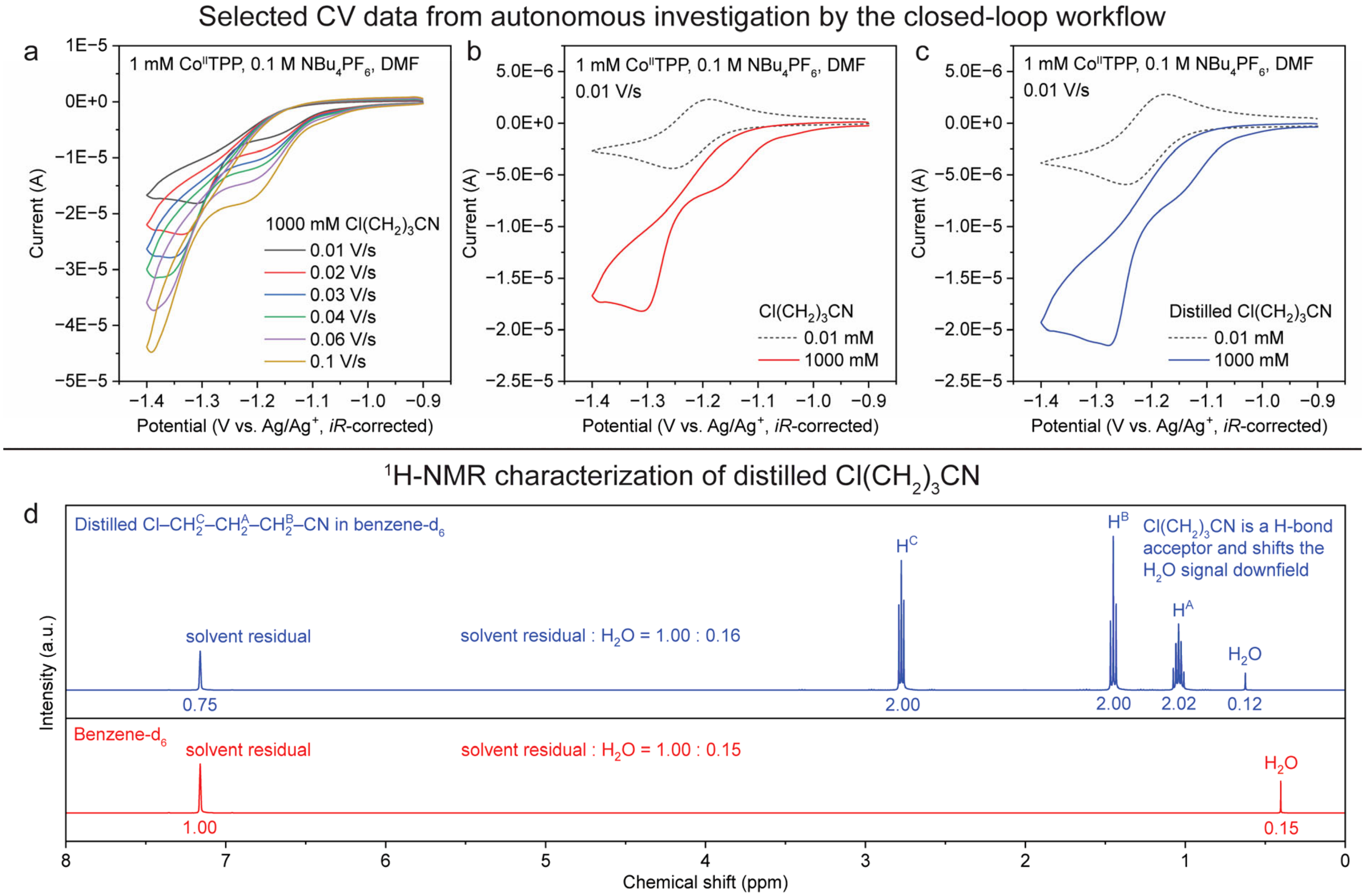

Supplementary Fig. 68. Exclusion of the potential artifact concerning chemical purity. Selected CV data from autonomous investigation by the closed-loop workflow using (a, b) as-received or (c) distilled $Cl(CH_2)_3CN$. (d) Proton nuclear magnetic resonance (^{1}H-NMR) characterizations of as-received versus distilled $Cl(CH_2)_3CN$.

To exclude the potential artifact concerning chemical purity, we distilled as-received $Cl(CH_2)_3CN$ and collected the fraction at 181–185 °C in a Schlenk flask, followed by the same procedure as mentioned (evacuated under vacuum on a Schlenk line, brought into the glovebox, and dried over 3 Å molecular sieves). Autonomous investigation was conducted again using distilled $Cl(CH_2)_3CN$. As shown above in Supplementary Fig. 68c, the same CV responses that deviate from an *EC* mechanism were still observed. Supplementary Fig. 68d shows the results of proton nuclear

magnetic resonance (^{1}H-NMR) characterizations performed on a Bruker Avance 400 MHz NMR spectrometer, which confirm the purity and dryness of distilled $Cl(CH_2)_3CN$. Note that the H_2O signal comes from the benzene-d_6 solvent (Cambridge Isotope Laboratories, Inc.), as the peak area ratio of the solvent residual signal to the H_2O signal is almost the same for the blank solvent and the sample that contains $Cl(CH_2)_3CN$.

To exclude other potential artifacts concerning automated electrolyte formulation or electrode fouling, we further performed additional manual CV measurements using a freshly polished glassy carbon disk working electrode. We manually prepared 4 mL of the electrolyte solution (1 mM $Co^{II}TPP$ in anhydrous DMF solvent with 0.1 M NBu_4PF_6 supporting electrolyte), and measured CV at 0.01 V/s while sequentially titrating $Cl(CH_2)_3CN$ with a total volume of 10, 20, 40, 100, 200, and 400 μL into the electrolyte solution, resulting in 26, 53, 106, 264, 529, and 1057 equivalent (equiv.) of $Cl(CH_2)_3CN$ relative to $Co^{II}TPP$. The CV measured manually under 1057 equiv. of $Cl(CH_2)_3CN$ (Supplementary Fig. 69b) matches with that measured from autonomous investigation under 1000 mM $Cl(CH_2)_3CN$ (Supplementary Fig. 68b), indicating that automated electrolyte formulation is trustworthy and there is no electrode fouling during autonomous investigation. Moreover, in the absence of $Co^{II}TPP$, $Cl(CH_2)_3CN$ itself is electrochemically inert in the measured potential range (Supplementary Fig. 69c), confirming it is indeed the follow-up reaction between $Co^{I}TPP$ and $Cl(CH_2)_3CN$ that deviates the CV responses from an *EC* mechanism.

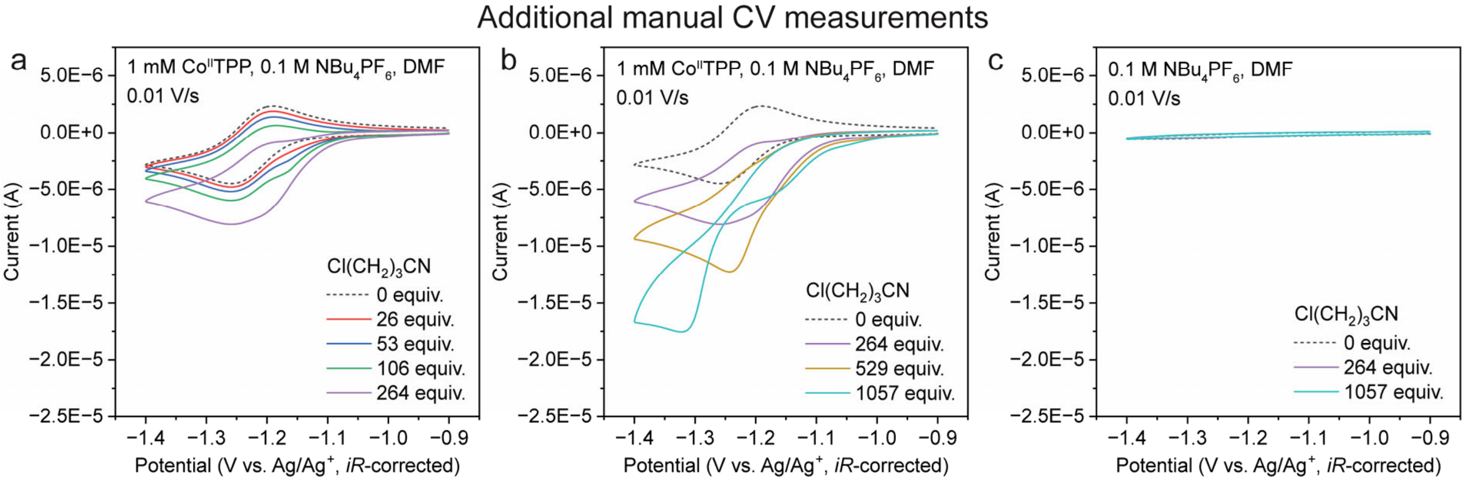

Supplementary Fig. 69. Exclusion of other potential artifacts concerning automated electrolyte formulation or electrode fouling. Manual CV measurements using a freshly polished glassy carbon disk working electrode under different equivalents of $Cl(CH_2)_3CN$ in the (a, b) presence or (c) absence of $Co^{II}TPP$.

Based on all the additional control experiments presented in this Supplementary Note 12, we confirm $Cl(CH_2)_3CN$ as a mechanistic outlier that reacts with CoTPP but does not follow an *EC* mechanism, with the exclusion of potential artifacts including chemical purity, automated electrolyte formulation, or electrode fouling. Mechanistic insights into the existence of such mechanistic outlier are further discussed in Supplementary Note 13.

Supplementary Note 13. Mechanistic insights into the existence of $Cl(CH_2)_3CN$ as a mechanistic outlier that reacts with CoTPP but does not follow an *EC* mechanism.

In Supplementary Note 12, additional manual CV measurements confirm $Cl(CH_2)_3CN$ as a mechanistic outlier that reacts with CoTPP but does not follow an *EC* mechanism, with the exclusion of potential artifacts concerning chemical purity, automated electrolyte formulation, or electrode fouling. Those CV data are reproduced below for further discussions in this Supplementary Note 13.

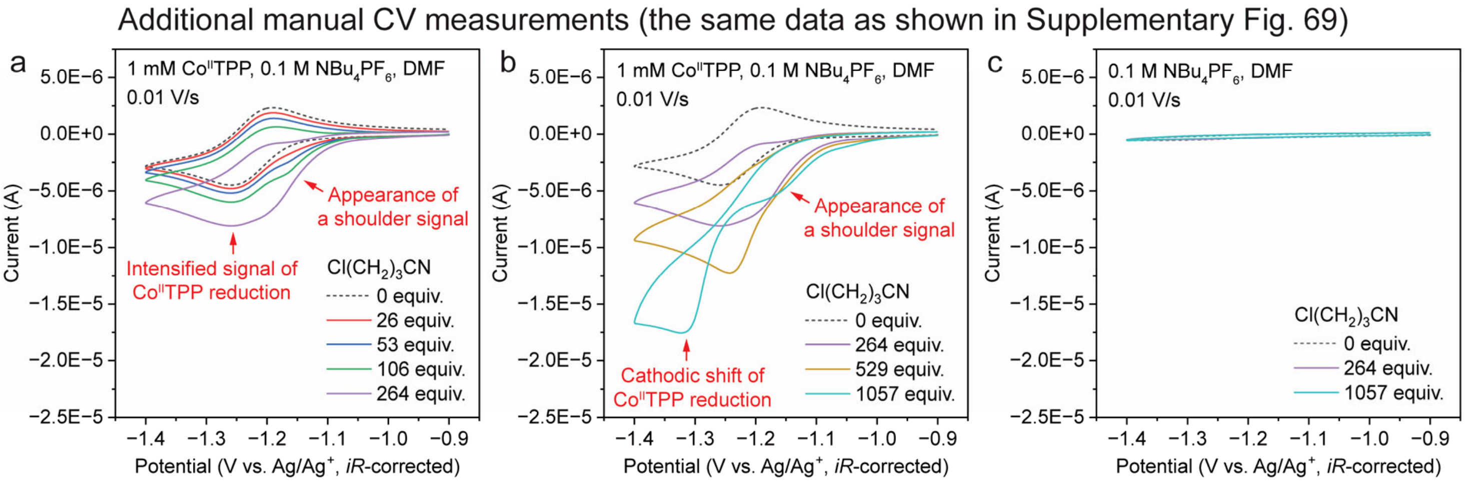

Supplementary Fig. 70. Further analysis of the CV responses of CoTPP with $Cl(CH_2)_3CN$. The same data as shown in Supplementary Fig. 69 are reproduced in this figure. In the presence of both CoTPP and $Cl(CH_2)_3CN$, the CV responses that deviate from a prototypical *EC* mechanism are labeled with red arrows and annotations.

With an increase in $[Cl(CH_2)_3CN]$, the following CV responses deviate from a prototypical *EC* mechanism: (1) appearance of an irreversible reductive shoulder peak anodic of the $Co^{II/I}$ redox; (2) an intensified reductive peak of Co^{II}TPP; (3) a cathodic shift of the reduction peak of Co^{II}TPP. The latter two features are indicative of catalytic regeneration[29, 38] of Co^{II}TPP. As mentioned in Supplementary Note 11, oxidative addition of RX electrophiles to Co^{I}TPP could possibly undergo a one-electron process, via either an outer-sphere electron transfer pathway or a halogen-atom abstraction pathway, which yields an alkyl radical intermediate and regenerates Co^{II}TPP. In the context of Co^{I}TPP exposed to $Cl(CH_2)_3CN$, the reactions can be written as:

$$\text{Outer-sphere electron transfer: } [Co^{I}TPP]^{-} + Cl(CH_2)_3CN \rightarrow Co^{II}TPP + NC(CH_2)_3\cdot + Cl^{-}$$

$$\text{Halogen-atom abstraction: } [Co^{I}TPP]^{-} + Cl(CH_2)_3CN \rightarrow [Cl\text{–}Co^{II}TPP]^{-} + NC(CH_2)_3\cdot$$

The yielded $NC(CH_2)_3\cdot$ radical intermediate may either recombine with the metal center to form a metal–alkyl bond or be electrochemically reduced (but the formal potential for reducing this alkyl radical is unknown to the best of our knowledge).

$$Co^{II}TPP + NC(CH_2)_3\cdot \rightarrow NC(CH_2)_3\text{–}Co^{III}TPP \text{ (if outer-sphere electron transfer is in operation)}$$

$$[Cl\text{–}Co^{II}TPP]^{-} + NC(CH_2)_3\cdot \rightarrow NC(CH_2)_3\text{–}Co^{III}TPP + Cl^{-} \text{ (if halogen-atom abstraction is in operation)}$$

$NC(CH_2)_3\cdot + e^- \rightarrow NC(CH_2)_3^-$ (if $NC(CH_2)_3\cdot$ is electrochemically reduced)

Regarding the irreversible reductive shoulder peak anodic of the $Co^{II/I}$ redox, we reason that it is unlikely to result from electroreduction of $NC(CH_2)_3\cdot$. If $NC(CH_2)_3\cdot$ is electrochemically reduced, its recombination with Co^{II}TPP would be suppressed and catalytic regeneration of Co^{II}TPP would be facilitated, so we would expect a pronounced catalytic wave rather than a shoulder peak. Instead, here is our proposed explanation for the shoulder peak: (1) near the foot of the Co^{II}TPP reduction wave, generation of $NC(CH_2)_3\cdot$ is relatively slow in rate, so that catalytic regeneration of Co^{II}TPP is relatively less affected by recombination between $NC(CH_2)_3\cdot$ and Co^{II}TPP; (2) as the potential is swept cathodically, generation of $NC(CH_2)_3\cdot$ increases in rate, so that catalytic regeneration of Co^{II}TPP is increasingly suppressed by recombination between $NC(CH_2)_3\cdot$ and Co^{II}TPP, resulting in a leveling-off of the catalytic current and thus a shoulder peak; (3) as the potential is further swept cathodically and passes the formal potential of the $Co^{II/I}$ redox, an intensified reductive peak of Co^{II}TPP is observed, due to the much thinner reaction-diffusion layer compared to the diffusion layer[38].

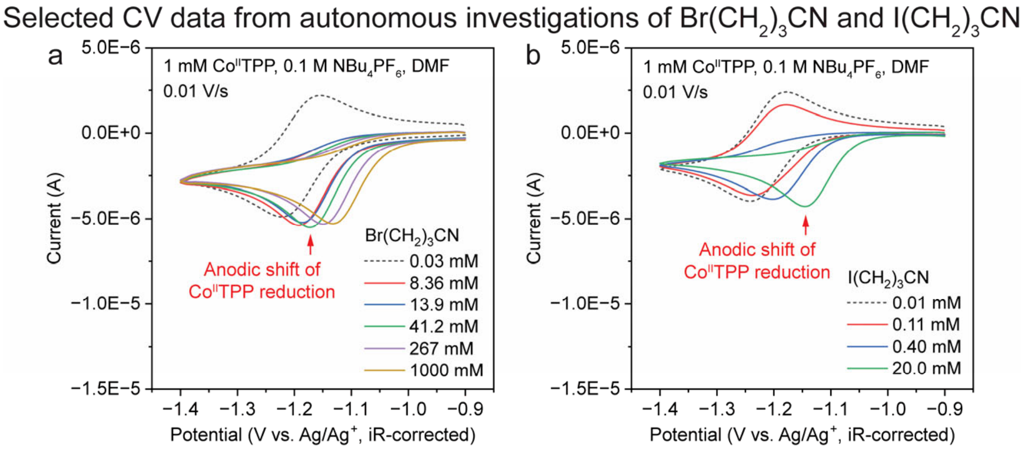

Supplementary Fig. 71. The CV responses of CoTPP with $Br(CH_2)_3CN$ and $I(CH_2)_3CN$. Selected CV data from autonomous investigations by the closed-loop workflow using (a) $Br(CH_2)_3CN$ or (b) $I(CH_2)_3CN$.

To shed light on whether the proposed one-electron process between $Cl(CH_2)_3CN$ and Co^{I}TPP undergoes an outer-sphere electron transfer pathway or a halogen-atom abstraction pathway, we further examine the CV responses of its bromide ($Br(CH_2)_3CN$) and iodide ($I(CH_2)_3CN$) counterparts (Supplementary Fig. 71). Both $Br(CH_2)_3CN$ and $I(CH_2)_3CN$ react with CoTPP following a prototypical *EC* mechanism, where an increase in [RX] leads to an anodic shift of the reductive peak of Co^{II}TPP with little change in peak height[11, 12]. Given that the general reactivity trend for halogen-atom abstraction is iodide > bromide > chloride[39], if a halogen-atom abstraction pathway is in operation, $Br(CH_2)_3CN$ and $I(CH_2)_3CN$ would be expected to deviate from an *EC* mechanism to a greater extent than $Cl(CH_2)_3CN$ does, inconsistent with our observations. With a halogen-atom abstraction pathway disfavored, we hypothesize that $Cl(CH_2)_3CN$ is likely to undergo an outer-sphere electron transfer pathway with Co^{I}TPP. In fact, a competition between outer-sphere electron transfer and S_N2 pathways has been documented in literature[40-42], so that $Cl(CH_2)_3CN$ can be considered as a borderline case to reflect such mechanistic competition.

Supplementary References

(1) Rodríguez, O., Pence, M. A. & Rodríguez-López, J. Hard Potato: a Python library to control commercial potentiostats and to automate electrochemical experiments. *Anal. Chem.* **95**, 4840–4845 (**2023**).

(2) Paria, B., Kandasamy, K. & Póczos, B. A flexible framework for multi-objective bayesian optimization using random scalarizations. In *Proceedings of The 35th Uncertainty in Artificial Intelligence Conference* (eds Ryan, P. A. & Vibhav, G.) **115**, 766 (PMLR: Proceedings of Machine Learning Research, 2020).

(3) Kandasamy, K. et al. Tuning hyperparameters without grad students: Scalable and robust bayesian optimisation with dragonfly. *J. Mach. Learn. Res.* **21**, 3098–3124 (**2020**).

(4) Reglo ICC Operating Manual. https://pim-resources.coleparmer.com/literature/14-036-e-ismatec-reglo-icc-english-rev-c.pdf (accessed August 2023).

(5) Pump Tubing Selection Guide. https://shop12456.hstatic.dk/upload_dir/shop/slanger/Masterflex-tubing-selection-guide-2021.pdf (accessed August 2023).

(6) Masterflex Tubing Chemical Compatibility Tables. https://envcoglobal.com/wp-content/uploads/2014/10/masteflex-chemchart3.pdf (accessed August 2023).

(7) Hoar, B. B. et al. Electrochemical Mechanistic Analysis from Cyclic Voltammograms Based on Deep Learning. *ACS Meas. Sci. Au* **2**, 595–604 (**2022**).

(8) Nicholson, R. S. & Shain, I. Theory of stationary electrode polarography. Single scan and cyclic methods applied to reversible, irreversible, and kinetic systems. *Anal. Chem.* **36**, 706–723 (**1964**).

(9) Nicholson, R. S. Semiempirical procedure for measuring with stationary electrode polarography rates of chemical reactions involving the product of electron transfer. *Anal. Chem.* **38**, 1406 (**1966**).

(10) Zanello, P. & Connelly, N. G. *Inorganic Electrochemistry: Theory, Practice and Applications* (The Royal Society of Chemistry, 2003).

(11) Savéant, J.-M. & Costentin, C. *Elements of Molecular and Biomolecular Electrochemistry: An Electrochemical Approach to Electron Transfer Chemistry*, 2nd ed (John Wiley & Sons, 2019).

(12) Costentin, C. et al. Concertedness in proton-coupled electron transfer cleavages of carbon–metal bonds illustrated by the reduction of an alkyl cobalt porphyrin. *Chem. Sci.* **4**, 819–823 (**2013**).

(13) Anslyn, E. & Dougherty, D. *Modern Physical Organic Chemistry* (University Science Books, 2006).

(14) Hansch, C., Leo, A. & Taft, R. W. A survey of Hammett substituent constants and resonance and field parameters. *Chem. Rev.* **91**, 165–195 (**1991**).

(15) Jiang, X. & Ji, G. A self-consistent and cross-checked scale of spin-delocalization substituent constants, the $\sigma^{\bullet}_{JJ}$ scale. *J. Org. Chem.* **57**, 6051–6056 (**1992**).

(16) Ağirbas, H. & Jackson, R. A. Free radical reactions in solution. Part 9. Further $\sigma^{\bullet}$ values from decomposition of substituted dibenzylmercurials. *J. Chem. Soc., Perkin Trans. 2*, 739–742 (**1983**).

(17) Dust, J. M. & Arnold, D. R. Substituent effects on benzyl radical ESR hyperfine coupling constants. The $\sigma_{\alpha}^{\bullet}$ scale based upon spin delocalization. *J. Am. Chem. Soc.* **105**, 1221–1227 (**1983**).

(18) Creary, X. Super radical stabilizers. *Acc. Chem. Res.* **39**, 761–771 (**2006**).

(19) Fisher, T. H. & Meierhoefer, A. W. Substituent effects in free-radical reactions. A study of 4-substituted 3-cyanobenzyl free radicals. *J. Org. Chem.* **43**, 224–228 (**1978**).

(20) Dinçtürk, S. & Jackson, R. A. Free radical reactions in solution. Part 7. Substituent effects on free radical reactions: comparison of the $\sigma^{\bullet}$ scale with other measures of radical stabilization. *J. Chem. Soc., Perkin Trans. 2*, 1127–1131 (**1981**).

(21) Swain, C. G. & Langsdorf, W. P., Jr. Concerted displacement reactions. VI. m- and p-substituent effects as evidence for a unity of mechanism in organic halide reactions. *J. Am. Chem. Soc.* **73**, 2813–2819 (**1951**).

(22) Hudson, R. F. & Klopman, G. 198. Nucleophilic reactivity. Part II. The reaction between substituted thiophenols and benzyl bromides. *J. Chem. Soc.* **1962**, 1062–1067 (**1962**).

(23) Ballistreri, F. P., Maccarone, E. & Mamo, A. Kinetics and mechanism of benzylation of anilines. *J. Org. Chem.* **41**, 3364–3367 (**1976**).

(24) Young, P. R. & Jencks, W. P. Separation of polar and resonance substituent effects in the reactions of acetophenones with bisulfite and of benzyl halides with nucleophiles. *J. Am. Chem. Soc.* **101**, 3288–3294 (**1979**).

(25) Bordwell, F. G. & Hughes, D. L. Hammett and Bronsted-type relationships in reactions of 9-substituted fluorenyl anions with benzyl halides. *J. Org. Chem.* **45**, 3320–3325 (**1980**).

(26) Sandford, C. et al. Mechanistic studies into the oxidative addition of Co(I) complexes: combining electroanalytical techniques with parameterization. *J. Am. Chem. Soc.* **141**, 18877–18889 (**2019**).

(27) Tang, T. et al. Analyzing mechanisms in Co(I) redox catalysis using a pattern recognition platform. *Chem. Sci.* **12**, 4771–4778 (**2021**).

(28) Lin, Q. et al. Monovalent nickel-mediated radical formation: a concerted halogen-atom dissociation pathway determined by electroanalytical studies. *J. Am. Chem. Soc.* **143**, 14196–14206 (**2021**).

(29) Sandford, C. et al. Mechanistic studies into the oxidative addition of Co(I) complexes: combining electroanalytical techniques with parameterization. *J. Am. Chem. Soc.* **141**, 18877–18889 (**2019**).

(30) Malapit, C. A. et al. Advances on the merger of electrochemistry and transition metal catalysis for organic synthesis. *Chem. Rev.* **122**, 3180–3218 (**2022**).

(31) Doyle, M. P. et al. Outer-sphere one-electron reductions of arenediazonium salts. *J. Am. Chem. Soc.* **109**, 1536–1540 (**1987**).

(32) Aubart, M. A. & Bergman, R. G. Reaction of organic disulfides with cobalt-centered metal radicals. Use of the E- and C-Based dual-parameter substituent model and quantitative solvent effect analyses to compare outer-sphere and inner-sphere electron-transfer processes. *J. Am. Chem. Soc.* **120**, 8755–8766 (**1998**).

(33) Tang, T. et al. Analyzing mechanisms in Co(I) redox catalysis using a pattern recognition platform. *Chem. Sci.* **12**, 4771–4778 (**2021**).

(34) Tang, T. et al. Comparing halogen atom abstraction kinetics for Mn(I), Fe(I), Co(I), and Ni(I) complexes by combining electroanalytical and statistical modeling. *Eur. J. Org. Chem.*, e202200064 (**2022**).

(35) Schrauzer, G. N. & Deutsch, E. Reactions of cobalt(I) supernucleophiles. The alkylation of vitamin B12s, cobaloximes(I), and related compounds. *J. Am. Chem. Soc.* **91**, 3341–3350 (**1969**).

(36) Streitwieser, A., Jr. Solvolytic displacement reactions at saturated carbon atoms. *Chem. Rev.* **56**, 571–752 (**1956**).

(37) Halpern, J. & Maher, J. P. Kinetics of the reactions of pentacyanocobaltate(II) with organic halides. *J. Am. Chem. Soc.* **87**, 5361–5366 (**1965**).

(38) Costentin, C. & Savéant, J.-M. Homogeneous molecular catalysis of electrochemical reactions: manipulating intrinsic and operational factors for catalyst improvement. *J. Am. Chem. Soc.* **140**, 16669–16675 (**2018**).

(39) Juliá, F., Constantin, T. & Leonori, D. Applications of halogen-atom transfer (XAT) for the generation of carbon radicals in synthetic photochemistry and photocatalysis. *Chem. Rev.* **122**, 2292–2352 (**2022**).

(40) Lexa, D., Mispelter, J. & Saveant, J. M. Electroreductive alkylation of iron in porphyrin complexes. Electrochemical and spectral characteristics of *s*-alkylironporphyrins. *J. Am. Chem. Soc.* **103**, 6806–6812 (**1981**).

(41) Lexa, D. et al. Single electron transfer and nucleophilic substitution. Reaction of alkyl bromides with aromatic anion radicals and low-oxidation-state iron porphyrins. *J. Am. Chem. Soc.* **110**, 7617–7625 (**1988**).

(42) Maiya, G. B., Han, B. C. & Kadish, K. M. Electrochemical studies of cobalt-carbon bond formation. A kinetic investigation of the reaction between (tetraphenylporphinato)cobalt(I) and alkyl halides. *Langmuir* **5**, 645–650 (**1989**).
