# Supplementary material for: Autonomous closed-loop mechanistic investigation of molecular electrochemistry via automation
Source: Nat Commun. 2024 Mar 30;15:2781. doi: 10.1038/s41467-024-47210-x (PMC10981680; doi:10.1038/s41467-024-47210-x)
Supplement: Supplementary file 3 — Description of Additional Supplementary Files [file 41467_2024_47210_MOESM3_ESM.pdf]

Description of Additional Supplementary Files

Title: Supplementary Software

Description: Source Python code
